# Supplementary material for: Copper-catalyzed regio- and stereo-selective hydrosilylation of terminal allenes to access (E)-allylsilanes
Source: Nat Commun. 2022 Jun 27;13:3691. doi: 10.1038/s41467-022-31458-2 (PMC9237096; doi:10.1038/s41467-022-31458-2)
Supplement: Supplementary file 1 — Supplementary information [file 41467_2022_31458_MOESM1_ESM.pdf]

Supplementary Information

Copper-catalyzed regio- and stereo-selective hydrosilylation of terminal allenes to access (*E*)-allylsilanes

Shaowei Chen,[1] Xiaoqian He,[2] Yi Jin,[1] Yu Lan, [2,3]* and Xiao Shen[1]*

[1]The Institute for Advanced Studies, Engineering Research Center of Organosilicon Compounds & Materials (Ministry of Education), Wuhan University, 430072, Wuhan, P. R. China. [2]School of Chemistry and Chemical Engineering, Chongqing Key Laboratory of Theoretical and Computational Chemistry, Chongqing University, Chongqing 400030, PR China. [3]College of Chemistry and Molecular Engineering, Zhengzhou University, Zhengzhou 450001, PR China.

*E-mail: xiaoshen@whu.edu.cn; lanyu@cqu.edu.cn.

Contents

1. Supplementary Methods……………………………………………..………………2
  1.1 General information…………………………………………………….……….2
  1.2 Preparation of substrates used in this work…………………….……………..2
  1.3 General Procedure for the Hydrosilylation of 1,1-disubsitituted Allenes……10
  1.4 General Procedure for the Hydrosilylation of Monosubstituted Allenes…….23
  1.5 Synthetic transformations ………………………………………………33
  1.6 Mechanism study……………………….……………………………………...35
2. Supplementary Figures…………………………..…………………………39
  2.1 Spectra data…………………….……………………………………………39
3. Supplementary References…..…………………………………………………100

1. Supplementary Methods

1.1 General information

Chromatography: HaiLang Silica Flash P60 size 40-63 µm (200-300 mesh), TLC: HaiLang silica gel 60 (0.25mm). Visualization of the chromatogram was performed by UV, I2. Mass spectra were recorded on Bruker UltiMate 3000 & Compact, Thermo ISQ LT, LTQ XL and VELOS pro & ORBITRIP mass spectrometers. ^{1}H, ^{13}C, ^{19}F were recorded on Bruker 400 and JNM-ECZ 400 using $CDCl_3$ as solvent. Chemical shift values are reported in ppm with the solvent resonance as the internal standard ($CDCl_3$: δ 7.26 for ^{1}H, δ 77.16 for ^{13}C). Data are reported as follows: chemical shifts, multiplicity (s = singlet, bs = broad singlet, d = doublet, dd = doublet of doublets, t = triplet, td = triplet of doublets, m = multiplet), coupling constants (Hz), and integration. Infrared spectra were recorded on an Agilent Technologies Cary 630 FTIR and wavelengths are reported in cm^{-1}. Melting point was measured by INESA SGW X-4. All reagents were used as received and solvents were dried and degassed according to standard procedure. Reactions were carried out under N_2 atmosphere unless otherwise noted. $Cu(OAc)_2$ was purchased from Alfa, Xantphos was purchased from Laajoo. Ph_2SiH_2 was purchased from Adamas.

1.2 Preparation of substrates used in this work

Allenes were synthesized according to reported procedures[1~6].

General Procedure (GP1) for the preparation of allenes:

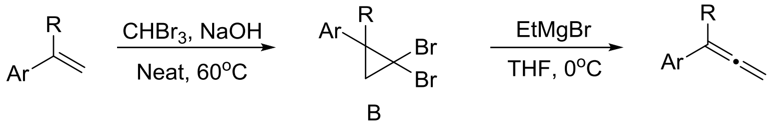

Under N_2, to a mixture of styrene (20 mmol, 1.0 equiv.), $CHBr_3$ (8g, 32 mmol, 1.6 equiv.) and triethylbenzylammonium chloride (45.6 mg, 0.2 mmol, 0.01 equiv.) was added dropwise a 50% aq. NaOH (6.4 g, 80 mmol, 4.0 equiv.) over 1 h. The resulting mixture was stirred vigorously at 60 ℃ for 24 h, cooled to room temperature, followed by addition of water (30 mL). The mixture was extracted with ethyl acetate (30 mL x 3), and the combined organic phases were dried over sodium sulfate, and concentrated. The crude product was purified with column chromatography on silica gel (200~300 mesh) to afford B. To a stirred solution of B (6 mmol, 1.0 equiv.) in dry THF (6 mL) at 0 ℃ was added dropwise EtMgBr (4.5 mL, 9 mmol, 2.0 M in THF, 1.5 equiv.) under nitrogen over 30 min. The resulting mixture was stirred at room temperature for 2 h, quenched with 3 M hydrochloric acid solution and the mixture was diluted with ethyl ether (50 mL). The organic phase was washed with water (50 mL), dried over magnesium sulfate, and concentrated. The crude product was purified with column chromatography on silica gel (200~300 mesh) to afford allene.

General Procedure (GP2) for the preparation of allenes:

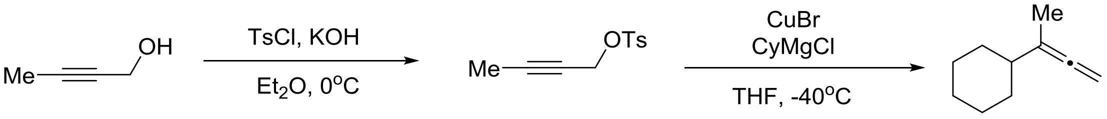

To a solution of 2-butyn-1-ol (700 mg, 10 mmol) in diethyl ether (20 mL) at 0 °C was added *p*-toluenesulfonyl chloride (2.3 g, 12 mmol) and crushed KOH (3.4 g) sequentially. The resulting mixture was stirred at 0 °C for 1 h before it was poured into an ice-water mixture. Ethyl ether (20

mL) was added and the organic phase was separated, dried over magnesium sulfate, and concentrated to give propargyl tosylate as a light yellow oil. To this oil in a dried 100 mL 3-necked flask was added CuBr (144 mg, 1 mmol) and THF (20 mL). To the mixture at -40 °C was charged dropwise CyMgCl (2.0 M in ethyl ether, 5.5 mL, 11 mmol) over 10 min. The resulting mixture was stirred at the same temperature for 3.5 h before it was quenched by saturated NH_4Cl solution (10 mL). Ethyl ether (20 mL) was added and the organic layer was separated, dried over magnesium sulfate, and concentrated. The residue was purified by column chromatography over silica gel with *n*-pentane as eluent to give buta-2,3-dien-2-ylcyclohexane.

Buta-2,3-dien-2-ylbenzene (1a)

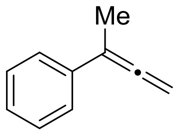

The title compound was prepared following the general procedure **GP1**. Purification by flash column chromatography (Pentane) gave the title compound as a colourless oil (663 mg, 85% yield). The spectroscopic data for this product matched the literature data[1]. ^{1}H NMR (400 MHz, $CDCl_3$, 25 °C) δ 7.46–7.40 (m, 2H), 7.34 (t, *J* = 7.7 Hz, 2H), 7.24–7.17 (m, 1H), 5.04 (q, *J* = 3.2 Hz, 2H), 2.11 (t, *J* = 3.2 Hz, 3H).

1-(buta-2,3-dien-2-yl)-4-methylbenzene (1b)

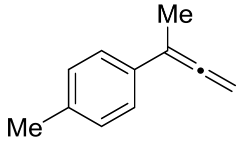

The title compound was prepared following the general procedure **GP1**. Purification by flash column chromatography (Pentane) gave the title compound as a colourless oil (691 mg, 80% yield). The spectroscopic data for this product matched the literature data[1]. ^{1}H NMR (400 MHz, $CDCl_3$, 25 °C) δ 7.31 (d, *J* = 8.2 Hz, 2H), 7.15 (d, *J* = 8.5 Hz, 1H), 5.01 (q, *J* = 3.2 Hz, 2H), 2.35 (s, 3H), 2.09 (t, *J* = 3.2 Hz, 3H).

1-(buta-2,3-dien-2-yl)-3-methylbenzene (1c)

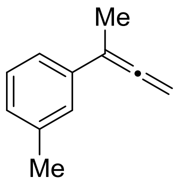

The title compound was prepared following the general procedure **GP1**. Purification by flash column chromatography (Pentane) gave the title compound as a colourless oil (639 mg, 74% yield). The spectroscopic data for this product matched the literature data[1]. ^{1}H NMR (400 MHz, $CDCl_3$, 25 °C) δ 7.26–7.19 (m, 3H), 7.10–6.97 (m, 1H), 5.03 (q, *J* = 3.2 Hz, 2H), 2.37 (s, 3H), 2.11 (t, *J* = 3.2 Hz, 3H).

1-(buta-2,3-dien-2-yl)-4-ethylbenzene (1d)

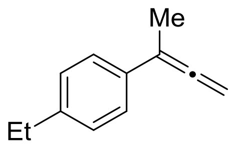

The title compound was prepared following the general procedure **GP1**. Purification by flash column chromatography (Pentane) gave the title compound as a colourless oil (862 mg, 91% yield). The spectroscopic data for this product matched the literature data[1]. ^{1}H NMR (400 MHz, $CDCl_3$, 25 °C) δ 7.33 (d, J = 8.3 Hz, 2H), 7.17 (d, J = 8.5 Hz, 2H), 5.01 (q, J = 3.1 Hz, 2H), 2.64 (q, J = 7.6 Hz, 2H), 2.09 (t, J = 3.2 Hz, 3H), 1.23 (t, J = 7.6 Hz, 3H).

1-(buta-2,3-dien-2-yl)-4-(tert-butyl)benzene (1e)

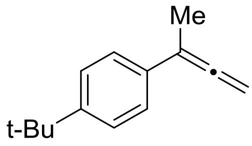

The title compound was prepared following the general procedure **GP1**. Purification by flash column chromatography (Pentane) gave the title compound as a colourless oil (862 mg, 91% yield). The spectroscopic data for this product matched the literature data[3]. ^{1}H NMR (400 MHz, $CDCl_3$, 25 °C) δ 7.40–7.33 (m, 4H), 5.01 (p, J = 2.9 Hz, 2H), 2.12–2.07 (m, 3H), 1.33 (s, 9H).

1-(buta-2,3-dien-2-yl)-4-fluorobenzene (1f)

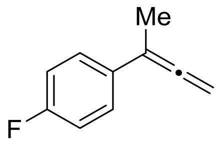

The title compound was prepared following the general procedure **GP1**. Purification by flash column chromatography (Pentane) gave the title compound as a colourless oil (577 mg, 65% yield). The spectroscopic data for this product matched the literature data[1]. ^{1}H NMR (400 MHz, $CDCl_3$, 25 °C) δ 7.52–7.33 (m, 2H), 7.24–7.08 (m, 2H), 5.05 (q, J = 3.2 Hz, 2H), 2.09 (t, J = 3.2 Hz, 3H).

1-(buta-2,3-dien-2-yl)-2-fluorobenzene (1g)

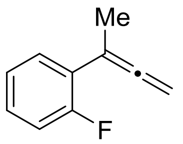

The title compound was prepared following the general procedure **GP1**. Purification by flash column chromatography (Pentane) gave the title compound as a colourless oil (621 mg, 70% yield). The spectroscopic data for this product matched the literature data[1]. ^{1}H NMR (400 MHz, $CDCl_3$, 25 °C) δ 7.30 (td, J = 7.9, 1.9 Hz, 1H), 7.24–7.16 (m, 1H), 7.10 (td, J = 7.5, 1.3 Hz, 1H), 7.03 (ddd, J = 11.4, 8.1, 1.4 Hz, 1H), 4.91 (q, J = 3.2 Hz, 2H), 2.12 (td, J = 3.2, 1.8 Hz, 3H).

1-(buta-2,3-dien-2-yl)-3-chlorobenzene (1i)

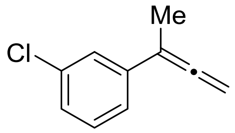

The title compound was prepared following the general procedure **GP1**. Purification by flash column chromatography (Pentane) gave the title compound as a colourless oil (836 mg, 85% yield). The spectroscopic data for this product matched the literature data[1]. ^{1}H NMR (400 MHz, $CDCl_3$, 25 ℃) δ 7.38 (s, 1H), 7.31–7.22 (m, 2H), 7.21–7.13 (m, 1H), 5.06 (q, J = 3.2 Hz, 2H), 2.07 (t, J = 3.2 Hz, 3H).

1-(buta-2,3-dien-2-yl)-4-chlorobenzene (1j)

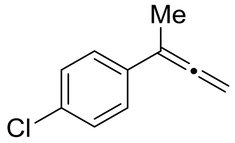

The title compound was prepared following the general procedure **GP1**. Purification by flash column chromatography (Pentane) gave the title compound as a colourless oil (816 mg, 83% yield). The spectroscopic data for this product matched the literature data[1]. ^{1}H NMR (400 MHz, $CDCl_3$, 25 ℃) δ 7.39–7.10 (m, 4H), 5.04 (q, J = 3.2 Hz, 2H), 2.07 (t, J = 3.2 Hz, 3H).

1-bromo-3-(buta-2,3-dien-2-yl)benzene (1k)

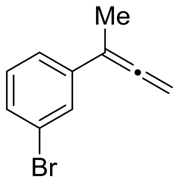

The title compound was prepared following the general procedure **GP1**. Purification by flash column chromatography (Pentane) gave the title compound as a colourless oil (1.07g, 86% yield). The spectroscopic data for this product matched the literature data[4]. ^{1}H NMR (400 MHz, $CDCl_3$, 25 ℃) δ 7.53 (t, J = 1.9 Hz, 1H), 7.35–7.29 (m, 2H), 7.22–7.13 (m, 1H), 5.07 (q, J = 3.2 Hz, 2H), 2.07 (t, J = 3.2 Hz, 3H).

1-(buta-2,3-dien-2-yl)-3-(trifluoromethyl)benzene (1l)

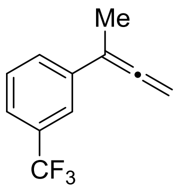

The title compound was prepared following the general procedure **GP1**. Purification by flash column chromatography (Pentane) gave the title compound as a colourless oil (891 mg, 75% yield). The spectroscopic data for this product matched the literature data[2]. ^{1}H NMR (400 MHz, $CDCl_3$, 25 ℃) δ 7.63 (s, 1H), 7.57 (d, J = 8.3 Hz, 1H), 7.43 (q, J = 8.1 Hz, 2H), 5.09 (q, J = 3.1 Hz, 2H), 2.11 (t, J = 3.2 Hz, 3H).

2-(buta-2,3-dien-2-yl)thiophene (1n)

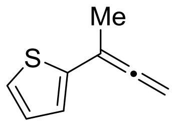

The title compound was prepared following the general procedure **GP1**. Purification by flash column chromatography (Pentane) gave the title compound as a colourless oil (685 mg, 84% yield). The spectroscopic data for this product matched the literature data[5]. ^{1}H NMR (400 MHz, $CDCl_3$, 25 ℃) δ 7.18 (d, *J* = 5.1 Hz, 1H), 7.01–6.95 (m, 1H), 6.94–6.88 (m, 1H), 5.04 (q, *J* = 3.1 Hz, 2H), 2.11 (t, *J* = 3.1 Hz, 3H).

2-(buta-2,3-dien-2-yl)naphthalene (1o)

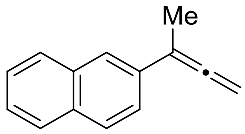

The title compound was prepared following the general procedure **GP1**. Purification by flash column chromatography (Pentane) gave the title compound as a colourless oil (853 mg, 79% yield). The spectroscopic data for this product matched the literature data[1]. ^{1}H NMR (600 MHz, $CDCl_3$, 25 ℃) δ 7.84–7.70 (m, 4H), 7.67–7.61 (m, 1H), 7.49–7.38 (m, 2H), 5.11 (q, *J* = 3.1 Hz, 2H), 2.22 (t, *J* = 3.1 Hz, 3H).

5-(buta-2,3-dien-2-yl)benzo[d][1,3]dioxole (1r)

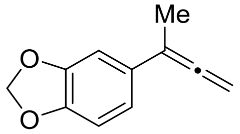

The title compound was prepared following the general procedure **GP1**. Purification by flash column chromatography (Pentane:EA=100:1) gave the title compound as a colourless oil (1.17 g, 89% yield). The spectroscopic data for this product matched the literature data[4]. ^{1}H NMR (400 MHz, $CDCl_3$, 25 ℃) δ 6.94 (d, *J* = 1.8 Hz, 1H), 6.85 (dd, *J* = 8.1, 1.8 Hz, 1H), 6.78 (d, *J* = 8.1 Hz, 1H), 5.95 (s, 2H), 5.01 (q, *J* = 3.2 Hz, 2H), 2.05 (t, *J* = 3.2 Hz, 3H).

1-(Buta-2,3-dien-2-yl)-4-(trifluoromethoxy)benzene (1t)

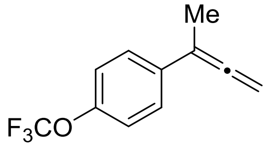

The title compound was prepared following the general procedure **GP1**. Purification by flash column chromatography (Pentane:EA=100:1) gave the title compound as a colourless oil (1.17 g, 89% yield).^{1}H NMR (400 MHz, $CDCl_3$, 25 ℃) δ 7.45–7.39 (m, 2H), 7.20–7.13 (m, 2H), 5.05 (q, *J* = 3.2 Hz, 2H), 2.09 (t, *J* = 3.2 Hz, 3H); ^{13}C NMR (151 MHz, $CDCl_3$, 25 ℃) δ 209.2, 148.0, 135.7, 127.0, 121.0, 120.6 (t, *J* = 257.2 Hz), 99.0, 77.5, 16.9; IR (ATR): 3046, 2989,2930, 2863, 1946, 1588, 1506, 1431, 1375, 1260, 1163, 1066, 1018, 849, 809 cm^{-1}; HRMS (ESI, m/z): calcd for.

$C_{11}H_{10}F_3O_1^+$ $(M+H)^+$: 215.0678; Found: 215.0684.

(4-(Buta-2,3-dien-2-yl)phenyl)(methyl)sulfane (1u)

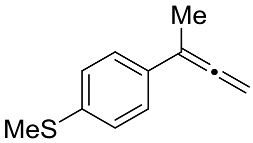

The title compound was prepared following the general procedure **GP1**. Purification by flash column chromatography (Pentane) gave the title compound as a colourless oil (535 mg, 82% yield). ^{1}H NMR (400 MHz, $CDCl_3$, 25 ºC) δ 7.35–7.31 (m, 2H), 7.24–7.20 (m, 2H), 5.03 (q, *J* = 3.1 Hz, 2H), 2.48 (s, 3H), 2.08 (t, *J* = 3.2 Hz, 3H); ^{13}C NMR (151 MHz, $CDCl_3$, 25 ºC) δ 209.0, 136.6, 133.8, 126.9, 126.2, 99.5, 16.8, 16.2; IR (ATR): 2982, 2922, 2855, 1942, 1677, 1592, 1491, 1424, 1392, 1320, 1264, 1100, 1014, 854, 820 cm^{-1}; HRMS (ESI, m/z): calcd for. $C_{11}H_{13}S_1^+$ $(M+H)^+$: 177.0733; Found: 177.0734.

Hexa-1,2-dien-3-ylbenzene (1v)

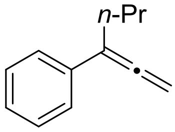

The title compound was prepared following the general procedure **GP1**. Purification by flash column chromatography (Pentane) gave the title compound as a colourless oil (711 mg, 75% yield). The spectroscopic data for this product matched the literature data[5]. ^{1}H NMR (400 MHz, $CDCl_3$, 25 ℃) δ 7.41 (d, *J* = 8.5 Hz, 2H), 7.36–7.29 (m, 2H), 7.23–7.15 (m, 1H), 5.07 (t, *J* = 3.3 Hz, 2H), 2.45–2.33 (m, 2H), 1.64–1.52 (m, 2H), 0.99 (t, *J* = 7.4 Hz, 3H).

Buta-2,3-dien-2-ylcyclohexane (1w)

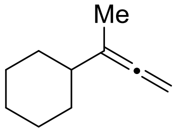

The title compound was prepared following the general procedure **GP2**. Purification by flash column chromatography (Pentane) gave the title compound as a colourless oil (856 mg, 63% yield for two steps). The spectroscopic data for this product matched the literature data[4]. ^{1}H NMR (600 MHz, $CDCl_3$, 25 ℃) δ 4.59 (p, *J* = 3.0 Hz, 2H), 1.84–1.60 (m, 9 H), 1.35–1.04 (m, 5H).

(4-methylpenta-1,2-dien-3-yl)benzene (1ad)

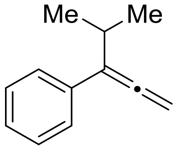

The title compound was prepared following the general procedure **GP1**. Purification by flash column chromatography (Pentane) gave the title compound as a colourless oil (673 mg, 71% yield). The spectroscopic data for this product matched the literature data[6]. ^{1}H NMR (400 MHz, $CDCl_3$, 25

℃) δ 7.40 (d, J = 7.7 Hz, 2H), 7.36–7.15 (m, 3H), 5.08 (d, J = 2.7 Hz, 2H), 2.87–2.73 (m, 1H), 1.14 (d, J = 6.7 Hz, 6H).

Propa-1,2-dien-1-ylbenzene (4a)

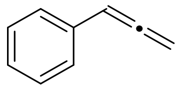

The title compound was prepared following the general procedure **GP1**. Purification by flash column chromatography (Pentane) gave the title compound as a colourless oil (626 mg, 90% yield). The spectroscopic data for this product matched the literature data[5]. ^{1}H NMR (400 MHz, $CDCl_3$, 25 ℃) δ 7.45–7.44 (m, 4H), 7.36–7.32 (m, 1H), 6.31 (t, J = 6.4 Hz, 1H), 5.28 (d, J = 6.8 Hz, 2H).

1-methyl-3-(propa-1,2-dien-1-yl)benzene (4b)

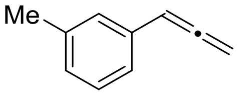

The title compound was prepared following the general procedure **GP1**. Purification by flash column chromatography (Pentane) gave the title compound as a colourless oil (624 mg, 80% yield). The spectroscopic data for this product matched the literature data[5]. ^{1}H NMR (400 MHz, $CDCl_3$, 25 ℃) δ 7.30 (t, J = 7.5 Hz, 1H), 7.25–7.17 (m, 2H), 7.11 (d, J = 7.5 Hz, 1H), 6.24 (t, J = 6.8 Hz, 1H), 5.23 (d, J = 6.8 Hz, 2H), 2.43 (s, 3H).

1-methyl-4-(propa-1,2-dien-1-yl)benzene (4c)

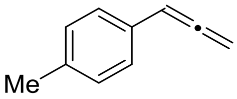

The title compound was prepared following the general procedure **GP1**. Purification by flash column chromatography (Pentane) gave the title compound as a colourless oil (585 mg, 75% yield). The spectroscopic data for this product matched the literature data[5]. ^{1}H NMR (400 MHz, $CDCl_3$, 25 ℃) δ 7.20 (d, J = 8.1 Hz, 2H), 7.12 (d, J = 6.5 Hz, 2H), 6.14 (t, J = 6.8 Hz, 1H), 5.13 (d, J = 6.8 Hz, 2H), 2.33 (s, 3H).

1-methyl-2-(propa-1,2-dien-1-yl)benzene (4d)

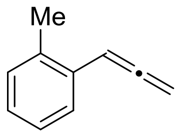

The title compound was prepared following the general procedure **GP1**. Purification by flash column chromatography (Pentane) gave the title compound as a colourless oil (507 mg, 65% yield). The spectroscopic data for this product matched the literature data[5]. ^{1}H NMR (400 MHz, $CDCl_3$, 25 ℃) δ 7.38 (d, J = 7.5 Hz, 1H), 7.22–7.02 (m, 3H), 6.34 (t, J = 6.8 Hz, 1H), 5.10 (d, J = 7.0 Hz, 2H), 2.34 (s, 3H).

1-(tert-butyl)-4-(propa-1,2-dien-1-yl)benzene (4e)

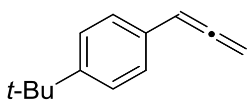

The title compound was prepared following the general procedure **GP1**. Purification by flash column chromatography (Pentane) gave the title compound as a colourless oil (763 mg, 74% yield). The spectroscopic data for this product matched the literature data[5]. ^{1}H NMR (400 MHz, $CDCl_3$, 25 ℃) δ 7.43 (d, *J* = 8.1 Hz, 2H), 7.33 (d, *J* = 8.0 Hz, 2H), 6.24 (t, *J* = 6.9 Hz, 1H), 5.21 (d, *J* = 6.9 Hz, 2H), 1.40 (s, 9H).

1-methoxy-4-(propa-1,2-dien-1-yl)benzene (4f)

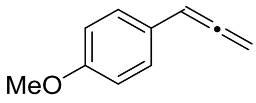

The title compound was prepared following the general procedure **GP1**. Purification by flash column chromatography (Pentane:EA=100:1) gave the title compound as a colourless oil (639 mg, 73% yield). The spectroscopic data for this product matched the literature data[5]. ^{1}H NMR (400 MHz, $CDCl_3$, 25 ℃) δ 7.15 (d, *J* = 7.4 Hz, 2H), 6.78 (d, *J* = 8.8 Hz, 2H), 6.05 (t, *J* = 6.8 Hz, 1H), 5.04 (d, *J* = 6.8 Hz, 2H), 3.72 (s, 3H).

1-fluoro-4-(propa-1,2-dien-1-yl)benzene (4h)

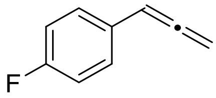

The title compound was prepared following the general procedure **GP1**. Purification by flash column chromatography (Pentane) gave the title compound as a colourless oil (578 mg, 72% yield). The spectroscopic data for this product matched the literature data[5]. ^{1}H NMR (400 MHz, $CDCl_3$, 25 ℃) δ 7.31–7.16 (m, 2H), 7.06–6.93 (m, 2H), 6.13 (t, *J* = 6.8 Hz, 1H), 5.15 (d, *J* = 6.8 Hz, 2H).

1-fluoro-3-(propa-1,2-dien-1-yl)benzene (4i)

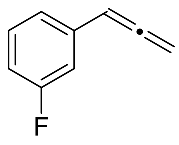

The title compound was prepared following the general procedure **GP1**. Purification by flash column chromatography (Pentane) gave the title compound as a colourless oil (651 mg, 81% yield). The spectroscopic data for this product matched the literature data[5]. ^{1}H NMR (400 MHz, $CDCl_3$, 25 ℃) δ 7.29–7.20 (m, 1H), 7.11–6.98 (m, 2H), 6.96–6.85 (m, 1H), 6.14 (t, *J* = 6.8 Hz, 1H), 5.18 (d, *J* = 6.9 Hz, 2H).

1-chloro-4-(propa-1,2-dien-1-yl)benzene (4k)

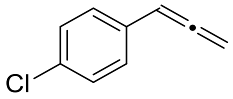

The title compound was prepared following the general procedure **GP1**. Purification by flash

column chromatography (Pentane) gave the title compound as a colourless oil (657 mg, 73% yield). The spectroscopic data for this product matched the literature data[5]. ^{1}H NMR (400 MHz, $CDCl_3$, 25 ℃) δ 7.30–7.24 (m, 2H), 7.24–7.19 (m, 2H), 6.12 (t, *J* = 6.8 Hz, 1H), 5.16 (d, *J* = 6.8 Hz, 2H).

1-chloro-2-(propa-1,2-dien-1-yl)benzene (4l)

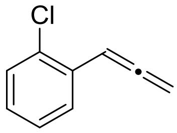

The title compound was prepared following the general procedure **GP1**. Purification by flash column chromatography (Pentane) gave the title compound as a colourless oil (648 mg, 72% yield). The spectroscopic data for this product matched the literature data[5]. ^{1}H NMR (400 MHz, $CDCl_3$, 25 ℃) δ 7.55–7.40 (m, 2H), 7.27 (dd, *J* = 10.8, 4.2 Hz,1H), 7.20–7.16 (m, 1H), 6.68 (t, *J* = 6.8 Hz, 1H), 5.25 (d, *J* = 6.9 Hz, 2H).

4-(propa-1,2-dien-1-yl)-1,1'-biphenyl (4p)

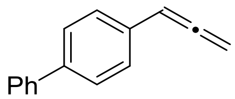

The title compound was prepared following the general procedure **GP1**. Purification by flash column chromatography (Pentane:EA=100:1) gave the title compound as a white solide (921 mg, 80% yield). The spectroscopic data for this product matched the literature data[5]. ^{1}H NMR (400 MHz, $CDCl_3$, 25 ℃) δ 7.63–7.53 (m, 4H), 7.44 (t, *J* = 7.5 Hz, 2H), 7.40–7.29 (m, 3H), 6.21 (t, *J* = 6.8 Hz, 1H), 5.18 (d, *J* = 6.8 Hz, 2H).

1.3 General Procedure for the Cu-catalyzed Hydrosilylation of 1,1-disubsitituted Allenes

In an Ar-filled dry box, $Cu(OAc)_2$ (1.8 mg, 0.01 mmol), Xantphos (8.67 mg, 0.015 mmol) and THF (0.4 mL) were added to a 4-mL screw-capped vial and stirred for 15 mins. Then 1,1-disubsitituted allenes (0.2 mmol) and Ph_2SiH_2 (1.2 equiv., 0.24 mmol) were added. The vial was sealed with a cap containing a PTFE septum. The reaction mixture was stirred at room temperature for 12 h and the resulting solution was concentrated in vacuum. The crude product was purified by column chromatography on silica gel with a mixture of ethyl acetate and hexane as eluent. The conditions for flash chromatography and data for characterization of the products are listed below.

(*E*)-Diphenyl(3-phenylbut-2-en-1-yl)silane (3a)

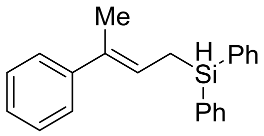

In an Ar-filled dry box, $Cu(OAc)_2$ (1.8 mg, 0.01 mmol), Xantphos (8.67 mg, 0.015 mmol) and THF (0.4 mL) were added to a 4-mL screw-capped vial and stirred for 15 mins. Then **1a** (0.2 mmol, 26 mg) and Ph_2SiH_2 (0.24 mmol, 45 mg) were added. The vial was sealed with a cap containing a PTFE septum. The reaction mixture was stirred at room temperature for 12 h and the resulting solution

was concentrated in vacuum. The title compound was isolated (53 mg, 85% yield) as a colorless oil after chromatography on silica with PE/EA (100:1). Rf = 0.50. The spectroscopic data for this product matched the literature data[7]. ^{1}H NMR (400 MHz, $CDCl_3$, 25 ºC) δ 7.64–7.53 (m, 4H), 7.37 (dd, J = 12.1, 7.2 Hz, 6H), 7.26 (d, J = 4.4 Hz, 4H), 7.21–7.11 (m, 1H), 5.89 (t, J = 7.7 Hz, 1H), 4.92 (t, J = 3.5 Hz, 1H), 2.25 (dd, J = 8.6, 3.6 Hz, 2H), 1.83 (s, 3H).

(E)-Diphenyl(3-(p-tolyl)but-2-en-1-yl)silane (3b)

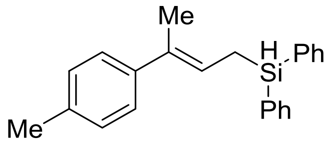

In an Ar-filled dry box, $Cu(OAc)_2$ (1.8 mg, 0.01 mmol), Xantphos (8.67 mg, 0.015 mmol) and THF (0.4 mL) were added to a 4-mL screw-capped vial and stirred for 15 mins. Then **1b** (0.2 mmol, 29 mg) and Ph_2SiH_2 (0.24 mmol, 45 mg) were added. The vial was sealed with a cap containing a PTFE septum. The reaction mixture was stirred at room temperature for 12 h and the resulting solution was concentrated in vacuum. The title compound was isolated (49 mg, 75% yield) as a colorless oil after chromatography on silica with PE/EA (100:1). Rf = 0.50. The spectroscopic data for this product matched the literature data[7]. ^{1}H NMR (600 MHz, $CDCl_3$, 25 °C) δ 7.65–7.57 (m, 4H), 7.45–7.40 (m, 2H), 7.38 (m, 4H), 7.19 (m, 2H), 7.10 (d, J = 7.9 Hz, 2H), 5.88 (t, J = 8.5 Hz, 1H), 4.93 (t, J = 3.5 Hz, 1H), 2.33 (s, 3H), 2.27 (dd, J = 8.5, 3.6 Hz, 2H), 1.83 (s, 3H).

(*E*)-Diphenyl(3-(m-tolyl)but-2-en-1-yl)silane (3c)

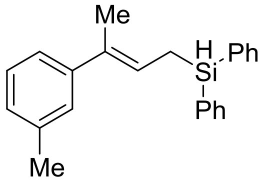

In an Ar-filled dry box, $Cu(OAc)_2$ (1.8 mg, 0.01 mmol), Xantphos (8.67 mg, 0.015 mmol) and THF (0.4 mL) were added to a 4-mL screw-capped vial and stirred for 15 mins. Then **1c** (0.2 mmol, 29 mg) and Ph_2SiH_2 (0.24 mmol, 45 mg) were added. The vial was sealed with a cap containing a PTFE septum. The reaction mixture was stirred at room temperature for 12 h and the resulting solution was concentrated in vacuum. The title compound was isolated (53 mg, 81% yield) as a colorless oil after chromatography on silica with PE/EA (100:1). Rf = 0.50. ^{1}H NMR (400 MHz, $CDCl_3$, 25 ºC) δ 7.61 (d, J = 6.0 Hz, 4H), 7.53–7.32 (m, 6H), 7.22–6.98 (m, 4H), 5.89 (t, J = 9.3 Hz, 1H), 4.93 (t, J = 3.6 Hz, 1H), 2.34 (s, 3H), 2.27 (dd, J = 8.6, 3.6 Hz, 2H), 1.84 (s, 3H). ^{13}C NMR (151 MHz, $CDCl_3$, 25 ºC) δ 144.3, 137.7, 135.3, 134.3, 133.9, 129.9, 128.2, 128.1, 127.1, 126.5, 122.8, 122.8, 21.7, 15.9, 15.9. IR (ATR): ν 3049, 2922, 1956, 1886, 1774, 1684, 1487, 1427, 1375, 1330, 1274, 1192, 1058, 864, 805, 734 cm^{-1}. HRMS (ESI+): (m/z) calcd for $C_{23}H_{25}Si^+$ ($M+H^+$), 329.1720; found, 329.1719.

(*E*)-(3-(4-Ethylphenyl)but-2-en-1-yl)diphenylsilane (3d)

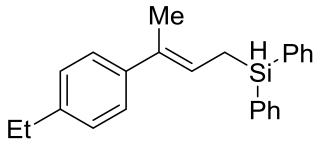

In an Ar-filled dry box, $Cu(OAc)_2$ (1.8 mg, 0.01 mmol), Xantphos (8.67 mg, 0.015 mmol) and THF (0.4 mL) were added to a 4-mL screw-capped vial and stirred for 15 mins. Then **1d** (0.2 mmol, 32 mg) and Ph_2SiH_2 (0.24 mmol, 45 mg) were added. The vial was sealed with a cap containing a PTFE septum. The reaction mixture was stirred at room temperature for 12 h and the resulting solution was concentrated in vacuum.The title compound was isolated (52 mg, 76% yield) as a colorless oil after chromatography on silica with PE/EA (100:1). Rf = 0.50. ^{1}H NMR (400 MHz, $CDCl_3$, 25 ºC) δ 7.61 (d, *J* = 6.1 Hz, 4H), 7.49–7.34 (m, 6H), 7.25–7.08 (m, 4H), 5.98–5.74 (m, 1H), 4.93 (t, *J* = 3.5 Hz, 1H), 2.64 (q, *J* = 7.6 Hz, 2H), 2.27 (dd, *J* = 8.6, 3.6 Hz, 2H), 1.84 (s, 3H), 1.24 (t, *J* = 7.8 Hz, 3H). ^{13}C NMR (151 MHz, $CDCl_3$, 25 ºC) δ 142.4, 141.6, 135.3, 134.0, 134.0, 129.8, 128.1, 127.7, 125.6, 122.1, 28.6, 15.9, 15.9, 15.8. IR (ATR): ν 3134, 2967, 2870, 2124, 1900, 1818, 1766, 1681, 1587, 1375, 1267, 1062, 969, 827, 734 cm^{-1}. HRMS (APCI+): (m/z) calcd for $C_{24}H_{27}Si^+$ ($M+H^+$), 343.1877; found, 343.1874.

(*E*)-(3-(4-(Tert-butyl)phenyl)but-2-en-1-yl)diphenylsilane (3e)

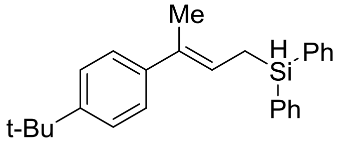

In an Ar-filled dry box, $Cu(OAc)_2$ (1.8 mg, 0.01 mmol), Xantphos (8.67 mg, 0.015 mmol) and THF (0.4 mL) were added to a 4-mL screw-capped vial and stirred for 15 mins. Then **1e** (0.2 mmol, 37 mg) and Ph_2SiH_2 (0.24 mmol, 45 mg) were added. The vial was sealed with a cap containing a PTFE septum. The reaction mixture was stirred at room temperature for 12 h and the resulting solution was concentrated in vacuum.The title compound was isolated (53 mg, 72% yield) as a colorless oil after chromatography on silica with PE/EA (100:1). Rf = 0.50. The spectroscopic data for this product matched the literature data[7]. ^{1}H NMR (600 MHz, $CDCl_3$, 25 ºC) δ 7.63–7.57 (m, 4H), 7.44–7.40 (m, 2H), 7.40–7.35 (m, 4H), 7.34–7.28 (m, 2H), 7.25–7.20 (m, 2H), 5.90 (t, *J* = 8.5 Hz, 1H), 4.92 (t, *J* = 3.5 Hz, 1H), 2.27 (dd, *J* = 8.8, 4.0 Hz, 2H), 1.84 (s, 3H), 1.32 (s, 9H).

(*E*)-(3-(4-Fluorophenyl)but-2-en-1-yl)diphenylsilane (3f)

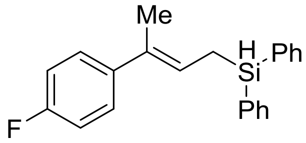

In an Ar-filled dry box, $Cu(OAc)_2$ (1.8 mg, 0.01 mmol), Xantphos (8.67 mg, 0.015 mmol) and THF (0.4 mL) were added to a 4-mL screw-capped vial and stirred for 15 mins. Then **1f** (0.2 mmol, 30 mg) and Ph_2SiH_2 (0.24 mmol, 45 mg) were added. The vial was sealed with a cap containing a PTFE septum. The reaction mixture was stirred at room temperature for 12 h and the resulting solution was concentrated in vacuum. The title compound was isolated (49 mg, 74% yield) as a colorless oil after chromatography on silica with PE/EA (100:1). Rf = 0.45. The spectroscopic data for this product matched the literature data[7]. ^{1}H NMR (600 MHz, $CDCl_3$, 25 ºC) δ 7.60 (d, *J* = 9.5 Hz, 4H),

7.47–7.35 (m, 6H), 7.25–7.16 (m, 2H), 7.01–6.91 (m, 2H), 5.83 (t, J = 7.7 Hz, 1H), 4.93 (t, J = 3.5 Hz, 1H), 2.26 (dd, J = 8.5, 3.6 Hz, 2H), 1.82 (s, 3H).

(*E*)-(3-(2-Fluorophenyl)but-2-en-1-yl)diphenylsilane (3g)

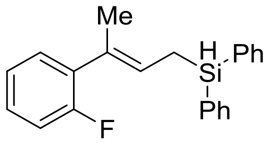

In an Ar-filled dry box, $Cu(OAc)_2$ (1.8 mg, 0.01 mmol), Xantphos (8.67 mg, 0.015 mmol) and THF (0.4 mL) were added to a 4-mL screw-capped vial and stirred for 15 mins. Then **1g** (0.2 mmol, 30 mg) and Ph_2SiH_2 (0.24 mmol, 45 mg) were added. The vial was sealed with a cap containing a PTFE septum. The reaction mixture was stirred at room temperature for 12 h and the resulting solution was concentrated in vacuum. The title compound was isolated (53 mg, 80% yield) as a colorless oil after chromatography on silica with PE/EA (100:1). Rf = 0.45. ^{1}H NMR (400 MHz, $CDCl_3$, 25 ºC) δ 7.63 (d, J = 7.7 Hz, 4H), 7.49–7.33 (m, 6H), 7.24–6.91 (m, 4H), 5.74 (t, 1H), 4.95 (t, J = 3.5 Hz, 1H), 2.27 (dd, J = 8.6, 3.5 Hz, 2H), 1.84 (s, 3H). ^{13}C NMR (151 MHz, $CDCl_3$, 25 ºC) δ 159.9 (d, J = 246.5 Hz), 135.3, 133.9, 132.9 (d, J = 14.2 Hz), 130.7, 129.9, 129.8 (d, J = 4.8 Hz), 128.2, 127.9 (d, J = 8.2 Hz), 126.2 (d, J = 2.2 Hz), 123.9 (d, J = 3.4 Hz), 115.7 (d, J = 22.8 Hz), 16.9, 16.9, 15.7. ^{19}F NMR (565 MHz, $CDCl_3$, 25 ºC) δ –115.1 – –115.4 (m, 1F). IR (ATR): ν 3067, 2922, 2124, 1953, 1882, 1766, 1695, 1487, 1330, 1252, 1151, 1058, 972, 849, 756cm^{-1}. HRMS (APCI+): (m/z) calcd for $C_{22}H_{22}FSi^{+}$ (M+H^{+}), 333.1469; found, 333.1472.

(*E*)-(3-(3-Fluorophenyl)but-2-en-1-yl)diphenylsilane (3h)

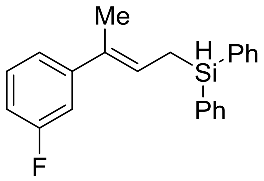

In an Ar-filled dry box, $Cu(OAc)_2$ (1.8 mg, 0.01 mmol), Xantphos (8.67 mg, 0.015 mmol) and THF (0.4 mL) were added to a 4-mL screw-capped vial and stirred for 15 mins. Then **1h** (0.2 mmol, 30 mg) and Ph_2SiH_2 (0.24 mmol, 45 mg) were added. The vial was sealed with a cap containing a PTFE septum. The reaction mixture was stirred at room temperature for 12 h and the resulting solution was concentrated in vacuum. The title compound was isolated (54 mg, 81% yield) as a colorless oil after chromatography on silica with PE/EA (100:1). Rf = 0.45. ^{1}H NMR (600 MHz, $CDCl_3$, 25 ºC) δ 7.65–7.56 (m, 4H), 7.49–7.35 (m, 6H), 7.30–7.18 (m, 1H), 7.07–6.79 (m, 3H), 5.94 (t, J = 7.9 Hz, 1H), 4.93 (t, J = 3.5 Hz, 1H), 2.28 (dd, J = 8.6, 3.6 Hz, 2H), 1.82 (s, 3H). ^{13}C NMR (151 MHz, $CDCl_3$, 25 ºC) δ 162.9 (d, J = 244.6 Hz), 146.5 (d, J = 7.2 Hz), 135.3, 133.7, 133.1 (d, J = 2.5 Hz), 129.9, 129.5 (d, J = 8.6 Hz), 128.2, 124.2, 121.1 (d, J = 2.5 Hz), 113.0 (d, J = 21.2 Hz), 112.4 (d, J = 21.5 Hz), 16.1, 15.7. IR (ATR): ν 3071, 2967, 2128, 1956, 1886, 1766, 1610, 1584, 1487, 1330, 1267, 1192, 1066, 954, 872, 734, 700 cm^{-1}. HRMS (ESI^{+}): (m/z) calcd for $C_{22}H_{22}FSi^{+}$ (M+H^{+}), 333.1469; found, 333.1465.

(*E*)-(3-(3-Chlorophenyl)but-2-en-1-yl)diphenylsilane (3i)

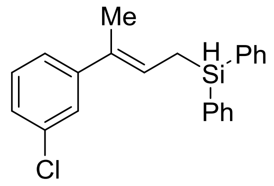

In an Ar-filled dry box, $Cu(OAc)_2$ (1.8 mg, 0.01 mmol), Xantphos (8.67 mg, 0.015 mmol) and THF (0.4 mL) were added to a 4-mL screw-capped vial and stirred for 15 mins. Then **1i** (0.2 mmol, 33 mg) and Ph_2SiH_2 (0.24 mmol, 45 mg) were added. The vial was sealed with a cap containing a PTFE septum. The reaction mixture was stirred at room temperature for 12 h and the resulting solution was concentrated in vacuum. The title compound was isolated (57 mg, 82% yield) as a colorless oil after chromatography on silica with PE/EA (100:1). Rf = 0.45. ^{1}H NMR (600 MHz, $CDCl_3$, 25 ºC) δ 7.71–7.58 (m, 4H), 7.53–7.44 (m, 2H), 7.44–7.37 (m, 4H), 7.31–7.24 (m, 1H), 7.25–7.12 (m, 3H), 5.95 (t, *J* = 7.9 Hz, 1H), 4.97 (t, *J* = 3.5 Hz, 1H), 2.30 (dd, *J* = 8.2, 4.0 Hz, 2H), 1.84 (s, 3H). ^{13}C NMR (151 MHz, $CDCl_3$, 25 ºC) δ 146.0, 135.3, 134.1, 133.7, 133.0, 129.9, 129.4, 128.2, 126.3, 125.8, 124.4, 123.7, 16.2, 15.7. IR (ATR): ν 3134, 2918, 2124, 1956, 1886, 1766, 1688, 1591, 1476, 1375, 1304, 1155, 1058, 849, 730, 693 cm^{-1}. HRMS (ESI+): (m/z) calcd for $C_{22}H_{22}ClSi^+$ (M+H$^+$), 349.1174; found, 349.1174.

(*E*)-(3-(4-Chlorophenyl)but-2-en-1-yl)diphenylsilane (3j)

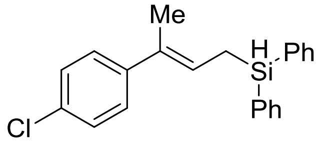

In an Ar-filled dry box, $Cu(OAc)_2$ (1.8 mg, 0.01 mmol), Xantphos (8.67 mg, 0.015 mmol) and THF (0.4 mL) were added to a 4-mL screw-capped vial and stirred for 15 mins. Then **1j** (0.2 mmol, 33 mg) and Ph_2SiH_2 (0.24 mmol, 45 mg) were added. The vial was sealed with a cap containing a PTFE septum. The reaction mixture was stirred at room temperature for 12 h and the resulting solution was concentrated in vacuum. The title compound was isolated (61 mg, 88% yield) as a colorless oil after chromatography on silica with PE/EA (100:1). Rf = 0.45. ^{1}H NMR (600 MHz, $CDCl_3$, 25 ºC) δ 7.62–7.56 (m, 4H), 7.47–7.32 (m, 6H), 7.25–7.21 (m, 2H), 7.20–7.16 (m, 2H), 5.88 (t, *J* = 7.1 Hz, 1H), 4.92 (t, *J* = 3.5 Hz, 1H), 2.27 (dd, *J* = 8.5, 3.6 Hz, 2H), 1.81 (s, 3H). ^{13}C NMR (151 MHz, $CDCl_3$, 25 ºC) δ 142.6, 135.3, 133.8, 133.1, 132.0, 129.9, 128.3, 128.2, 126.8, 123.6, 16.1, 15.7. IR (ATR): ν 3131, 2926, 1956, 1818, 1766, 1684, 1587, 1490, 1330, 1259, 1114, 928, 805, 734, 700 cm^{-1}. HRMS (APCI+): (m/z) calcd for $C_{22}H_{21}ClSi$ (M+H$^+$), 349.1174; found, 349.1166.

(*E*)-(3-(3-Bromophenyl)but-2-en-1-yl)diphenylsilane (3k)

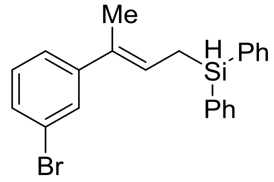

In an Ar-filled dry box, $Cu(OAc)_2$ (1.8 mg, 0.01 mmol), Xantphos (8.67 mg, 0.015 mmol) and THF (0.4 mL) were added to a 4-mL screw-capped vial and stirred for 15 mins. Then **1k** (0.2 mmol, 42 mg) and Ph_2SiH_2 (0.24 mmol, 45 mg) were added. The vial was sealed with a cap containing a PTFE

septum. The reaction mixture was stirred at room temperature for 12 h and the resulting solution was concentrated in vacuum. The title compound was isolated (51 mg, 65% yield) as a colorless oil after chromatography on silica with PE/EA (100:1). Rf = 0.45. ^{1}H NMR (600 MHz, $CDCl_3$, 25 ºC) δ 7.64–7.54 (m, 4H), 7.48–7.36 (m, 7H), 7.31 (d, J = 6.2 Hz, 1H), 7.20–7.09 (m, 2H), 5.90 (t, J = 8.6 Hz, 1H), 4.92 (t, J = 3.5 Hz, 1H), 2.27 (dd, J = 8.6, 3.6 Hz, 2H), 1.80 (s, 3H). ^{13}C NMR (151 MHz, $CDCl_3$, 25 ºC) δ 146.4, 135.3, 133.7, 132.9, 129.9, 129.7, 129.2, 128.7, 128.2, 124.5, 124.2, 122.5, 16.2, 15.7. IR (ATR): ν 3138, 2922, 1960, 1818, 1766, 1669, 1587, 1427, 1375, 1267, 1185, 1085, 812, 734 cm^{-1}. HRMS (APCI+): (m/z) calcd for $C_{22}H_{22}BrSi^+$ (M+H$^+$), 393.0669; found, 393.0674.

(*E*)-Diphenyl(3-(3-(trifluoromethyl)phenyl)but-2-en-1-yl)silane (3l)

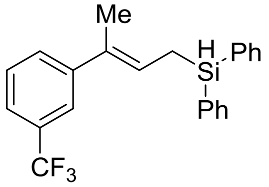

In an Ar-filled dry box, $Cu(OAc)_2$ (1.8 mg, 0.01 mmol), Xantphos (8.67 mg, 0.015 mmol) and THF (0.4 mL) were added to a 4-mL screw-capped vial and stirred for 15 mins. Then **1l** (0.2 mmol, 40 mg) and Ph_2SiH_2 (0.24 mmol, 45 mg) were added. The vial was sealed with a cap containing a PTFE septum. The reaction mixture was stirred at room temperature for 12 h and the resulting solution was concentrated in vacuum. The title compound was isolated (65 mg, 85% yield) as a colorless oil after chromatography on silica with PE/EA (100:1). Rf = 0.50. ^{1}H NMR (600 MHz, $CDCl_3$, 25 ºC) δ 7.63–7.59 (m, 4H), 7.48 (s, 1H), 7.47–7.35 (m, 10H), 5.94 (t, J = 8.5 Hz, 1H), 4.94 (t, J = 3.6 Hz, 1H), 2.30 (dd, J = 8.6, 3.6 Hz, 2H), 1.85 (s, 3H). ^{13}C NMR (151 MHz, $CDCl_3$, 25 ºC) δ 144.9, 135.3, 133.7, 133.0, 130.5 (q, J = 31.9 Hz), 130.0, 128.8, 128.7, 128.2, 124.9, 124.4 (q, J = 272.2 Hz), δ 123.0 (q, J = 3.8 Hz), 122.3 (q, J = 3.8 Hz), 16.2, 15.7. IR (ATR): ν 3071, 2937, 2124, 1956, 1886, 1770, 1692, 1487, 1379, 1259, 1162, 1077, 902, 853, 730, 700 cm^{-1}. HRMS (APCI+): (m/z) calcd for $C_{23}H_{22}F_3Si^+$ (M+H$^+$), 383.1437; found, 383.1440.

(*E*)-Diphenyl(3-(2-(trifluoromethyl)phenyl)but-2-en-1-yl)silane (3m)

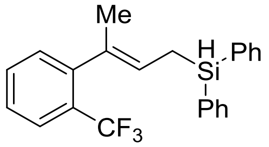

In an Ar-filled dry box, $Cu(OAc)_2$ (1.8 mg, 0.01 mmol), Xantphos (8.67 mg, 0.015 mmol) and THF (0.4 mL) were added to a 4-mL screw-capped vial and stirred for 15 mins. Then **1m** (0.2 mmol, 40 mg) and Ph_2SiH_2 (0.24 mmol, 45 mg) were added. The vial was sealed with a cap containing a PTFE septum. The reaction mixture was stirred at room temperature for 12 h and the resulting solution was concentrated in vacuum. The title compound was isolated (53 mg, 70% yield) as a colorless oil after chromatography on silica with PE/EA (100:1). Rf = 0.50. ^{1}H NMR (600 MHz, $CDCl_3$, 25 ºC) δ 7.67–7.56 (m, 5H), 7.47–7.37 (m, 7H), 7.30 (t, J = 7.7 Hz, 1H), 7.07 (d, J = 7.6 Hz, 1H), 5.44 (t, J = 8.5 Hz, 1H), 4.95 (t, J = 3.6 Hz, 1H), 2.25 (dd, J = 8.4, 3.7 Hz, 2H), 1.83 (s, 3H). ^{13}C NMR (151 MHz, $CDCl_3$, 25 ºC) δ 145.2, 135.3, 134.0, 133.1, 131.4, 130.8, 129.9, 128.1, 127.9 (q, J = 29.6 Hz), 126.5, 125.9 (q, J = 5.3 Hz), 125.1, 124.5 (q, J = 273.8 Hz).18.8, 15.3. ^{19}F NMR (565 MHz,

$CDCl_3$, 25 ºC) δ –58.3 (3F). IR (ATR): ν 3134, 2918, 1956, 1886, 1763, 1677, 1602, 1427, 1312, 1259, 1166, 1069, 805, 764, 730, 697 cm^{-1}. HRMS (APCI+): (m/z) calcd for $C_{23}H_{22}F_3Si^+$ ($M+H^+$), 383.1437; found, 383.14.

(*E*)-Diphenyl(3-(thiophen-2-yl)but-2-en-1-yl)silane (3n)

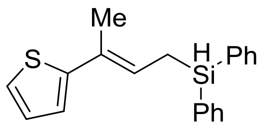

In an Ar-filled dry box, $Cu(OAc)_2$ (1.8 mg, 0.01 mmol), Xantphos (8.67 mg, 0.015 mmol) and THF (0.4 mL) were added to a 4-mL screw-capped vial and stirred for 15 mins. Then **1n** (0.2 mmol, 27 mg) and Ph_2SiH_2 (0.24 mmol, 45 mg) were added. The vial was sealed with a cap containing a PTFE septum. The reaction mixture was stirred at room temperature for 12 h and the resulting solution was concentrated in vacuum. The title compound was isolated (32 mg, 50% yield) as a colorless oil after chromatography on silica with PE/EA (100:1). Rf = 0.50. ^1H NMR (600 MHz, $CDCl_3$, 25 ºC) δ 7.63–7.55 (m, 4H), 7.46–7.40 (m, 2H), 7.40–7.33 (m, 4H), 7.23–7.20 (m, 1H), 7.18–7.12 (m, 1H), 7.01–6.97 (m, 1H), 6.02 (t, *J* = 9.3 Hz, 1H), 4.91 (t, *J* = 3.5 Hz, 1H), 2.25 (dd, *J* = 8.6, 3.6 Hz, 2H), 1.85 (s, 3H). ^{13}C NMR (151 MHz, $CDCl_3$, 25 ºC) δ 145.3, 135.3, 133.9, 129.9, 129.2, 128.1, 125.2, 125.2, 121.9, 15.6, 15.4. IR (ATR): ν 3067, 2926, 1971,1866, 1710, 1677, 1587, 1427, 1375, 1263, 1118, 1066, 969, 816, 738, 700 cm^{-1}. HRMS (APCI+): (m/z) calcd for $C_{20}H_{21}SSi^+$ ($M+H^+$), 321.1128; found, 321.1124.

(*E*)-(3-(Naphthalen-2-yl)but-2-en-1-yl)diphenylsilane (3o)

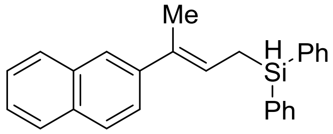

In an Ar-filled dry box, $Cu(OAc)_2$ (1.8 mg, 0.01 mmol), Xantphos (8.67 mg, 0.015 mmol) and THF (0.4 mL) were added to a 4-mL screw-capped vial and stirred for 15 mins. Then **1o** (0.2 mmol, 36 mg) and Ph_2SiH_2 (0.24 mmol, 45 mg) were added. The vial was sealed with a cap containing a PTFE septum. The reaction mixture was stirred at room temperature for 12 h and the resulting solution was concentrated in vacuum. The title compound was isolated (54 mg, 74% yield) as a colorless oil after chromatography on silica with PE/EA (100:1). Rf = 0.40. The spectroscopic data for this product matched the literature data[7]. ^1H NMR (600 MHz, $CDCl_3$, 25 ºC) δ 7.84–7.73 (m, 3H), 7.71–7.62 (m, 5H), 7.54–7.36 (m, 9H), 6.10 (t, *J* = 9.2 Hz, 1H), 4.99 (t, *J* = 3.5 Hz, 1H), 2.36 (dd, *J* = 8.6, 3.6 Hz, 2H), 1.98 (s, 3H).

(*E*)-(3-([1,1'-Biphenyl]-4-yl)but-2-en-1-yl)diphenylsilane (3p)

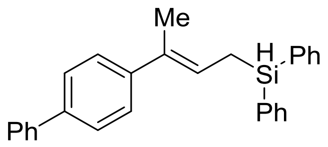

In an Ar-filled dry box, $Cu(OAc)_2$ (1.8 mg, 0.01 mmol), Xantphos (8.67 mg, 0.015 mmol) and THF (0.4 mL) were added to a 4-mL screw-capped vial and stirred for 15 mins. Then **1p** (0.2 mmol, 41 mg) and Ph_2SiH_2 (0.24 mmol, 45 mg) were added. The vial was sealed with a cap containing a PTFE septum. The reaction mixture was stirred at room temperature for 12 h and the resulting solution was concentrated in vacuum. The title compound was isolated (64 mg, 83% yield) as a colorless oil after chromatography on silica with PE/EA (100:1). Rf = 0.40. The spectroscopic data for this product matched the literature data[7]. ^{1}H NMR (600 MHz, $CDCl_3$, 25 ºC) δ 7.67–7.58 (m, 6H), 7.54 (d, J = 8.3 Hz, 2H), 7.48–7.30 (m, 11H), 6.00 (t, J = 7.8 Hz, 1H), 4.96 (t, J = 3.5 Hz, 1H), 2.32 (dd, J = 8.6, 3.6 Hz, 2H), 1.89 (s, 3H).

(*E*)-(3-(3-Methoxyphenyl)but-2-en-1-yl)diphenylsilane (3q)

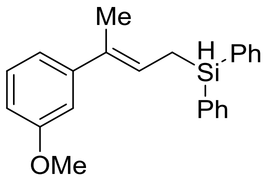

In an Ar-filled dry box, $Cu(OAc)_2$ (1.8 mg, 0.01 mmol), Xantphos (8.67 mg, 0.015 mmol) and THF (0.4 mL) were added to a 4-mL screw-capped vial and stirred for 15 mins. Then **1q** (0.2 mmol, 32 mg) and Ph_2SiH_2 (0.24 mmol, 45 mg) were added. The vial was sealed with a cap containing a PTFE septum. The reaction mixture was stirred at room temperature for 12 h and the resulting solution was concentrated in vacuum. The title compound was isolated (62 mg, 90% yield) as a colorless oil after chromatography on silica with PE/EA=50:1. Rf = 0.40. ^{1}H NMR (600 MHz, $CDCl_3$, 25 ºC) δ 7.67–7.53 (m, 4), 7.46–7.33 (m, 6), 7.24–7.15 (m, 1H), 6.93–6.71 (m, 3), 5.92 (t, J = 8.5 Hz, 1H), 4.93 (t, J = 3.5 Hz, 1H), 3.80 (s, 3H), 2.27 (dd, J = 8.1, 3.2 Hz, 2), 1.83 (s, 3). ^{13}C NMR (151 MHz, $CDCl_3$, 25 ºC) δ 159.5, 145.9 135.3, 134.1 133.9, 129.9 129.1, 128.1, 123.3 118.2, 111.6 111.6 55.3 15.9, 15.9. IR (ATR): ν 3067, 2937, 1960, 1866, 1766, 1636, 1483, 1289, 1114, 1043, 995, 864, 779, 700 cm^{-1}. HRMS (ESI$^+$): (m/z) calcd for $C_{23}H_{25}OSi^+$ (M+H$^+$), 345.1669; found, 345.1669.

(*E*)-(3-(Benzo[d][1,3]dioxol-5-yl)but-2-en-1-yl)diphenylsilane (3r)

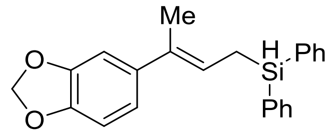

In an Ar-filled dry box, $Cu(OAc)_2$ (1.8 mg, 0.01 mmol), Xantphos (8.67 mg, 0.015 mmol) and THF (0.4 mL) were added to a 4-mL screw-capped vial and stirred for 15 mins. Then **1r** (0.2 mmol, 35 mg) and Ph_2SiH_2 (0.24 mmol, 45 mg) were added. The vial was sealed with a cap containing a PTFE septum. The reaction mixture was stirred at room temperature for 12 h and the resulting solution was concentrated in vacuum. The title compound was isolated (61 mg, 85% yield) as a colorless oil after chromatography on silica with PE/EA=50:1. Rf = 0.40. The spectroscopic data for this product matched the literature data[7]. ^{1}H NMR (600 MHz, $CDCl_3$, 25 ºC) δ 7.63–7.57 (m, 4H), 7.45–7.36 (m, 6H), 6.78 (d, J = 0.9 Hz, 1H), 6.76–6.71 (m, 2H), 5.93 (s, 2H), 5.79 (t, J = 8.5 Hz, 1H), 4.92 (t, J = 3.5 Hz, 1H), 2.25 (dd, J = 7.7, 3.5 Hz, 2H), 1.80 (s, 3H).

Ethyl (*E*)-4-(4-(diphenylsilyl)but-2-en-2-yl)benzoate (3s)

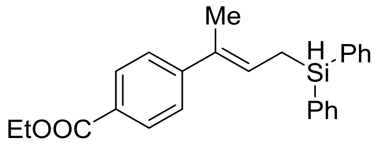

In an Ar-filled dry box, $Cu(OAc)_2$ (1.8 mg, 0.01 mmol), Xantphos (8.67 mg, 0.015 mmol) and THF (0.4 mL) were added to a 4-mL screw-capped vial and stirred for 15 mins. Then **1s** (0.2 mmol, 40 mg) and Ph_2SiH_2 (0.24 mmol, 45 mg) were added. The vial was sealed with a cap containing a PTFE septum. The reaction mixture was stirred at room temperature for 12 h and the resulting solution was concentrated in vacuum. The title compound was isolated (66 mg, 85% yield) as a colorless oil after chromatography on silica with PE/EA=50:1. Rf = 0.40.^{1}H NMR (600 MHz, $CDCl_3$, 25 ºC) δ 8.00–7.91 (m, 2H), 7.68–7.57 (m, 4H), 7.49 –7.36 (m, 6H), 7.36–7.28 (m, 2H), 6.04 (t, J = 8.6 Hz, 1H), 4.96 (t, J = 3.5 Hz, 1H), 4.38 (q, J = 7.2 Hz, 2H), 2.32 (dd, J = 8.1, 3.2 Hz, 2H), 1.86 (s, 3H), 1.41 (t, J = 7.1 Hz, 3H). ^{13}C NMR (101 MHz, $CDCl_3$, 25 ºC) δ 166.7, 148.5, 135.3, 134.5, 133.7, 129.9, 129.6, 128.2, 127.9, 125.5, 125.3, 60.9, 16.4, 15.6, 14.5. IR (ATR): ν 3049, 2981, 2128, 1960, 1886, 1714, 1602, 1505, 1427, 1367, 1274, 1185, 1058, 928, 853, 771 cm^{-1}. HRMS (ESI$^+$): (m/z) calcd for $C_{25}H_{27}O_2Si^+$ (M+H$^+$), 387.1775; found, 387.1775.

(*E*)-Diphenyl(3-(4-(trifluoromethoxy)phenyl)but-2-en-1-yl)silane (3t)

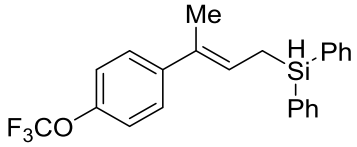

In an Ar-filled dry box, $Cu(OAc)_2$ (1.8 mg, 0.01 mmol), Xantphos (8.67 mg, 0.015 mmol) and THF (0.4 mL) were added to a 4-mL screw-capped vial and stirred for 15 mins. Then **1t** (0.2 mmol, 43 mg) and Ph_2SiH_2 (0.24 mmol, 45 mg) were added. The vial was sealed with a cap containing a PTFE septum. The reaction mixture was stirred at room temperature for 12 h and the resulting solution was concentrated in vacuum. The title compound was isolated (63 mg, 79% yield) as a colorless oil after chromatography on silica with PE/EA=50:1. Rf = 0.40. ^{1}H NMR (400 MHz, $CDCl_3$, 25 ºC) δ 7.71–7.58 (m, 4H), 7.5 –7.38 (m, 6H), 7.35–7.25 (m, 2H), 7.21–7.07 (m, 2H), 5.93 (t, *J* = 8.5 Hz, 1H), 4.98 (t, *J* = 3.5 Hz, 1H), 2.32 (dd, *J* = 8.6, 3.5 Hz, 2H), 1.87 (s, 3H). ^{13}C NMR (151 MHz, $CDCl_3$, 25 ºC) δ 147.8, 142.9, 135.3, 133.8, 133.0, 129.9, 128.2, 126.8, 124.0, 120.8, 120.7 (q, *J* = 256.7 Hz), 16.1, 15.8. ^{19}F NMR (565 MHz, $CDCl_3$, 25 ºC) δ –57.9 (s, 3F). IR (ATR): ν 3052, 2933, 1960, 1818, 1770, 1692, 1591, 1427, 1252, 1162, 1066, 924, 805, 700 cm^{-1}. HRMS (ESI$^+$): (m/z) calcd for $C_{23}H_{22}F_3OSi^+$ (M+H$^+$), 399.1387; found, 399.1387.

(*E*)-(3-(4-(Methylthio)phenyl)but-2-en-1-yl)diphenylsilane (3u)

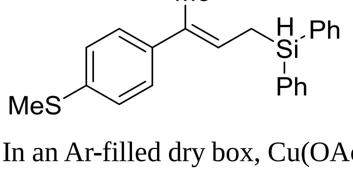

In an Ar-filled dry box, $Cu(OAc)_2$ (1.8 mg, 0.01 mmol), Xantphos (8.67 mg, 0.015 mmol) and THF (0.4 mL) were added to a 4-mL screw-capped vial and stirred for 15 mins. Then **1n** (0.2 mmol, 35 mg) and Ph_2SiH_2 (0.24 mmol, 45 mg) were added. The vial was sealed with a cap containing a PTFE septum. The reaction mixture was stirred at room temperature for 12 h and the resulting solution

was concentrated in vacuum. The title compound was isolated (60 mg, 83% yield) as a colorless oil after chromatography on silica with PE/EA=100:1. Rf = 0.40. ^{1}H NMR (600 MHz, $CDCl_3$, 25 ºC) δ 7.68–7.54 (m, 4H), 7.46–7.35 (m, 6H), 7.23–7.15 (m, 4H), 5.90 (t, J = 8.5 Hz, 1H), 4.93 (t, J = 3.5 Hz, 1H), 2.48 (s, 3H), 2.27 (dd, J = 7.7, 3.6 Hz, 2H), 1.82 (s, 3H). ^{13}C NMR (151 MHz, $CDCl_3$, 25 ºC) δ 141.2, 136.0, 135.3, 133.9, 133.5, 129.9, 128.1, 126.8, 126.0, 122.7, 16.3, 16.0, 15.7. IR (ATR): ν 3131, 2922, 1951, 1886, 1770, 1677, 1587, 1490, 1319, 1263, 1114, 969, 805, 700 cm^{-1}. HRMS (ESI^+): (m/z) calcd for $C_{23}H_{25}SSi^+$ ($M+H^+$), 361.1441; found, 361.1444.

(*E*)-Diphenyl(3-phenylhex-2-en-1-yl)silane (3v)

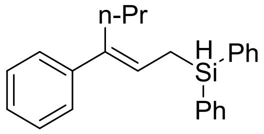

In an Ar-filled dry box, $Cu(OAc)_2$ (1.8 mg, 0.01 mmol), Xantphos (8.67 mg, 0.015 mmol) and THF (0.4 mL) were added to a 4-mL screw-capped vial and stirred for 15 mins. Then **1v** (0.2 mmol, 32 mg) and Ph_2SiH_2 (0.24 mmol, 45 mg) were added. The vial was sealed with a cap containing a PTFE septum. The reaction mixture was stirred at room temperature for 12 h and the resulting solution was concentrated in vacuum. The title compound was isolated (54mg, 81% yield) as a colorless oil after chromatography on silica with PE/EA=100:1. Rf = 0.50. ^{1}H NMR (600 MHz, $CDCl_3$, 25 ºC) δ 7.63–7.54 (m, 4H), 7.44–7.33 (m, 6H), 7.29–7.13 (m, 5H), 5.74 (t, J = 8.5 Hz, 1H), 4.91 (t, J = 3.6 Hz, 1H), 2.37–2.29 (m, 2H), 2.26 (dd, J = 8.5, 3.6 Hz, 2H), 1.32–1.13 (m, 2H), 0.80 (t, J = 7.3 Hz, 3H). ^{13}C NMR (151 MHz, $CDCl_3$, 25 ºC) δ 143.6, 139.8, 135.4, 133.9, 129.9, 128.2, 128.1, 126.4 126.3, 123.3 31.6, 21.6, 15.6 14. IR (ATR): ν 3067, 2959, 1956, 1818, 1766, 1673, 1595, 1490, 1379, 1267, 1155, 1066, 961, 805, 734, 700 cm^{-1}. HRMS (ESI^+): (m/z) calcd for $C_{24}H_{27}Si^+$ ($M+H^+$), 343.1877; found, 343.1871.

(*E*)-(3-Cyclohexylbut-2-en-1-yl)diphenylsilane (3w)

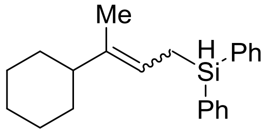

In an Ar-filled dry box, $Cu(OAc)_2$ (1.8 mg, 0.01 mmol), Xantphos (8.67 mg, 0.015 mmol) and THF (0.4 mL) were added to a 4-mL screw-capped vial and stirred for 15 mins. Then **1w** (0.2 mmol, 27 mg) and Ph_2SiH_2 (0.24 mmol, 45 mg) were added. The vial was sealed with a cap containing a PTFE septum. The reaction mixture was stirred at room temperature for 12 h and the resulting solution was concentrated in vacuum. The title compound was isolated (54mg, 81% yield, E:Z=89:11) as a colorless oil after chromatography on silica with PE/EA=100:1. Rf = 0.50. The spectroscopic data for this product matched the literature data[8]. ^{1}H NMR (400 MHz, $CDCl_3$, 25 ºC) δ 7.65–7.51 (m, 4H), 7.48–7.31 (m, 6H), 5.25 (E: tt, J = 8.3, 1.3 Hz, 0.89H), 5.17 (Z: td, J = 8.4, 1.6 Hz, 0.11H), 4.83 (t, J = 3.5 Hz, 1H), 2.11–1.96 (m, 2H), 1.88–1.55 (m, 6H), 1.40 (s, 3H), 1.31–1.02 (m, 5H).

(*E*)-Phenyl(3-phenylbut-2-en-1-yl)(p-tolyl)silane (3x)

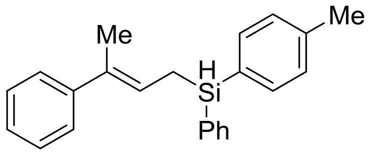

In an Ar-filled dry box, $Cu(OAc)_2$ (1.8 mg, 0.01 mmol), Xantphos (8.67 mg, 0.015 mmol) and THF (0.4 mL) were added to a 4-mL screw-capped vial and stirred for 15 mins. Then **1a** (0.2 mmol, 26 mg) and Phenyl(p-tolyl)silane (0.24 mmol, 48 mg) were added. The vial was sealed with a cap containing a PTFE septum. The reaction mixture was stirred at room temperature for 12 h and the resulting solution was concentrated in vacuum. The title compound was isolated (46 mg, 70% yield) as a colorless oil after chromatography on silica with PE/EA=100:1. ^{1}H NMR (600 MHz, $CDCl_3$, 25 ºC) δ 7.66–7.57 (m, 2H), 7.51 (d, *J* = 7.9 Hz, 2H), 7.46–7.40 (m, 1H), 7.40–7.35 (m, 2H), 7.29 (d, *J* = 3.8 Hz, 4H), 7.23–7.15 (m, 3H), 5.91 (t, *J* = 7.8 Hz, 1H), 4.92 (t, *J* = 3.5 Hz, 1H), 2.38 (s, 3H), 2.27 (dd, *J* = 8.6, 3.6 Hz, 2H), 1.86 (s, 3H). ^{13}C NMR (151 MHz, $CDCl_3$, 25 ºC) δ 144.3, 139.9, 135.4, 135.3, 134.3, 134.1, 130.2, 129.8, 128.9, 128.2, 128.1, 126.3, 125.6, 123.1, 21.7, 16.1, 15.9. IR (ATR): ν 3138, 2922, 1960, 1818, 1766, 1669, 1587, 1513, 1427, 1375, 1267, 1185, 1085, 812, 734 cm^{-1}. HRMS (APCI+): (m/z) calcd for $C_{23}H_{25}Si^{+}$ ($M+H^{+}$), 329.1720; found, 329.1719.

(*E*)-(3-Phenylbut-2-en-1-yl)di-p-tolylsilane (3y)

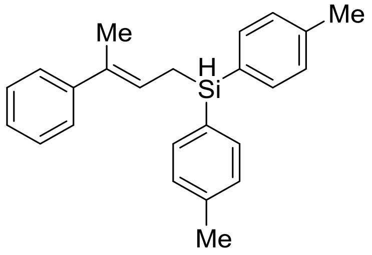

In an Ar-filled dry box, $Cu(OAc)_2$ (1.8 mg, 0.01 mmol), Xantphos (8.67 mg, 0.015 mmol) and THF (0.4 mL) were added to a 4-mL screw-capped vial and stirred for 15 mins. Then **1a** (0.2 mmol, 26 mg) and Di-p-tolylsilane (0.24 mmol, 51 mg) were added. The vial was sealed with a cap containing a PTFE septum. The reaction mixture was stirred at room temperature for 12 h and the resulting solution was concentrated in vacuum. The title compound was isolated (47 mg, 70% yield) as a colorless oil after chromatography on silica with PE/EA=100:1. ^{1}H NMR (600 MHz, $CDCl_3$, 25 ºC) δ 7.42–7.36 (m, 4H), 7.29–7.24 (m, 6H), 7.24–7.20 (m, 2H), 7.20–7.15 (m, 1H), 5.88 (t, *J* = 8.5 Hz, 1H), 4.86 (t, *J* = 3.5 Hz, 1H), 2.33 (s, 6H), 2.24 (dd, *J* = 8.6, 3.6 Hz, 2H), 1.85 (s, 3H). ^{13}C NMR (151 MHz, $CDCl_3$, 25 ºC) δ 144.3, 137.5, 135.9, 134.0, 133.9, 132.3, 130.6, 128.2, 128.0, 126.3, 125.6, 123.3, 21.6, 16.1, 15.9. IR (ATR): ν 3049, 2903, 2132, 1960, 1818, 1684, 1509, 1461, 1271, 1189, 1058, 1017, 902, 827, 734, 707 cm^{-1}. HRMS (APCI+): (m/z) calcd for $C_{24}H_{27}Si^{+}$ ($M+H^{+}$), 343.1877; found, 343.1863.

(*E*)-Bis(4-fluorophenyl)(3-phenylbut-2-en-1-yl)silane (3z)

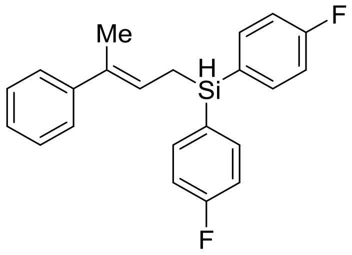

In an Ar-filled dry box, $Cu(OAc)_2$ (1.8 mg, 0.01 mmol), Xantphos (8.67 mg, 0.015 mmol) and THF (0.4 mL) were added to a 4-mL screw-capped vial and stirred for 15 mins. Then **1a** (0.2 mmol, 26 mg) and Bis(4-fluorophenyl)silane (0.24 mmol, 53 mg) were added. The vial was sealed with a cap containing a PTFE septum. The reaction mixture was stirred at room temperature for 12 h and the resulting solution was concentrated in vacuum. The title compound was isolated (57mg, 81% yield) as a colorless oil after chromatography on silica with PE/EA=100:1. ^{1}H NMR (600 MHz, $CDCl_3$, 25 ºC) δ 7.63–7.50 (m, 4H), 7.34–7.27 (m, 4H), 7.25–7.20 (m, 1H), 7.11 (dd, *J* = 10.1, 7.8 Hz, 4H), 5.89 (t, *J* = 8.5 Hz, 1H), 4.94 (t, *J* = 3.5 Hz, 1H), 2.27 (dd, *J* = 8.5, 3.5 Hz, 2H), 1.85 (s, 3H). ^{13}C NMR (151 MHz, $CDCl_3$, 25 ºC) δ 164.3 (d, *J* = 249.6 Hz), 144.0, 137.3 (d, *J* = 7.6 Hz), 134.6, 129.2 (d, *J* = 3.8 Hz), 128.3, 126.5, 125.6, 122.3, 115.5 (d, *J* = 19.8 Hz), 16.0, 15.9. ^{19}F NMR (565 MHz, $CDCl_3$, 25 ºC) δ –107.5 – –114.8 (m, 2F). IR (ATR): ν 3056, 2985, 1900, 1777, 1636, 1587, 1498, 1386, 1304, 1230, 1162, 1058, 928, 823, 756, 697 cm^{-1}. HRMS (APCI$^+$): (m/z) calcd for $C_{22}H_{21}F_2Si^+$ (M+H$^+$), 351.1375; found, 351.1371.

(*E*)-(3-Fluorophenyl)(phenyl)(3-phenylbut-2-en-1-yl)silane (3aa)

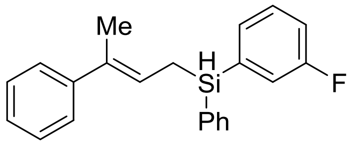

In an Ar-filled dry box, $Cu(OAc)_2$ (1.8 mg, 0.01 mmol), Xantphos (8.67 mg, 0.015 mmol) and THF (0.4 mL) were added to a 4-mL screw-capped vial and stirred for 15 mins. Then **1a** (0.2 mmol, 26 mg) and (3-Fluorophenyl)(phenyl)silane (0.24 mmol, 48 mg) were added. The vial was sealed with a cap containing a PTFE septum. The reaction mixture was stirred at room temperature for 12 h and the resulting solution was concentrated in vacuum. The title compound was isolated (56mg, 84% yield) as a colorless oil after chromatography on silica with PE/EA=100:1. ^{1}H NMR (600 MHz, $CDCl_3$, 25 ºC) δ 7.66–7.59 (m, 2H), 7.49–7.35 (m, 5H), 7.31 (m, 5H), 7.26–7.20 (m, 1H), 7.17–7.08 (m, 1H), 5.91 (t, *J* = 8.5 Hz, 1H), 4.96 (t, *J* = 3.5 Hz, 1H), 2.30 (dd, *J* = 8.5, 3.6 Hz, 2H), 1.89 (s, 3H). ^{13}C NMR (151 MHz, $CDCl_3$, 25 ºC) δ 162.7 (d, *J* = 248.6 Hz), 144.1, 137.0 (d, *J* = 4.3 Hz), 135.3, 134.6, 133.2, 130.9 (d, *J* = 3.2 Hz), 130.1, 129.9 (d, *J* = 7.1 Hz), 128.3, 128.3, 126.5, 125.6, 122.5, 121.6 (d, *J* = 19.0 Hz), 116.9 (d, *J* = 21.1 Hz), 15.9, 15.8. ^{19}F NMR (565 MHz, $CDCl_3$, 25 ºC) δ –110.2 – –119.8 (m, 1F). IR (ATR): ν 3052, 2937, 1945, 1878, 1774, 1688, 1599, 1476, 1408, 1259, 1159, 1051, 928, 872, 756, 697 cm^{-1}. HRMS (APCI$^+$): (m/z) calcd for $C_{22}H_{22}FSi^+$ (M+H$^+$), 333.1469; found, 333.1461.

(*E*)-(4-Fluorophenyl)(phenyl)(3-phenylbut-2-en-1-yl)silane (3ab)

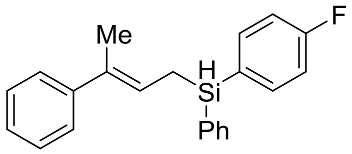

In an Ar-filled dry box, $Cu(OAc)_2$ (1.8 mg, 0.01 mmol), Xantphos (8.67 mg, 0.015 mmol) and THF (0.4 mL) were added to a 4-mL screw-capped vial and stirred for 15 mins. Then **1a** (0.2 mmol, 26 mg) and (4-Fluorophenyl)(phenyl)silane (0.24 mmol, 48 mg) were added. The vial was sealed with a cap containing a PTFE septum. The reaction mixture was stirred at room temperature for 12 h and the resulting solution was concentrated in vacuum. The title compound was isolated (55 mg, 83% yield) as a colorless oil after chromatography on silica with PE/EA=100:1. ^{1}H NMR (600 MHz, $CDCl_3$, 25 ºC) δ 7.60–7.52 (m, 4H), 7.45–7.39 (m, 1H), 7.37 (t, *J* = 7.2 Hz, 2H), 7.31–7.23 (m, 4H), 7.21–7.15 (m, 1H), 7.11–7.01 (m, 2H), 5.87 (t, *J* = 8.5 Hz, 1H), 4.91 (t, *J* = 3.5 Hz, 1H), 2.24 (dd, *J* = 8.6, 3.6 Hz, 2H), 1.82 (s, 3H). ^{13}C NMR (151 MHz, $CDCl_3$, 25 ºC) δ 164.3 (d, *J* = 249.1 Hz), 144.1, 137.3 (d, *J* = 7.6 Hz), 135.3, 134.4, 133.7, 130.0, 129.4 (d, *J* = 3.8 Hz), 128.3 (d, *J* = 9.4 Hz), 126.5, 125.6, 122.7, 115.5, 115.3, 16.0, 15.9. ^{19}F NMR (565 MHz, $CDCl_3$, 25 ºC) δ −110.6 – −110.7 (m,1F).IR (ATR): ν 3131, 2937, 1956, 1886, 1763, 1684, 1599, 1427, 1379, 1300, 1263, 1159, 924, 827, 700 cm^{-1}. HRMS (APCI+): (m/z) calcd for $C_{22}H_{22}FSi^+$ (M+H$^+$), 333.1469; found, 333.1469.

(*E*)-phenyl(3-phenylbut-2-en-1-yl)silane (3ac)

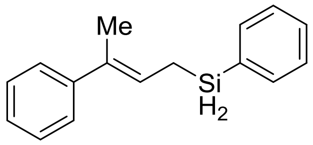

In an Ar-filled dry box, $Cu(OAc)_2$ (1.8 mg, 0.01 mmol), Xantphos (8.67 mg, 0.015 mmol) and THF (0.4 mL) were added to a 4-mL screw-capped vial and stirred at 30ºC for 15 mins. Then **1a** (0.2 mmol, 26 mg) and $PhSiH_3$ (0.4 mmol, 43 mg) were added. The vial was sealed with a cap containing a PTFE septum. The reaction mixture was stirred at 30 ºC for 3 h and the resulting solution was concentrated in vacuum. The title compound was isolated (31 mg, 66% yield) as a colorless oil after chromatography on silica with PE. ^{1}H NMR (400 MHz, $CDCl_3$, 25 ºC) δ 7.62–7.57 (m, 2H), 7.45–7.27 (m, 7H), 7.23–7.16 (m, 1H), 5.90 (td, *J* = 8.5, 1.4 Hz, 1H), 4.36 (t, *J* = 3.6 Hz, 2H), 2.05 (dt, *J* = 7.5, 3.7 Hz, 2H), 1.93 (s, 3H). ^{13}C NMR (151 MHz, $CDCl_3$, 25 ºC) δ 144.1, 135.4, 134.2, 132.2, 129.9, 128.3, 128.2, 126.5, 125.6, 123.2, 15.7, 13.8. IR (ATR): 3067,2922, 2847, 2120, 1446, 1114, 961, 797, 730, 697. HRMS (ESI$^+$): (m/z) calcd for $C_{16}H_{19}Si^+$ (M+H$^+$), 239.1251; found, 239.1243.

(4-methyl-3-phenylpent-2-en-1-yl)diphenylsilane (3ad)

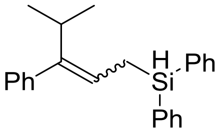

In an Ar-filled dry box, $Cu(OAc)_2$ (1.8 mg, 0.01 mmol), Xantphos (8.67 mg, 0.015 mmol) and THF(0.4 mL) were added to a 4-mL screw-capped vial and stirred for 15 mins. Then allene (0.2 mmol) and Ph_2SiH_2 (1.2 eq, 0.24 mmol) were added. The vial was sealed with a cap containing a PTFE septum. The reaction mixture was stirred at room temperature for 12 h and the resulting solution was concentrated in vacuum. The yield (79% of both *E* and *Z*-type product) and *Z*/*E* ratio

(E/Z=66:34) was determined by HNMR analysis of unpurified reaction mixtures, 1,2-dibromoethane is used as the internal label. ^{1}H NMR (600 MHz, $CDCl_3$, 25 ºC) δ 7.64–7.56 (m, 2H), 7.50–7.27 (m, 8H), 7.26–7.14 (m, 3H), 7.08–6.76 (m, 2H), 5.53–5.28 (m, 1H, E/Z=0.66/0.34), 4.95–4.75 (m, 1H), 2.97–2.40 (m, 1H), 2.28–1.80 (m, 2H), 0.94–0.82 (m, 6H). ^{13}C NMR (151 MHz, $CDCl_3$, 25 ºC) δ 147.3, 146.2, 143.4, 141.0, 135.4, 135.4, 134.0, 134.0, 129.9, 129.7, 129.27, 128.9, 128.1, 127.9, 127.9, 127.5, 126.2, 126.1, 122.8, 118.8, 36.4, 29.0, 22.0, 21.6, 14.9, 14.6. IR (ATR): ν 3049, 3015, 2959, 2929, 2870, 2120, 1490, 1427, 1151, 1114, 805, 734, 700. HRMS (ESI$^+$): (m/z) calcd for $C_{24}H_{27}Si^+$ (M+H$^+$), 343.1877; found, 343.1879.

1.4 General Procedure for the Cu-catalyzed Hydrosilylation of Monosubstituted Allenes

In an Ar-filled dry box, $Cu(OAc)_2$ (1.8 mg, 0.01 mmol), Xantphos (8.67 mg, 0.015 mmol) and THF (0.4 mL) were added to a 4-mL screw-capped vial and stirred for 15 mins. Then Monosubstituted allenes (0.2 mmol) and Ph_2SiH_2 (1.1 equiv., 0.22 mmol, 40 mg) were added. The vial was sealed with a cap containing a PTFE septum. The reaction mixture was stirred at room temperature for 6 h and the resulting solution was concentrated in vacuum. The crude product was purified by column chromatography on silica gel with a mixture of ethyl acetate and hexane as eluent. The conditions for flash chromatography and data for characterization of the products are listed below.

Cinnamyldiphenylsilane (5a)

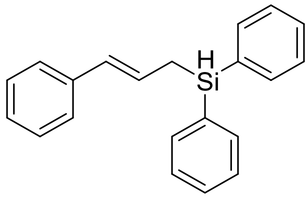

In an Ar-filled dry box, $Cu(OAc)_2$ (1.8 mg, 0.01 mmol), Xantphos (8.67 mg, 0.015 mmol) and THF(0.4 mL) were added to a 4-mL screw-capped vial and stirred for 15 mins. Then **4a** (0.2 mmol, 23 mg) and Ph_2SiH_2 (0.22 mmol, 40 mg) were added. The vial was sealed with a cap containing a PTFE septum. The reaction mixture was stirred at room temperature for 6 h and the resulting solution was concentrated in vacuum. The title compound was isolated (48 mg, 80% yield) as a colorless oil after chromatography on silica with PE/EA (50:1). Rf=0.5. ^{1}H NMR (400 MHz, $CDCl_3$, 25 ºC) δ 7.67–7.54 (m, 4H), 7.48–7.34 (m, 2H), 7.31–7.23 (m, 6H), 7.21–7.12 (m, 4H), 6.34 (d, J = 15.9 Hz, 1H), 6.29 (dt, J = 15.8, 7.0 Hz, 1H), 4.95 (t, J = 3.5 Hz, 1H), 2.32 (dd, J = 6.9, 3.5 Hz, 2H). ^{13}C NMR (101 MHz, $CDCl_3$, 25 ºC) δ 138.2, 135.4, 133.7, 130.3, 129.9, 128.6, 128.2, 126.7, 126.1, 125.8, 19.3. IR (ATR): ν 3066, 2918, 1956, 1885, 1774, 1643, 1488, 1397, 1267, 1155, 1028, 916, 849, 734, 700 cm^{-1}. HRMS (ESI$^+$): (m/z) calcd for $C_{21}H_{21}Si^+$ (M+H$^+$), 301.1407; found, 301.1410.

(*E*)-Diphenyl(3-(m-tolyl)allyl)silane (5b)

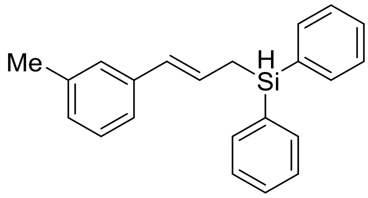

In an Ar-filled dry box, $Cu(OAc)_2$ (1.8 mg, 0.01 mmol), Xantphos (8.67 mg, 0.015 mmol) and

THF(0.4 mL) were added to a 4-mL screw-capped vial and stirred for 15 mins. Then **4b** (0.2 mmol, 26 mg) and Ph_2SiH_2 (0.22 mmol, 40 mg) were added. The vial was sealed with a cap containing a PTFE septum. The reaction mixture was stirred at room temperature for 6 h and the resulting solution was concentrated in vacuum. The title compound was isolated (55 mg, 88% yield) as a colorless oil after chromatography on silica with PE/EA (50:1). Rf=0.5. ^{1}H NMR (600 MHz, $CDCl_3$, 25 ºC) δ 7.65–7.54 (m, 4H), 7.47–7.33 (m, 6H), 7.16 (t, J = 7.5 Hz, 1H), 7.11–6.94 (m, 3H), 6.31 (d, J = 15.8 Hz, 1H), 6.22 (dt, J = 15.6, 7.8 Hz, 1H), 4.93 (t, J = 3.5 Hz, 1H), 2.32 (s, 3H), 2.30 (dd, J = 7.1, 3.5 Hz, 2H). ^{13}C NMR (151 MHz, $CDCl_3$, 25 ºC) δ 138.1, 138.1, 135.4, 133.7, 130.3, 129.9, 128.5, 128.2, 127.5, 126.6, 125.9, 122.9, 21.6, 19.3. IR (ATR): ν 3049, 2922, 1956, 1897, 1770, 1684, 1587, 1427, 1330, 1263, 1155, 1028, 961, 868, 734, 700 cm^{-1}. HRMS ($APCI^+$): (m/z) calcd for $C_{22}H_{23}Si^+$ ($M+H^+$), 315.1564; found, 315.1556.

(*E*)-Diphenyl(3-(p-tolyl)allyl)silane (5c)

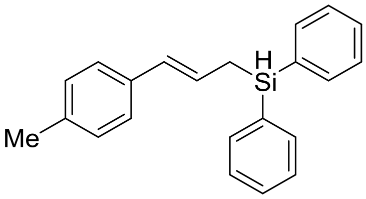

In an Ar-filled dry box, $Cu(OAc)_2$ (1.8 mg, 0.01 mmol), Xantphos (8.67 mg, 0.015 mmol) and THF(0.4 mL) were added to a 4-mL screw-capped vial and stirred for 15 mins. Then **4c** (0.2 mmol, 26 mg) and Ph_2SiH_2 (0.22 mmol, 40 mg) were added. The vial was sealed with a cap containing a PTFE septum. The reaction mixture was stirred at room temperature for 6 h and the resulting solution was concentrated in vacuum. The title compound was isolated (51 mg, 82% yield) as a colorless oil after chromatography on silica with PE/EA (50:1). Rf=0.5. ^{1}H NMR (600 MHz, $CDCl_3$, 25 ºC) δ 7.67–7.57 (m, 4H), 7.47–7.34 (m, 6H), 7.19–7.05 (m, 4H), 6.30 (d, J = 15.8 Hz, 1H), 6.26 (dt, J = 15.6, 7.8 Hz, 1H), 4.94 (t, J = 3.5 Hz, 1H), 2.33 (s, 3H), 2.30 (dd, J = 7.8, 3.5 Hz, 2H). ^{13}C NMR (151 MHz, $CDCl_3$, 25 ºC) δ 136.3, 135.4, 135.4, 133.7, 130.1, 129.9, 129.3, 128.2, 125.7, 124.9, 21.3, 19.2. IR (ATR): ν 3067, 2918, 1956, 1885, 1774, 1643, 1487, 1397, 1267, 1155, 1028, 916, 849, 734, 700 cm^{-1}. HRMS ($APCI^+$): (m/z) calcd for $C_{22}H_{23}Si^+$ ($M+H^+$), 315.1564; found, 315.1554.

(*E*)-Diphenyl(3-(o-tolyl)allyl)silane (5d)

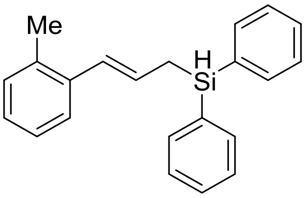

In an Ar-filled dry box, $Cu(OAc)_2$ (1.8 mg, 0.01 mmol), Xantphos (8.67 mg, 0.015 mmol) and THF(0.4 mL) were added to a 4-mL screw-capped vial and stirred for 15 mins. Then **4d** (0.2 mmol, 26 mg) and Ph_2SiH_2 (0.22 mmol, 40 mg) were added. The vial was sealed with a cap containing a PTFE septum. The reaction mixture was stirred at room temperature for 6 h and the resulting solution was concentrated in vacuum. The title compound was isolated (48 mg, 77% yield) as a

colorless oil after chromatography on silica with PE/EA (50:1). Rf=0.5. ^{1}H NMR (600 MHz, $CDCl_3$, 25 ºC) δ 7.67–7.59 (m, 4H), 7.47–7.37 (m, 6H), 7.33 (d, *J* = 6.7 Hz, 2H), 7.13 (m, 3H), 6.51 (d, *J* = 15.6 Hz, 1H), 6.17 (dt, *J* = 15.8, 8.0 Hz, 1H), 4.97 (t, *J* = 3.4 Hz, 1H), 2.36 (ddd, *J* = 8.0, 3.4, 1.4 Hz, 2H), 2.21 (s, 3H). ^{13}C NMR (151 MHz, $CDCl_3$, 25 ºC) δ 137.3, 135.4, 134.8, 133.7, 130.2, 129.9, 128.3, 128.2, 127.3, 126.7, 126.1, 125.4, 19.9, 19.6. IR (ATR): ν 3049, 2948, 2855, 2728, 2124, 1953, 1882, 1763, 1647, 1479, 1326, 1263, 1155, 913, 801, 700. cm^{-1}. HRMS ($APCI^+$): (m/z) calcd for $C_{22}H_{23}Si^+$ ($M+H^+$), 315.1564; found, 315.1560.

(*E*)-(3-(4-(*Tert*-butyl)phenyl)allyl)diphenylsilane (5e)

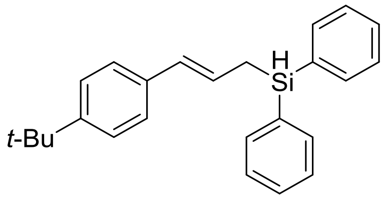

In an Ar-filled dry box, $Cu(OAc)_2$ (1.8 mg, 0.01 mmol), Xantphos (8.67 mg, 0.015 mmol) and THF(0.4 mL) were added to a 4-mL screw-capped vial and stirred for 15 mins. Then **4e** (0.2 mmol, 34 mg) and Ph_2SiH_2 (0.22 mmol, 40 mg) were added. The vial was sealed with a cap containing a PTFE septum. The reaction mixture was stirred at room temperature for 6 h and the resulting solution was concentrated in vacuum. The title compound was isolated (53 mg, 75% yield) as a colorless oil after chromatography on silica with PE/EA (50:1). Rf=0.5. ^{1}H NMR (600 MHz, $CDCl_3$, 25 ºC) δ 7.63–7.56 (m, 4H), 7.45–7.35 (m, 6H), 7.34–7.27 (m, 2H), 7.22–7.16 (m, 2H), 6.32 (d, *J* = 15.8 Hz, 1H), 6.23 (dt, *J* = 15.7, 7.8 Hz, 1H), 4.92 (t, *J* = 3.4 Hz, 1H), 2.29 (dd, *J* = 6.6, 3.5 Hz, 2H), 1.31 (s, 9H). ^{13}C NMR (151 MHz, $CDCl_3$, 25 ºC) δ 149.6, 135.5, 135.4, 133.7, 129.9, 129.9, 128.2, 125.5, 125.5, 125.3, 34.6, 31.4, 19.2. IR (ATR): ν 3067, 2959, 2128, 1640, 1587, 1461, 1394, 1267, 1155, 1017, 961, 872, 801, 734, 700 cm^{-1}. HRMS ($APCI^+$): (m/z) calcd for $C_{25}H_{29}Si^+$ ($M+H^+$), 357.2033; found, 357.2029.

(*E*)-(3-(4-Methoxyphenyl)allyl)diphenylsilane (5f)

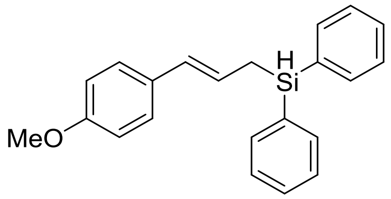

In an Ar-filled dry box, $Cu(OAc)_2$ (1.8 mg, 0.01 mmol), Xantphos (8.67 mg, 0.015 mmol) and THF(0.4 mL) were added to a 4-mL screw-capped vial and stirred for 15 mins. Then **4f** (0.2 mmol, 29 mg) and Ph_2SiH_2 (0.22 mmol, 40 mg) were added. The vial was sealed with a cap containing a PTFE septum. The reaction mixture was stirred at room temperature for 6 h and the resulting solution was concentrated in vacuum. The title compound was isolated (54 mg, 82% yield) as a colorless oil after chromatography on silica with PE/EA (20:1). Rf=0.5. ^{1}H NMR (400 MHz, $CDCl_3$, 25 ºC) δ 7.67–7.52 (m, 4H), 7.48–7.32 (m, 6H), 7.19 (d, *J* = 8.8 Hz, 2H), 6.82 (d, *J* = 8.7 Hz, 2H), 6.29 (d, *J* = 15.7 Hz, 1H), 6.12 (dt, *J* = 15.7, 7.8 Hz, 1H), 4.93 (t, *J* = 3.4 Hz, 2H), 3.80 (s, 3H), 2.28 (dd, *J* = 7.9, 4.7 Hz, 2H). ^{13}C NMR (151 MHz, $CDCl_3$, 25 ºC) δ 158.5, 135.4, 133.8, 131.1, 129.9, 129.6, 128.1, 126.8, 123.8, 113.9, 55.4, 19.1. IR (ATR): ν 3067, 2832, 2124, 1960, 1886, 1774, 1688, 1509, 1464, 1285, 1177, 1036, 961, 812, 738, 700 cm^{-1}. HRMS ($APCI^+$): (m/z) calcd for

$C_{28}H_{27}OSi^+$, 407.1826; found, 407.1828.

(*E*)-(3-Mesitylallyl)diphenylsilane (5g)

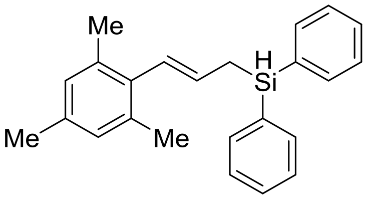

In an Ar-filled dry box, $Cu(OAc)_2$ (1.8 mg, 0.01 mmol), Xantphos (8.67 mg, 0.015 mmol) and THF(0.4 mL) were added to a 4-mL screw-capped vial and stirred for 15 mins. Then **4g** (0.2 mmol, 32 mg) and Ph_2SiH_2 0.22 mmol, 40 mg) were added. The vial was sealed with a cap containing a PTFE septum. The reaction mixture was stirred at room temperature for 6 h and the resulting solution was concentrated in vacuum. The title compound was isolated (54 mg, 82% yield) as a colorless oil after chromatography on silica with PE/EA (50:1). Rf=0.5. ^{1}H NMR (600 MHz, $CDCl_3$, 25 ºC) δ 7.67–7.58 (m, 4H), 7.48–7.33 (m, 6H), 6.81 (s, 2H), 6.24 (d, *J* = 16.0 Hz, 1H), 5.70 (dt, *J* = 15.9, 7.9 Hz, 1H), 4.96 (t, *J* = 3.6 Hz, 1H), 2.34 (ddd, *J* = 7.9, 3.6, 1.4 Hz, 2H), 2.25 (s, 3H), 2.11 (s, 6H). ^{13}C NMR (151 MHz, $CDCl_3$, 25 ºC) δ 136.0, 135.6, 135.4, 134.9, 133.8, 130.2, 129.9, 128.5, 128.2, 127.7, 21.0, 20.9, 19.5. IR (ATR): ν 3049, 2948, 2855, 2728, 2124, 1953, 1882, 1763, 1647, 1479, 1326, 1263, 1155, 969, 853, 700. HRMS (APCI$^+$): (m/z) calcd for $C_{24}H_{27}Si^+$,343.1877; found, 343.1871.

(*E*)-(3-(4-Fluorophenyl)allyl)diphenylsilane (5h)

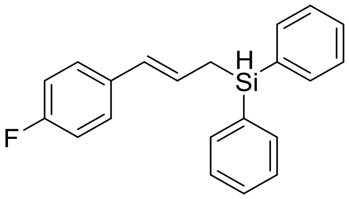

In an Ar-filled dry box, $Cu(OAc)_2$ (1.8 mg, 0.01 mmol), Xantphos (8.67 mg, 0.015 mmol) and THF(0.4 mL) were added to a 4-mL screw-capped vial and stirred for 15 mins. Then **4h** (0.2 mmol, 27 mg) and Ph_2SiH_2 (0.22 mmol, 40 mg) were added. The vial was sealed with a cap containing a PTFE septum. The reaction mixture was stirred at room temperature for 6 h and the resulting solution was concentrated in vacuum. The title compound was isolated (52 mg, 82% yield) as a colorless oil after chromatography on silica with PE/EA (50:1). Rf=0.5. ^{1}H NMR (600 MHz, $CDCl_3$, 25 ºC) δ 7.63–7.54 (m, 4H), 7.48–7.31 (m, 6H), 7.21–7.13 (m, 2H), 6.94 (t, *J* = 8.7 Hz, 2H), 6.27 (d, *J* = 15.7 Hz, 1H), 6.16 (dt, *J* = 15.8, 7.9 Hz, 1H), 4.92 (t, *J* = 3.5 Hz, 1H), 2.28 (ddd, *J* = 7.9, 3.5, 1.2 Hz, 2H). ^{13}C NMR (151 MHz, $CDCl_3$, 25 ºC) δ 161.8 (d, *J* = 245.3 Hz), 135.4, 134.3 (d, *J* = 3.2 Hz), 133.6, 129.9, 129.1, 128.2, 127.1 (d, *J* = 7.8 Hz), 125.8 (d, *J* = 2.2 Hz), 115.4 (d, *J* = 21.4 Hz), 19.2. ^{19}F NMR (565 MHz, $CDCl_3$, 25 ºC) δ –116.1 – –116.2 (m, 1F). IR (ATR): ν 3067, 2881, 2128, 1886, 1766, 1643, 1505, 1427, 1330, 1230, 1155, 1013, 961, 816, 734 cm^{-1}. HRMS (APCI$^+$): (m/z) calcd for $C_{21}H_{20}FSi^+$, 319.1313; found, 319.1307.

(*E*)-(3-(3-Fluorophenyl)allyl)diphenylsilane (5i)

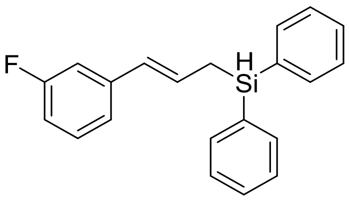

In an Ar-filled dry box, $Cu(OAc)_2$ (1.8 mg, 0.01 mmol), Xantphos (8.67 mg, 0.015 mmol) and THF(0.4 mL) were added to a 4-mL screw-capped vial and stirred for 15 mins. Then **4i** (0.2 mmol, 26 mg) and Ph_2SiH_2 (0.22 mmol, 40 mg) were added. The vial was sealed with a cap containing a PTFE septum. The reaction mixture was stirred at room temperature for 6 h and the resulting solution was concentrated in vacuum. The title compound was isolated (49 mg, 77% yield) as a colorless oil after chromatography on silica with PE/EA (50:1). Rf=0.5. ^{1}H NMR (600 MHz, $CDCl_3$, 25 ºC) δ 7.63–7.55 (m, 4H), 7.47–7.33 (m, 6H), 7.23–7.16 (m, 1H), 7.01–6.79 (m, 3H), 6.34–6.21 (m, 2H), 4.92 (t, *J* = 3.5 Hz, 1H), 2.30 (dd, *J* = 6.4, 3.5 Hz, 2H). ^{13}C NMR (151 MHz, $CDCl_3$, 25 ºC) δ 163.2 (d, *J* = 244.3 Hz), 140.5 (d, *J* = 7.7 Hz), 135.3, 133.4, 130.0, 129.9 (d, *J* = 8.4 Hz), 129.2 (d, *J* = 2.7 Hz), 128.2, 127.8, 121.6 (d, *J* = 2.6 Hz), 113.4 (d, *J* = 21.3 Hz), 112.2 (d, *J* = 21.6 Hz), 19.4. ^{19}F NMR (565 MHz, $CDCl_3$, 25 ºC) δ –113.9 – –114.0 (m). IR (ATR): ν 3067, 2959, 2124, 1956, 1886, 1770, 1640, 1580, 1487, 1326, 1267, 1140, 961, 872, 734, 700. HRMS (APCI$^+$): (m/z) calcd for $C_{21}H_{20}FSi^+$, 319.1313; found, 319.1312.

(*E*)-(3-(2-Fluorophenyl)allyl)diphenylsilane (5j)

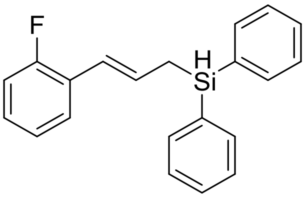

In an Ar-filled dry box, $Cu(OAc)_2$ (1.8 mg, 0.01 mmol), Xantphos (8.67 mg, 0.015 mmol) and THF(0.4 mL) were added to a 4-mL screw-capped vial and stirred for 15 mins. Then **4j** (0.2 mmol, 26 mg) and Ph_2SiH_2 (0.22 mmol, 40 mg) were added. The vial was sealed with a cap containing a PTFE septum. The reaction mixture was stirred at room temperature for 6 h and the resulting solution was concentrated in vacuum. The title compound was isolated (42 mg, 66% yield) as a colorless oil after chromatography on silica with PE/EA (50:1). Rf=0.5. ^{1}H NMR (600 MHz, $CDCl_3$, 25 ºC) δ 7.64–7.53 (m, 4H), 7.48–7.35 (m, 6H), 7.34–7.29 (m, 1H), 7.17–7.09 (m, 1H), 7.07–6.95 (m, 2H), 6.50 (d, *J* = 15.9 Hz, 1H), 6.36 (dt, *J* = 15.9, 8.0 Hz, 1H), 4.96 (t, *J* = 3.5 Hz, 1H), 2.36 (dd, *J* = 8.1, 3.5 Hz, 2H). ^{13}C NMR (151 MHz, $CDCl_3$, 25 ºC) δ 159.8 (d, *J* = 248.0 Hz), 135.4, 133.5, 129.9, 129.0 (d, *J* = 4.6 Hz), 128.2, 127.8 (d, *J* = 8.2 Hz), 126.9 (d, *J* = 4.2 Hz), 125.9 (d, *J* = 12.5 Hz), 124.1 (d, *J* = 3.4 Hz), 122.5, 115.7 (d, *J* = 22.3 Hz), 19.9. IR (ATR): ν 3067, 2926, 1956, 1818, 1640, 1576, 1487, 1330, 1230, 1192, 1028, 965, 805, 752, 700. HRMS (APCI$^+$): (m/z) calcd for $C_{21}H_{20}FSi^+$, 319.1313; found, 319.1307.

(*E*)-(3-(4-Chlorophenyl)allyl)diphenylsilane (5k)

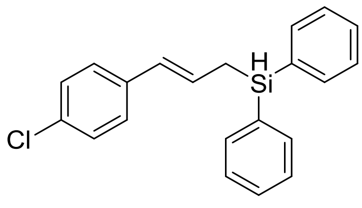

In an Ar-filled dry box, $Cu(OAc)_2$ (1.8 mg, 0.01 mmol), Xantphos (8.67 mg, 0.015 mmol) and THF(0.4 mL) were added to a 4-mL screw-capped vial and stirred for 15 mins. Then **4k** (0.2 mmol, 30 mg) and Ph_2SiH_2 (0.22 mmol, 40 mg) were added. The vial was sealed with a cap containing a PTFE septum. The reaction mixture was stirred at room temperature for 6 h and the resulting solution was concentrated in vacuum. The title compound was isolated (53 mg, 80% yield) as a colorless oil after chromatography on silica with PE/EA (50:1). Rf=0.5. ^{1}H NMR (600 MHz, $CDCl_3$, 25 ºC) δ 7.62–7.55 (m, 4H), 7.43 (m, 2H), 7.41–7.34 (m, 4H), 7.24–7.12 (m, 4H), 6.38–6.12 (m, 2H), 4.93 (t, J = 3.5 Hz, 1H), 2.30 (dd, J = 6.4, 3.5 Hz, 2H). ^{13}C NMR (151 MHz, $CDCl_3$, 25 ºC) δ 136.6, 135.4, 133.5, 132.1, 130.0, 129.0, 128.7, 128.2, 126.9, 19.4. IR (ATR): ν 3067, 2929, 2881, 1956, 1893, 1774, 1640, 1587, 1490, 1397, 1151, 1013, 961, 808, 734, 667 cm^{-1}. HRMS (APCI$^+$): (m/z) calcd for $C_{21}H_{20}ClSi^+$ (M+H$^+$), 335.1017; found, 335.1010.

(*E*)-(3-(2-Chlorophenyl)allyl)diphenylsilane (5l)

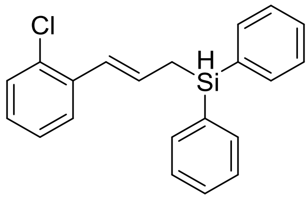

In an Ar-filled dry box, $Cu(OAc)_2$ (1.8 mg, 0.01 mmol), Xantphos (8.67 mg, 0.015 mmol) and THF(0.4 mL) were added to a 4-mL screw-capped vial and stirred for 15 mins. Then **4l** (0.2 mmol, 30 mg) and Ph_2SiH_2 (0.22 mmol, 40 mg) were added. The vial was sealed with a cap containing a PTFE septum. The reaction mixture was stirred at room temperature for 6 h and the resulting solution was concentrated in vacuum. The title compound was isolated (41 mg, 61% yield) as a colorless oil after chromatography on silica with PE/EA (50:1). Rf=0.5. ^{1}H NMR (600 MHz, $CDCl_3$, 25 ºC) δ 7.64–7.57 (m, 4H), 7.46–7.33 (m, 7H), 7.32–7.27 (m, 1H), 7.18–7.06 (m, 2H), 6.70 (d, J = 15.7 Hz, 1H), 6.23 (dt, J = 15.9, 8.1 Hz, 1H), 4.94 (t, J = 3.5 Hz, 1H), 2.36 (ddd, J = 8.1, 3.5, 1.4 Hz, 2H). ^{13}C NMR (151 MHz, $CDCl_3$, 25 ºC) δ 136.2, 135.4, 133.5, 132.4, 129.9, 129.6, 129.3, 128.2, 127.7, 126.8, 126.6, 126.5, 19.8. IR (ATR): ν 3067, 2922, 1953, 1882, 1774, 1636, 1587, 1468, 1326, 1267, 1151, 1051, 961, 868, 734, 700 cm^{-1}. HRMS (APCI$^+$): (m/z) calcd for $C_{21}H_{20}ClSi^+$ (M+H$^+$), 335.1017; found, 335.1009.

(*E*)-(3-(Naphthalen-2-yl)allyl)diphenylsilane (5m)

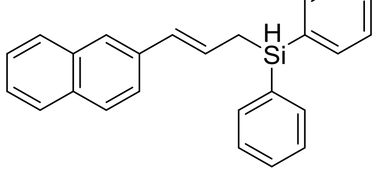

In an Ar-filled dry box, $Cu(OAc)_2$ (1.8 mg, 0.01 mmol), Xantphos (8.67 mg, 0.015 mmol) and THF(0.4 mL) were added to a 4-mL screw-capped vial and stirred for 15 mins. Then **4m** (0.2 mmol,

33 mg) and Ph_2SiH_2 (0.22 mmol, 40 mg) were added. The vial was sealed with a cap containing a PTFE septum. The reaction mixture was stirred at room temperature for 6 h and the resulting solution was concentrated in vacuum.The title compound was isolated (61 mg, 87% yield) as a colorless oil after chromatography on silica with PE/EA (50:1). Rf=0.5. ^{1}H NMR (600 MHz, $CDCl_3$, 25 ºC) δ 7.80–7.69 (m, 3H), 7.67–7.55 (m, H), 7.50–7.35 (m, 9H), 6.48 (d, *J* = 15.8 Hz, 1H), 6.40 (dt, *J* = 15.6, 7.8 Hz, 1H), 4.96 (t, *J* = 3.5 Hz, 1H), 2.36 (dd, *J* = 7.3, 2.9 Hz, 2H). ^{13}C NMR (151 MHz, Chloroform-*d*) δ 135.6, 135.4, 133.8, 133.6, 132.6, 130.4, 129.9, 128.2, 128.1, 127.9, 127.7, 126.7, 126.2, 125.5, 125.0, 123.6, 19.5. IR (ATR): ν 3052, 2124, 1953, 1818, 1695, 1595, 1427, 1375, 1267, 1118, 1013, 961, 894, 849, 738, 700 cm^{-1}. HRMS (APCI$^+$): (m/z) calcd for C25H23Si$^+$, 351.1564; found, 351.1562.

(*E*)-(3-(4-(Benzyloxy)phenyl)allyl)diphenylsilane (5o)

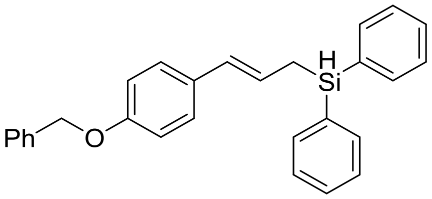

In an Ar-filled dry box, $Cu(OAc)_2$ (1.8 mg, 0.01 mmol), Xantphos (8.67 mg, 0.015 mmol) and THF(0.4 mL) were added to a 4-mL screw-capped vial and stirred for 15 mins. Then **4o** (0.2 mmol, 44 mg) and Ph_2SiH_2 (0.22 mmol, 40 mg) were added. The vial was sealed with a cap containing a PTFE septum. The reaction mixture was stirred at room temperature for 6 h and the resulting solution was concentrated in vacuum. The title compound was isolated (68 mg, 84% yield) as a colorless oil after chromatography on silica with PE/EA (50:1). Rf=0.4. ^{1}H NMR (600 MHz, $CDCl_3$, 25 ºC) δ 7.65–7.56 (m, 4H), 7.49–7.32 (m, 11H), 7.19 (d, *J* = 8.7 Hz, 2H), 6.89 (d, *J* = 8.7 Hz, 2H), 6.28 (d, *J* = 15.8 Hz, 1H), 6.13 (dt, *J* = 15.8, 7.9 Hz, 1H), 5.06 (s, 2H), 4.93 (t, *J* = 3.4 Hz, 1H), 2.29 (dd, *J* = 7.3, 2.8 Hz, 2H). ^{13}C NMR (151 MHz, $CDCl_3$, 25 ºC) δ 157.7, 137.2, 135.4, 133.8, 131.3, 129.9, 129.6, 128.7, 128.1, 128.1, 127.4, 126.9, 123.9, 114.9, 70.1, 19.1. IR (ATR): ν 3067, 3026, 2922, 1956, 1818, 1774, 1640, 1505, 1453, 1379, 1285, 1174, 916, 849, 734, 700 cm^{-1}. HRMS (APCI$^+$): (m/z) calcd for $C_{28}H_{27}OSi^+$, 407.1826; found, 407.1828.

(*E*)-(3-([1,1'-Biphenyl]-4-yl)allyl)diphenylsilane (5p)

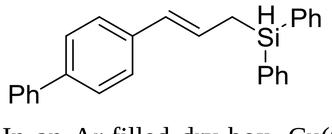

In an Ar-filled dry box, $Cu(OAc)_2$ (1.8 mg, 0.01 mmol), Xantphos (8.67 mg, 0.015 mmol) and THF(0.4 mL) were added to a 4-mL screw-capped vial and stirred for 15 mins. Then **4p** (0.2 mmol, 38 mg) and Ph_2SiH_2 (0.22 mmol, 40 mg) were added. The vial was sealed with a cap containing a PTFE septum. The reaction mixture was stirred at room temperature for 6 h and the resulting solution was concentrated in vacuum. The title compound was isolated (68 mg, 84% yield) as a colorless oil after chromatography on silica with PE/EA (50:1). Rf=0.4. ^{1}H NMR (600 MHz, $CDCl_3$, 25 ºC) δ 7.65–7.55 (m, 6H), 7.51 (d, *J* = 8.3 Hz, 2H), 7.47–7.36 (m, 8H), 7.32 (d, *J* = 8.2 Hz, 3H), 6.36 (d, *J* = 15.8 Hz, 1H), 6.32 (dt, *J* = 15.8, 7.9 Hz, 1H), 4.95 (t, *J* = 3.5 Hz, 1H), 2.33 (dd, *J* = 6.9, 3.5 Hz, 2H). ^{13}C NMR (151 MHz, $CDCl_3$, 25 ºC) δ 140.9, 139.4, 137.2, 135.4, 133.6, 129.9, 129.8,

128.9, 128.2, 127.3, 127.2, 127.0, 126.4, 126.2, 19.4. IR (ATR): ν 3053, 2124, 1953, 1904, 1819, 1625, 1595, 1427, 1375, 1267, 1118, 1013, 961, 849, 737, 700 cm^{-1}. HRMS (APCI$^+$): (m/z) calcd for $C_{27}H_{25}Si^+$, 377.1720; found, 377.1712.

Cinnamyl(4-fluorophenyl)(phenyl)silane (5q)

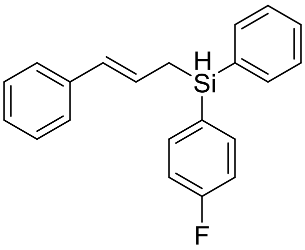

In an Ar-filled dry box, $Cu(OAc)_2$ (1.8 mg, 0.01 mmol), Xantphos (8.67 mg, 0.015 mmol) and THF(0.4 mL) were added to a 4-mL screw-capped vial and stirred for 15 mins. Then **4a** (0.2 mmol, 23 mg) and (4-Fluorophenyl)(phenyl)silane (0.22 mmol, 44 mg) were added. The vial was sealed with a cap containing a PTFE septum. The reaction mixture was stirred at room temperature for 6 h and the resulting solution was concentrated in vacuum. The title compound was isolated (47 mg, 74% yield) as a colorless oil after chromatography on silica with PE/EA (50:1). Rf=0.4. ^{1}H NMR (600 MHz, $CDCl_3$, 25 ºC) δ 7.60–7.51 (m, 4H), 7.47–7.34 (m, 3H), 7.29–7.19 (m, 4H), 7.19–7.12 (m, 1H), 7.10–7.03 (m, 2H), 6.30 (d, J = 15.7 Hz, 1H), 6.23 (dt, J = 15.6, 7.7 Hz, 1H), 4.91 (t, J = 3.4 Hz, 1H), 2.27 (dd, J = 7.5, 3.0 Hz, 2H). ^{13}C NMR (151 MHz, $CDCl_3$, 25 ºC) δ 164.3 (d, J = 249.1 Hz), 138.1, 137.4 (d, J = 7.6 Hz), 135.3, 133.4, 130.4, 130.1, 129.1 (d, J = 3.8 Hz), 128.6, 128.3, 126.7, 125.8, 115.5, 115.4, 19.3. ^{19}F NMR (565 MHz, CDCl3, 25 ºC) δ –110.47 – –110.53 (m, 1F). IR (ATR): ν 3063, 2926, 2124, 1960, 1815, 1640, 1587, 1498, 1386, 1233, 1107, 1073, 961, 864, 738, 700 cm^{-1}. HRMS (APCI$^+$): (m/z) calcd for $C_{21}H_{20}FSi^+$ (M+H$^+$), 319.1313; found, 319.1305.

Cinnamyl(3-fluorophenyl)(phenyl)silane (5r)

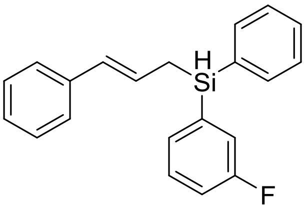

In an Ar-filled dry box, $Cu(OAc)_2$ (1.8 mg, 0.01 mmol), Xantphos (8.67 mg, 0.015 mmol) and THF(0.4 mL) were added to a 4-mL screw-capped vial and stirred for 15 mins. Then **4a** (0.2 mmol, 23 mg) and (3-Fluorophenyl)(phenyl)silane (0.22 mmol, 44 mg) were added. The vial was sealed with a cap containing a PTFE septum. The reaction mixture was stirred at room temperature for 6 h and the resulting solution was concentrated in vacuum. The title compound was isolated (53 mg, 83% yield) as a colorless oil after chromatography on silica with PE/EA (50:1). Rf=0.5. ^{1}H NMR (600 MHz, $CDCl_3$, 25 ºC) δ 7.61–7.52 (m, 2H), 7.46–7.31 (m, 5H), 7.28–7.20 (m, 5H), 7.19–7.12 (m, 1H), 7.12–7.04 (m, 1H), 6.32 (d, J = 15.7 Hz, 1H), 6.23 (dt, J = 15.7, 7.8 Hz, 1H), 4.91 (t, J = 3.5 Hz, 1H), 2.28 (dd, J = 7.3, 4.1 Hz, 2H). ^{13}C NMR (151 MHz, $CDCl_3$, 25 ºC) δ 162.7 (d, J = 249.0 Hz), 138.0, 136.7 (d, J = 4.3 Hz), 135.3, 132.9, 130.9 (d, J = 3.2 Hz), 130.6, 130.2, 130.0 (d,

J = 7.1 Hz), 128.6, 128.3, 126.8, 125.8, 125.6, 121.6 (d, J = 19.0 Hz), 116.9 (d, J = 20.9 Hz), 19.1. ^{19}F NMR (565 MHz, $CDCl_3$, 25 ºC) δ –112.9 – –113.0 (m, 1F). IR (ATR): ν 3063, 2926 1945, 1878, 1744, 1684, 1599, 1476, 1304, 1259, 1155, 1069, 961, 864, 782, 693 cm^{-1}. HRMS ($APCI^+$): (m/z) calcd for $C_{21}H_{20}FSi^+$ ($M+H^+$), 319.1313; found, 319.1306.

Cinnamylbis(4-fluorophenyl)silane (5s)

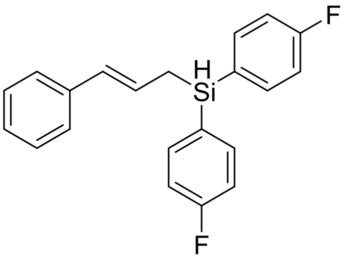

In an Ar-filled dry box, $Cu(OAc)_2$ (1.8 mg, 0.01 mmol), Xantphos (8.67 mg, 0.015 mmol) and THF(0.4 mL) were added to a 4-mL screw-capped vial and stirred for 15 mins. Then **4a** (0.2 mmol, 23 mg) and Bis(4-fluorophenyl)silane (0.22 mmol, 48 mg) were added. The vial was sealed with a cap containing a PTFE septum. The reaction mixture was stirred at room temperature for 6 h and the resulting solution was concentrated in vacuum. The title compound was isolated (54 mg, 81% yield) as a colorless oil after chromatography on silica with PE/EA (50:1). Rf=0.5. ^1H NMR (600 MHz, $CDCl_3$, 25 ºC) δ 7.5 (dd, J = 8.4, 6.2 Hz, 4H), 7.3– 7.2 (m, 4H), 7.19–7.14 (m, 1H), 7.11–7.03 (m, 4H), 6.29 (d, J = 15.8 Hz, 1H), 6.20 (dt, J = 15.7, 7.8 Hz, 1H), 4.91 (t, J = 3.4 Hz, 1H), 2.25 (dd, J = 8.4, 2.8 Hz, 2H). ^{13}C NMR (151 MHz, $CDCl_3$, 25 ºC) δ 164.4 (d, J = 249.6 Hz), 137.9, 137.3 (d, J = 7.6 Hz), 130.6, 128.9 (d, J = 3.8 Hz), 128.6, 126.8, 125.8, 125.4, 115.6 (d, J = 20.0 Hz), 19.3. ^{19}F NMR (565 MHz, $CDCl_3$, 25 ºC) δ –110.18 – –110.23 (m, 2F). IR (ATR): ν 3060, 2937, 2791, 2132, 1909, 1774, 1587, 1498, 1386, 1233, 1162, 1017, 961, 864, 749, 700 cm^{-1}. HRMS ($APCI^+$): (m/z) calcd for $C_{21}H_{19}F_2Si^+$ ($M+H^+$), 337.1219; found, 337.1211.

(3-Cyclohexylallyl)diphenylsilane (5t)

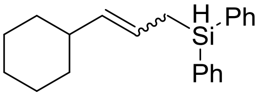

In an Ar-filled dry box, $Cu(OAc)_2$ (1.8 mg, 0.01 mmol), Xantphos (8.67 mg, 0.015 mmol) and THF(0.4 mL) were added to a 4-mL screw-capped vial and stirred for 15 mins. Then **4t** (0.4 mmol, 48 mg) and Ph_2SiH_2 (0.2 mmol, 36.4 mg) were added. The vial was sealed with a cap containing a PTFE septum. The reaction mixture was stirred at room temperature for 6 h and the resulting solution was concentrated in vacuum. The title compound yield (58% yield of *E* product, *E/Z*=89:11) was determamined by HNMR according to the literature data[8,10]. *E* product: ^1H NMR (600 MHz, $CDCl_3$, 25 ºC) δ 7.61–7.55 (m, 4H), 7.44–7.33 (m, 6H), 5.42 (dt, J = 15.3, 7.3 Hz, 1H), 5.30 (dd, J = 15.4, 6.9 Hz, 1H), 4.85 (t, J = 3.5 Hz, 1H), 2.05 (dd, J = 7.8, 3.5 Hz, 2H), 1.91–1.81 (m, 1H), 1.72–1.64 (m, 2H), 1.64–1.58 (m, 3H), 1.27–1.18 (m, 2H), 1.18–1.07 (m, 1H), 1.04–0.93 (m, 2H). ^{13}C NMR (151 MHz, $CDCl_3$, 25 ºC) δ 137.5, 135.4, 134.2, 129.7, 128.0, 121.9, 41.1, 33.4, 26.3, 26.2, 18.1. IR (ATR): ν 3067, 3011, 2922, 2847, 2120, 1446, 1155, 1114, 965, 797, 730, 700. HRMS ($APCI^+$): (m/z) calcd for $C_{21}H_{27}Si^+$ ($M+H^+$), 307.1877; found, 307.1871. *Z* product: ^1H NMR (600

MHz, $CDCl_3$, 25 ºC) δ 5.35–5.31 (m, 1H), 5.18–5.10 (m, 1H), 2.17–2.08 (m, 3H), other signals are overlapping with those of *E*-isomers. *Z* product was known compound, and the characteristic spectroscopic data for this product matched the literature data[10].

Cinnamyl(phenyl)silane (5u)

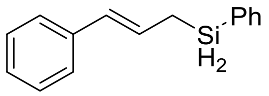

In an Ar-filled dry box, $Cu(OAc)_2$ (0.9 mg, 0.005 mmol), Xantphos (4.3 mg, 0.0075 mmol) and THF(0.4 mL) were added to a 4-mL screw-capped vial and stirred for 15 mins. Then **4a** (0.2 mmol, 23 mg) and $PhSiH_3$ (0.4 mmol, 43 mg) were added. The vial was sealed with a cap containing a PTFE septum. The reaction mixture was stirred at room temperature for 6 h and the resulting solution was concentrated in vacuum. The title compound was isolated (32 mg, 72% yield) as a colorless oil after chromatography on silica with PE. Rf=0.7. ^{1}H NMR (600 MHz, $CDCl_3$, 25 ºC) δ 7.64–7.57 (m, 2H), 7.46–7.40 (m, 1H), 7.41–7.35 (m, 2H), 7.33–7.27 (m, 4H), 7.23–7.15 (m, 1H), 6.36 (d, *J* = 15.8 Hz, 1H), 6.29 (dt, *J* = 15.6, 7.6 Hz, 1H), 4.39 (t, *J* = 3.7 Hz, 2H), 2.13–2.04 (m, 2H). ^{13}C NMR (151 MHz, $CDCl_3$, 25 ºC) δ 138.1, 135.4, 131.8, 130.2, 129.9, 128.6, 128.2, 126.8, 126.3, 125.8, 17.1. IR (ATR): ν 3067, 3022, 2959, 2885, 2139, 1640, 1595, 1494, 1427, 1252, 1155, 1118, 931, 875, 700 . HRMS (ESI$^+$): (m/z) calcd for $C_{15}H_{17}Si^+$ (M+H$^+$), 225.1094; found, 225.1086.

Benzyl(cinnamyl)(hexyl)silane (5v)

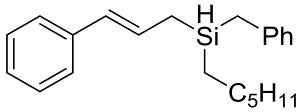

In an Ar-filled dry box, $Cu(OAc)_2$ (3.6 mg, 0.02 mmol), Xantphos (16 mg, 0.03 mmol) and THF(0.4 mL) were added to a 4-mL screw-capped vial and stirred for 15 mins. Then **4a** (0.2 mmol, 23 mg) and silane (0.4 mmol, 83 mg) were added. The vial was sealed with a cap containing a PTFE septum. The reaction mixture was stirred at 30 ºC for 12 h and the resulting solution was concentrated in vacuum. The title compound was isolated (34 mg, 53% yield) as a colorless oil after chromatography on silica with PE. Rf=0.6. ^{1}H NMR (600 MHz, $CDCl_3$, 25 ºC) δ 7.29–7.26 (m, 4H), 7.26–7.21 (m, 2H), 7.19–7.14 (m, 1H), 7.12–7.05 (m, 3H), 6.25 (d, *J* = 15.7 Hz, 1H), 6.16 (dt, *J* = 15.8, 8.0 Hz, 1H), 3.91–3.86 (m, 1H), 2.22 (d, *J* = 3.4 Hz, 2H), 1.74 (dd, *J* = 8.3, 3.1 Hz, 2H), 1.38–1.17 (m, 8H), 0.87 (t, *J* = 7.0 Hz, 3H), 0.68–0.59 (m, 2H). ^{13}C NMR (151 MHz, $CDCl_3$, 25 ºC) δ 139.8, 138.3, 129.3, 128.6, 128.6, 128.4, 127.0, 126.6, 125.7, 124.5, 32.9, 31.6, 24.4, 22.7, 21.2, 18.1, 14.3, 10.8. IR (ATR): ν 3067, 3015, 2922, 2847, 2120, 1466, 1397, 1155, 1114, 961, 797, 730, 697.

cinnamyl(methyl)(phenyl)silane (5w)

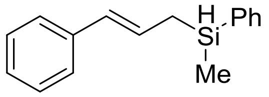

In an Ar-filled dry box, $Cu(OAc)_2$ (1.8 mg, 0.01 mmol), Xantphos (8.67 mg, 0.015 mmol) and THF(0.4 mL) were added to a 4-mL screw-capped vial and stirred for 15 mins. Then **4a** (0.2 mmol, 23 mg) and Bis(4-fluorophenyl)silane (0.4 mmol, 48.8 mg) were added. The vial was sealed with a cap containing a PTFE septum. The reaction mixture was stirred at 30 ºC for 3 h and the resulting

solution was concentrated in vacuum. The title compound was isolated (33 mg, 70% yield) as a colorless oil after chromatography on silica with PE/EA (100:1). Rf=0.6. ^{1}H NMR (600 MHz, $CDCl_3$, 25 ºC) δ 7.60–7.55 (m, 2H), 7.44–7.36 (m, 3H), 7.32–7.27 (m, 4H), 7.18 (m, 1H), 6.31 (d, J = 15.7 Hz, 1H), 6.25 (dt, J = 15.8, 7.9 Hz, 1H), 4.46–4.42 (m, 1H), 2.09–1.90 (m, 2H), 0.42 (d, J = 3.7 Hz, 3H). ^{13}C NMR (151 MHz, $CDCl_3$, 25 ºC) δ 138.3, 135.7, 134.5, 129.6, 129.6, 128.6, 128.1, 126.7, 126.6, 125.8, 20.6, -5.9. IR (ATR): ν 3067, 3022, 2955, 2124, 1494, 1427, 1252, 1118, 879, 827, 730, 700. HRMS (ESI$^+$): (m/z) calcd for $C_{16}H_{19}Si^+$ (M+H$^+$), 239.1251; found, 239.1256.

1.5 Synthetic transformations.

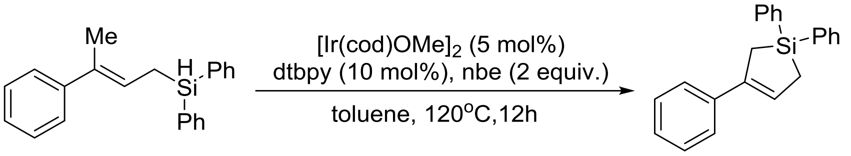

3a **6**, 60% yield

In a glovebox, to an oven-dried 10 mL Schlenk tube equipped with a magnetic stir bar was added $[Ir(COD)OMe]_2$ (6.64 mg, 0.01 mmol, 5 mol%), dtbpy (7.12 mg, 0.02 mmol), toluene (0.5 mL) and stirred for 15 min, Then nbe (0.4 mmol, 37.72 mg) and **3a** (62.8mg, 0.2 mmol) was added. The tube was then sealed, and the resulting mixture was stirred at 120 ºC in a heating block for 12h. after which the mixture was cooled to room temperature. The reaction mixture was filtered through a thin silica gel plug with CH_2Cl_2 (30 mL) as the eluent. The organic phase was concentrated under reduced pressure. The crude product was purified with column chromatography on silica gel (300~400 mesh) with PE/EA (50:1). as eluent to afford the title compound as a colorless oil (37 mg, 60% yield). ^{1}H NMR (600 MHz, $CDCl_3$, 25 ºC) δ 7.63–7.59 (m, 4H), 7.58–7.54 (m, 2H), 7.44–7.36 (m, 6H), 7.36–7.31 (m, 2H), 7.26–7.21 (m, 1H), 6.54–6.51 (m, 1H), 2.26 (d, J = 1.8 Hz, 2H), 2.10 (s, 2H). ^{13}C NMR (151 MHz, $CDCl_3$, 25 ºC) δ 141.9, 140.4, 135.7, 134.9, 129.8, 128.4, 128.2, 127.2, 127.0, 125.9, 18.6, 18.3. IR (ATR): ν 3049, 3022, 2914, 2873, 1643, 1490, 1427, 1397, 1114, 1073, 961, 849, 700. HRMS (APCI$^+$): (m/z) calcd for $C_{22}H_{21}Si^+$ (M+H$^+$), 313.1407; found, 331.1398.

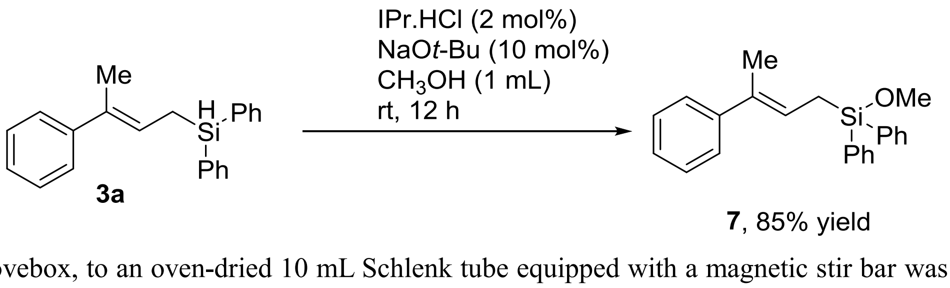

3a **7**, 85% yield

In a glovebox, to an oven-dried 10 mL Schlenk tube equipped with a magnetic stir bar was added IPr.HCl (0.85 mg, 0.02 mmol, 2 mol%), NaOtBu (1mg, 0.01 mmol), CH_3OH (1 mL), **3a** (31.4mg, 0.1 mmol), The tube was then sealed, and the resulting mixture was stirred at room temperature for 12h, after which the reaction mixture was filtered through a thin silica gel plug with CH_2Cl_2 (30 mL) as the eluent. The organic phase was concentrated under reduced pressure. The crude product was purified with column chromatography on silica gel (300~400 mesh) with PE/EA (50:1). as eluent to afford the title compound as a colorless oil (29 mg, 85% yield). ^{1}H NMR (600 MHz, $CDCl_3$, 25 ºC) δ 7.65–7.59 (m, 4H), 7.46–7.34 (m, 6H), 7.29–7.22 (m, 4H), 7.19–7.14 (m, 1H), 5.88 (td, J = 8.5, 1.4 Hz, 1H), 3.57 (s, 3H), 2.29 (d, J = 8.5 Hz, 2H), 1.76 (s, 3H). ^{13}C NMR (151 MHz, $CDCl_3$, 25

°C) δ 144.4, 134.9, 134.4, 134.3, 130.2, 128.2, 128.0, 126.3, 125.6, 122.1, 51.8, 17.6, 15.8. IR (ATR): 3049, 3022, 2937, 2832,1889, 1636, 1595, 1490, 1427, 1185, 1114, 1080, 1028, 801, 723, 700. HRMS (APCI$^+$): (m/z) calcd for $C_{23}H_{25}OSi^+$ (M+H$^+$), 345.1669; found, 345.1664.

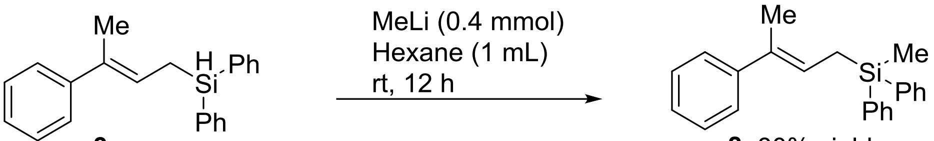

3a **8**, 80% yield

Under N_2, to the solution of **3a** (0.2 mmol, in 1 mL hexane) was added MeLi (1.6 M, 0.25 mL), then stirred at room temperature for 3h. The reaction mixture was quenched with NH_4Cl (aq), extract with dichloromethane (15 mL) for three times. The organic phase was concentrated and purified with column chromatography on silica gel (300~400 mesh) with PE/EA (100:1). as eluent to afford the title compound as a colorless oil (52 mg, 80% yield). ^{1}H NMR (600 MHz, $CDCl_3$, 25 °C) δ 7.63–7.53 (m, 4H), 7.46–7.35 (m, 6H), 7.31 (d, J = 4.7 Hz, 4H), 7.24–7.18 (m, 1H), 5.91 (td, J = 8.6, 1.4 Hz, 1H), 2.25 (d, J = 8.7 Hz, 2H), 1.86 (s, 3H), 0.63 (s, 3H). ^{13}C NMR (151 MHz, $CDCl_3$, 25 °C) δ 144.4, 136.8, 134.7, 133.6, 129.5, 128.2, 127.9, 126.3, 125.6, 123.6, 18.1, 15.9, –4.3. IR (ATR): ν 3049, 3022, 2955, 2922, 1882, 1595, 1490, 1427, 1252, 1110, 1058, 924, 875, 820, 730, 697. HRMS (APCI$^+$): (m/z) calcd for $C_{23}H_{25}Si^+$ (M+H$^+$), 329.1720; found, 329.1721.

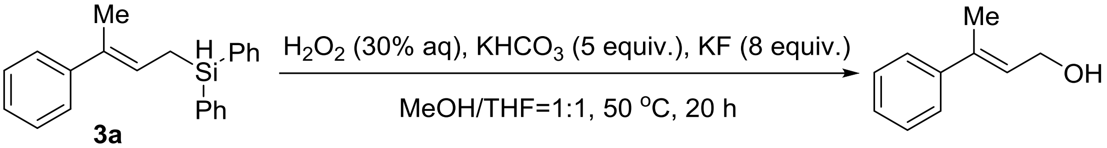

3a

9, 91% yield

To the solution of A (0.5 mmol, in 6 mL MeOH/THF=1:1) was added KF (4 mmol, 232 mg), $KHCO_3$(2.5 mmol, 250 mg), H_2O_2(1.2 mL) and then stirred at 50°C for 20 h. after which the reaction mixture was filtered through a thin silica gel plug with CH_2Cl_2 (30 mL) as the eluent. The organic phase was concentrated under reduced pressure. The crude product was purified with column chromatography on silica gel (300~400 mesh) with PE/EA (5:1). as eluent to afford the title compound as a colorless oil (67 mg, 91% yield). ^{1}H NMR (600 MHz, $CDCl_3$, 25 °C) δ 7.41–7.36 (m, 2H), 7.33–7.28 (m, 2H), 7.27–7.21 (m, 1H), 5.96 (td, J = 6.6, 1.4 Hz, 1H), 4.34 (d, J = 6.7 Hz, 2H), 2.05 (s, 4H). The spectroscopic data for this product matched the literature data[9].

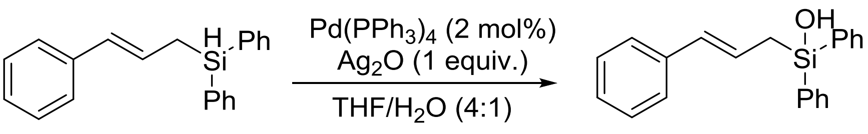

5a **10**, 90% yield

In a glovebox, to an oven-dried 10 mL Schlenk tube equipped with a magnetic stir bar was added $Pd(PPh_3)_4$ (4.62 mg, 0.004 mmol, 2 mol%), Ag_2O (46.2 mg, 0.2 mmol), THF (0.8 mL), H_2O (0.2 mL), and 5a (60 mg, 0.2 mmol) The tube was then sealed, and the resulting mixture was stirred at 80°C for 12h, after which the reaction mixture was filtered through a thin silica gel plug with CH_2Cl_2 (30 mL) as the eluent. The organic phase was concentrated under reduced pressure. The crude product was purified with column chromatography on silica gel (300~400 mesh) with PE/EA (25:1).

as eluent to afford the title compound as a colorless oil (57 mg, 90% yield). ^{1}H NMR (600 MHz, $CDCl_3$, 25 °C) δ 7.55–7.47 (m, 4H), 7.39–7.33 (m, 2H), 7.29–7.22 (m, 4H), 7.22–7.16 (m, 2H), 7.15–7.09 (m, 1H), 7.04 (d, J = 7.3 Hz, 2H), 6.14–6.04 (m, 2H), 2.22 (d, J = 6.2 Hz, 2H). ^{13}C NMR (151 MHz, $CDCl_3$, 25 °C) δ 138.2, 135.8, 134.6, 130.6, 129.9, 128.5, 127.9, 126.5, 125.8, 125.3, 23.0. IR (ATR): ν 3049, 3022, 2922, 2877, 1640, 1595, 1490, 1427, 1114, 1073, 961, 700.

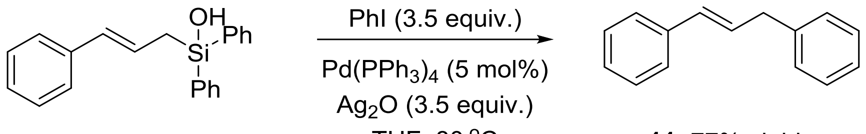

10 **11**, 77% yield

In a glovebox, to an oven-dried 10 mL Schlenk tube equipped with a magnetic stir bar was added $Pd(PPh_3)_4$ (5.8 mg, 0.005 mmol, 2 mol%), Ag_2O (69 mg, 0.3 mmol), THF (0.2 mL), PhI (71.4 mg, 0.35 mmol) and **10** (31.6 mg, 0.1 mmol) The tube was then sealed, and the resulting mixture was stirred at 80°C for 12h, after which the reaction mixture was filtered through a thin silica gel plug with CH_2Cl_2 (30 mL) as the eluent. The organic phase was concentrated under reduced pressure. The crude product was purified with column chromatography on silica gel (300~400 mesh) with PE/EA (100:1). as eluent to afford the title compound as a colorless oil (15 mg, 77% yield). ^{1}H NMR (600 MHz, $CDCl_3$, 25 °C) δ 7.37–7.33 (m, 2H), 7.32–7.26 (m, 4H), 7.25–7.21 (m, 2H), 7.21–7.16 (m, 2H), 6.44 (d, J = 15.7 Hz, 1H), 6.35 (dt, J = 15.7, 6.8 Hz, 1H), 3.54 (d, J = 5.4 Hz, 2H). The spectroscopic data for this product matched the literature data[11].

1.6 Mechanism study

a) A deuterium-labeling reaction indicate that the hydrogen comes from the silane.

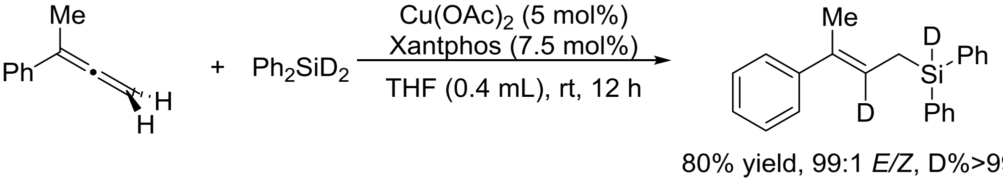

80% yield, 99:1 *E/Z*, D%>99%

In an Ar-filled dry box, $Cu(OAc)_2$ (1.8 mg, 0.01 mmol), Xantphos (8.67 mg, 0.015 mmol) and THF(0.4 mL) were added to a 4-mL screw-capped vial and stirred for 15 mins. Then 1,1-disubsitituted allenes (0.2 mmol) and Ph_2SiD_2 (1.2 eq, 0.24 mmol) were added. The vial was sealed with a cap containing a PTFE septum. The reaction mixture was stirred at room temperature for 12 h and the resulting solution was concentrated in vacuum. The organic phase was concentrated and purified with column chromatography on silica gel (300~400 mesh) with PE/EA (100:1). as eluent to afford the title compound as a colorless oil (51 mg, 80% yield). ^{1}H NMR (600 MHz, $CDCl_3$, 25 °C) δ 7.65–7.58 (m, 4H), 7.46–7.36 (m, 6H), 7.31–7.27 (m, 4H), 7.24–7.17 (m, 1H), 2.28 (s, 2H), 1.86 (s, 3H). ^{2}H NMR (92 MHz,) δ 6.07 (s, 1H), 5.10 (s, 1H).

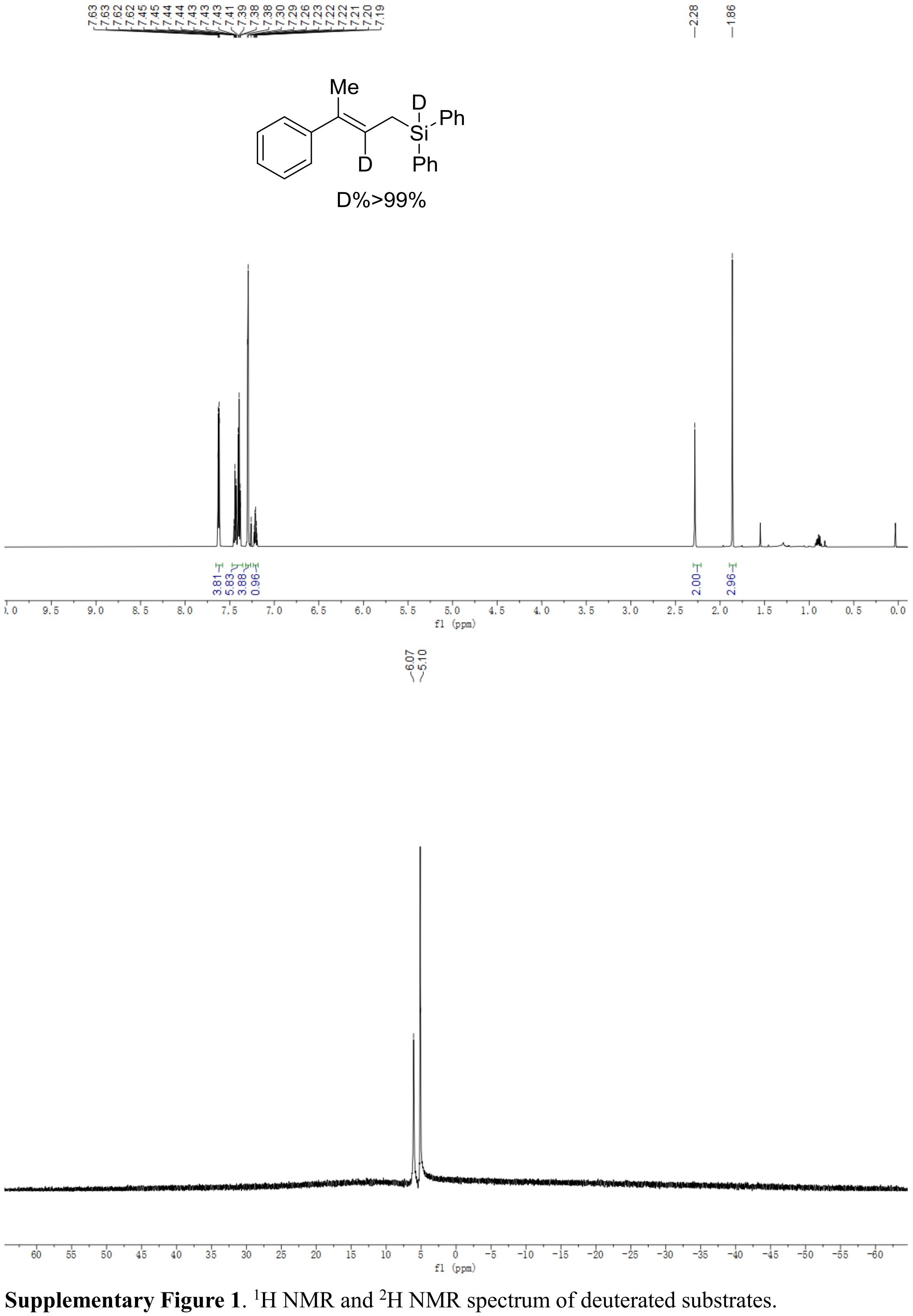

Supplementary Figure 1. ^{1}H NMR and ^{2}H NMR spectrum of deuterated substrates.

b) KIE experiment

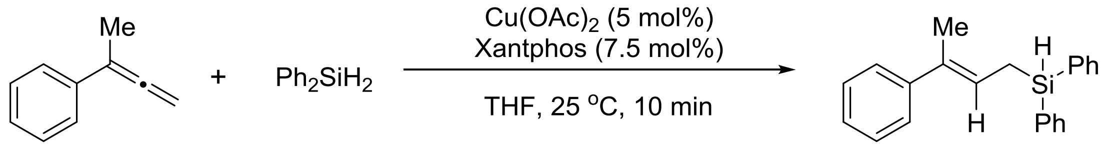

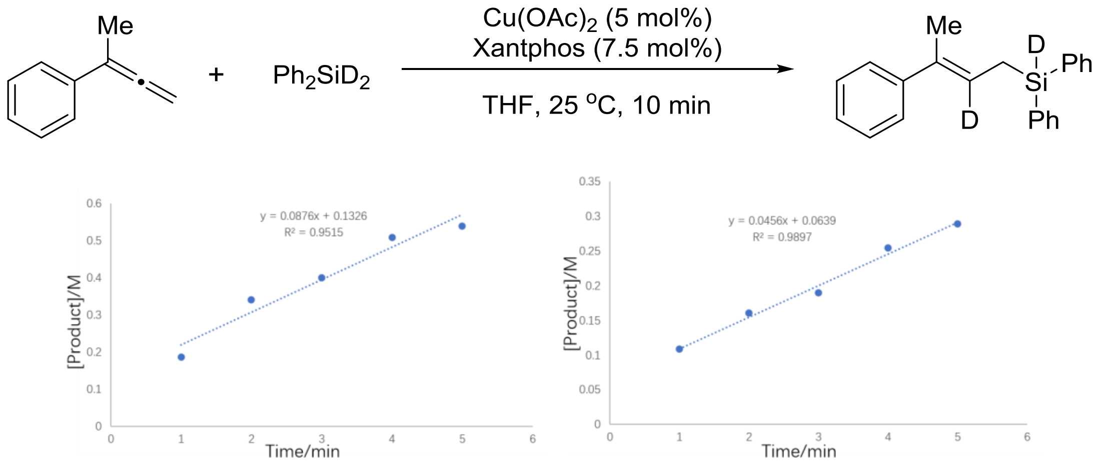

Supplementary Figure 2. KIE experiment.

In an Ar-filled dry box, $Cu(OAc)_2$ (1.8 mg, 0.01 mmol), Xantphos (8.67 mg, 0.015 mmol) and THF(0.4 mL) were added to a 4-mL screw-capped vial and stirred for 15 mins. Then dodecane (10 uL, internal standard), 1,1-disubsitituted allenes (26 mg, 0.2 mmol) and Ph_2SiH_2 (1.2 eq, 0.24 mmol) or Ph_2SiD_2 (1.2 eq, 0.24 mmol) were added. The reaction mixture was stirred at 25°C and the resulting solution was concentrated in vacuum. The reactions were monitored by GC. The initial rate of two reactions showed that the KIE was 1.92.

c) Isomerization Studies

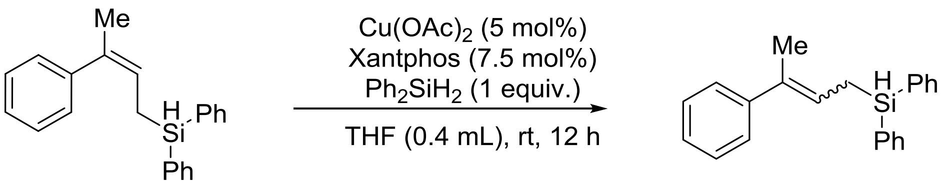

Z-**3a**, 99:1 *Z/E* 99:1 *Z/E*

***Z*-3a** was synthesized according to the literature[7]. ^{1}H NMR (600 MHz, $CDCl_3$, 25 °C) δ 7.48 (d, J = 8.0 Hz, 4H), 7.42–7.36 (m, 2H), 7.36–7.30 (m, 4H), 7.30–7.24 (m, 2H), 7.23–7.16 (m, 1H), 7.03 (d, J = 6.8 Hz, 2H), 5.54 (t, J = 8.4 Hz, 1H), 4.84 (t, J = 3.5 Hz, 1H), 2.04 (dd, J = 8.2, 3.7 Hz, 2H), 1.97 (s, 3H).

In an Ar-filled dry box, $Cu(OAc)_2$ (1.8 mg, 0.01 mmol), Xantphos (8.67 mg, 0.015 mmol) and THF(0.4 mL) were added to a 4-mL screw-capped vial and stirred for 15 mins. Then ***Z*-3a** (0.2 mmol) and Ph_2SiH_2 (1 eq, 0.2 mmol) were added. The vial was sealed with a cap containing a PTFE septum. The reaction mixture was stirred at room temperature for 12 h and the resulting solution was concentrated in vacuum. The yield (95% recovery) and *Z*/*E* ratio (*Z*/*E* = 99:1) was determined by HNMR analysis of unpurified reaction mixtures, 1,2-dibromoethane is used as the internal label, and no isomerization was found.

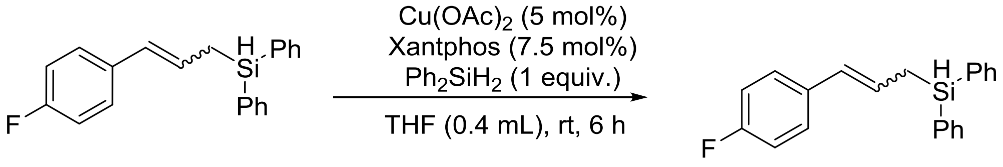

5**h'**, 91:9 *Z/E* 91:9 *Z/E*

5h' was synthesized according to this procedure: in an Ar-filled dry box, $Cu(OAc)_2$ (1.8 mg, 0.01 mmol), DPPM (3.84 mg, 0.01 mmol) and THF(0.4 mL) were added to a 4-mL screw-capped vial and stirred for 15 mins. Then allene (0.2 mmol) and Ph_2SiH_2 (1.1 equiv., 0.22 mmol) were added. The vial was sealed with a cap containing a PTFE septum. The reaction mixture was stirred at room temperature for 12 h and the resulting solution was concentrated in vacuum. The organic phase was concentrated and purified with column chromatography on silica gel (300~400 mesh) with PE/EA (100:1). as eluent to afford the title compound as a colorless oil (60 mg, 95% yield, *E*/*Z* = 91:9). The test to determine possible isomerization was performed with the Z/E mixture: in an Ar-filled dry box, $Cu(OAc)_2$ (1.8 mg, 0.01 mmol), Xantphos (8.67 mg, 0.015 mmol) and THF(0.4 mL) were added to a 4-mL screw-capped vial and stirred for 15 mins. Then **5h'** (0.2 mmol) and Ph_2SiH_2 (1 eq, 0.2 mmol) were added. The vial was sealed with a cap containing a PTFE septum. The reaction mixture was stirred at room temperature for 12 h .The *Z*/*E* ratio (*E*/*Z* = 91:9) was determined by GC-MS analysis of unpurified reaction mixtures, indicating that there is no isomerization of the product.

d) Computational Details

Computational methods

All DFT calculations were carried out using the Gaussian 16 series of programs[12]. Geometries of intermediates and transition states were optimized using dispersion-corrected B3LYP-D3(BJ) functional[13] with a mixed basis set of LANL2DZ for Cu and 6-31G(d) for other atoms in the gas phase. Vibrational frequency calculations were performed for all stationary points to confirm if each optimized structure is a local minimum or a transition state structure. All optimized transition state structures have only one imaginary (negative) frequency, and all minima (reactants, products, and intermediates) have no imaginary frequencies. The M06-L functional[14] with a mixed basis set of SDD for Cu and 6-311+G(d,p) for other atoms was used for single-point energy calculations in solution. Solvation energy corrections were calculated in tetrahydrofuran as solvent with the SMD continuum solvation model[15] based on the gas-phase optimized geometries. The 3D images of structures were prepared using CYLView[16]. The independent gradient model (IGM)[17] was calculated at the M06-L/6-311+G(d,p) level.

2. Supplementary Figures

2.1 Spectroscopic Data

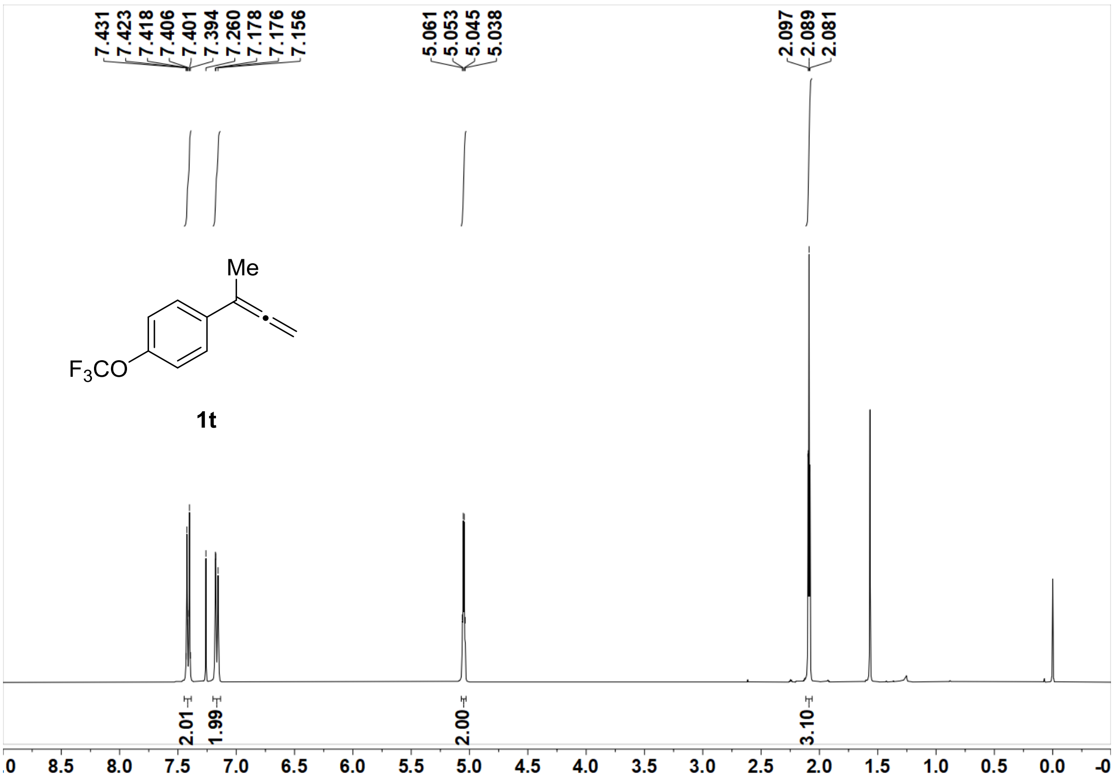

Supplementary Figure 3. ^{1}H NMR (400 MHz, $CDCl_3$, 25 °C) spectra of **1t**.

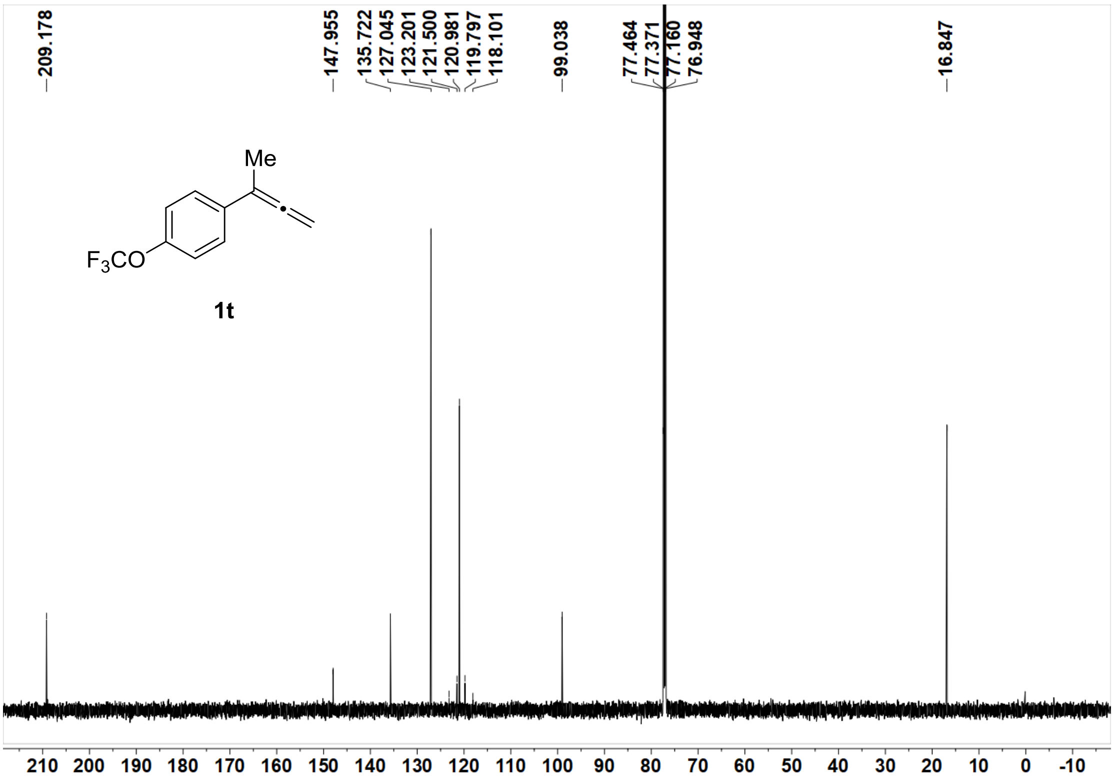

Supplementary Figure 4. ^{13}C NMR (151 MHz, $CDCl_3$, 25 °C) spectra of **1t**.

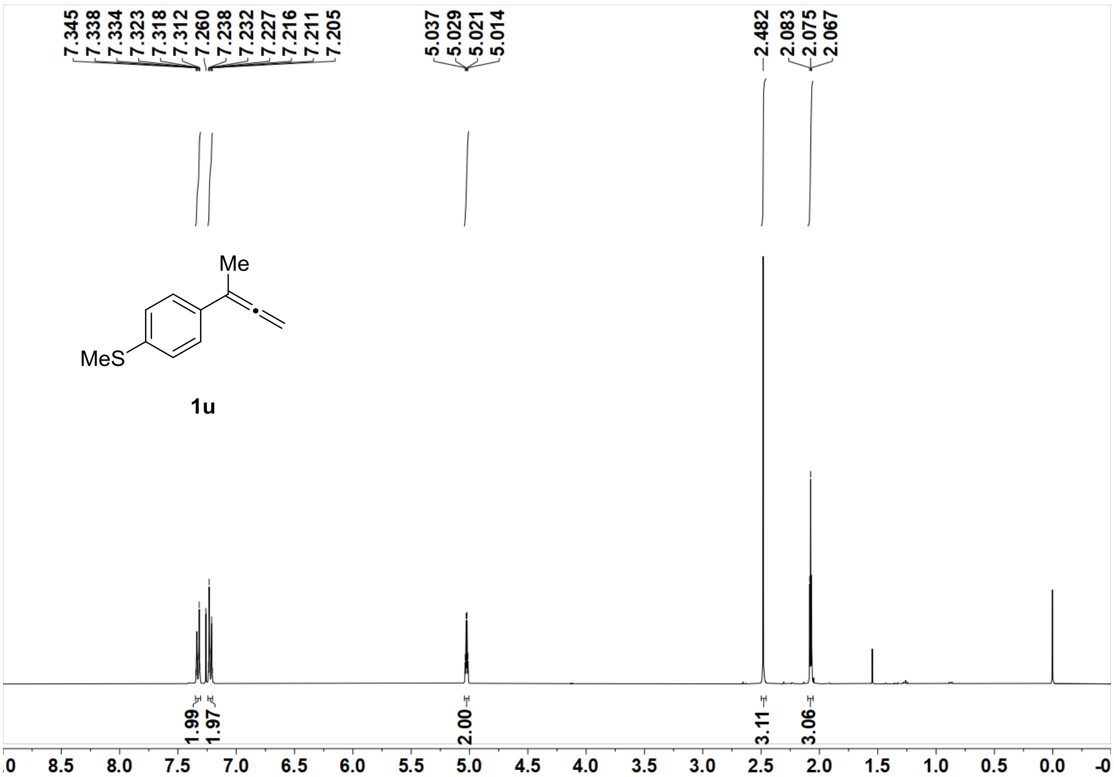

Supplementary Figure 5. ^{1}H NMR (400 MHz, $CDCl_3$, 25 ºC) spectra of **1u**.

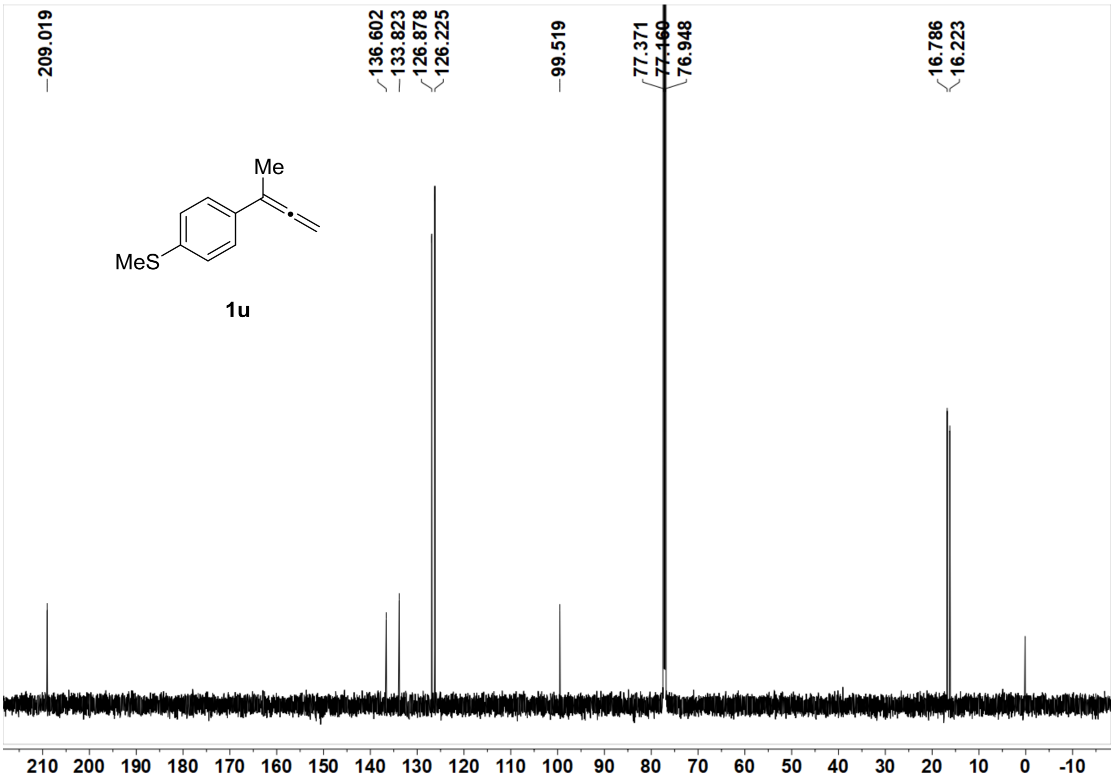

Supplementary Figure 6. ^{13}C NMR (151 MHz, $CDCl_3$, 25 ºC) spectra of **1u**.

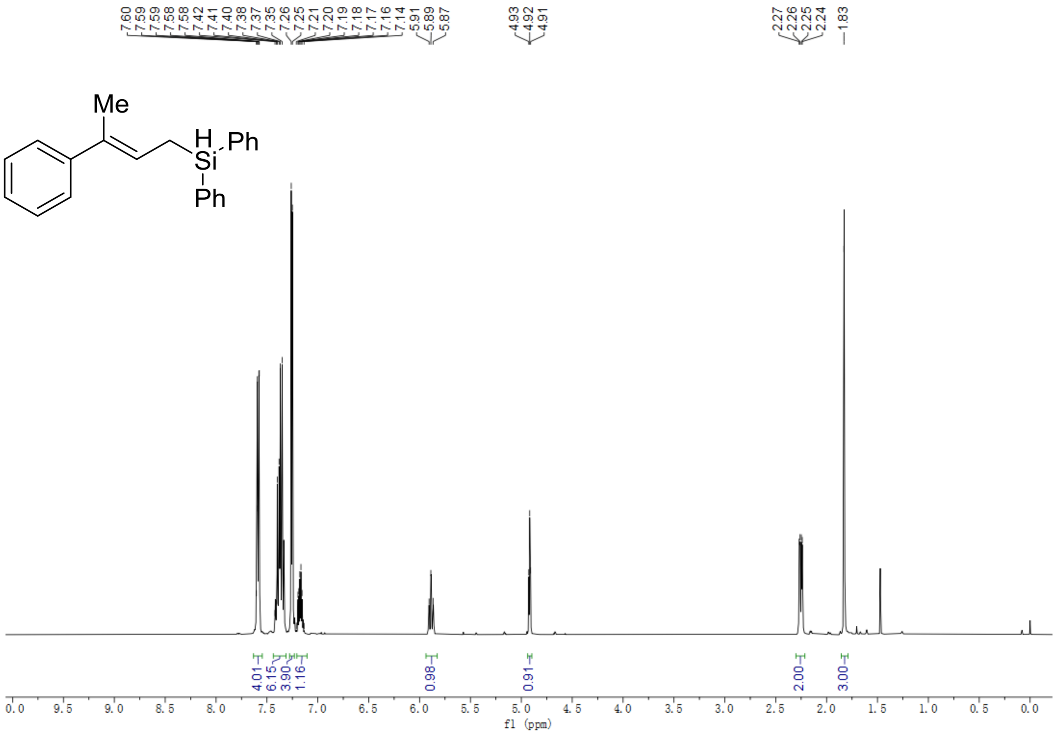

Supplementary Figure 7. ^{1}H NMR (400 MHz, $CDCl_3$, 25 ºC) spectra of **3a**.

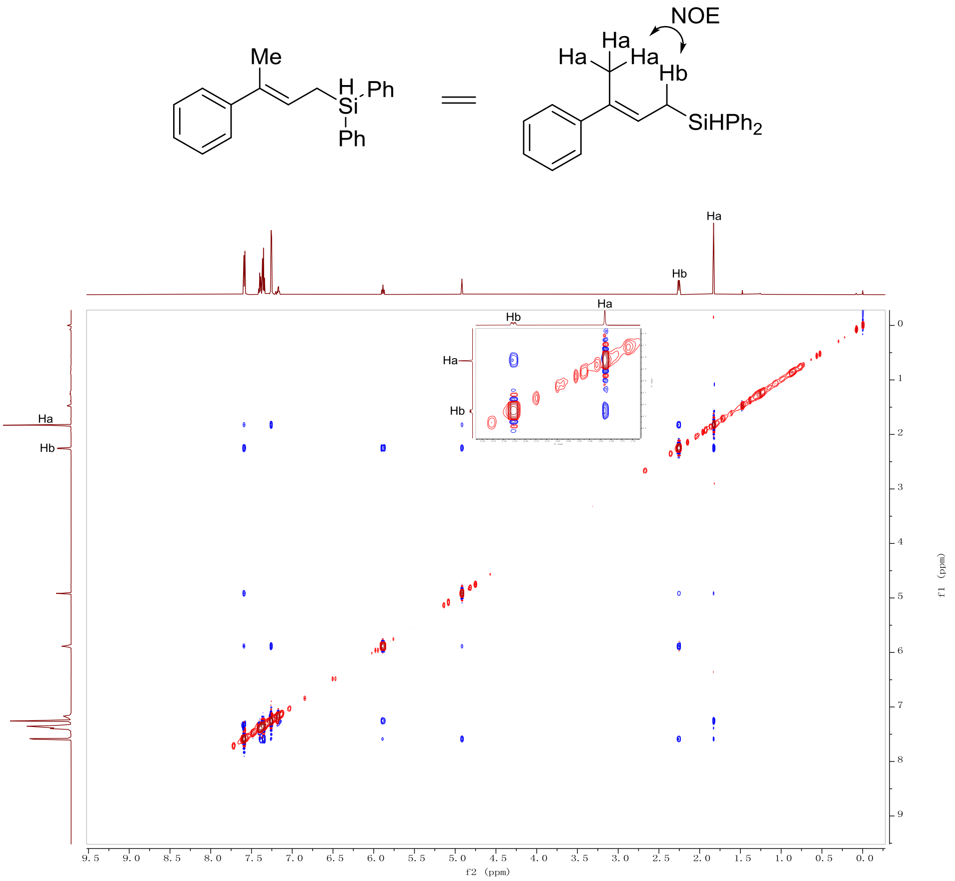

Supplementary Figure 8. ^{1}H-^{1}H NOESY spectroscopy of **3a**

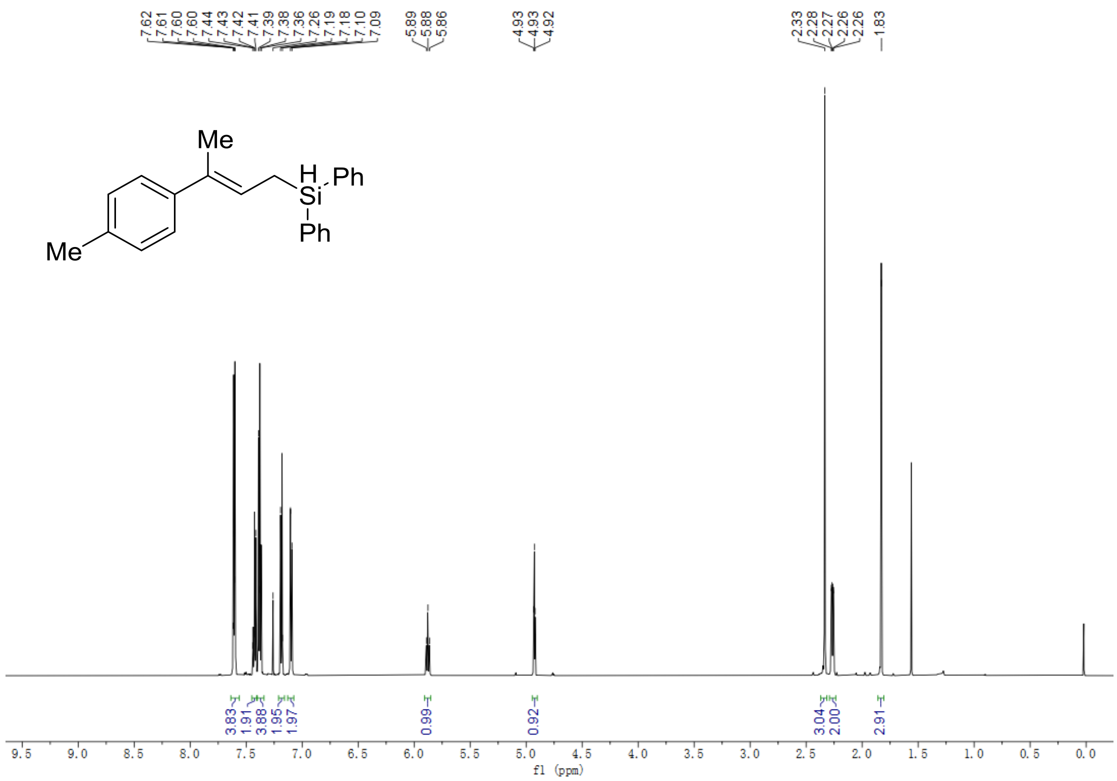

Supplementary Figure 9. ^{1}H NMR (600 MHz, $CDCl_3$, 25 ºC) spectra of **3b**.

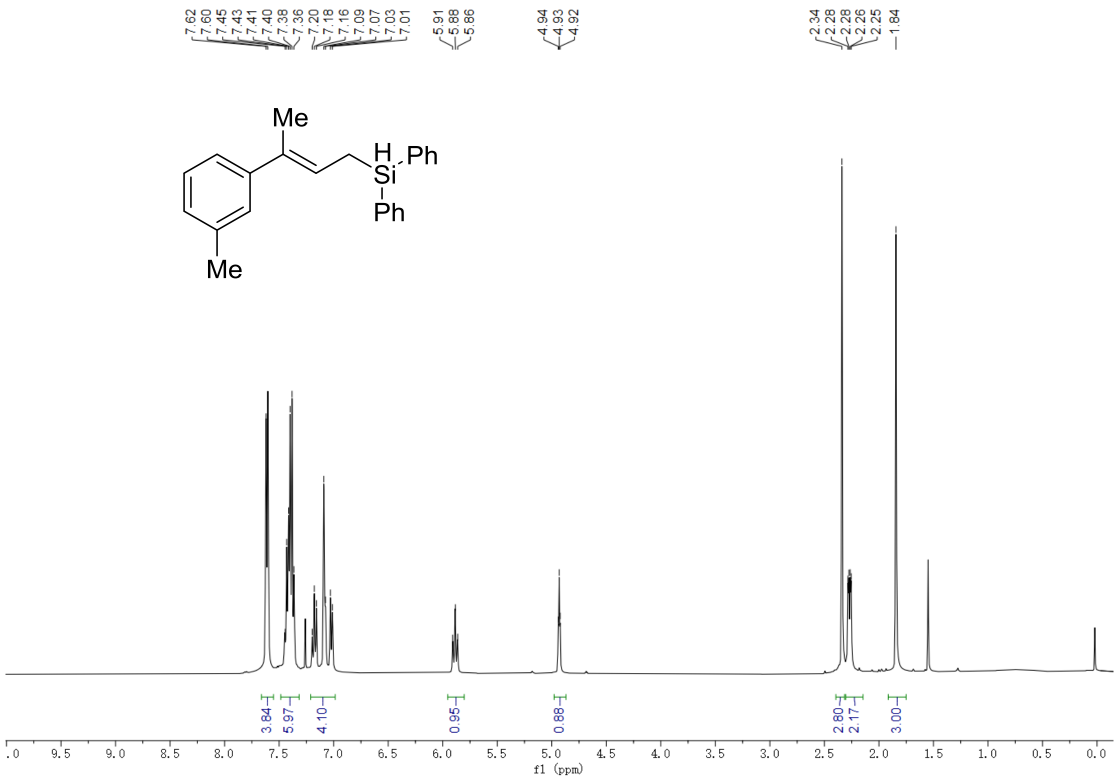

Supplementary Figure 10. ^{1}H NMR (400 MHz, $CDCl_3$, 25 ºC) spectra of **3c**.

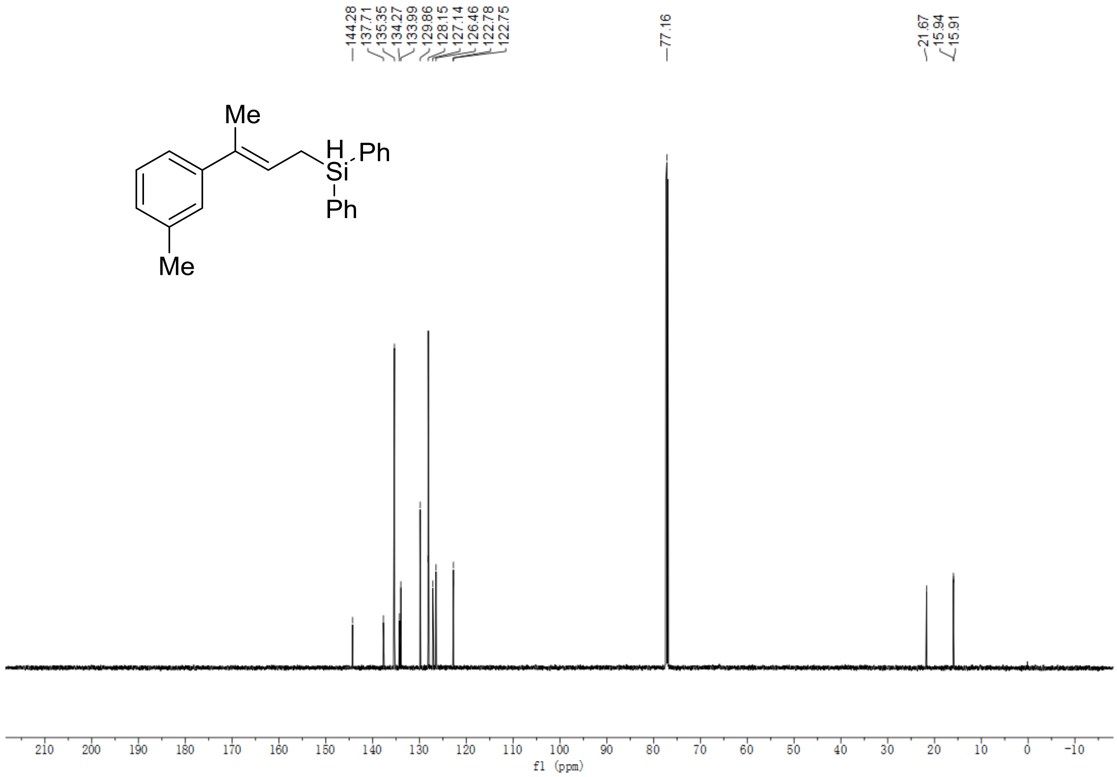

Supplementary Figure 11. ^{13}C NMR (151 MHz, $CDCl_3$, 25 ºC) spectra of **3c**.

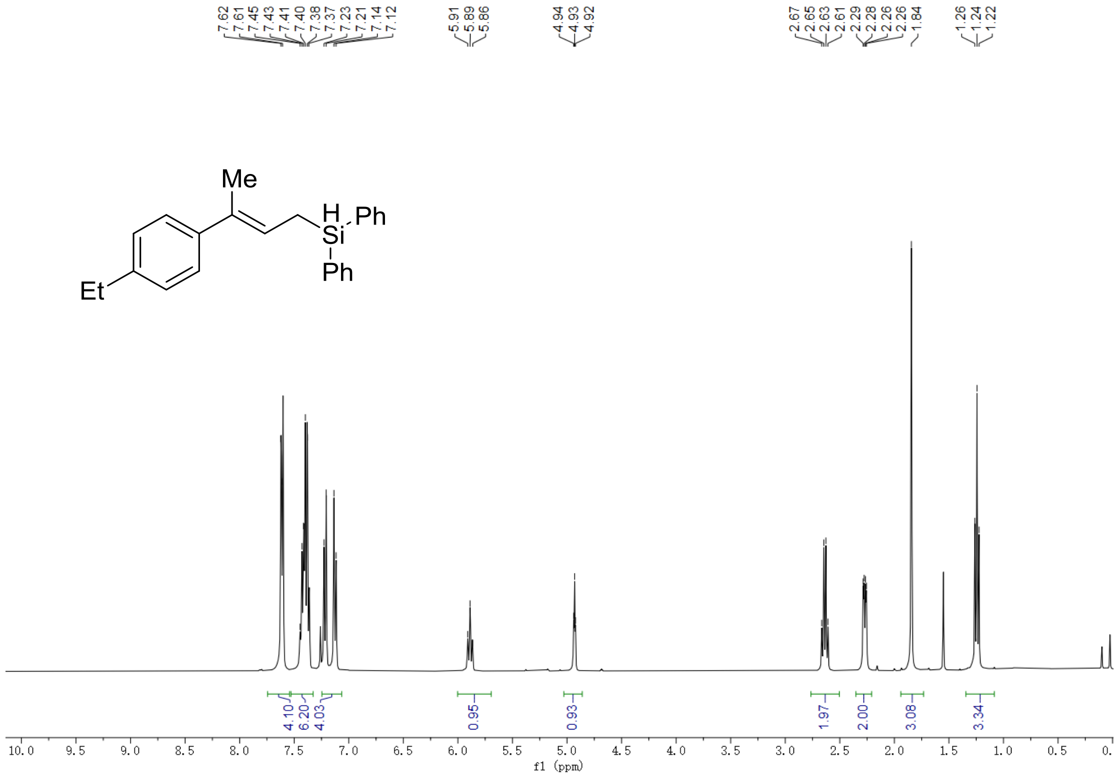

Supplementary Figure 12. ^{1}H NMR (400 MHz, $CDCl_3$, 25 ºC) spectra of **3d**.

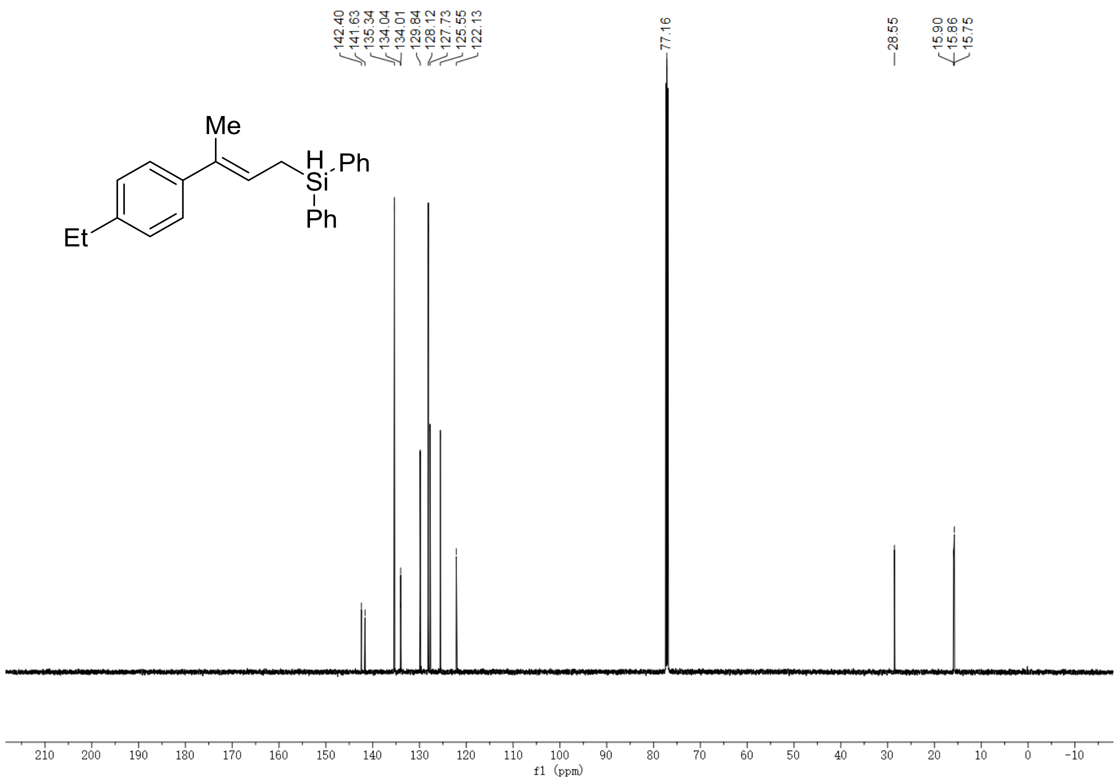

Supplementary Figure 13. ^{13}C NMR (151 MHz, $CDCl_3$, 25 ºC) spectra of **3d**.

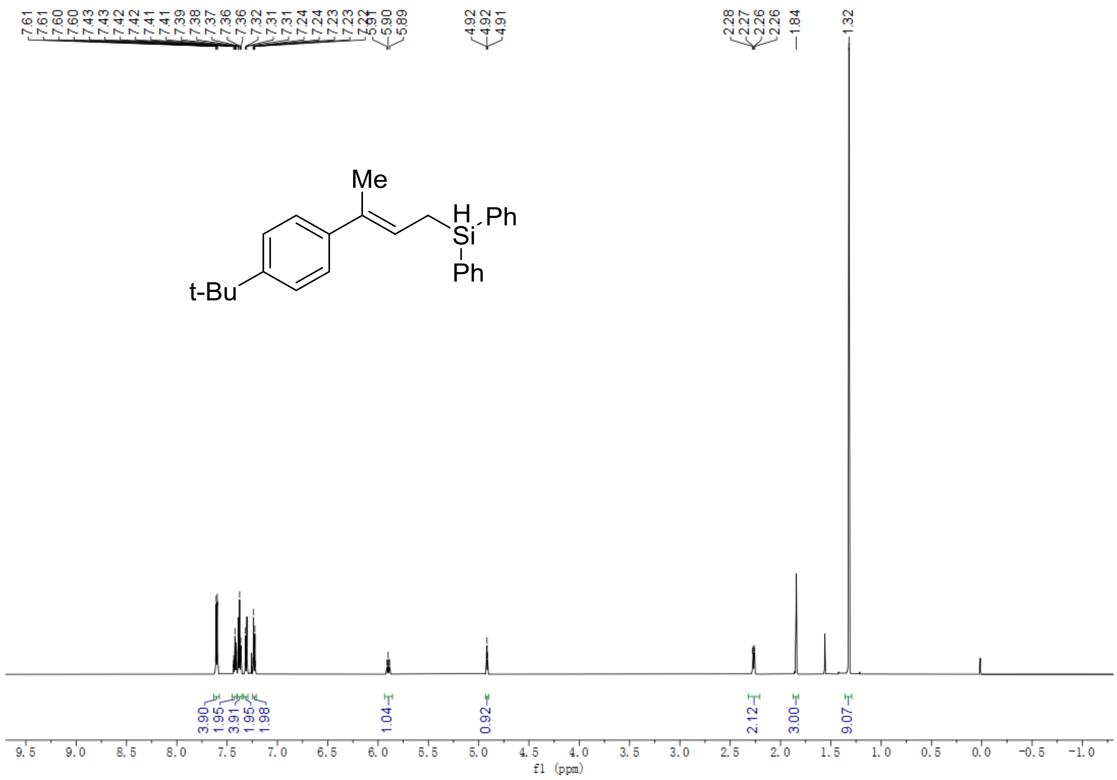

Supplementary Figure 14. ^{1}H NMR (600 MHz, $CDCl_3$, 25 ºC) spectra of **3e**.

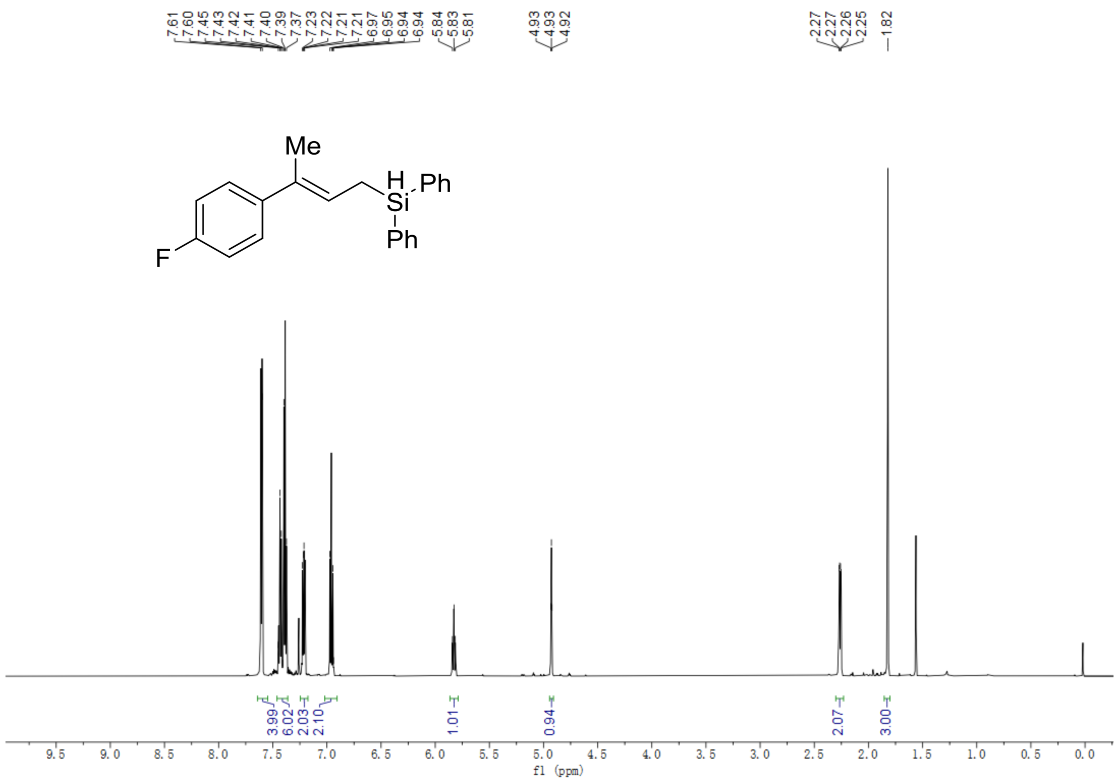

Supplementary Figure 15. ^{1}H NMR (600 MHz, $CDCl_3$, 25 ºC) of **3f**.

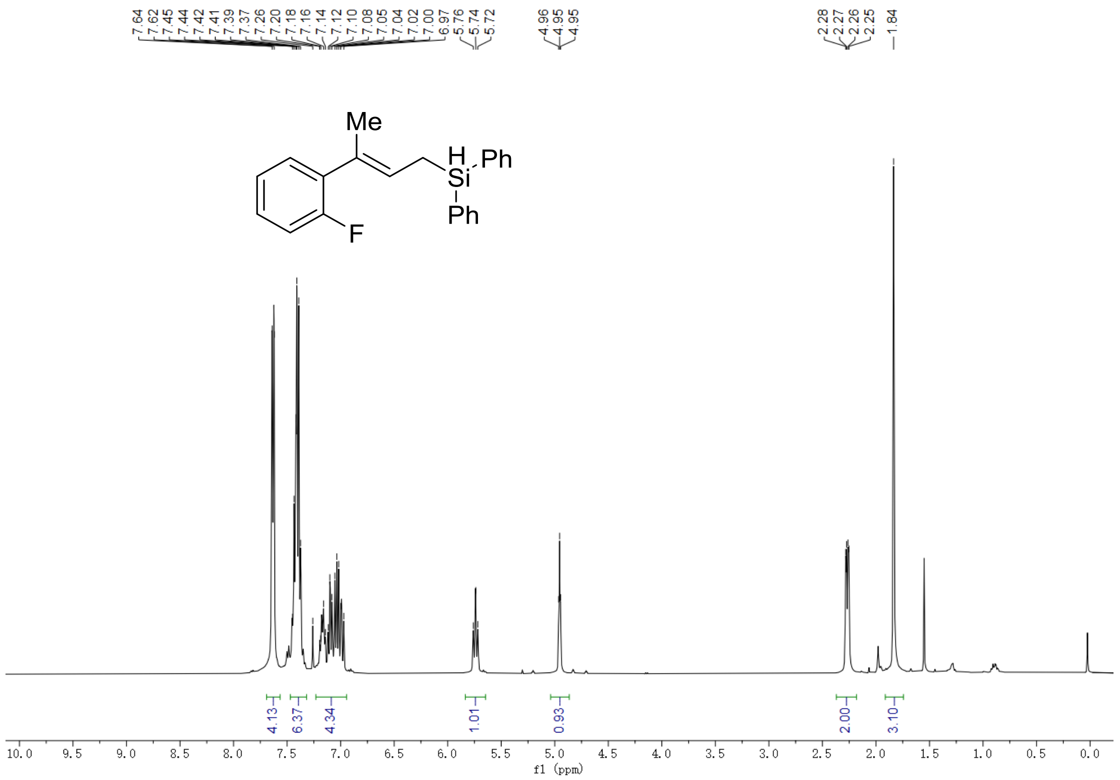

Supplementary Figure 16. ^{1}H NMR (400 MHz, $CDCl_3$, 25 ºC) spectra of **3g**.

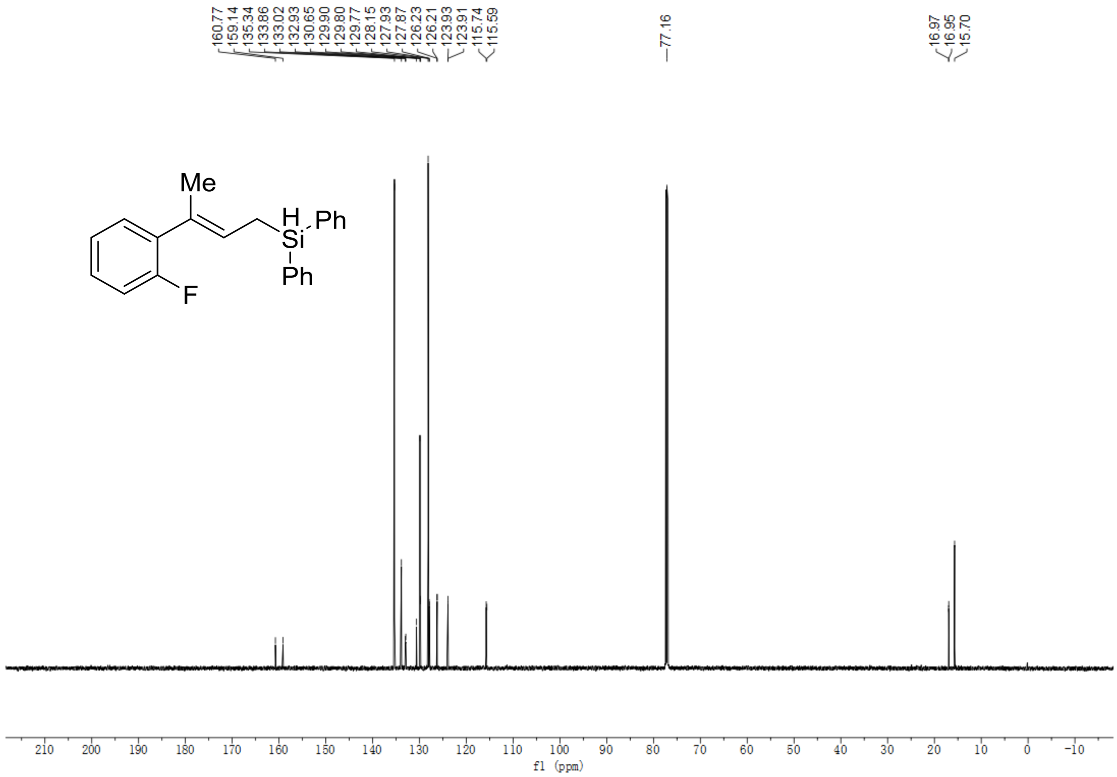

Supplementary Figure 17. ^{13}C NMR (151 MHz, $CDCl_3$, 25 ºC) spectra of **3g**.

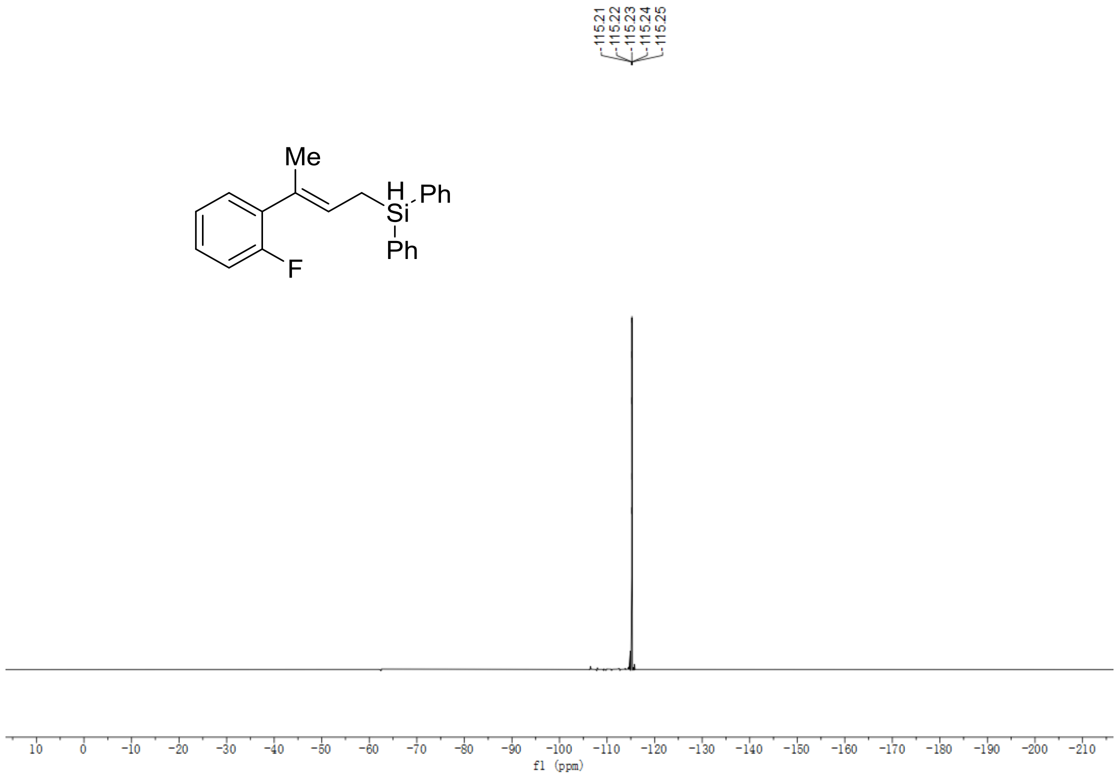

Supplementary Figure 18. ^{19}F NMR (565 MHz, $CDCl_3$, 25 ºC) spectra of **3g**.

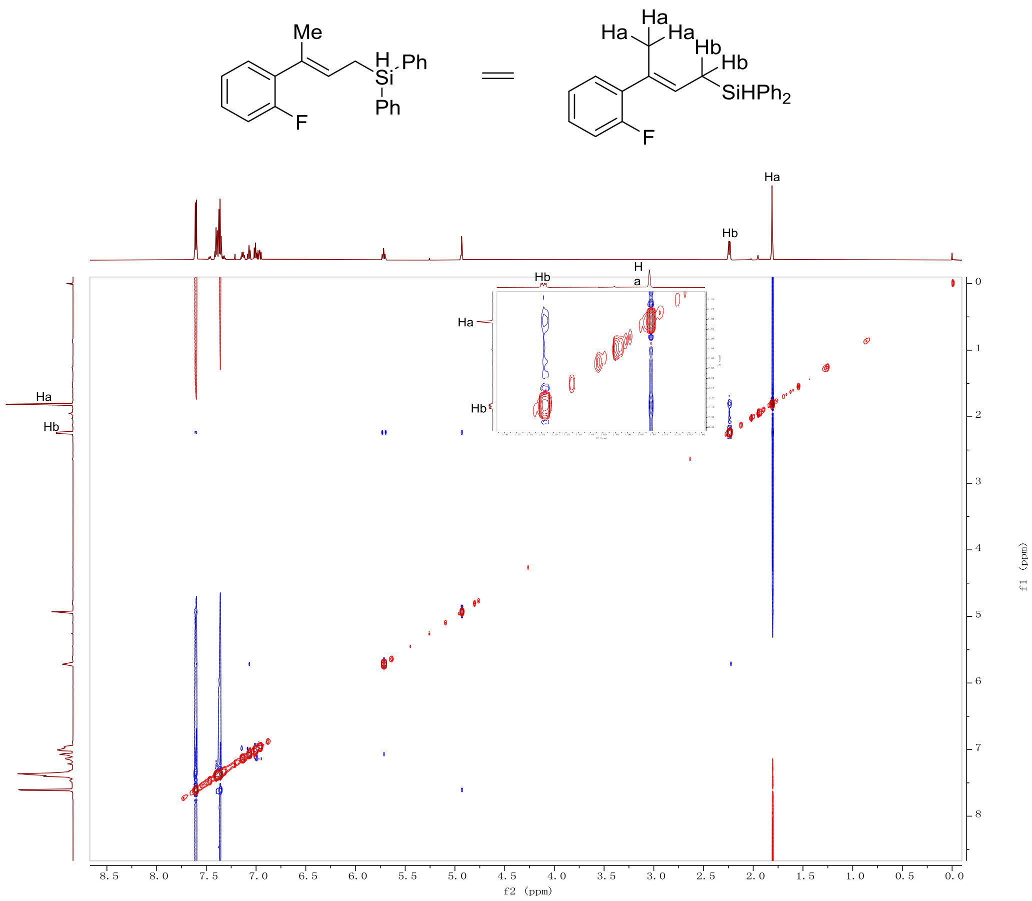

Supplementary Figure 19. ^{1}H-^{1}H NOESY spectra of **3g**.

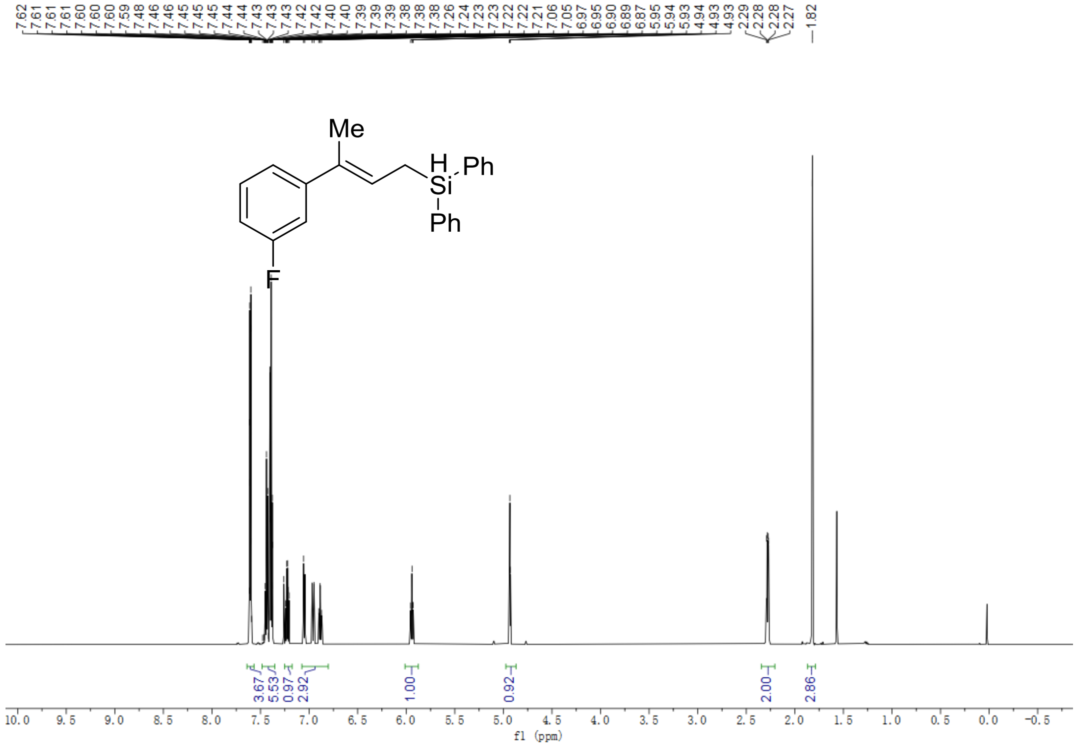

Supplementary Figure 20. ^{1}H NMR (600 MHz, $CDCl_3$, 25 ºC) spectra of **3h**.

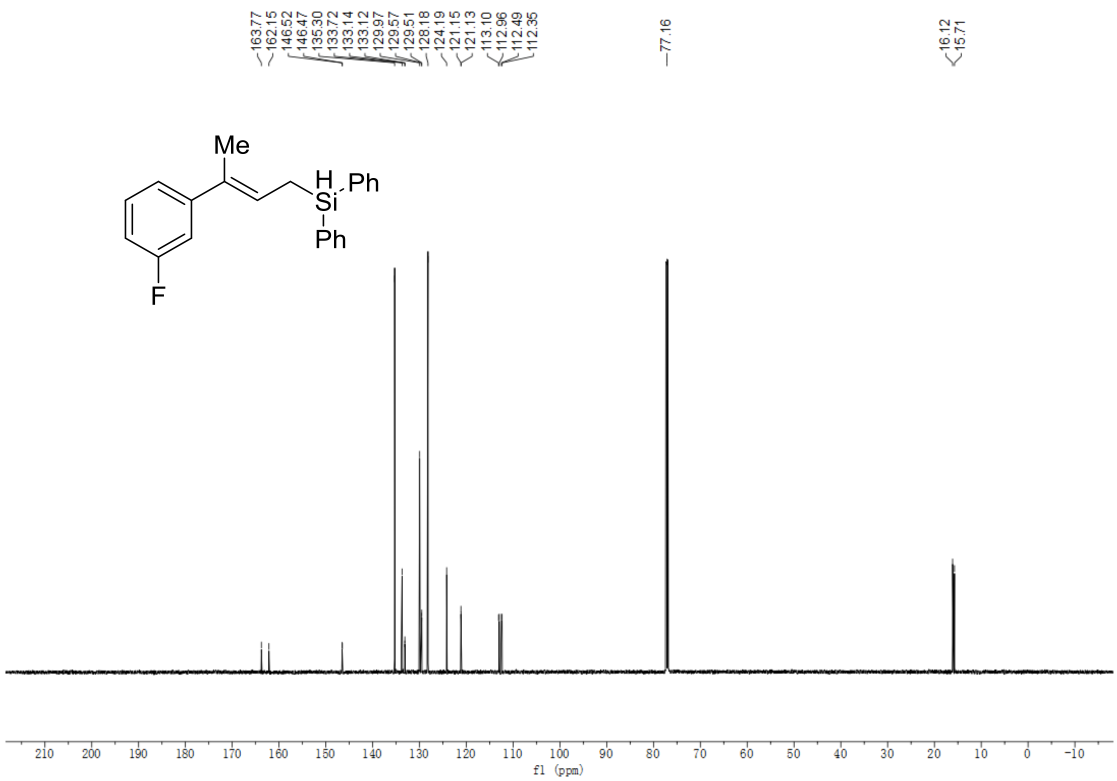

Supplementary Figure 21. ^{13}C NMR (151 MHz, $CDCl_3$, 25 ºC) spectra of **3h**.

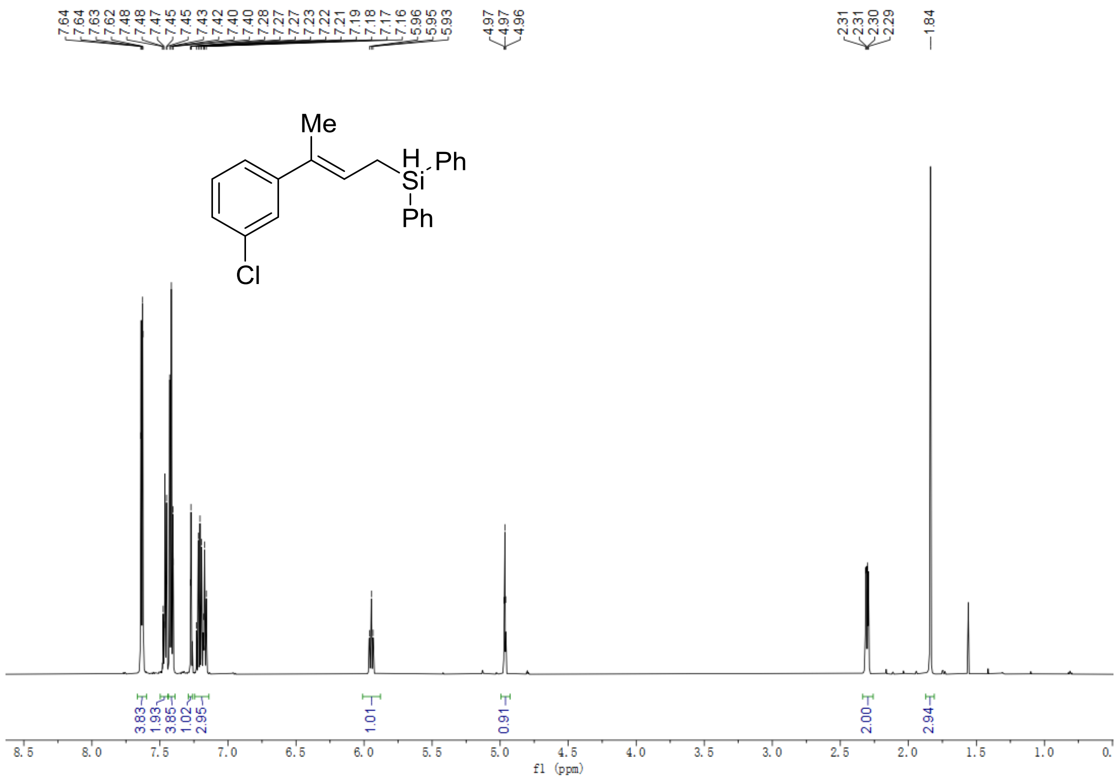

Supplementary Figure 22. ^{1}H NMR (600 MHz, $CDCl_3$, 25 ºC) of **3i**.

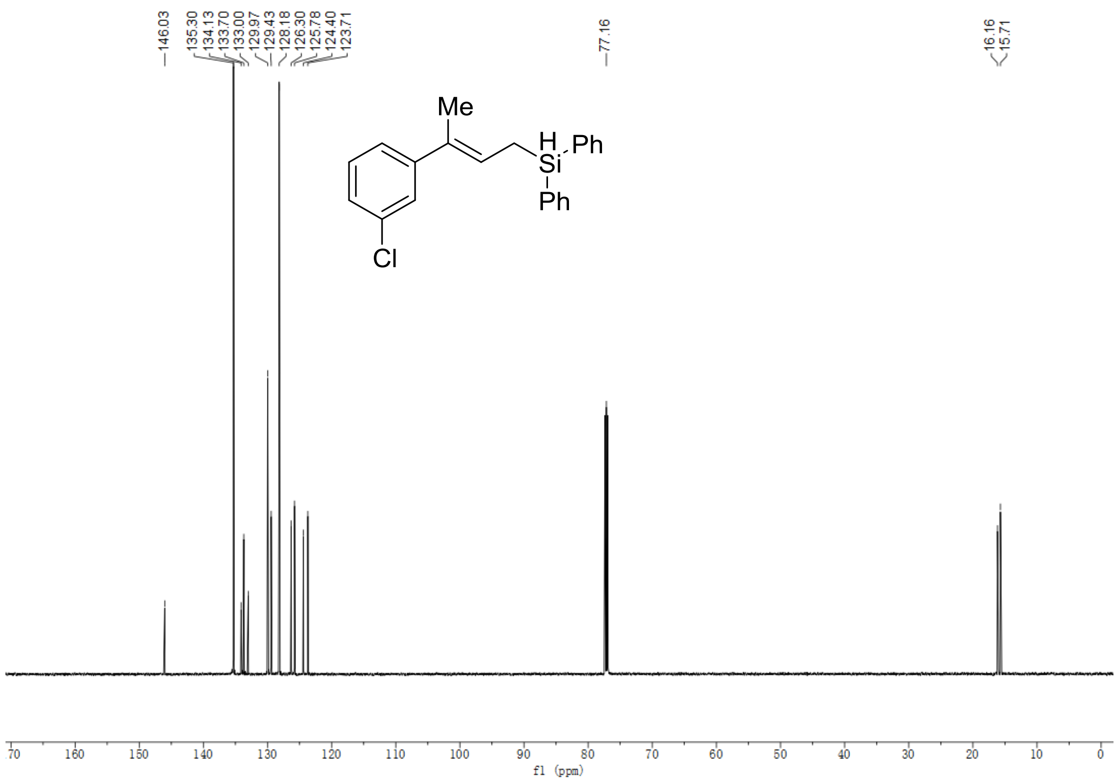

Supplementary Figure 23. ^{13}C NMR (151 MHz, $CDCl_3$, 25 ºC) of **3i**.

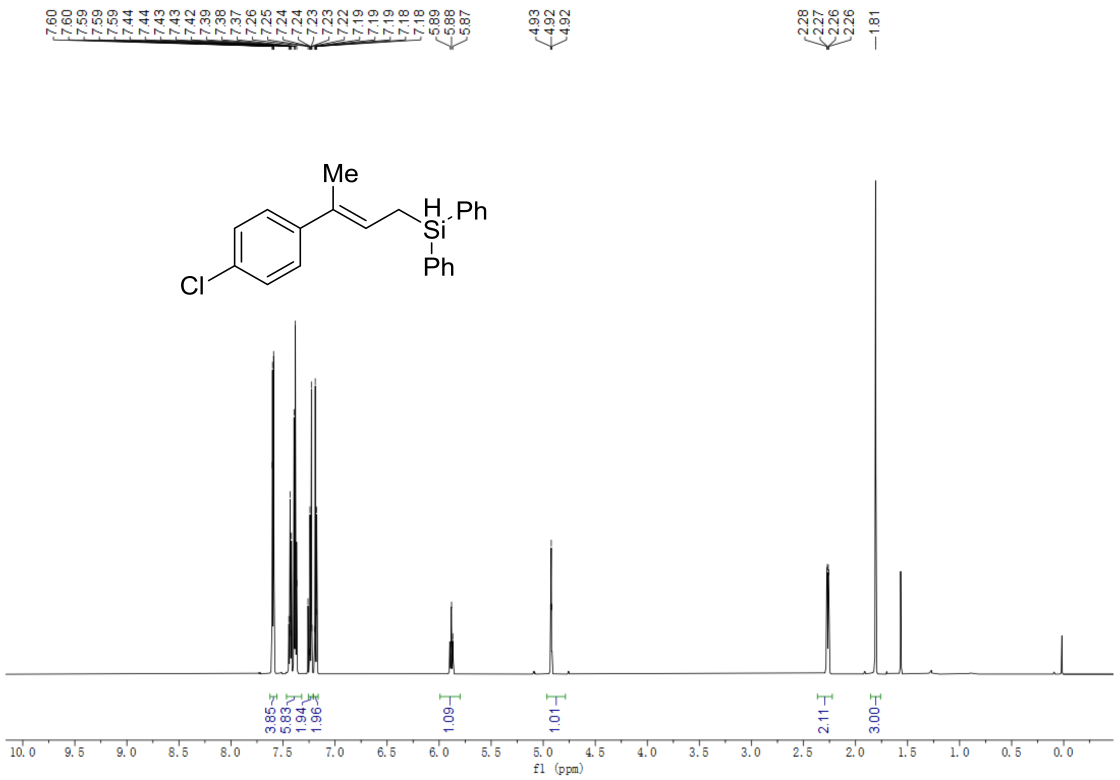

Supplementary Figure 24. ^{1}H NMR (600 MHz, $CDCl_3$, 25 ºC) spectra of **3j**.

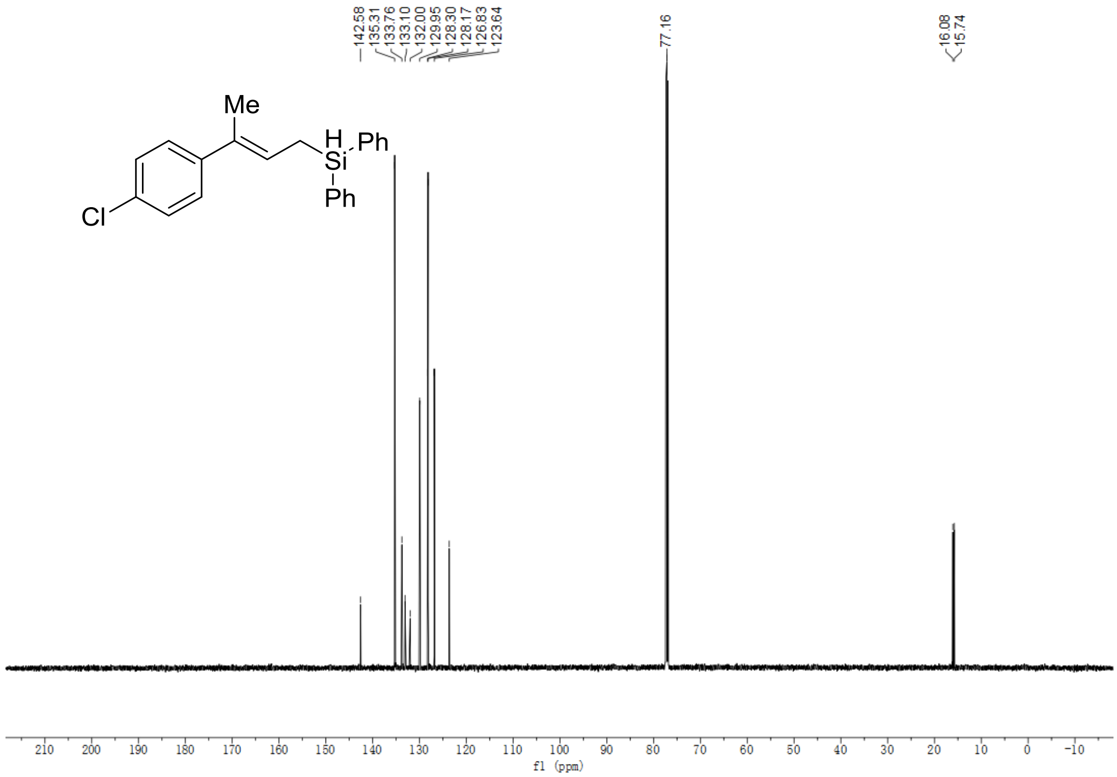

Supplementary Figure 25. ^{13}C NMR (151 MHz, $CDCl_3$, 25 ºC) spectra of **3j**.

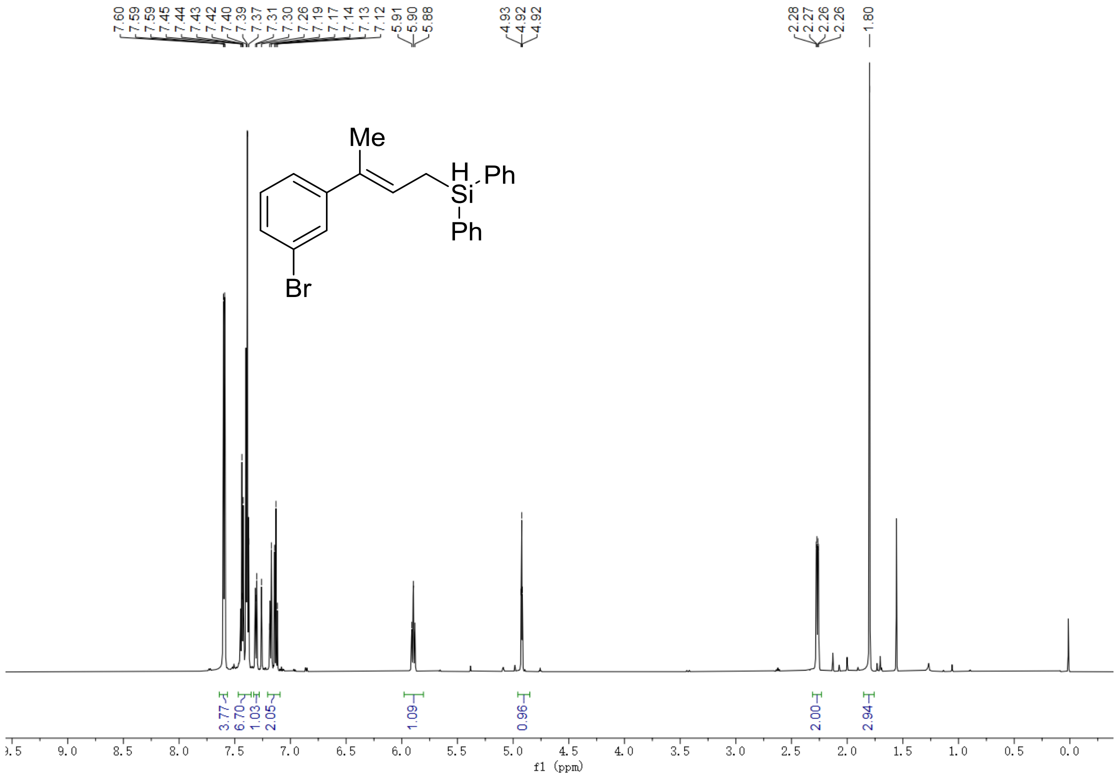

Supplementary Figure 26. ^{1}H NMR (600 MHz, $CDCl_3$, 25 ºC) spectra of **3k**.

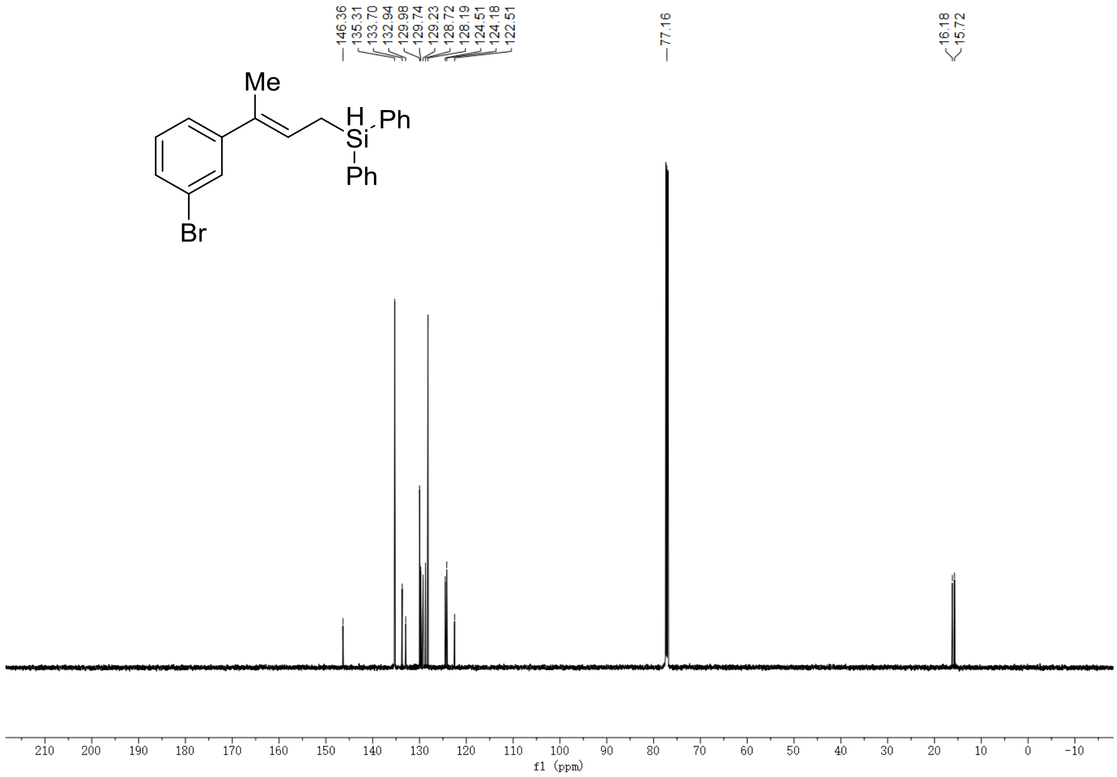

Supplementary Figure 27. ^{13}C NMR (151 MHz, $CDCl_3$, 25 ºC) spectra of **3k**.

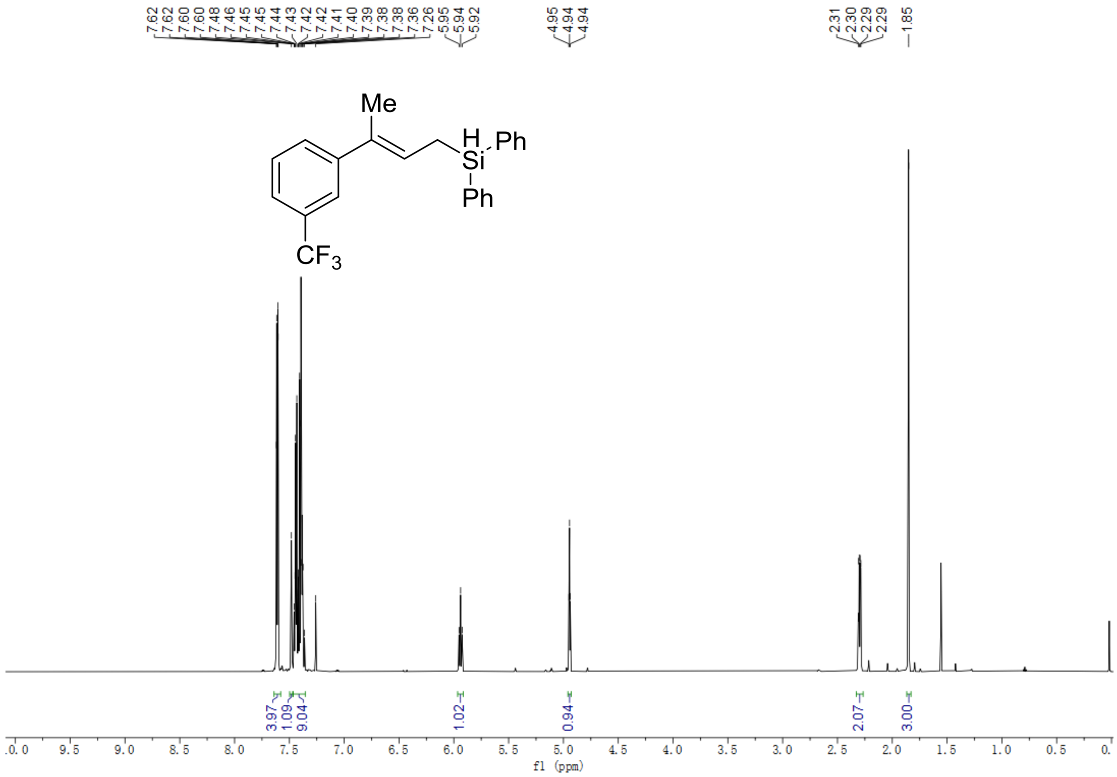

Supplementary Figure 28. ^{1}H NMR (600 MHz, $CDCl_3$, 25 ºC) spectra of **3l**.

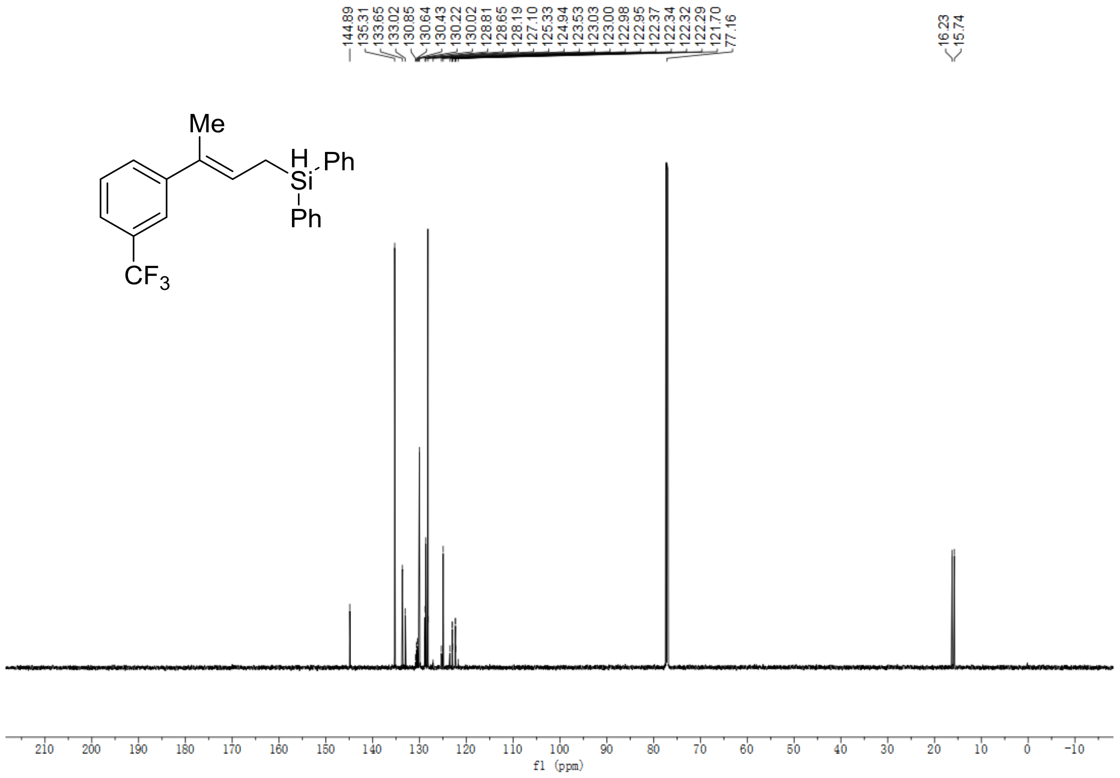

Supplementary Figure 29. ^{13}C NMR (151 MHz, $CDCl_3$, 25 ºC) spectra of **3l**.

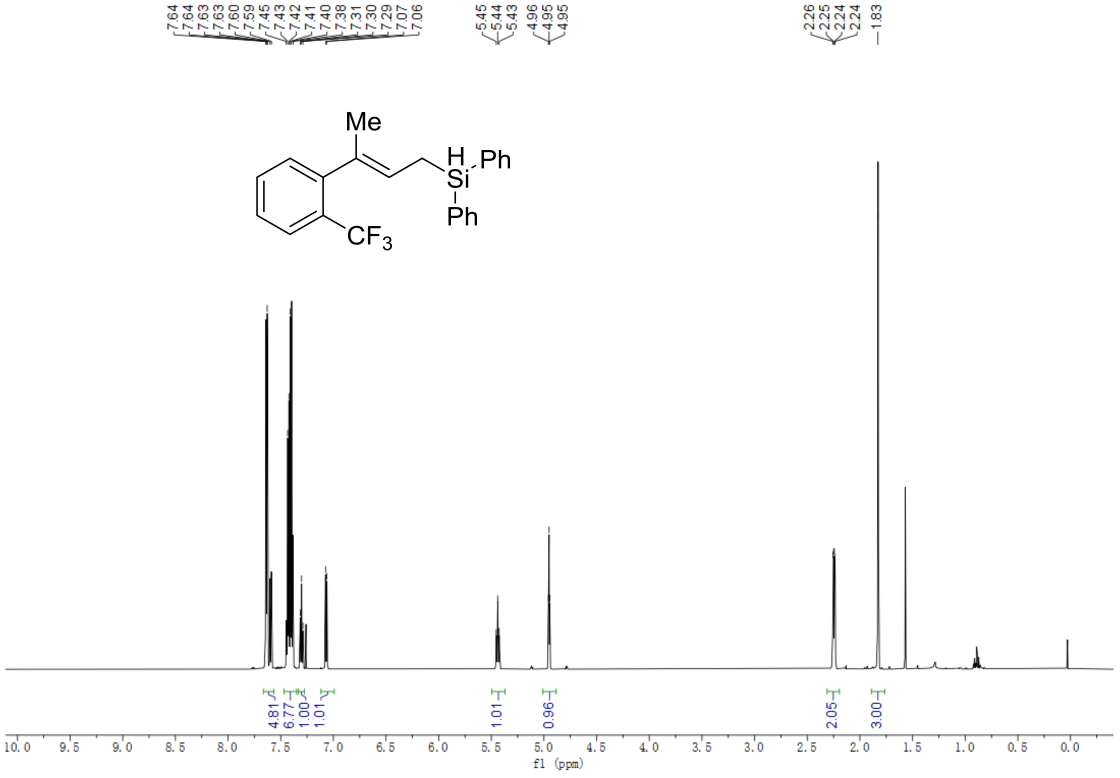

Supplementary Figure 30. ^{1}H NMR (600 MHz, $CDCl_3$, 25 ºC) spectra of **3m**.

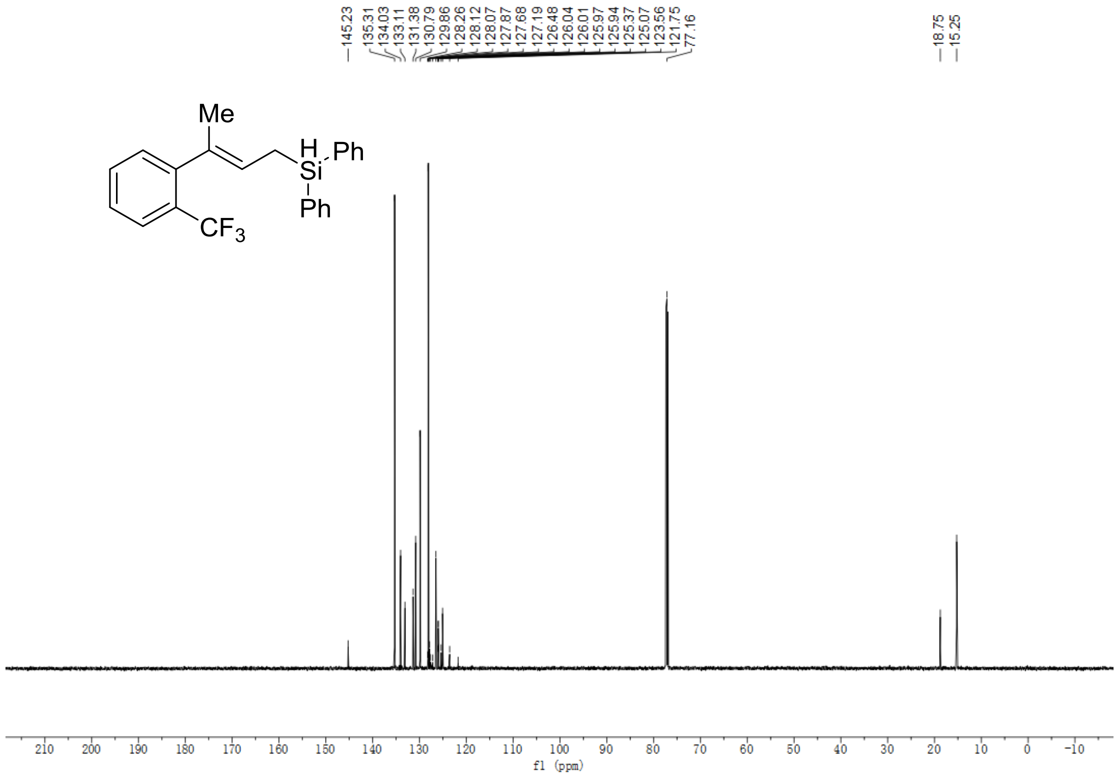

Supplementary Figure 31. ^{13}C NMR (151 MHz, $CDCl_3$, 25 ºC) spectra of **3m**.

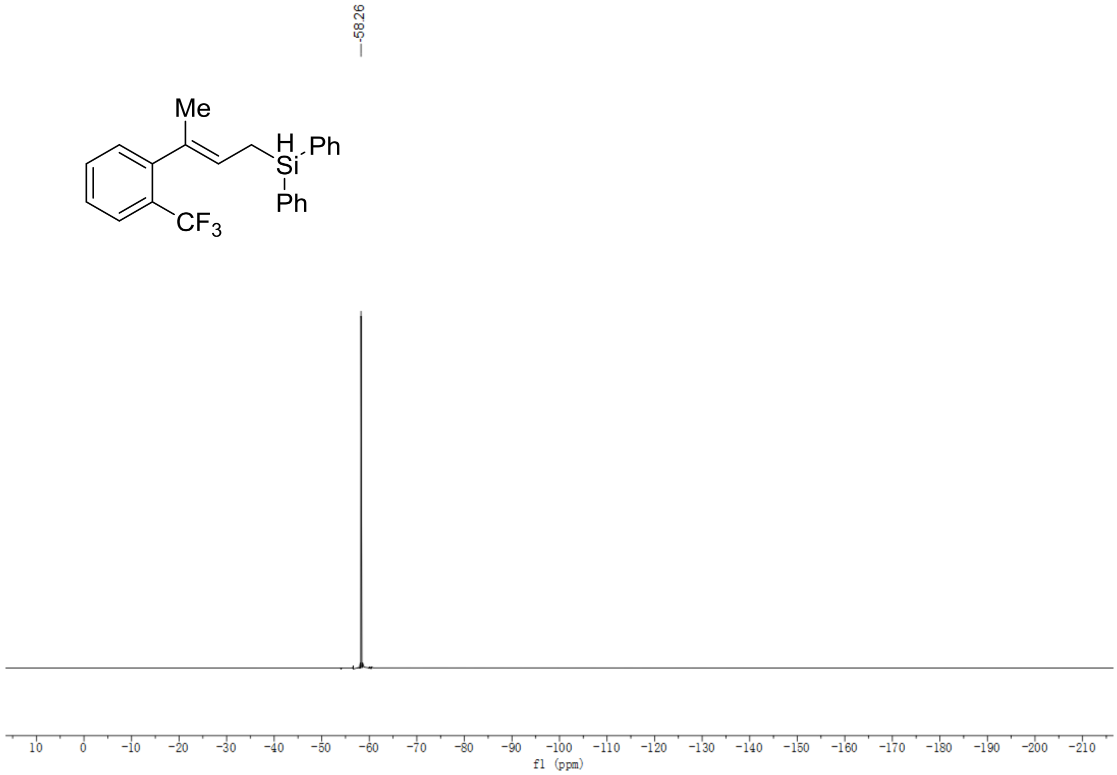

Supplementary Figure 32. ^{19}F NMR (565 MHz, CDCl3, 25 ºC) spectra of **3m**.

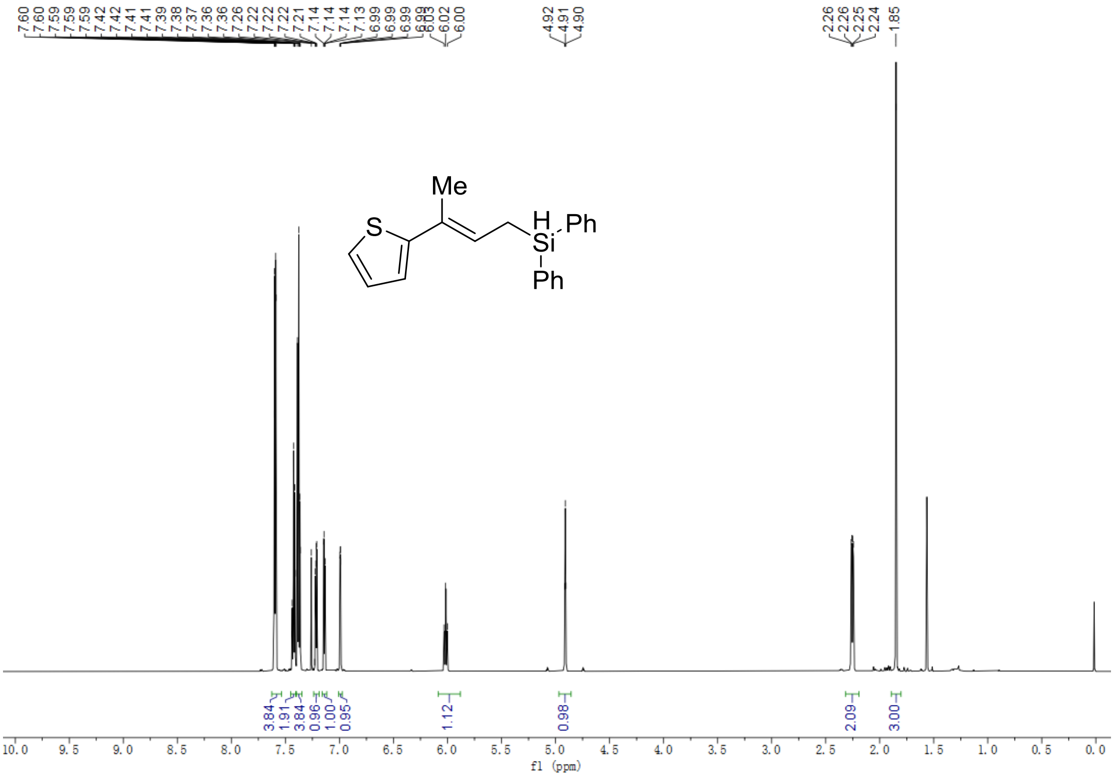

Supplementary Figure 33. ^{1}H NMR (600 MHz, $CDCl_3$, 25 ºC) spectra of **3n**.

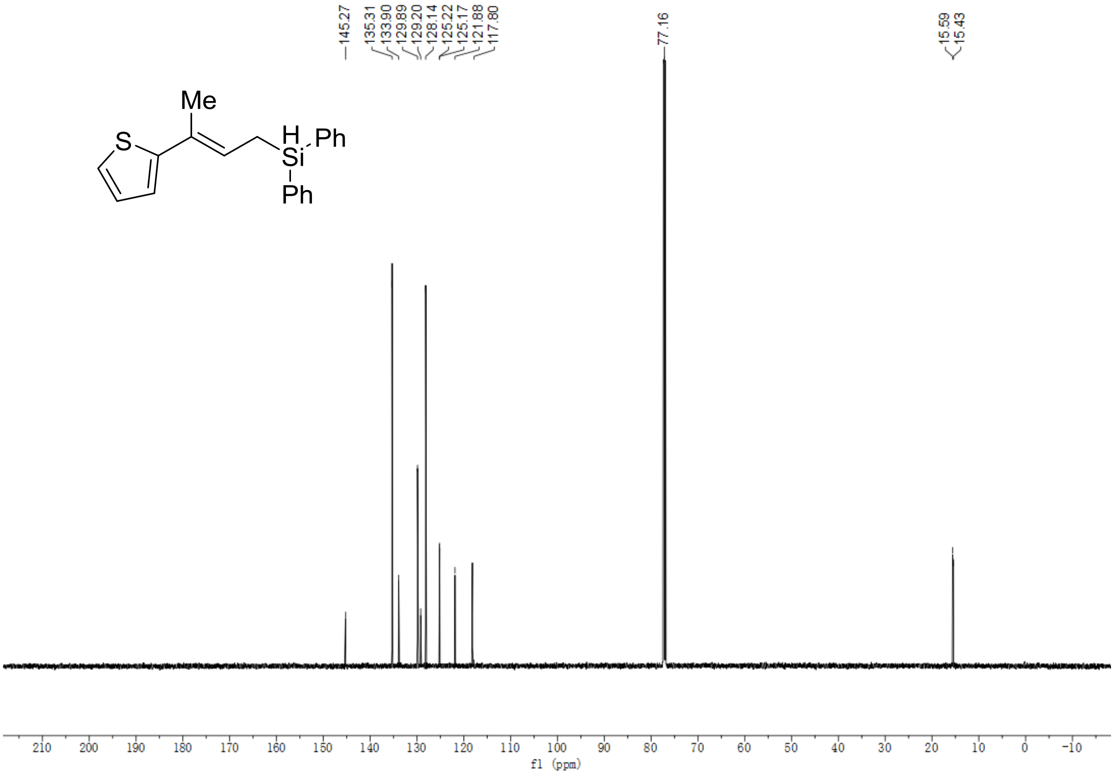

Supplementary Figure 34. ^{13}C NMR (151 MHz, $CDCl_3$, 25 ºC) spectra of **3n**.

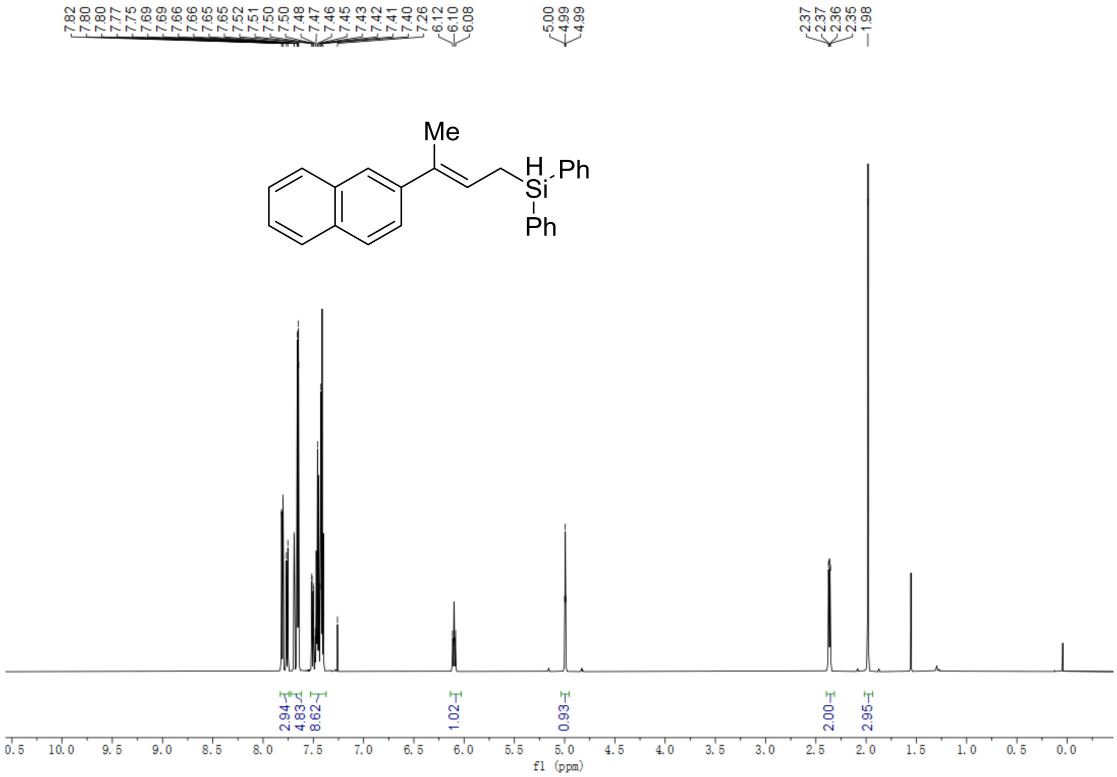

Supplementary Figure 35. ¹H NMR (600 MHz, $CDCl_3$, 25 ºC) spectra of **3o**.

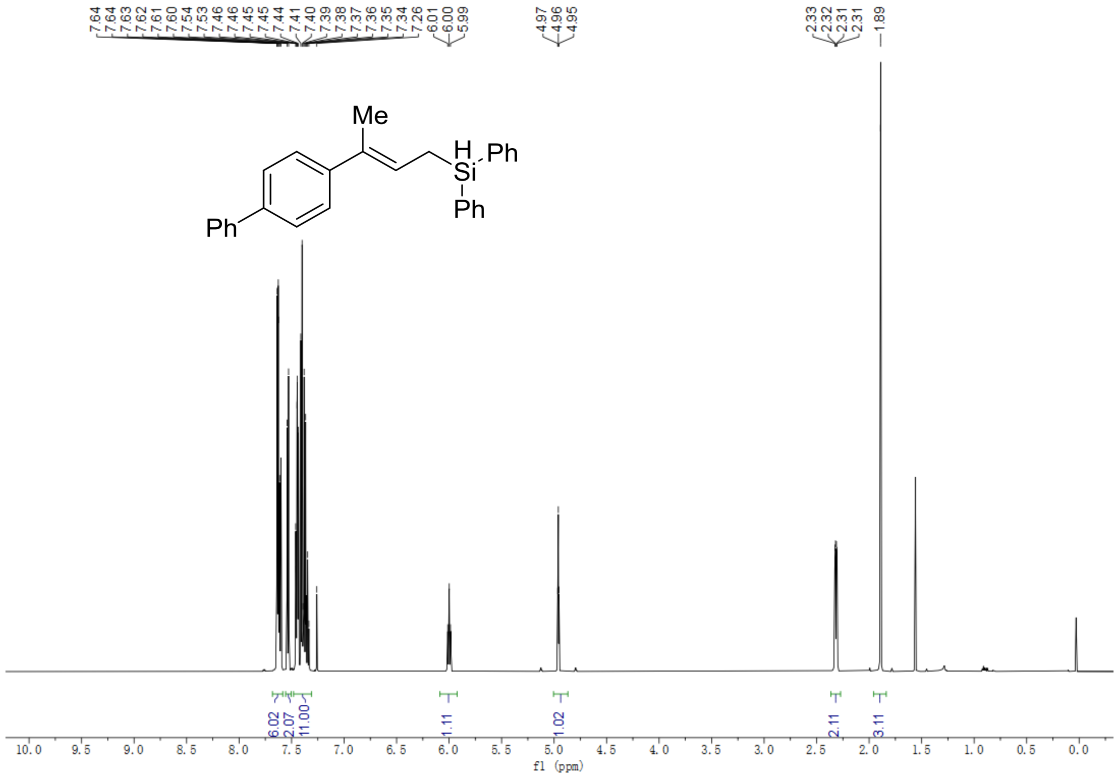

Supplementary Figure 36. ¹H NMR (600 MHz, $CDCl_3$, 25 ºC) spectra of **3p**.

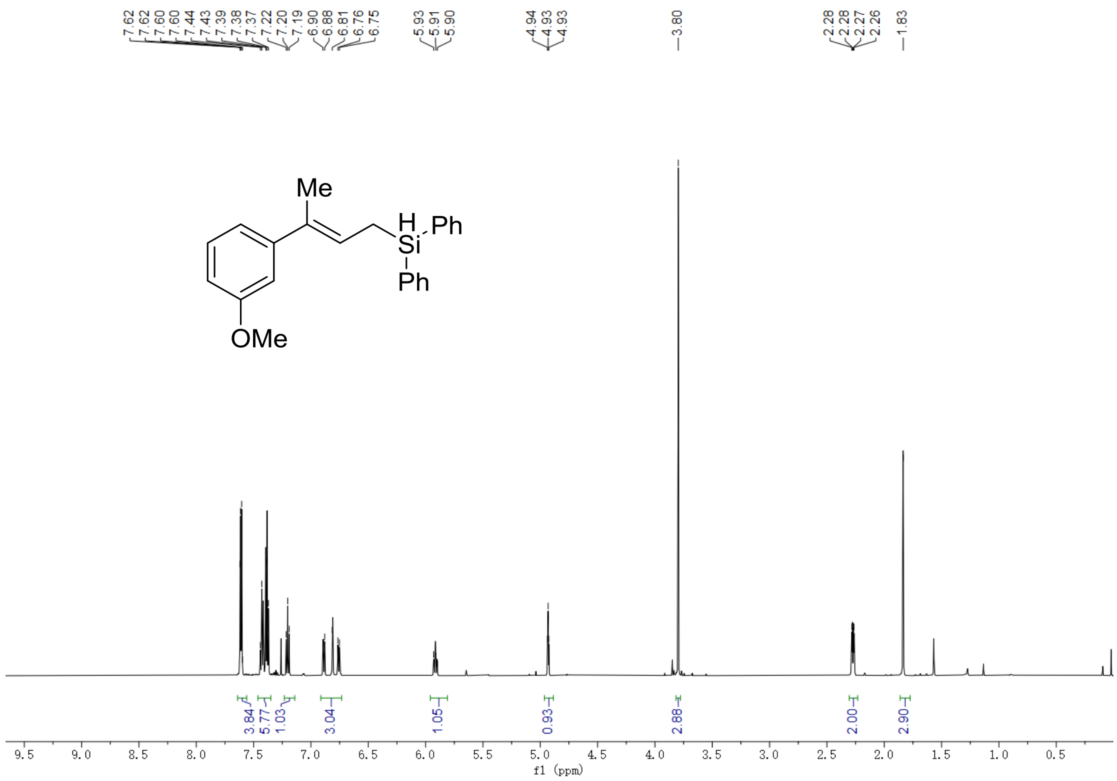

Supplementary Figure 37. ^{1}H NMR (600 MHz, $CDCl_3$, 25 ºC) spectra of **3q**.

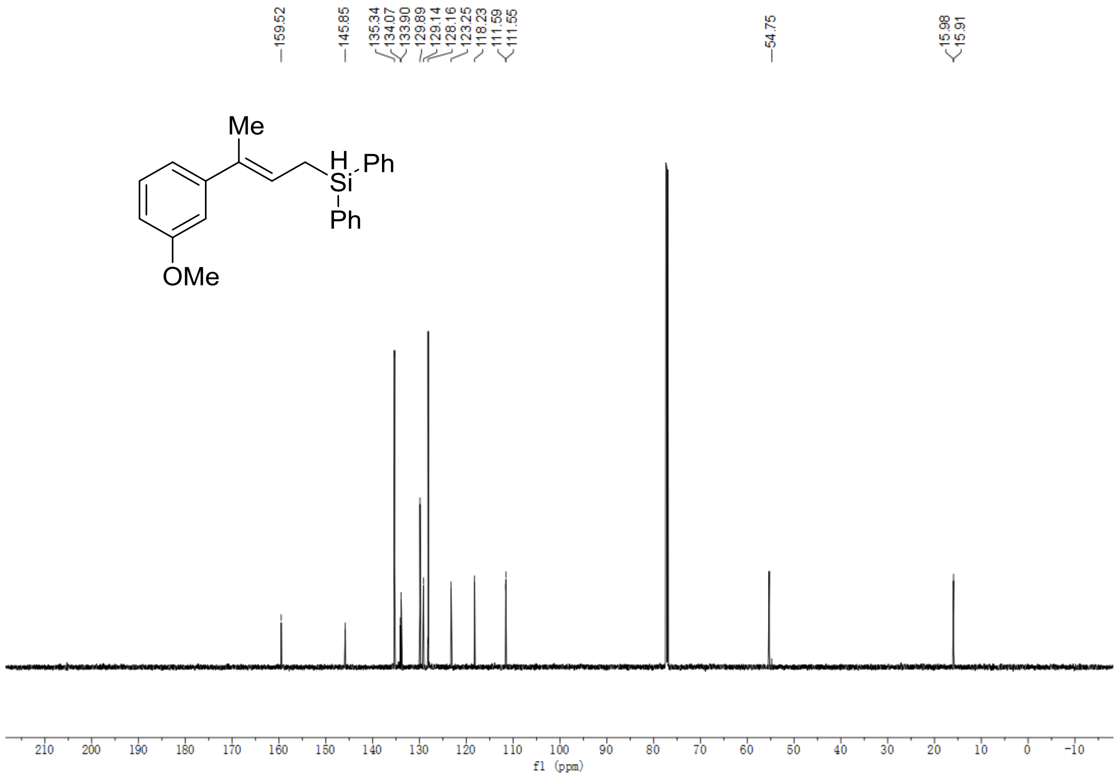

Supplementary Figure 38. ^{13}C NMR (151 MHz, $CDCl_3$, 25 ºC) spectra of **3q**.

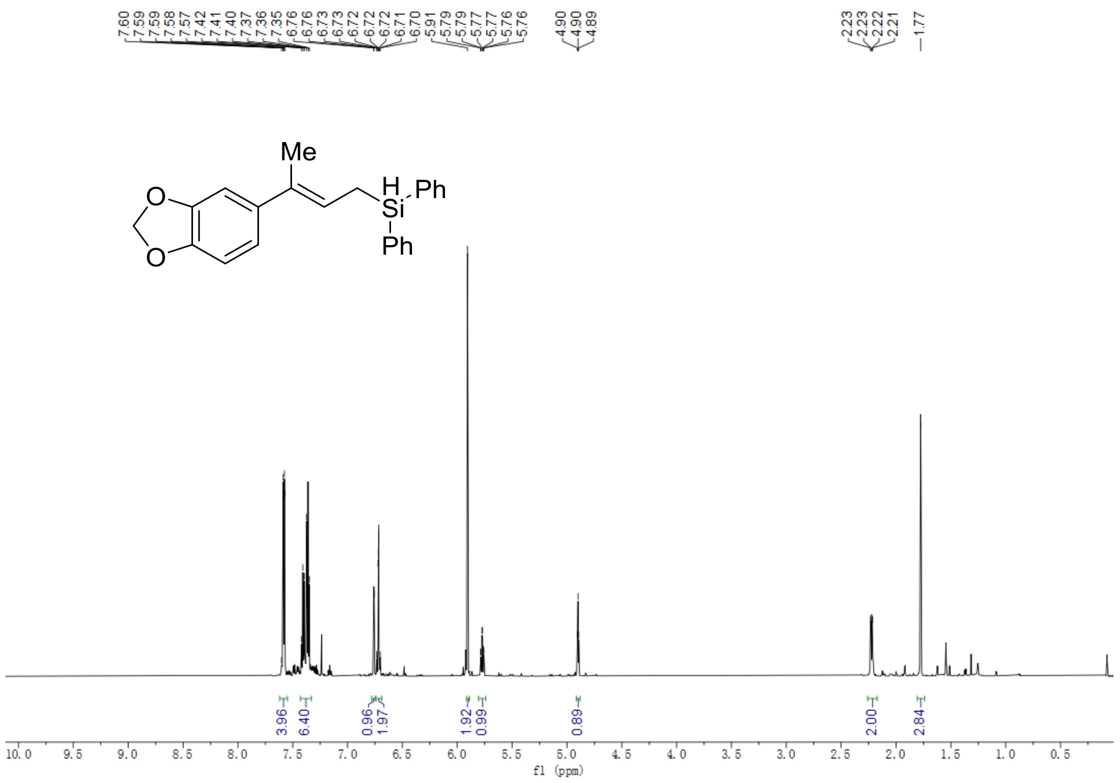

Supplementary Figure 39. ^{1}H NMR (600 MHz, $CDCl_3$, 25 ºC) spectra of **3r**.

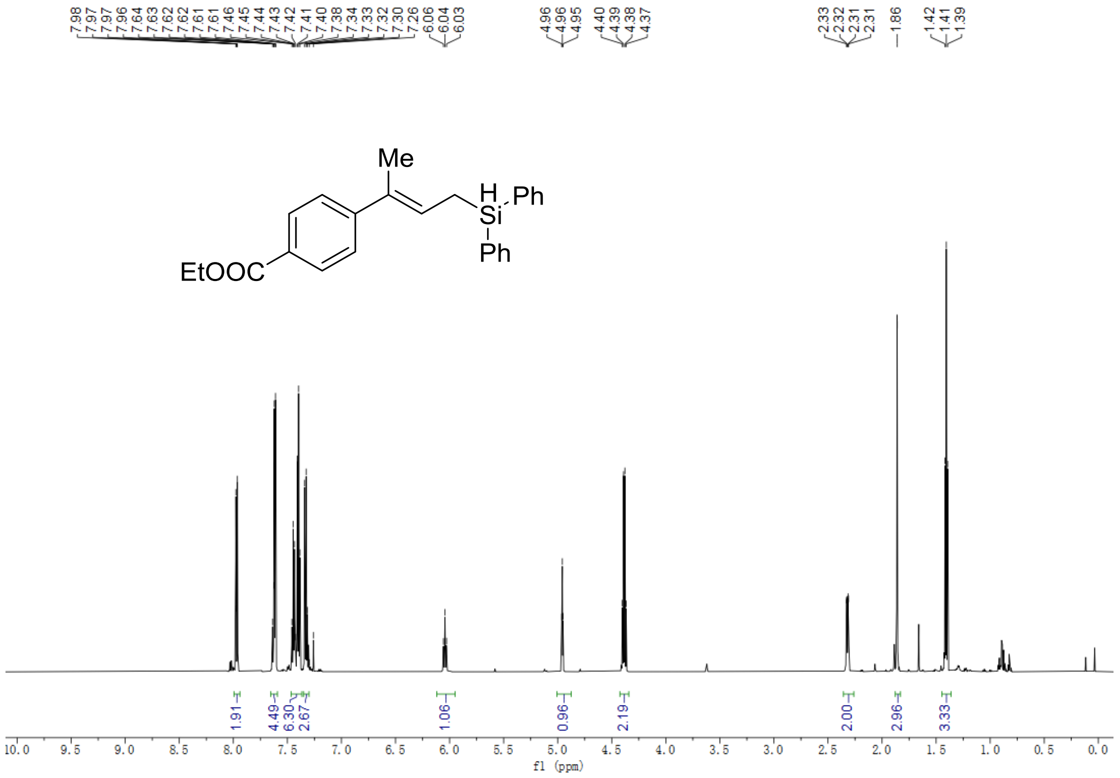

Supplementary Figure 40. ^{1}H NMR (600 MHz, $CDCl_3$, 25 ºC) spectra of **3s**.

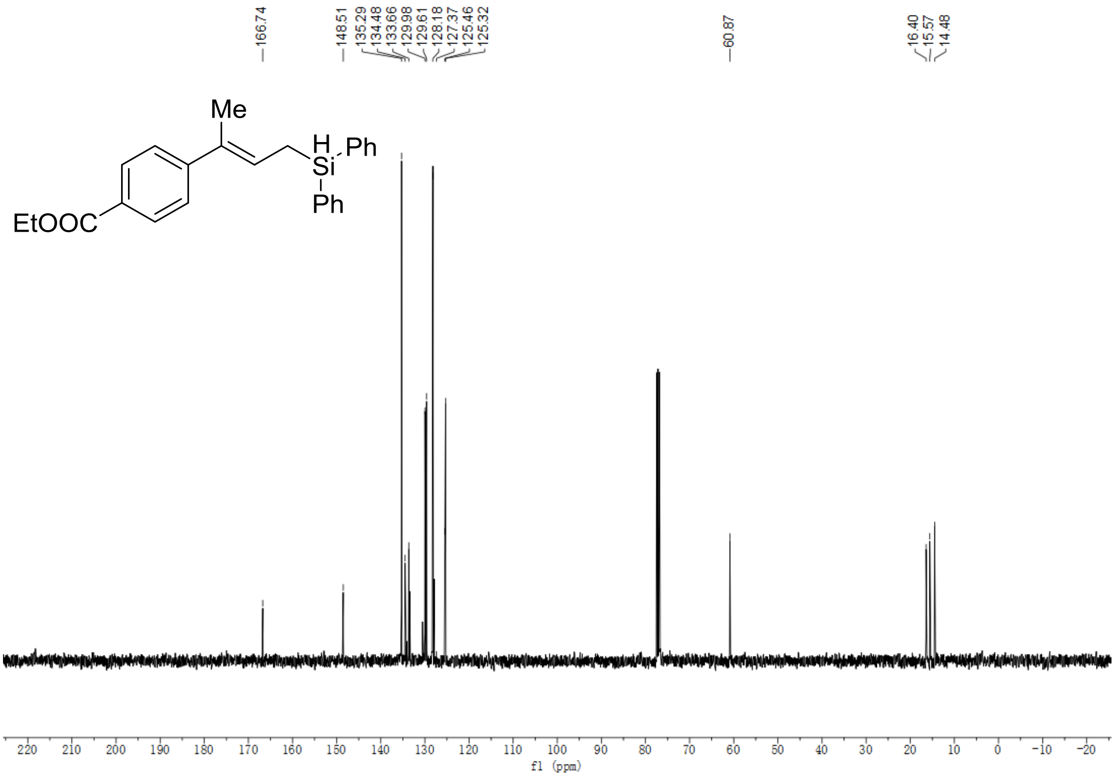

Supplementary Figure 41. ^{13}C NMR (151 MHz, $CDCl_3$, 25 ºC) spectra of **3s**.

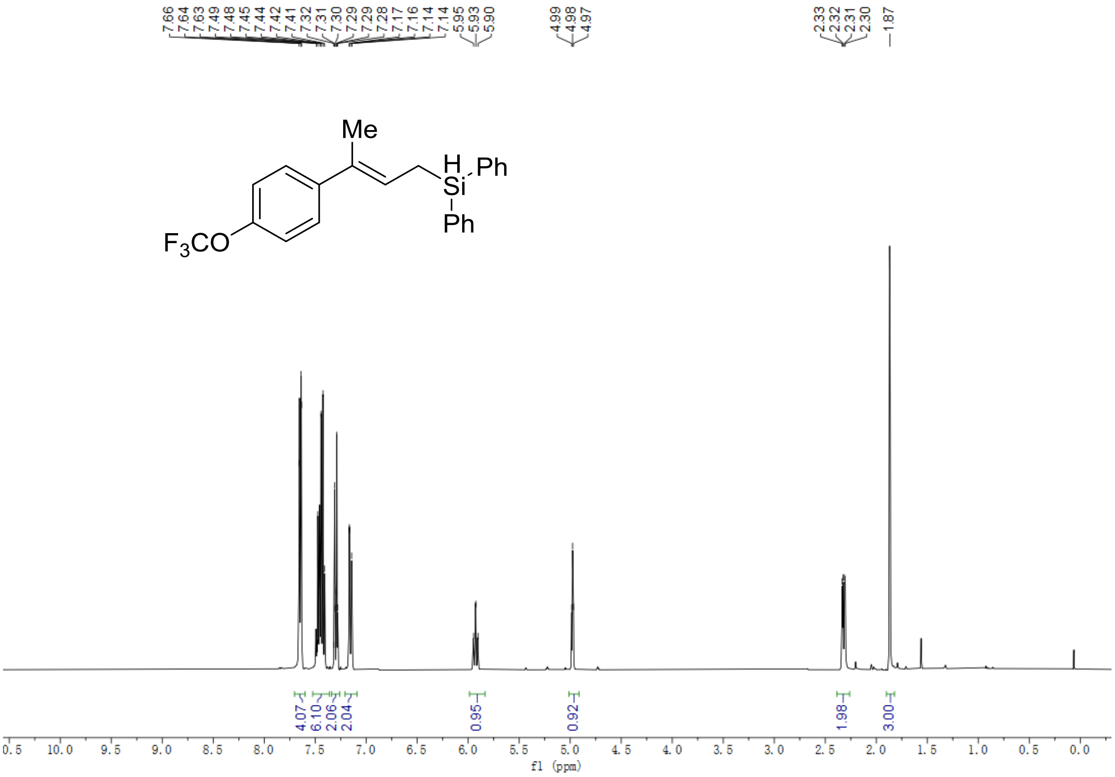

Supplementary Figure 42. ^{1}H NMR (600 MHz, $CDCl_3$, 25 ºC) spectra of **3t**.

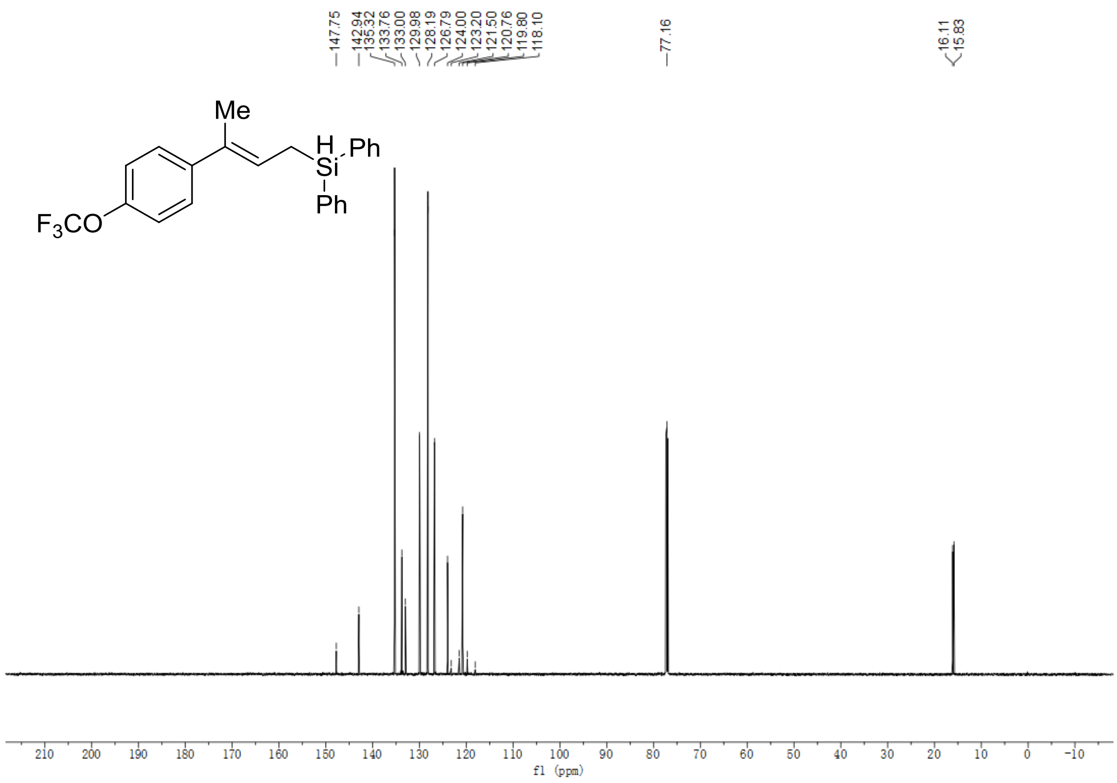

Supplementary Figure 43. ^{13}C NMR (151 MHz, $CDCl_3$, 25 ºC) spectra of **3t**.

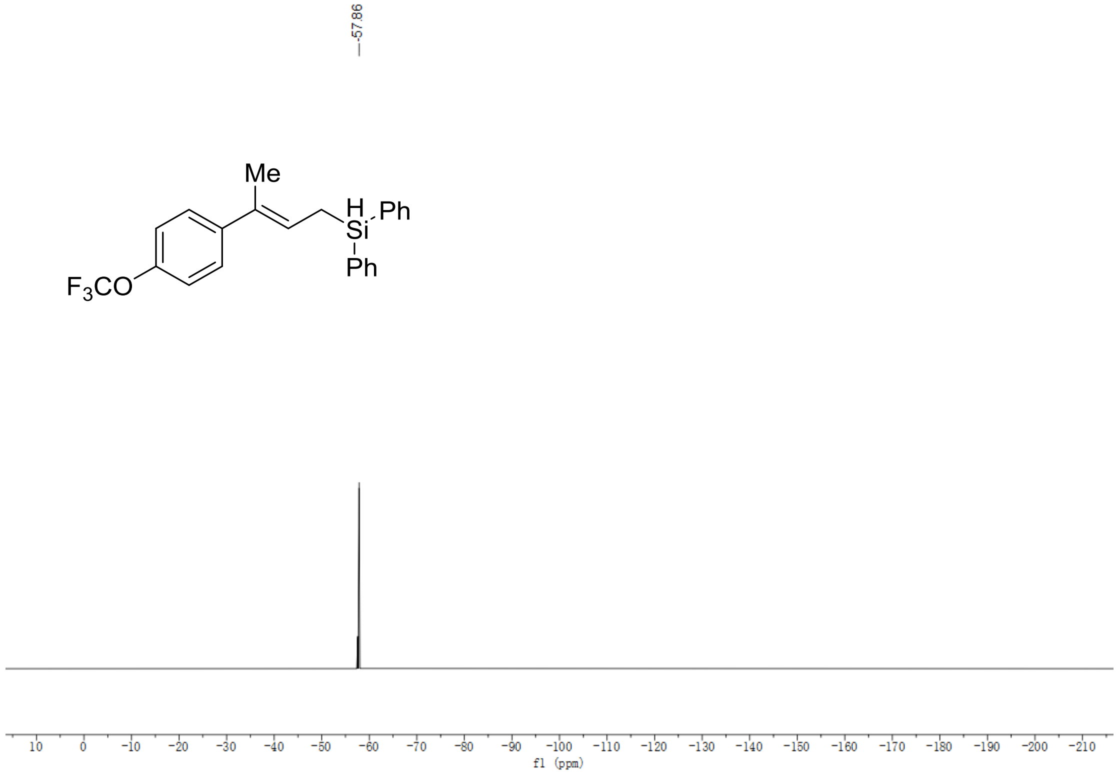

Supplementary Figure 44. ^{19}F NMR (565 MHz, CDCl3, 25 ºC) spectra of **3t**.

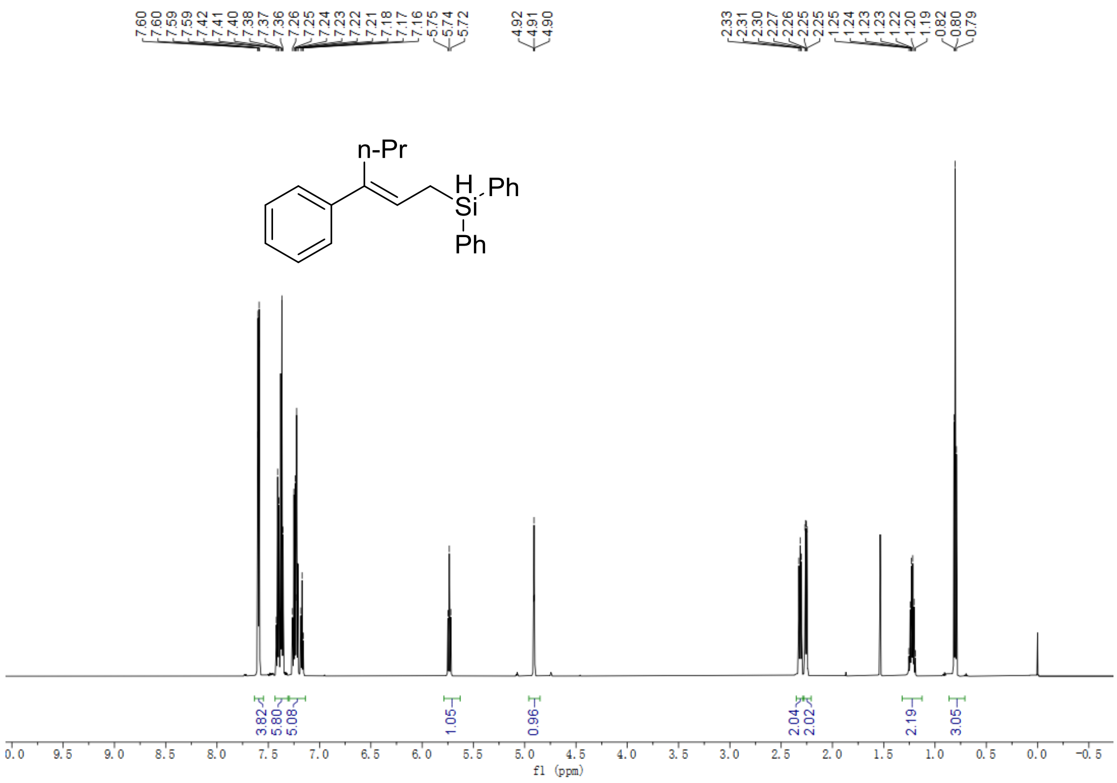

Supplementary Figure 45. ^{1}H NMR (600 MHz, $CDCl_3$, 25 ºC) spectra of **3u**.

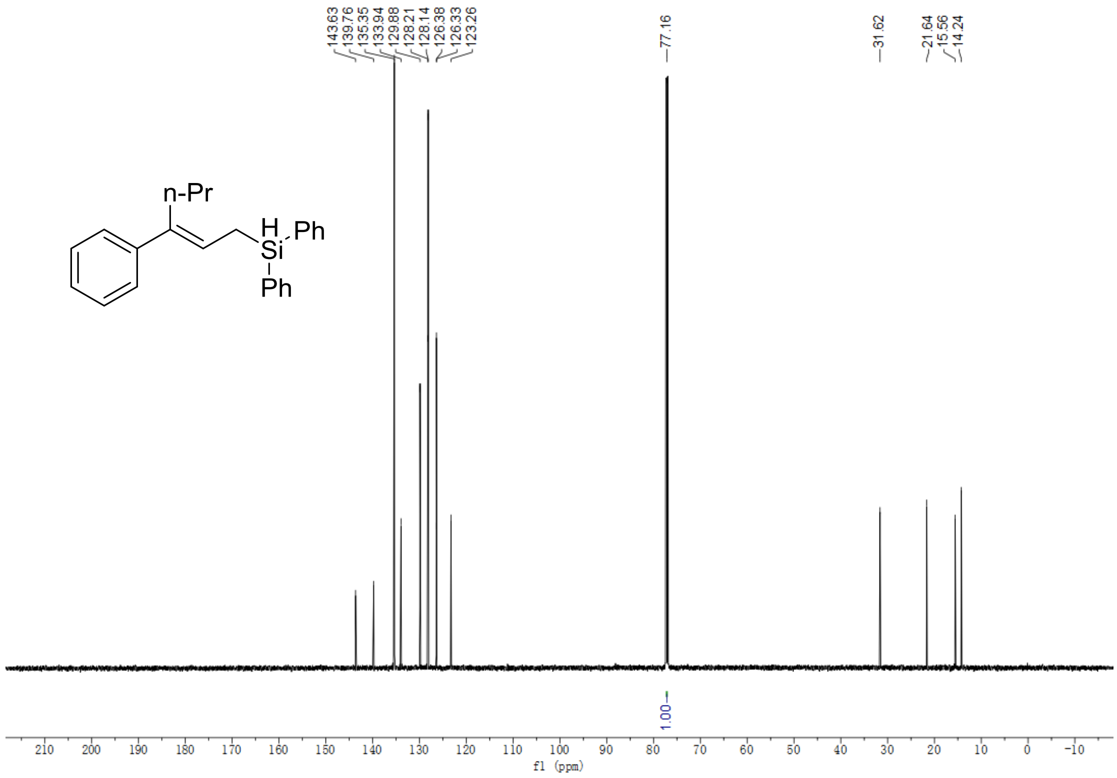

Supplementary Figure 46. ^{13}C NMR (151 MHz, $CDCl_3$, 25 ºC) spectra of **3u**.

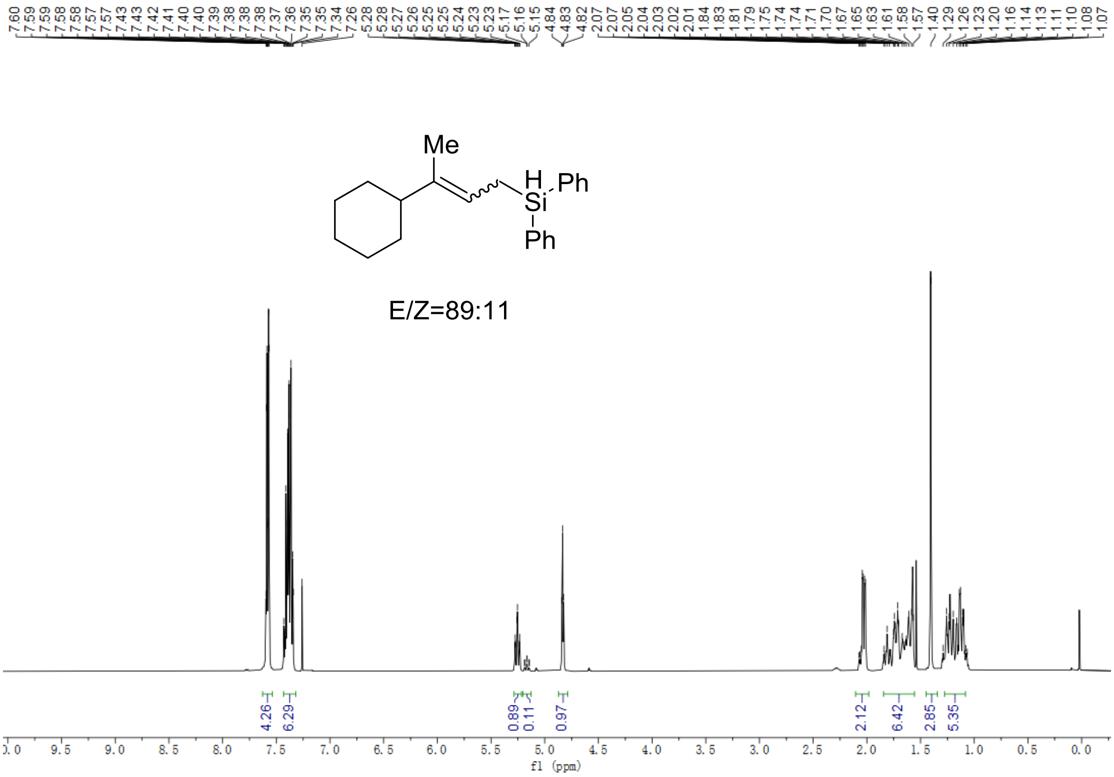

Supplementary Figure 47. ^{1}H NMR (600 MHz, $CDCl_3$, 25 ºC) spectra of **3w**.

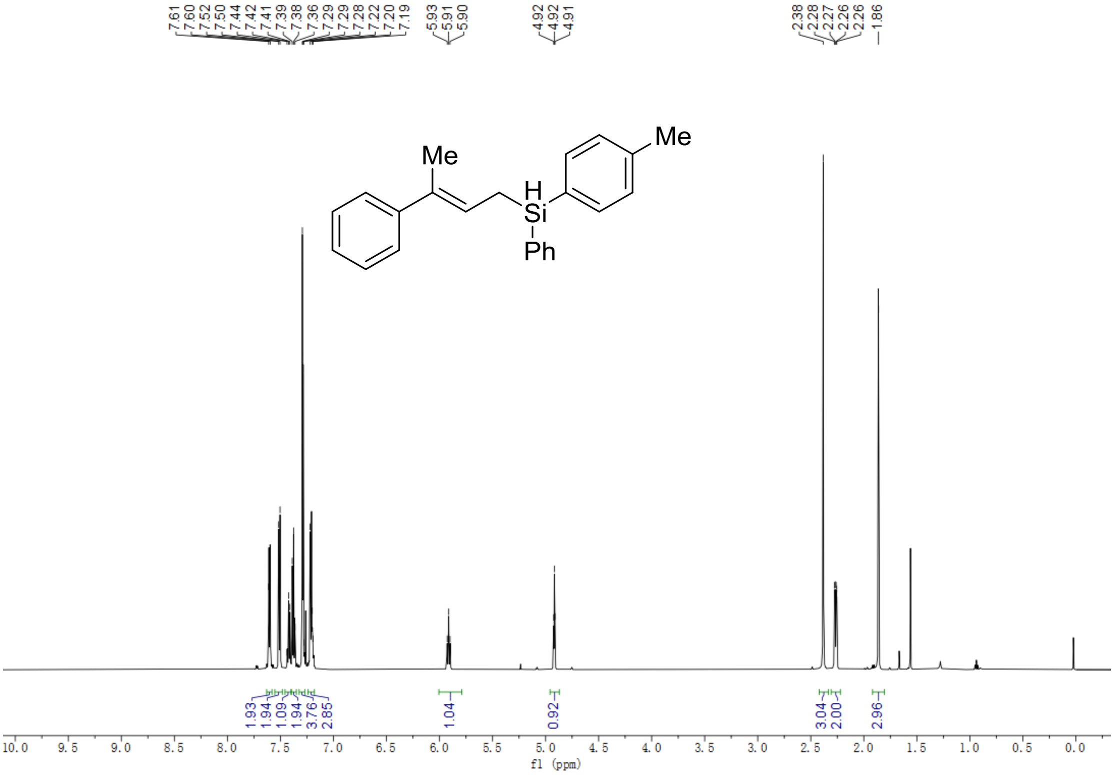

Supplementary Figure 48. ^{1}H NMR (600 MHz, $CDCl_3$, 25 ºC) spectra of **3x**.

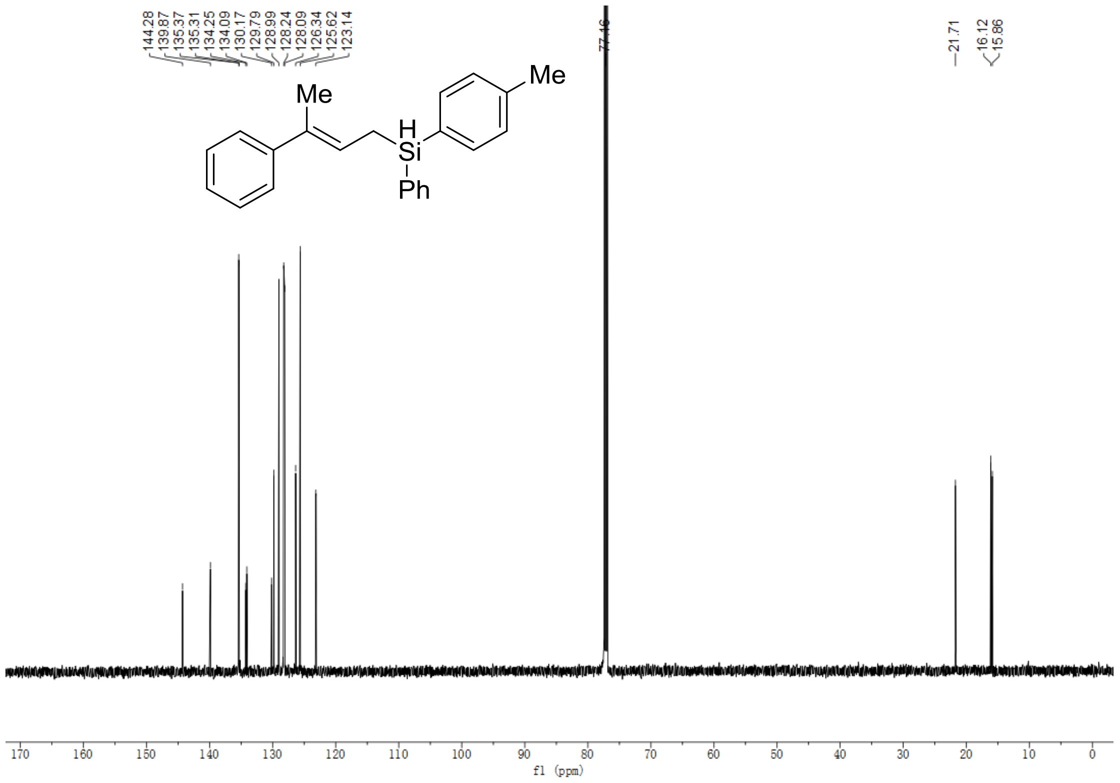

Supplementary Figure 49. ^{13}C NMR (151 MHz, $CDCl_3$, 25 ºC) spectra of **3x**.

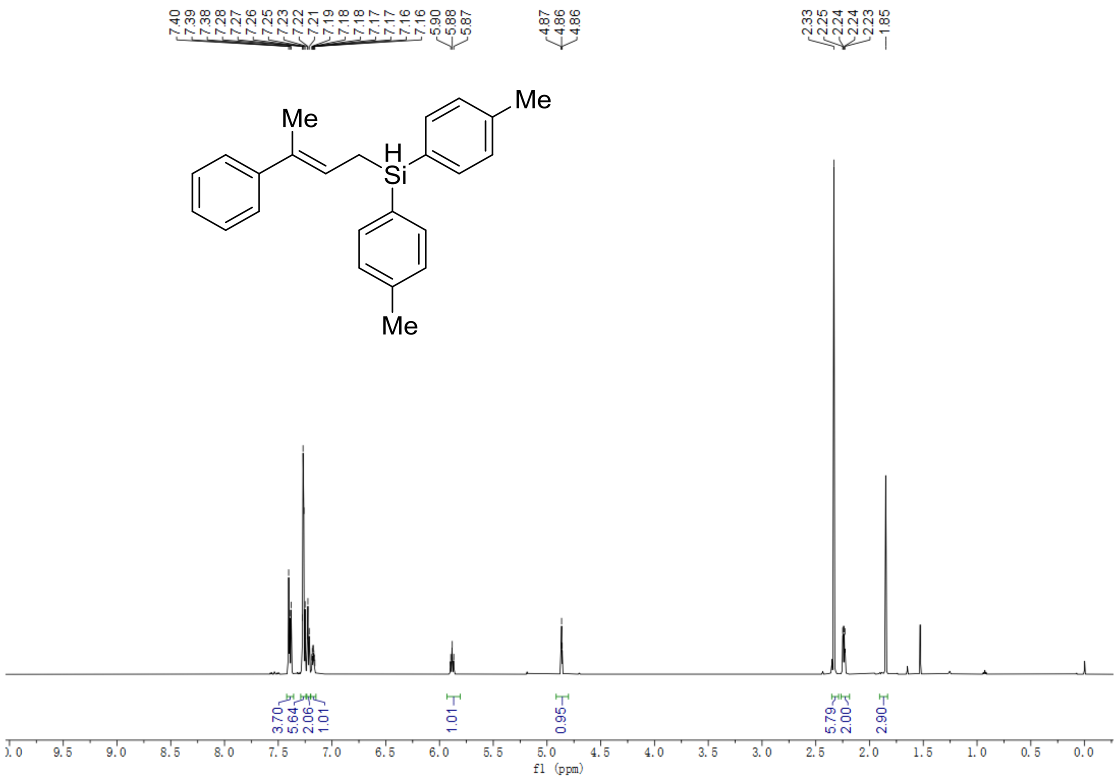

Supplementary Figure 50. ^{1}H NMR (600 MHz, $CDCl_3$, 25 ºC) spectra of **3y**.

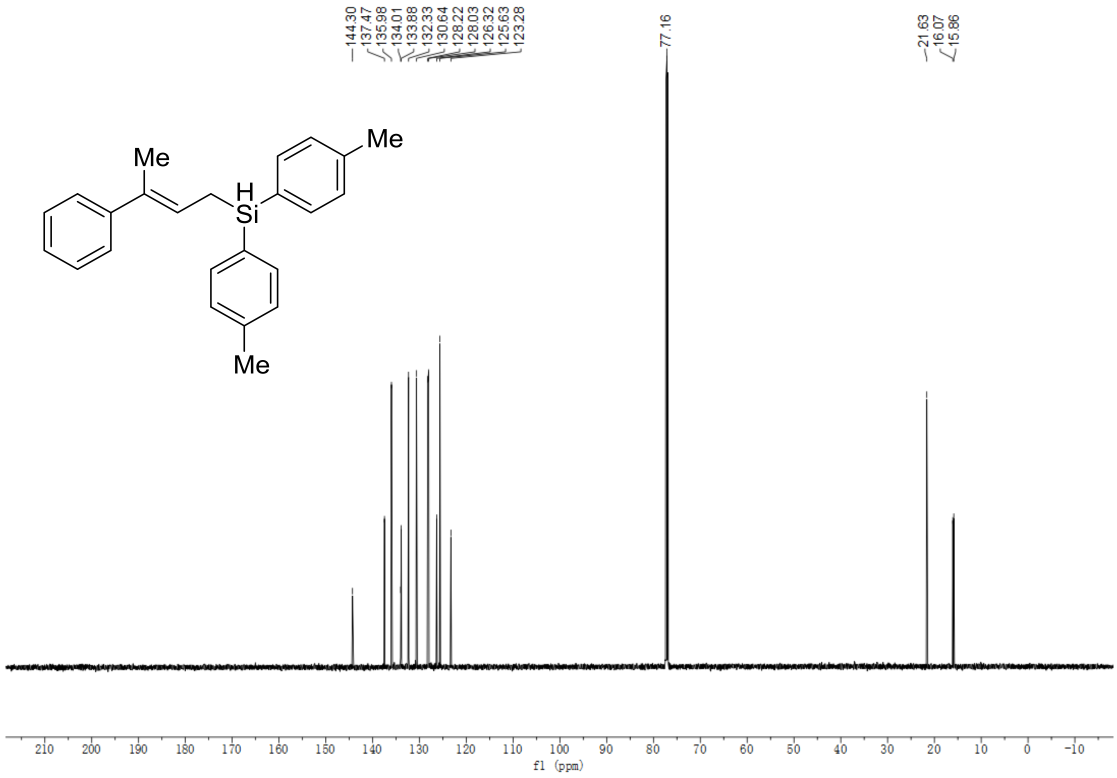

Supplementary Figure 51. ^{13}C NMR (151 MHz, $CDCl_3$, 25 ºC) spectra of **3y**.

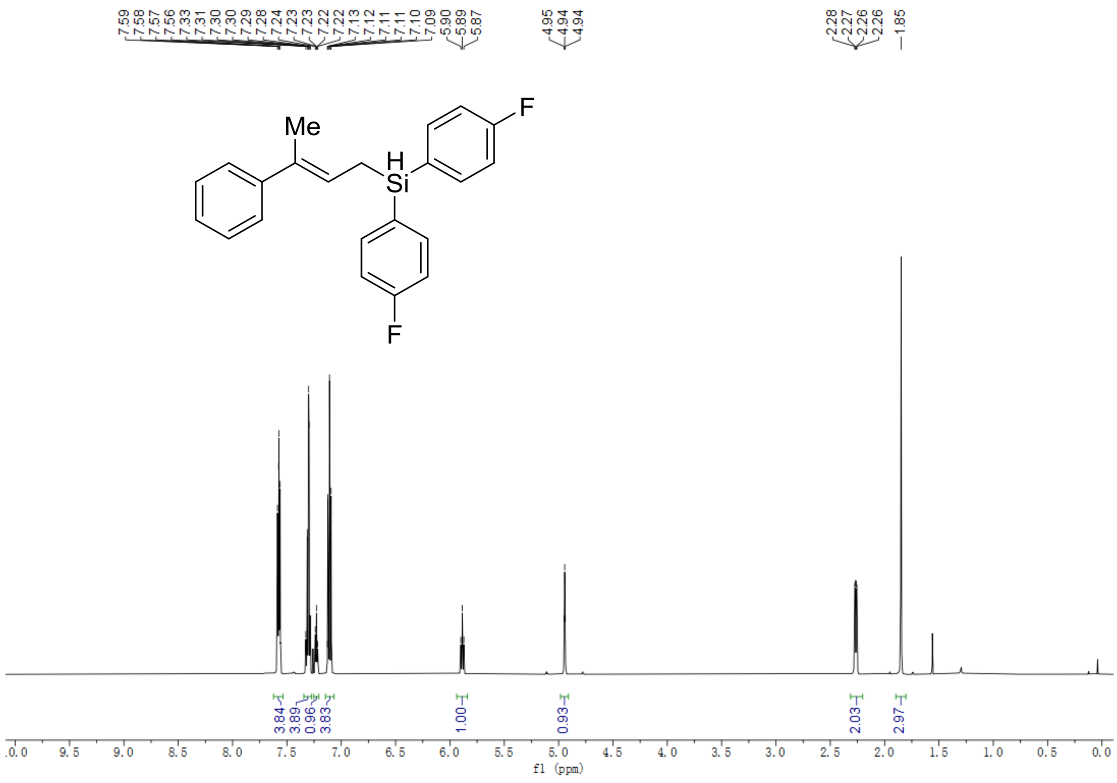

Supplementary Figure 52. ^{1}H NMR (600 MHz, $CDCl_3$, 25 ºC) spectra of **3z**.

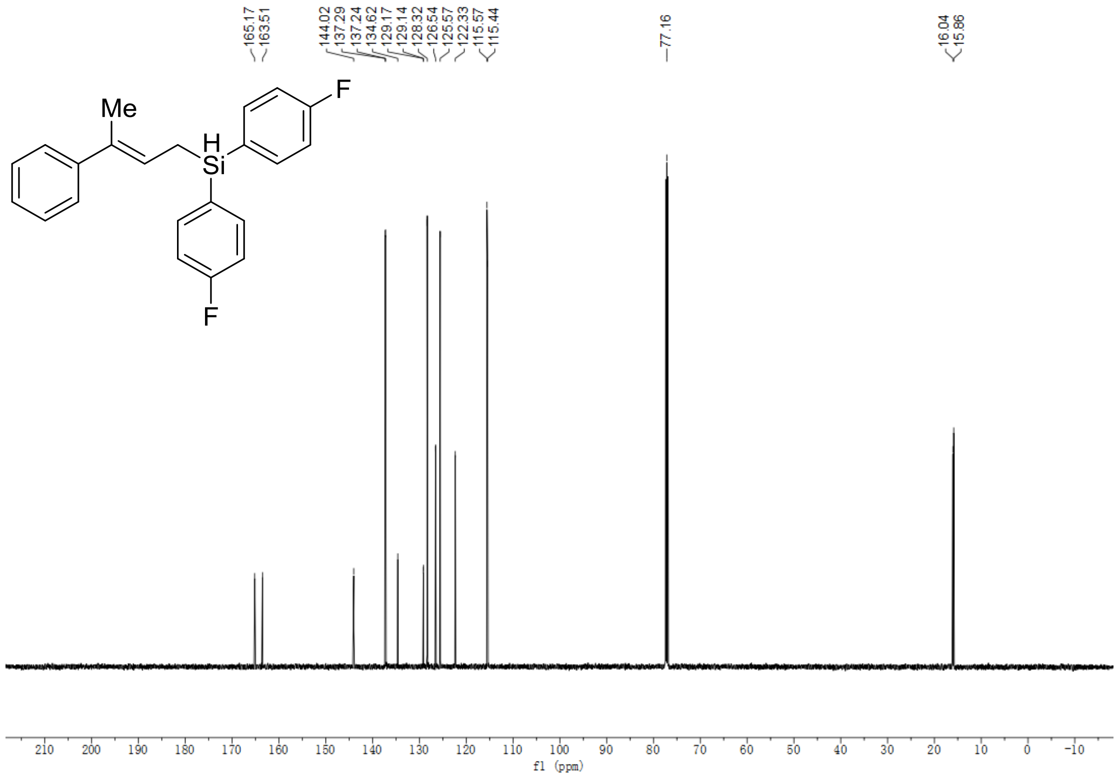

Supplementary Figure 53. ^{13}C NMR (151 MHz, $CDCl_3$, 25 ºC) spectra of **3z**.

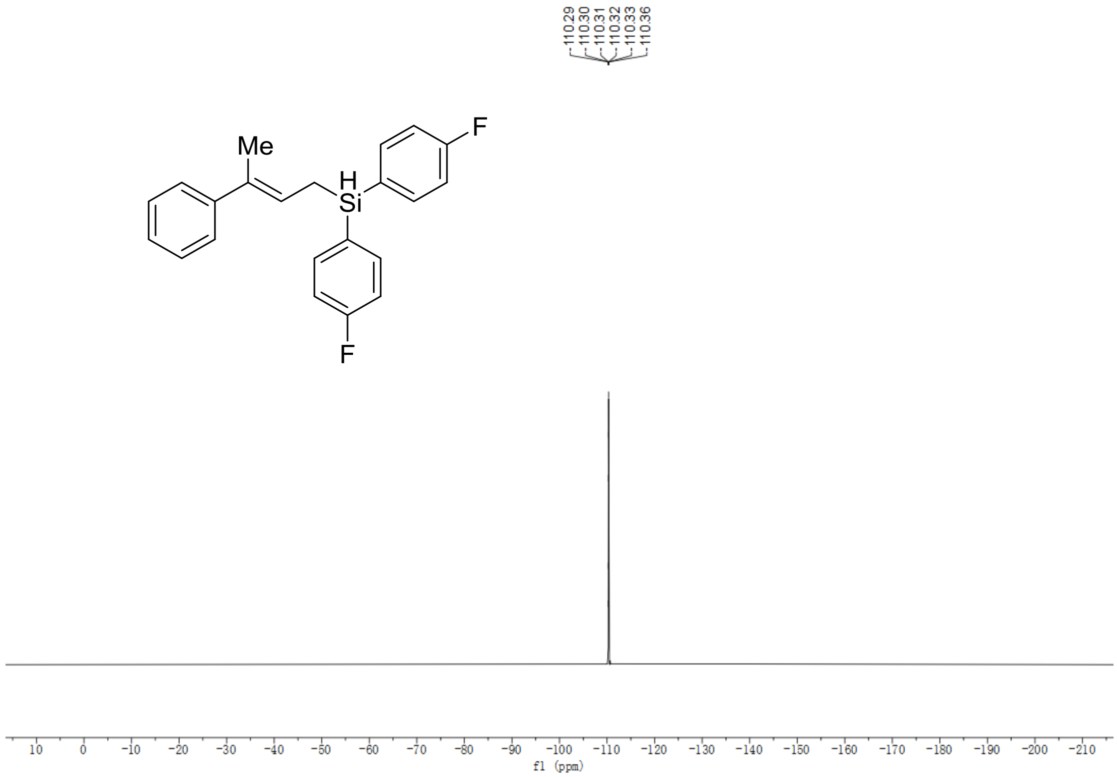

Supplementary Figure 54. ^{19}F NMR (565 MHz, $CDCl_3$, 25 ºC) spectra of **3z**.

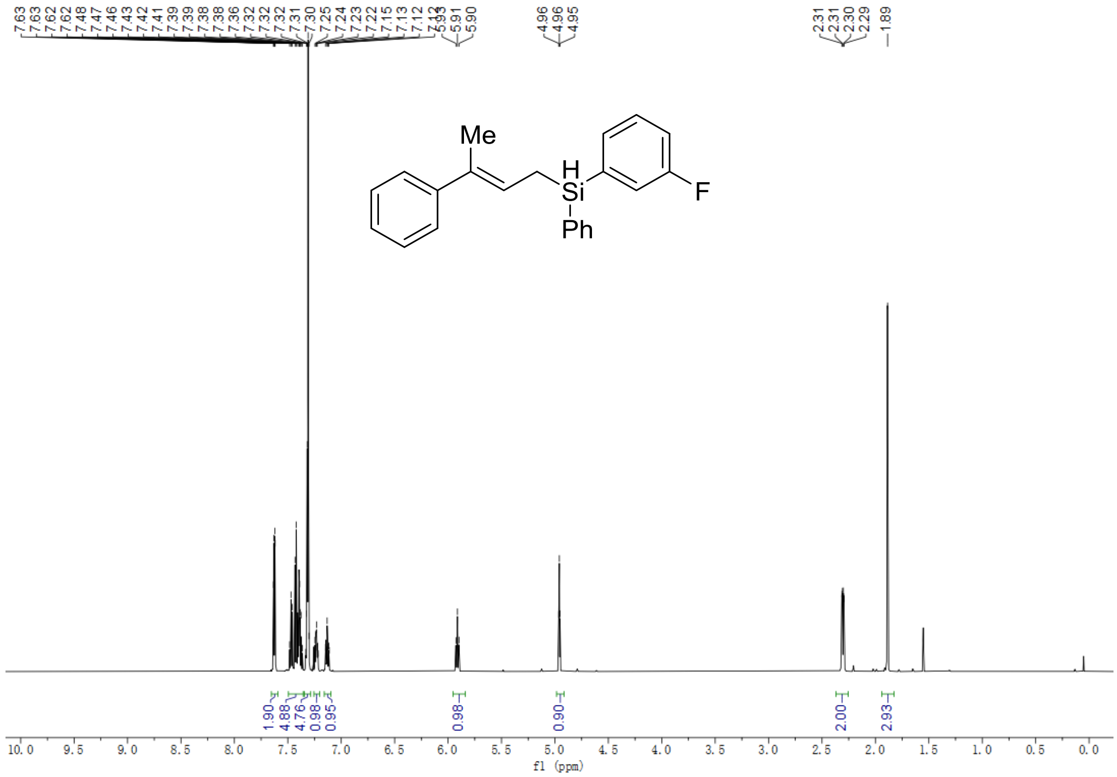

Supplementary Figure 55. ^{1}H NMR (600 MHz, $CDCl_3$, 25 ºC) spectra of **3aa**.

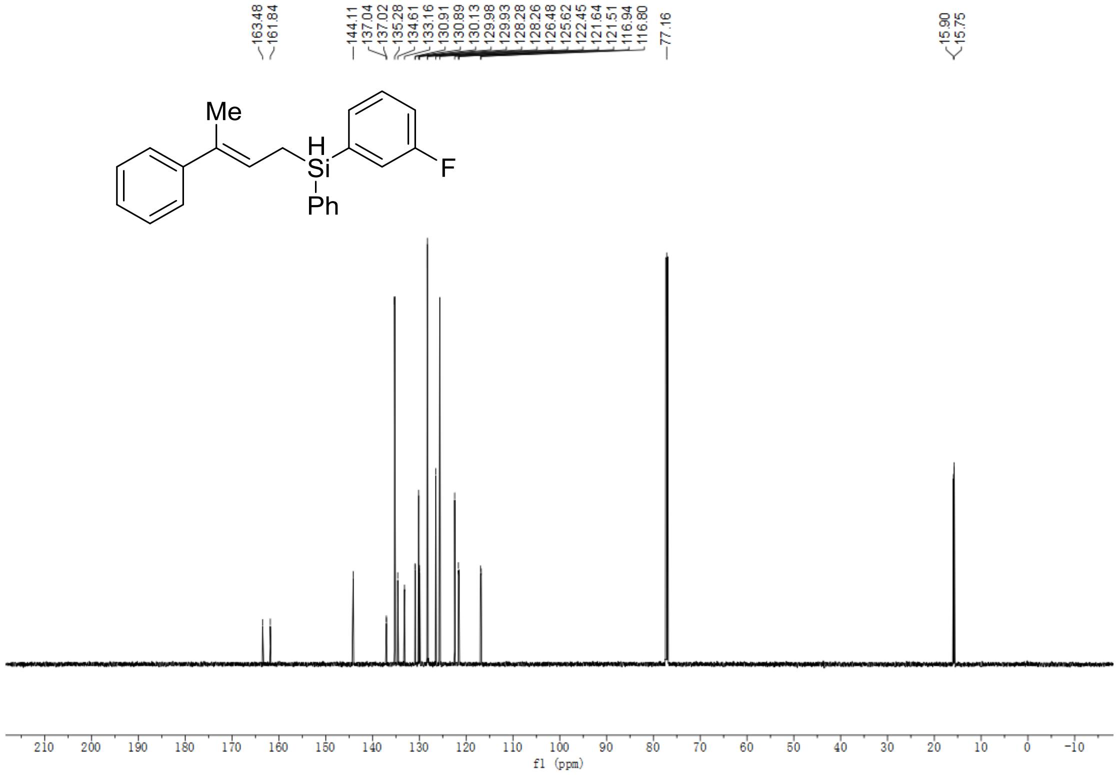

Supplementary Figure 56. ^{13}C NMR (151 MHz, $CDCl_3$, 25 ºC) spectra of **3aa**.

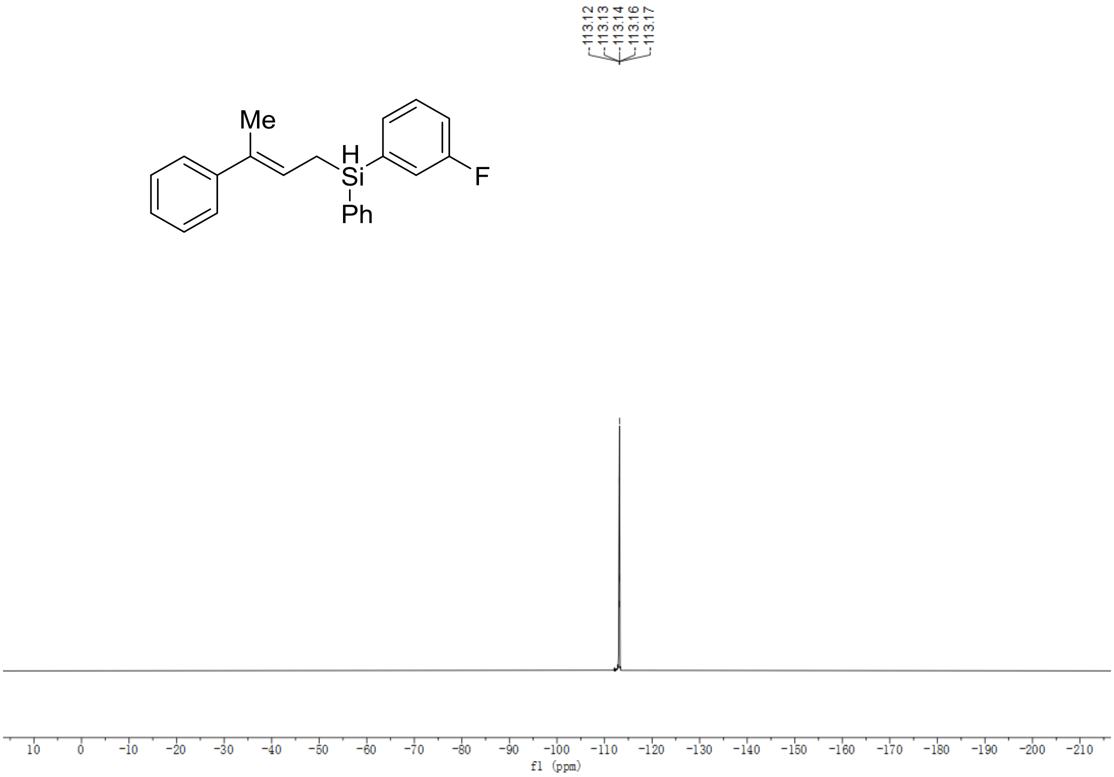

Supplementary Figure 57. ^{19}F NMR (565 MHz, CDCl3, 25 ºC) spectra of **3aa**.

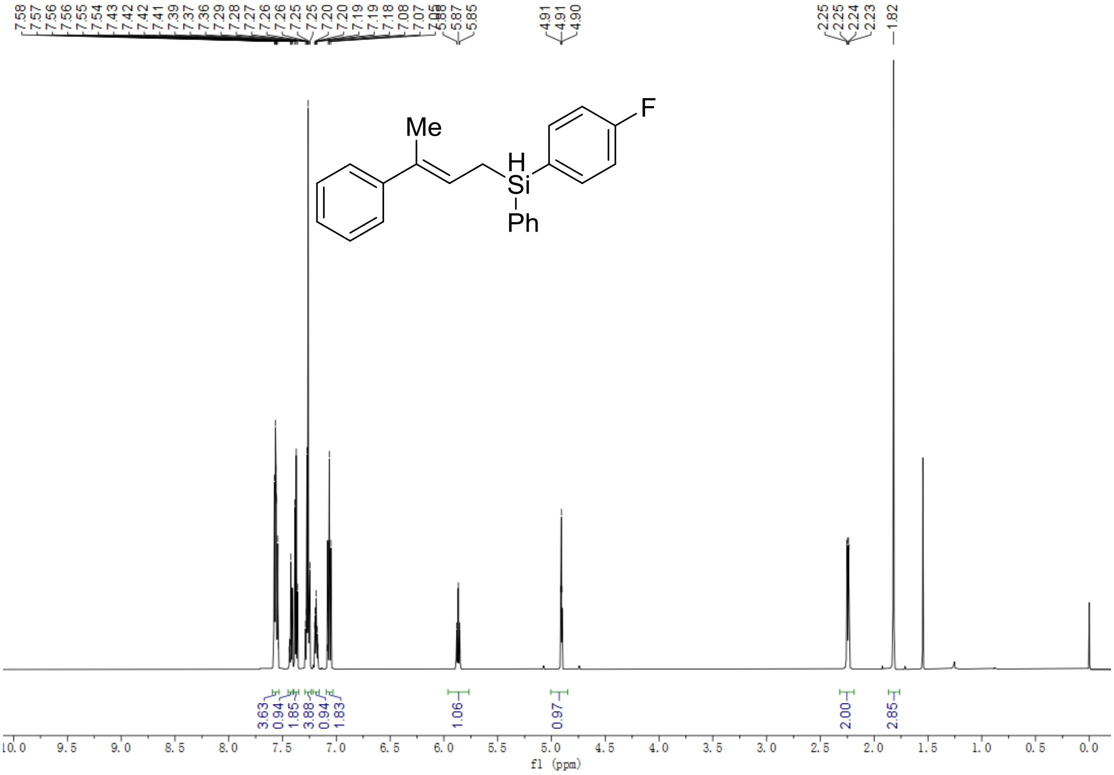

Supplementary Figure 58. ^{1}H NMR (600 MHz, $CDCl_3$, 25 ºC) spectra of **3ab**.

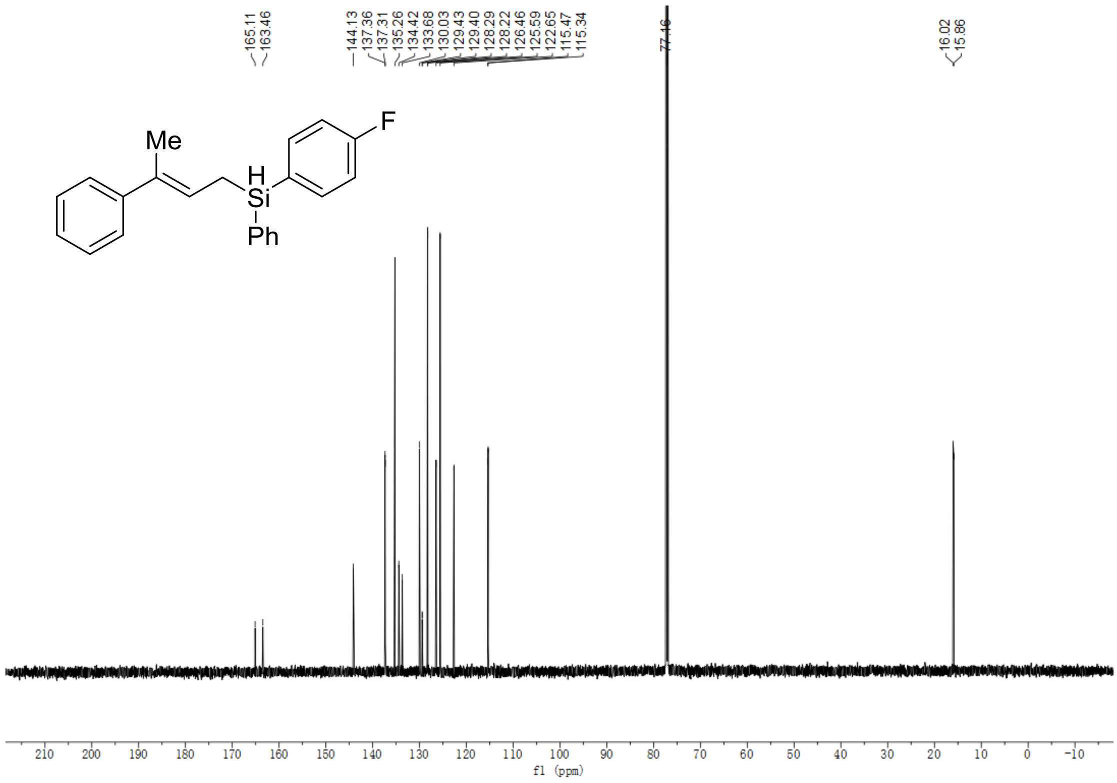

Supplementary Figure 59. ^{13}C NMR (151 MHz, $CDCl_3$, 25 ºC) spectra of **3ab**.

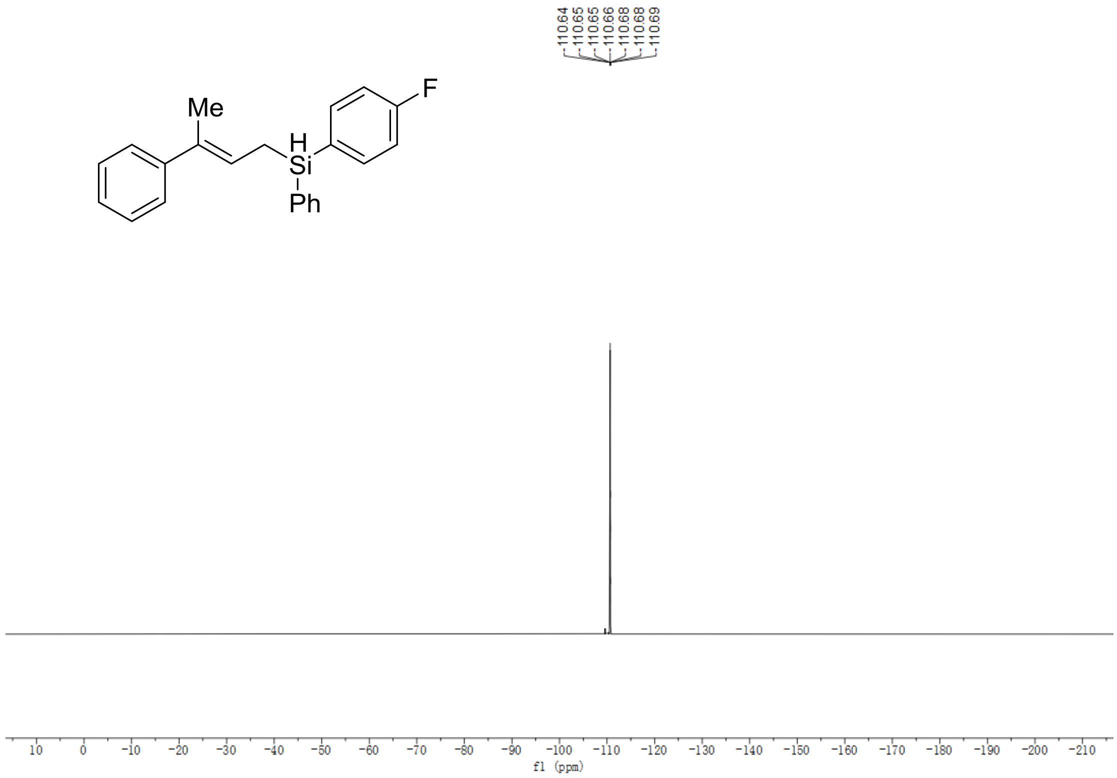

Supplementary Figure 60. ^{19}F NMR (565 MHz, CDCl3, 25 ºC) spectra of **3ab**.

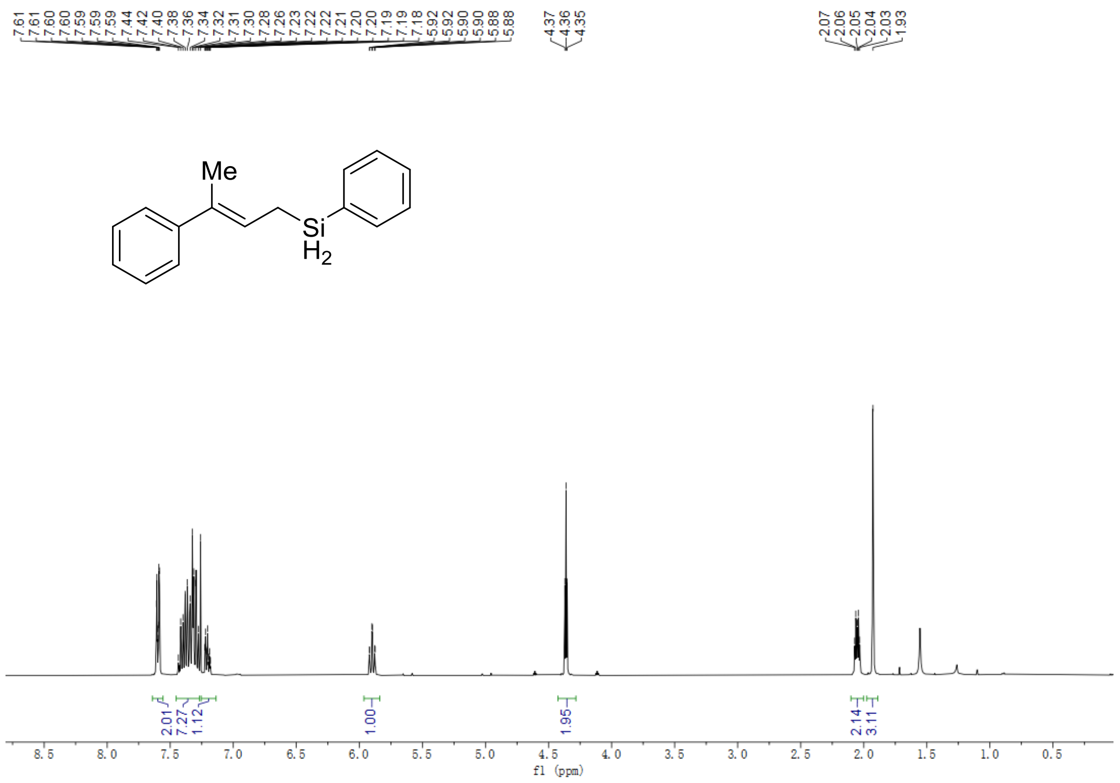

Supplementary Figure 61. ^{1}H NMR (400 MHz, $CDCl_3$, 25 ºC) spectra of **3ac**.

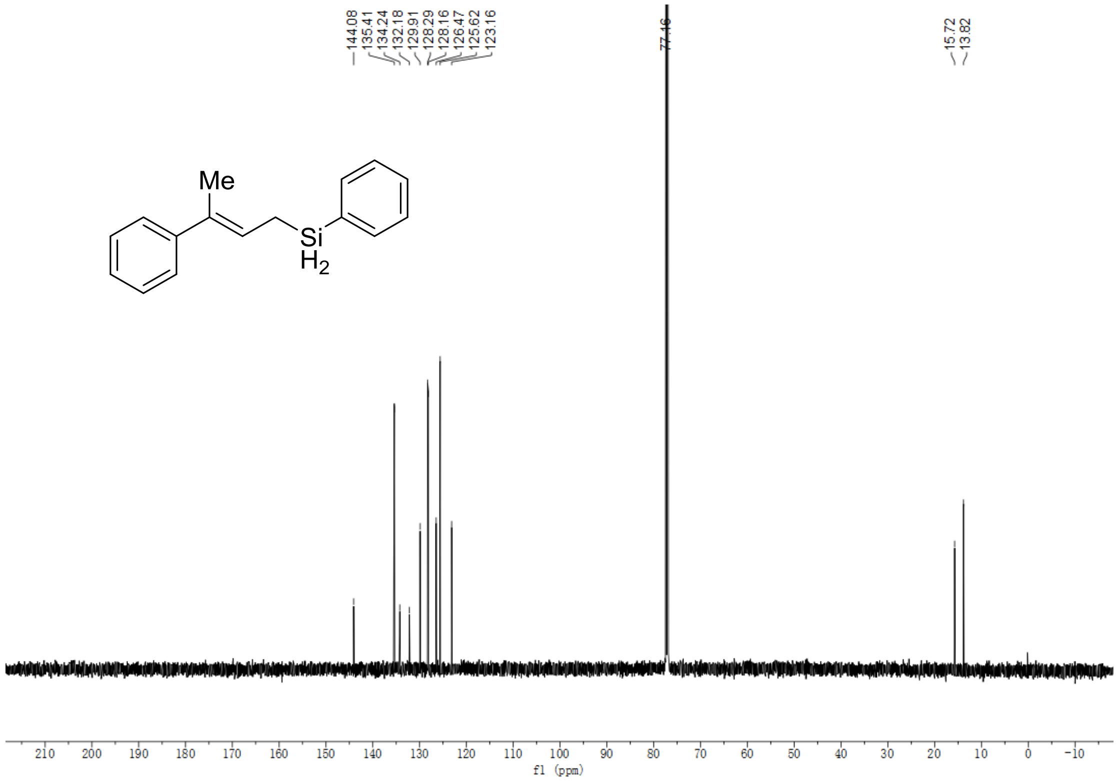

Supplementary Figure 62. ^{13}C NMR (151 MHz, $CDCl_3$, 25 ºC) spectra of **3ac**.

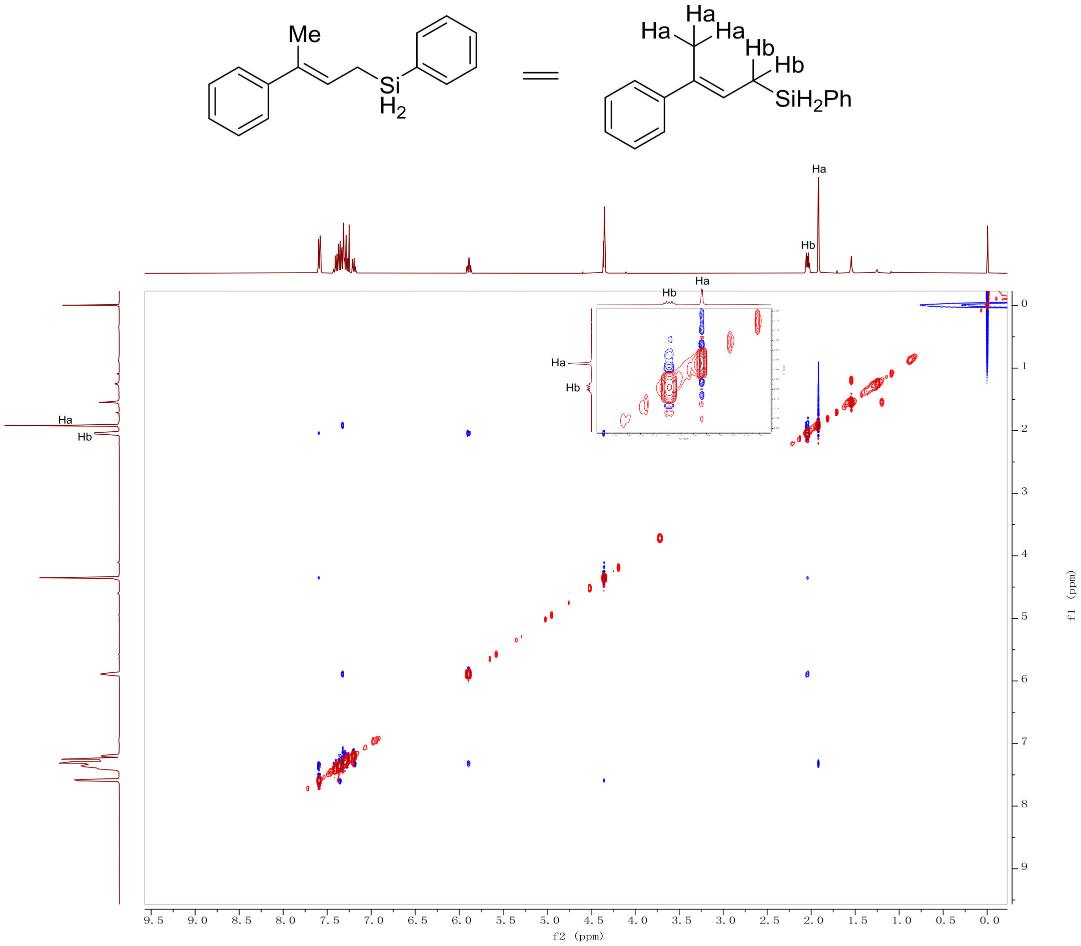

Supplementary Figure 63. ^{1}H-^{1}H NOESY spectra of **3ac**.

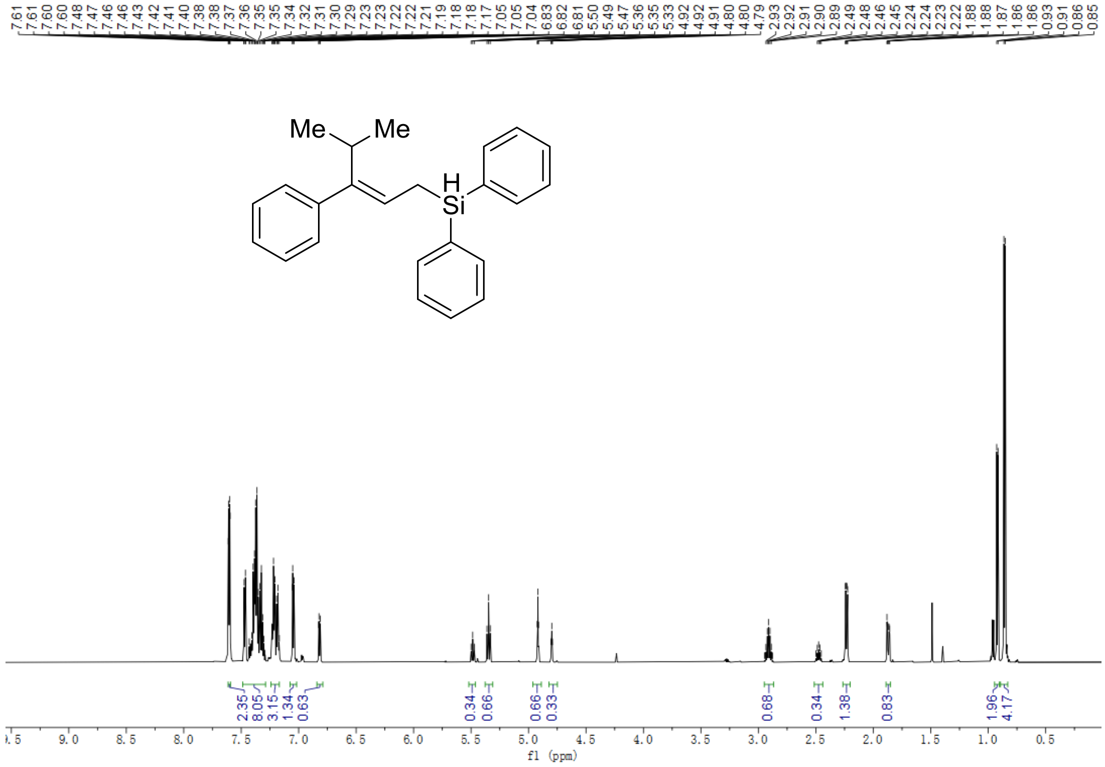

Supplementary Figure 64. ^{1}H NMR (600 MHz, $CDCl_3$, 25 ºC) spectra of **3ad**.

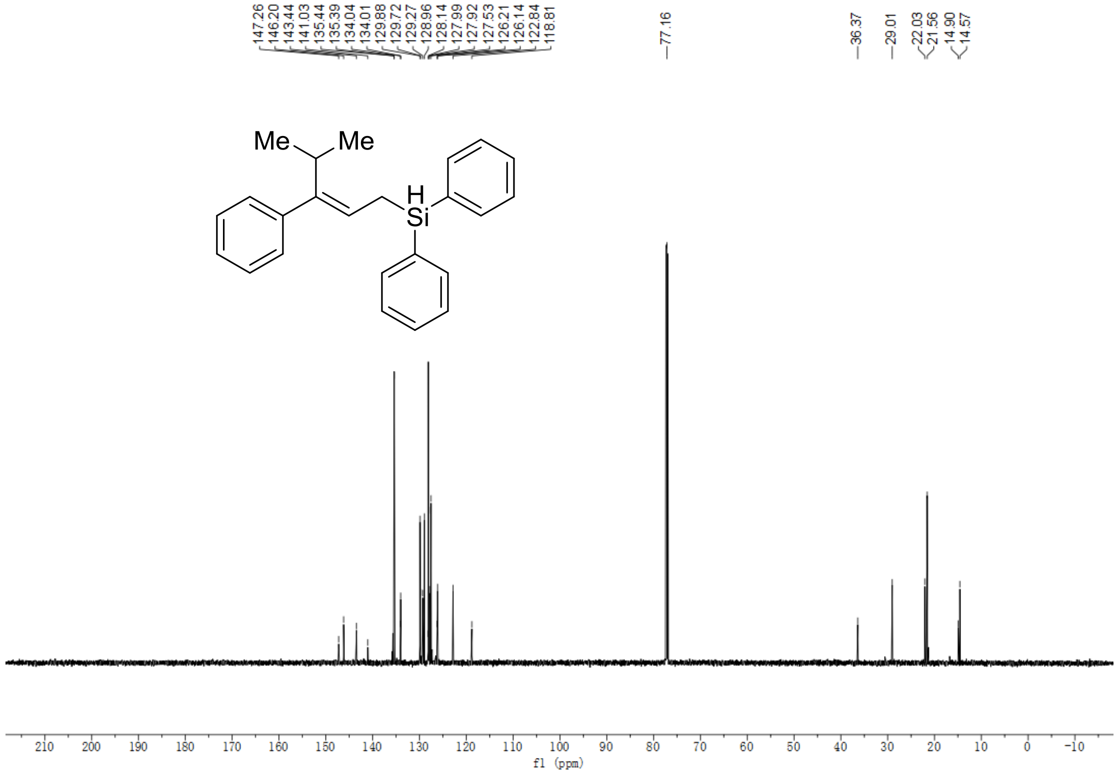

Supplementary Figure 65. ^{13}C NMR (151 MHz, $CDCl_3$, 25 ºC) spectra of **3ad**.

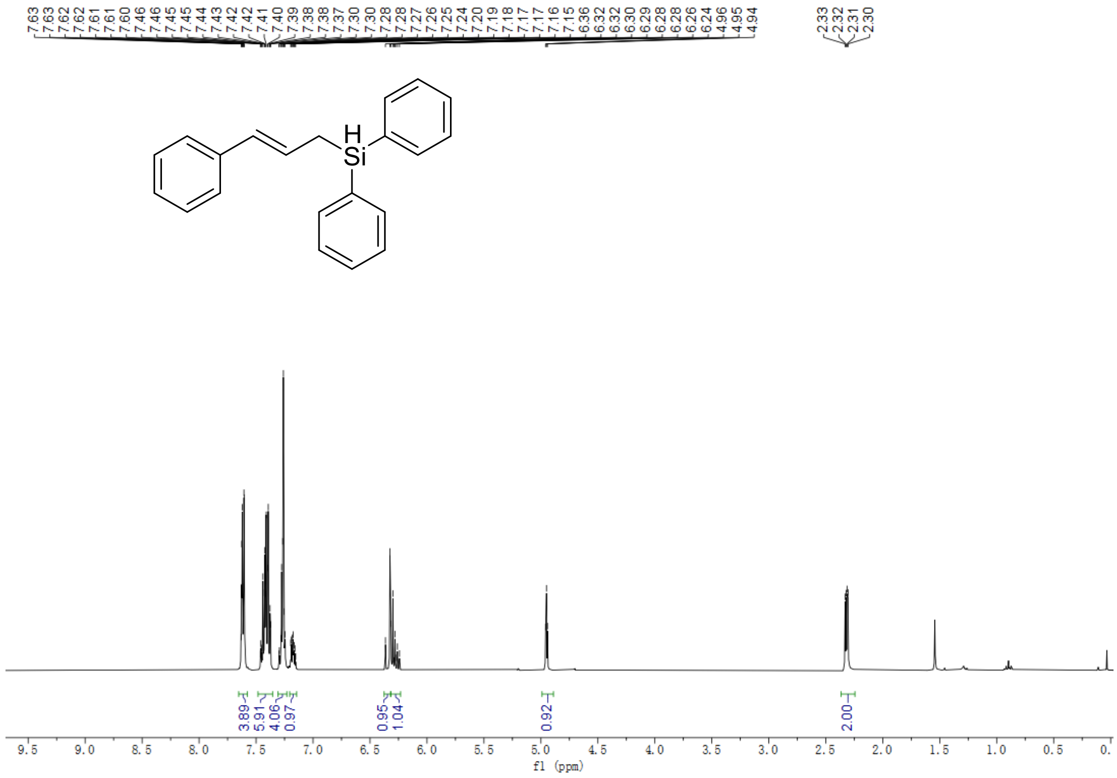

Supplementary Figure 66. ^{1}H NMR (600 MHz, $CDCl_3$, 25 ºC) spectra of **5a**.

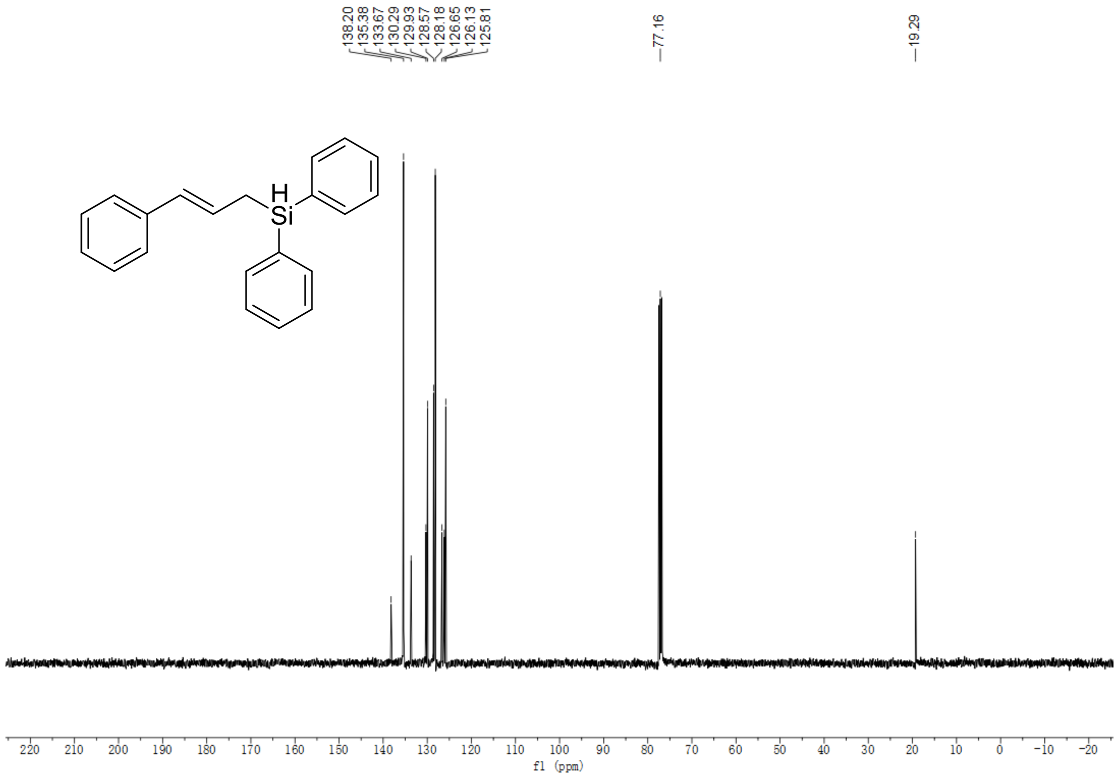

Supplementary Figure 67. ^{13}C NMR (151 MHz, $CDCl_3$, 25 ºC) spectra of **5a**.

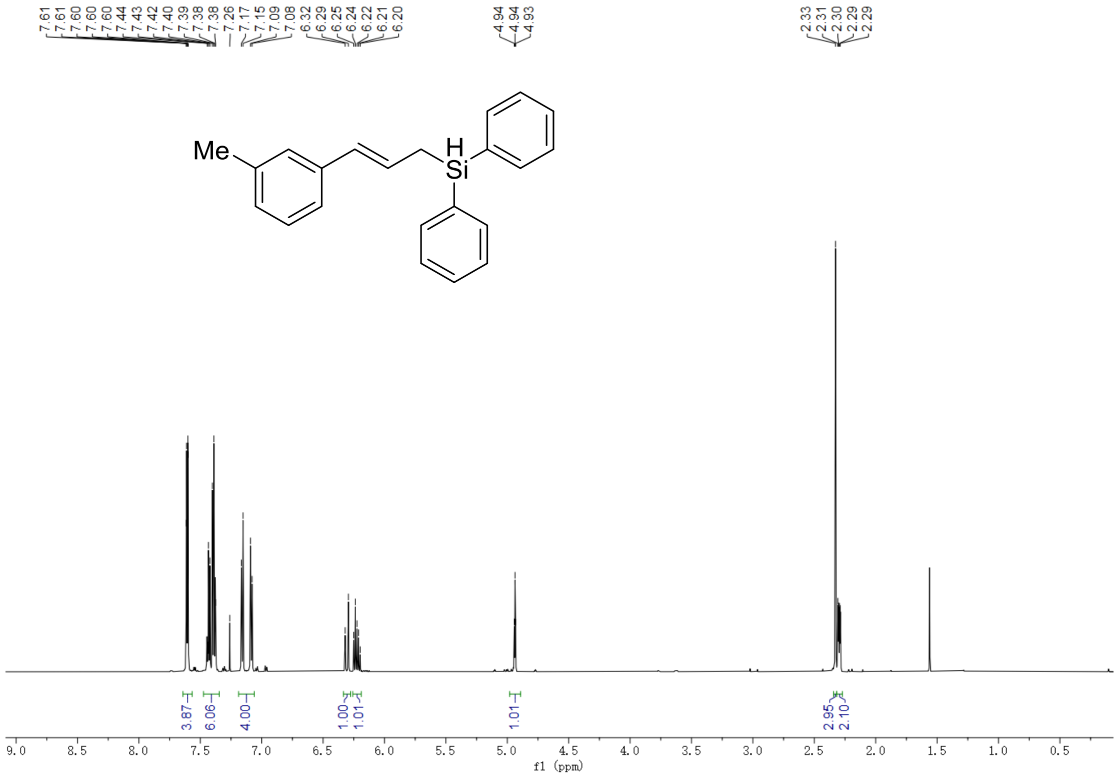

Supplementary Figure 68. ^{1}H NMR (600 MHz, $CDCl_3$, 25 ºC) spectra of **5b**.

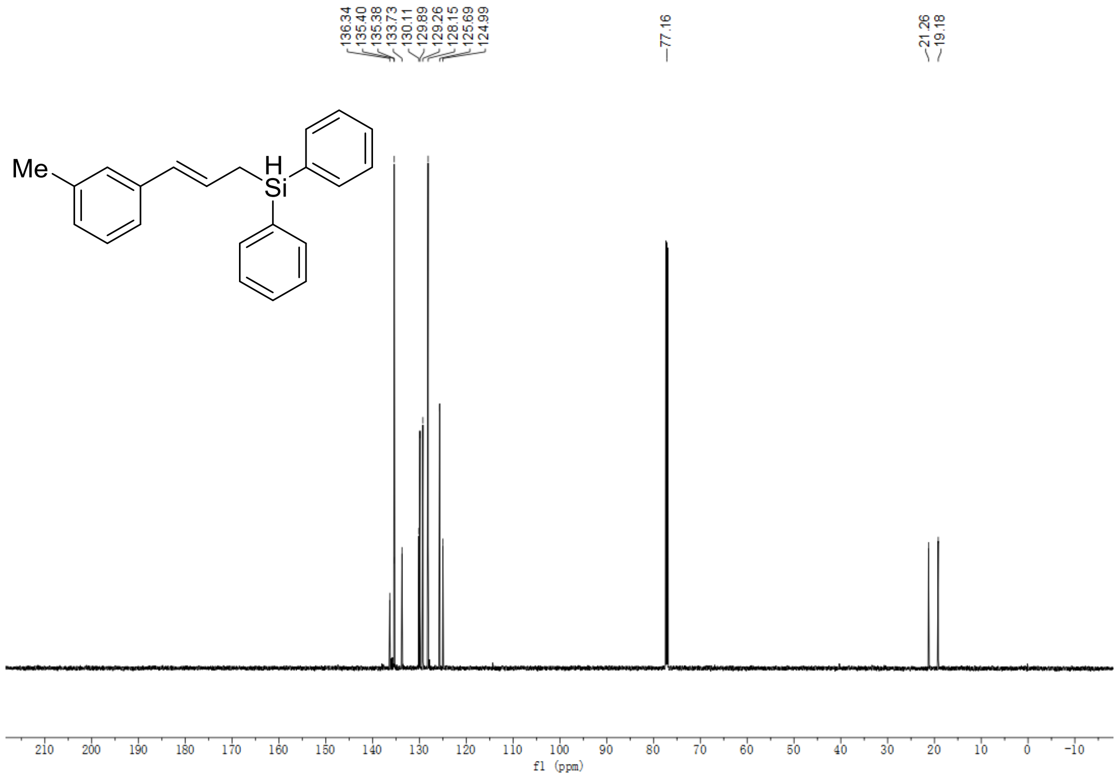

Supplementary Figure 69. ^{13}C NMR (151 MHz, $CDCl_3$, 25 ºC) spectra of **5b**.

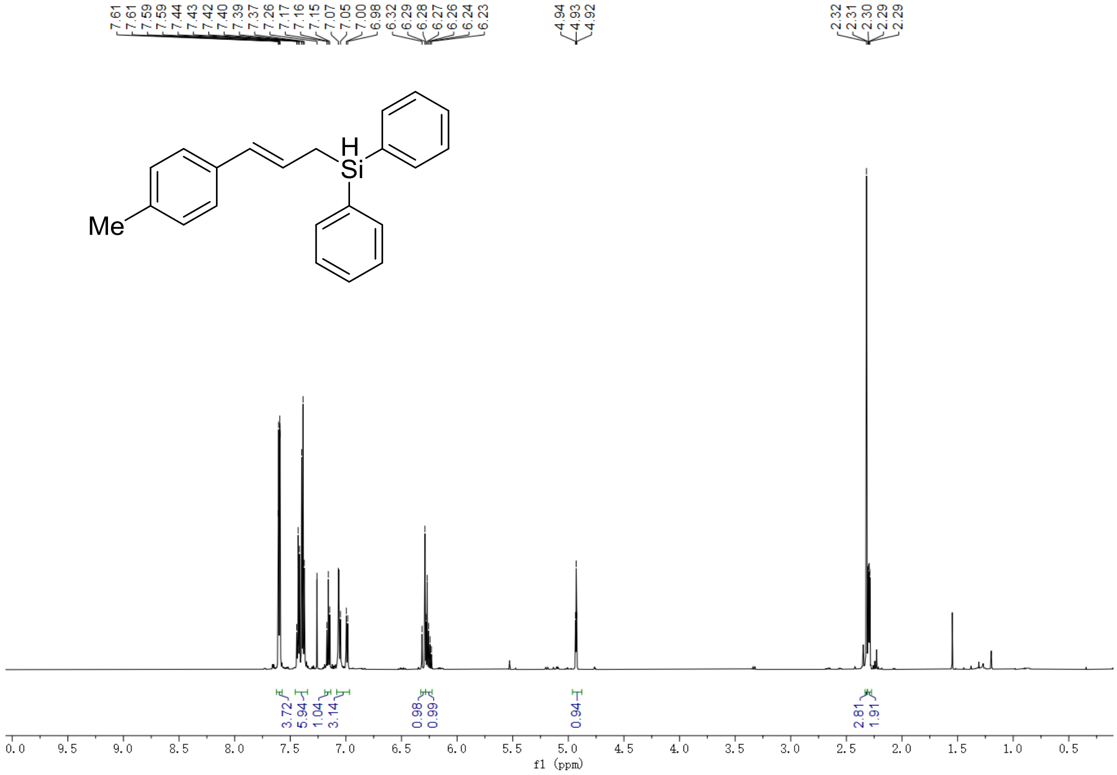

Supplementary Figure 70. ^{1}H NMR (600 MHz, $CDCl_3$, 25 ºC) spectra of **5c**.

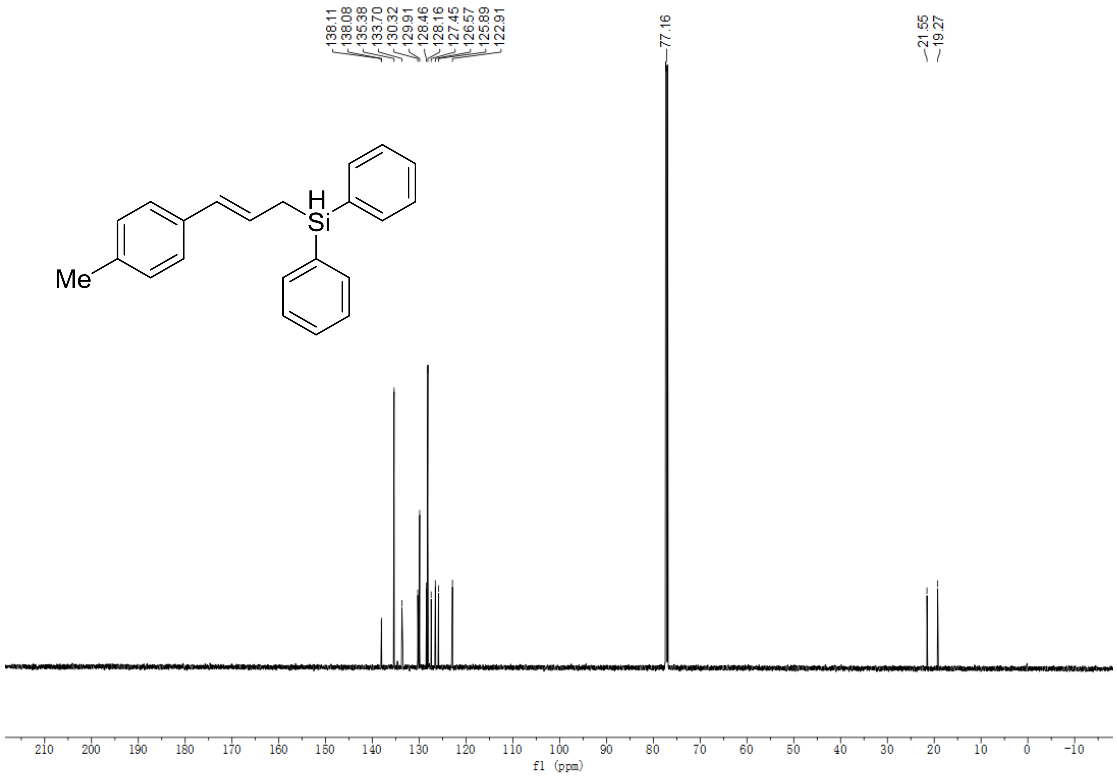

Supplementary Figure 71. ^{13}C NMR (151 MHz, $CDCl_3$, 25 ºC) spectra of **5c**.

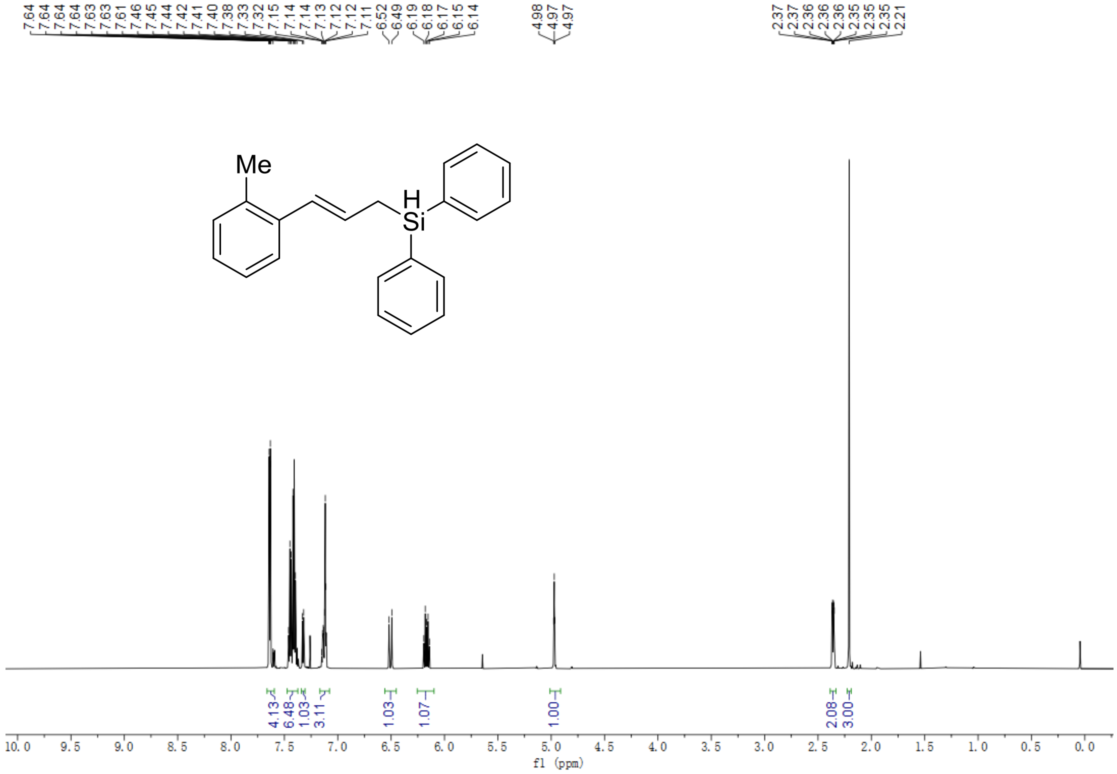

Supplementary Figure 72. ^{1}H NMR (600 MHz, $CDCl_3$, 25 ºC) spectra of **5d**.

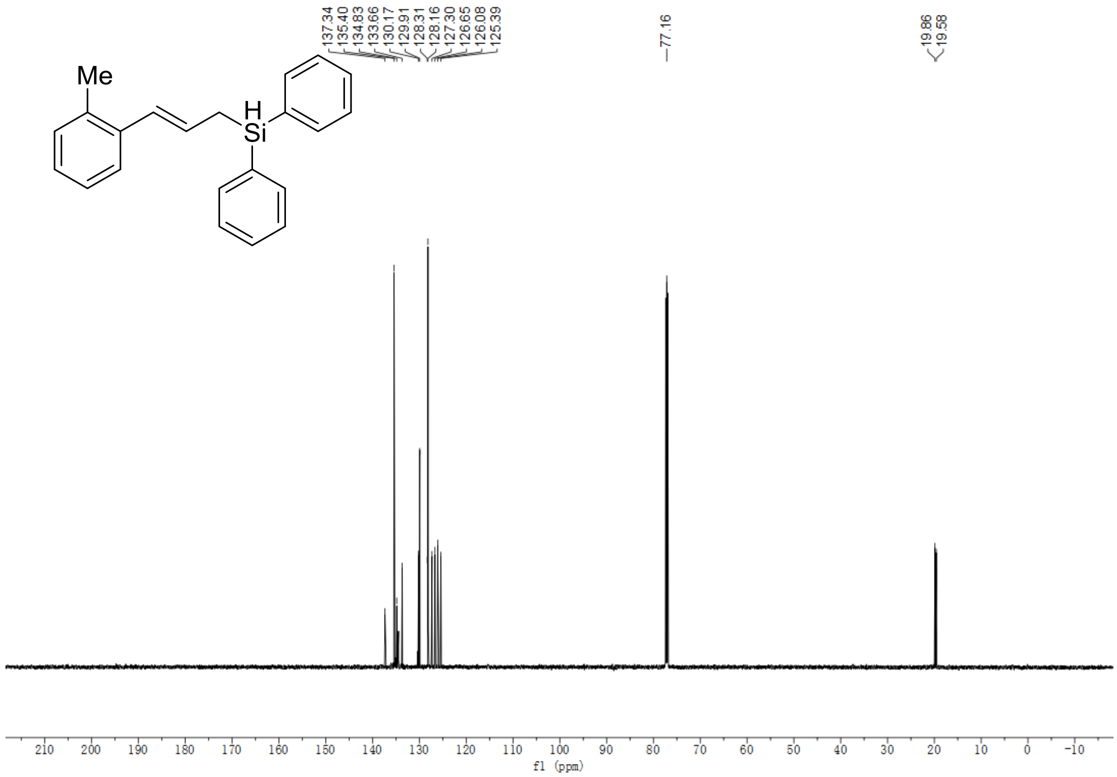

Supplementary Figure 73. ^{13}C NMR (151 MHz, $CDCl_3$, 25 °C) spectra of **5d**.

^{1}H NMR (600 MHz, $CDCl_3$, 25 °C) of **5e**

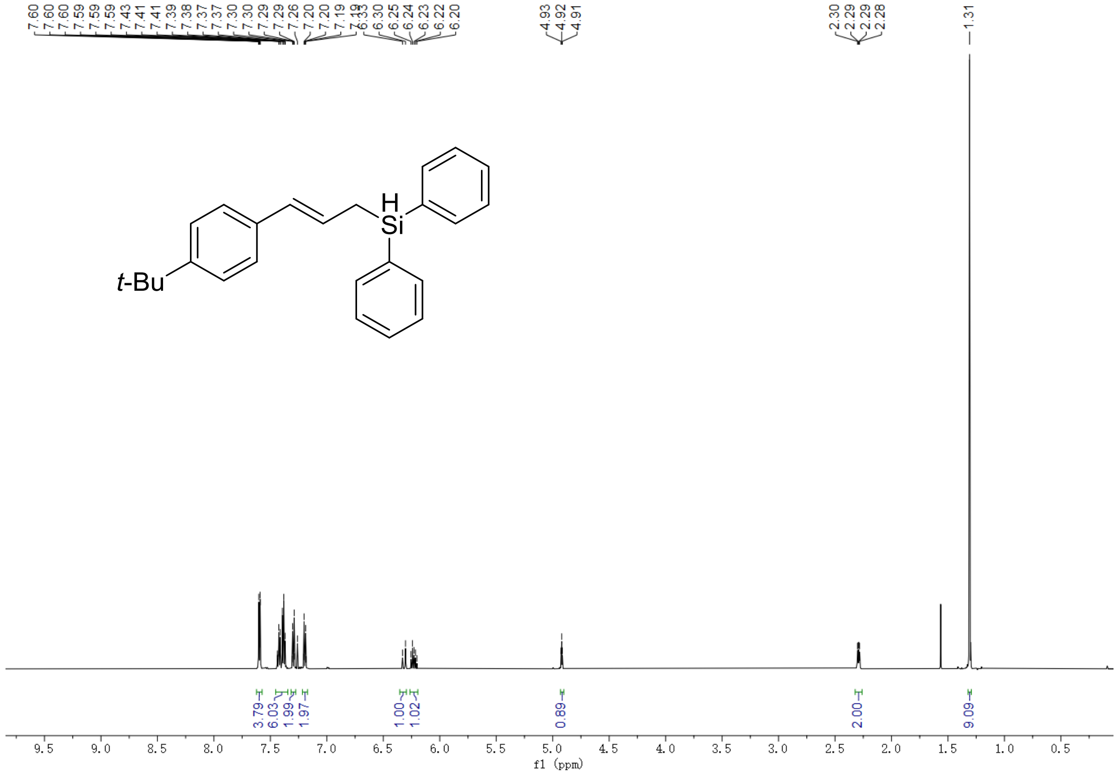

Supplementary Figure 74. ^{1}H NMR spectra of **5e**.

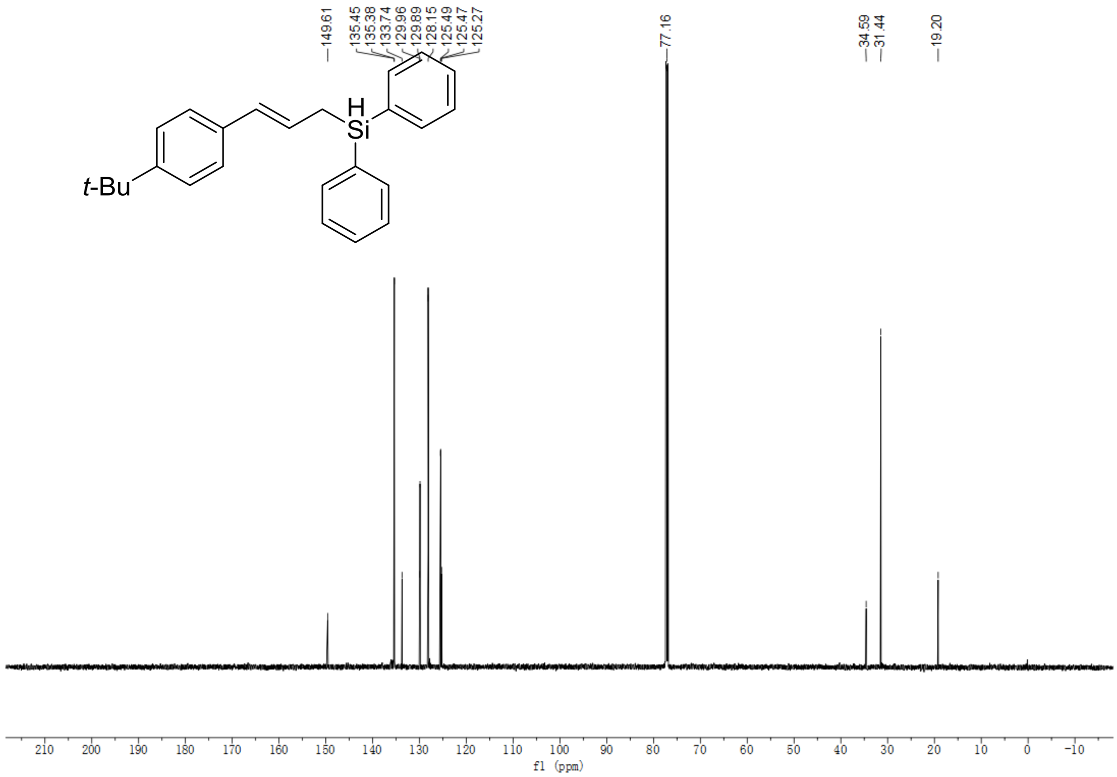

Supplementary Figure 75. ^{13}C NMR (151 MHz, $CDCl_3$, 25 ºC) spectra of **5e**.

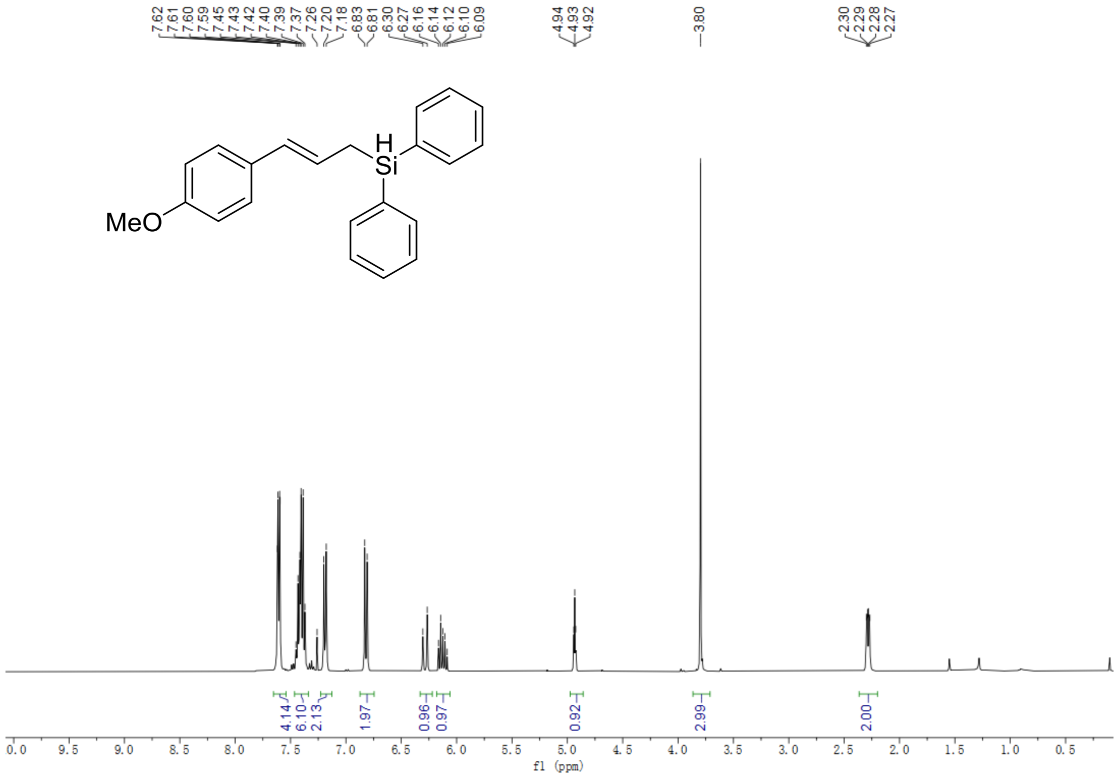

Supplementary Figure 76. ^{1}H NMR (600 MHz, $CDCl_3$, 25 ºC) spectra of **5f**.

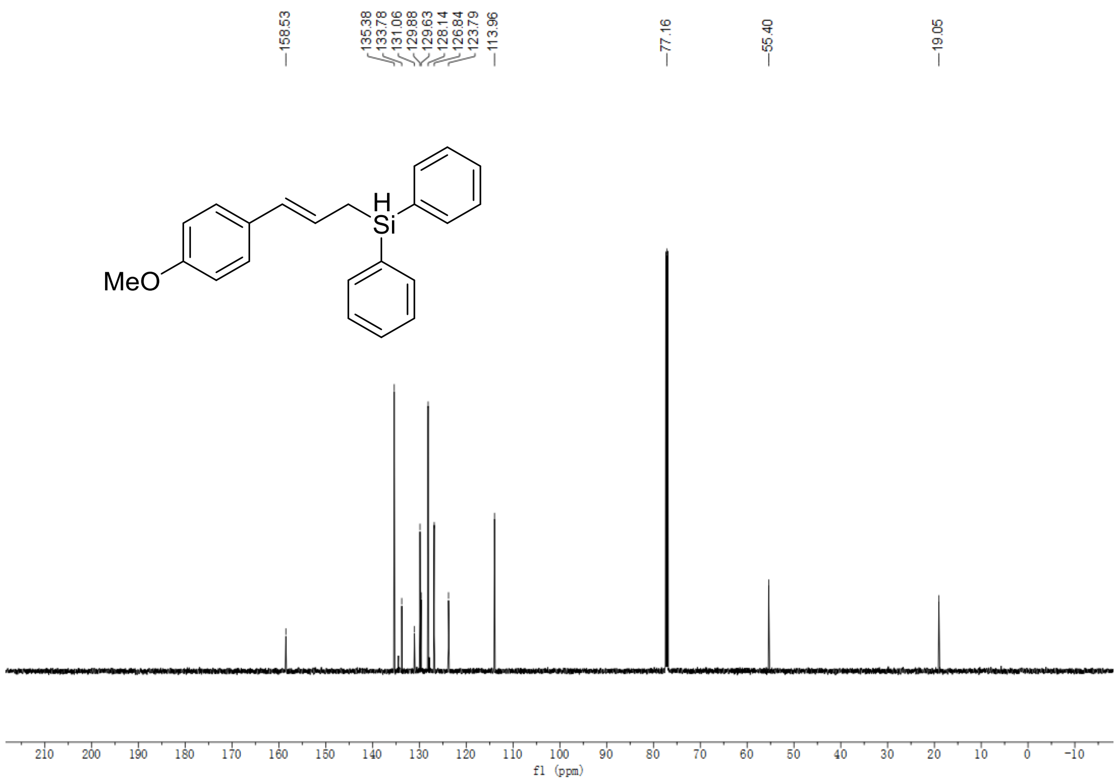

Supplementary Figure 77. ^{13}C NMR (151 MHz, $CDCl_3$, 25 ºC) spectra of **5f**.

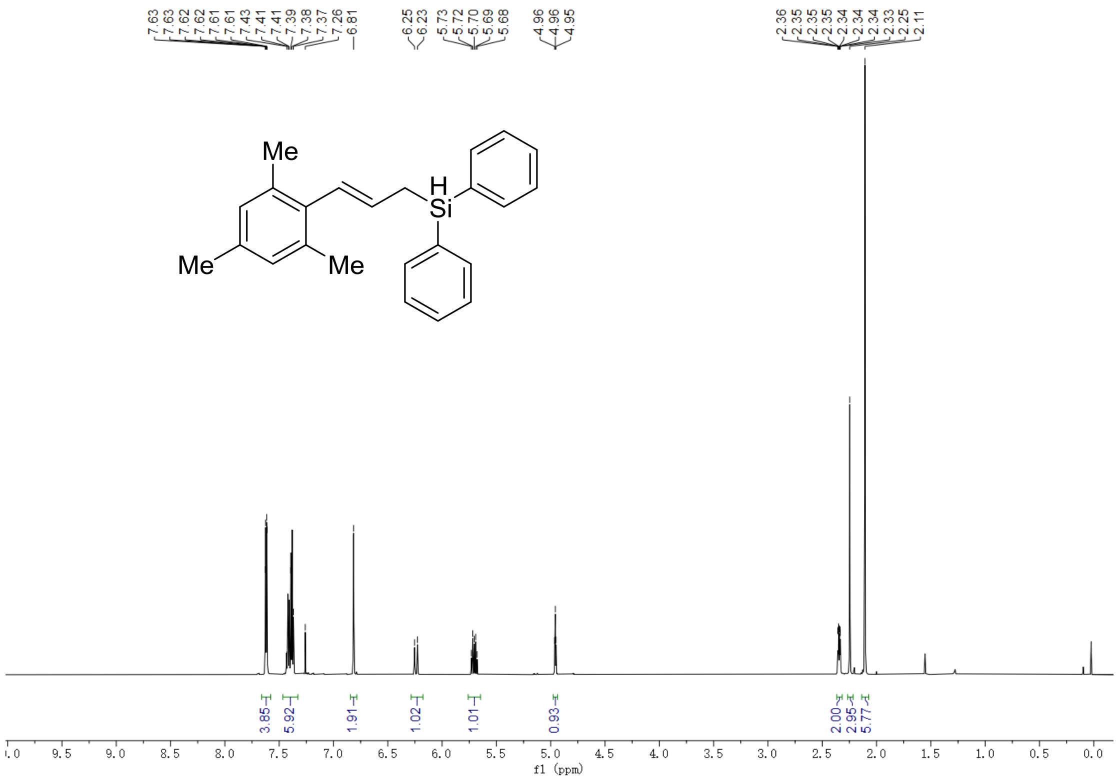

Supplementary Figure 78. ^{1}H NMR (600 MHz, $CDCl_3$, 25 ºC) spectra of **5g**.

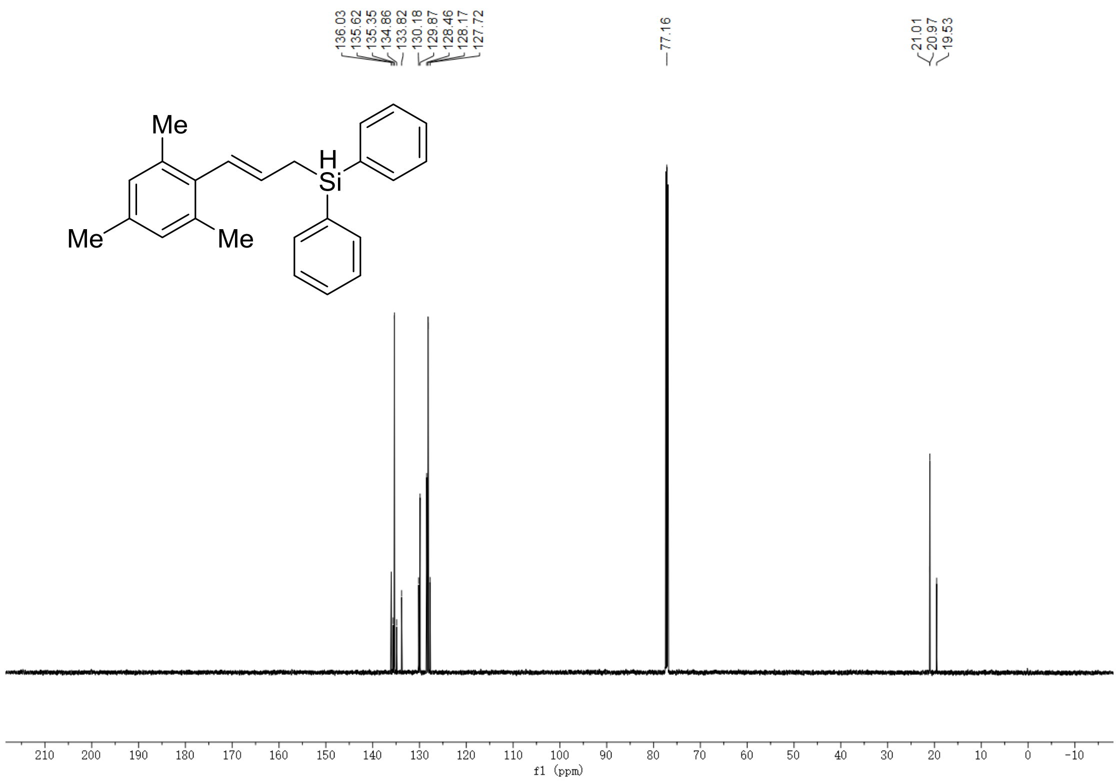

Supplementary Figure 79. ^{13}C NMR (151 MHz, $CDCl_3$, 25 ºC) spectra of **5g**.

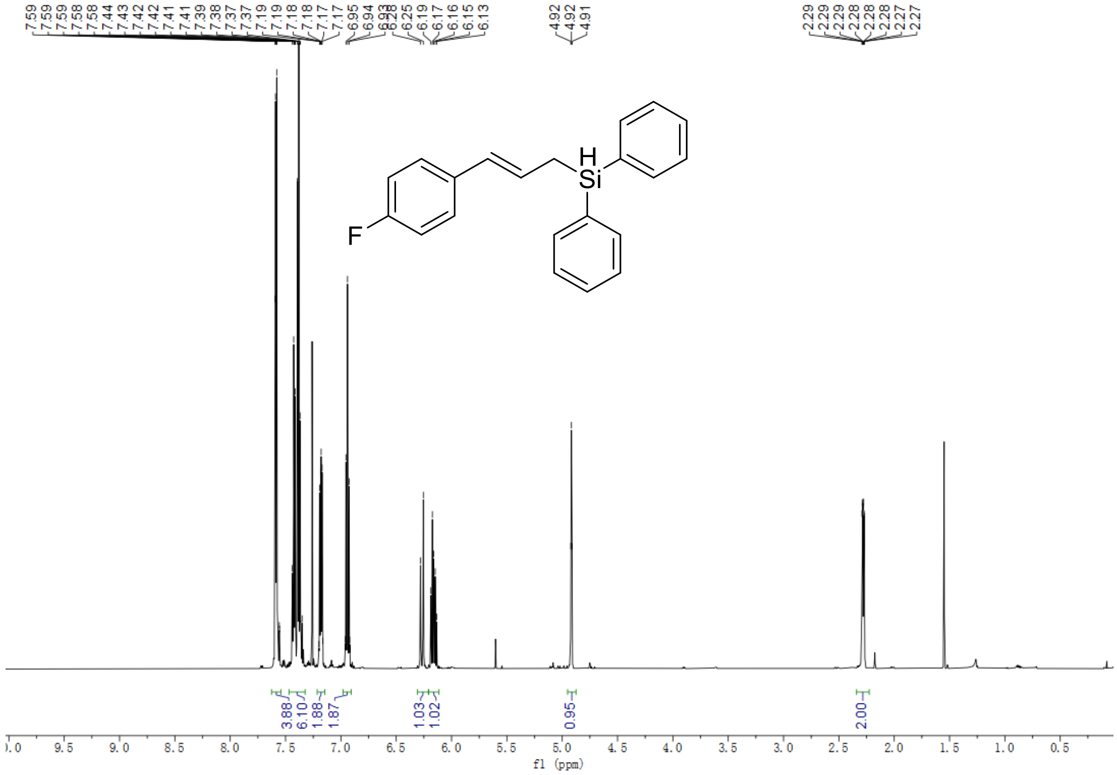

Supplementary Figure 80. ^{1}H NMR (600 MHz, $CDCl_3$, 25 ºC) spectra of **5h**.

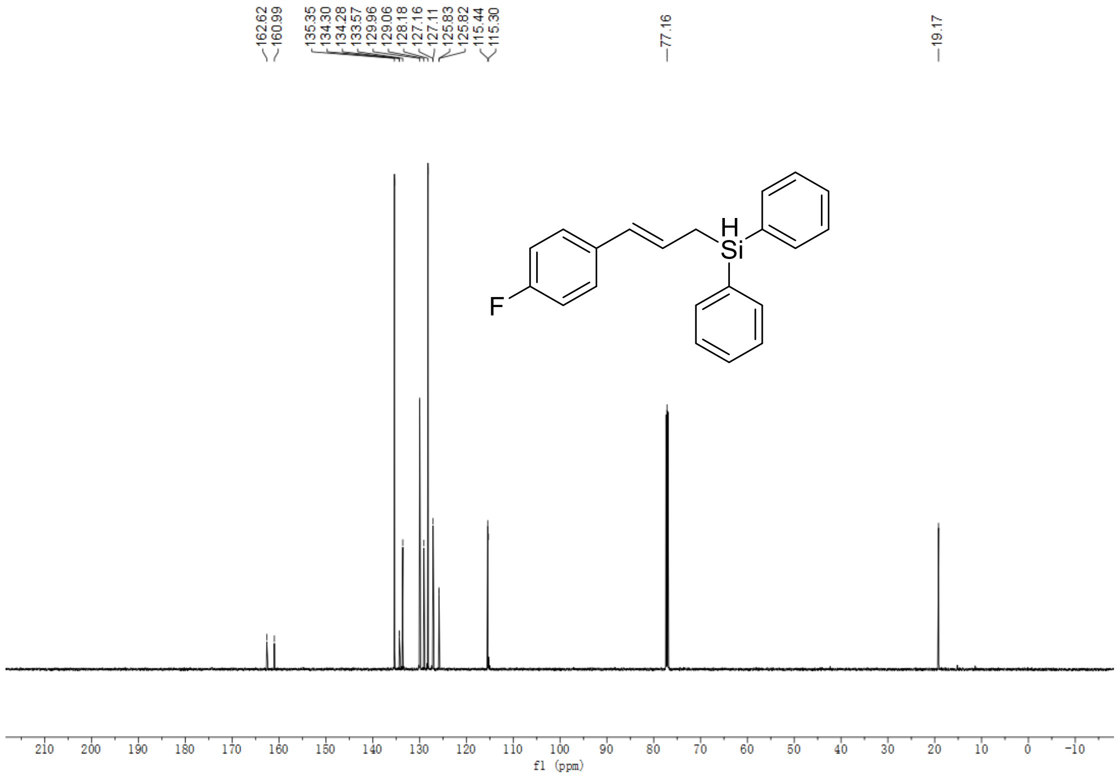

Supplementary Figure 81. ^{13}C NMR (151 MHz, $CDCl_3$, 25 ºC) spectra of **5h**.

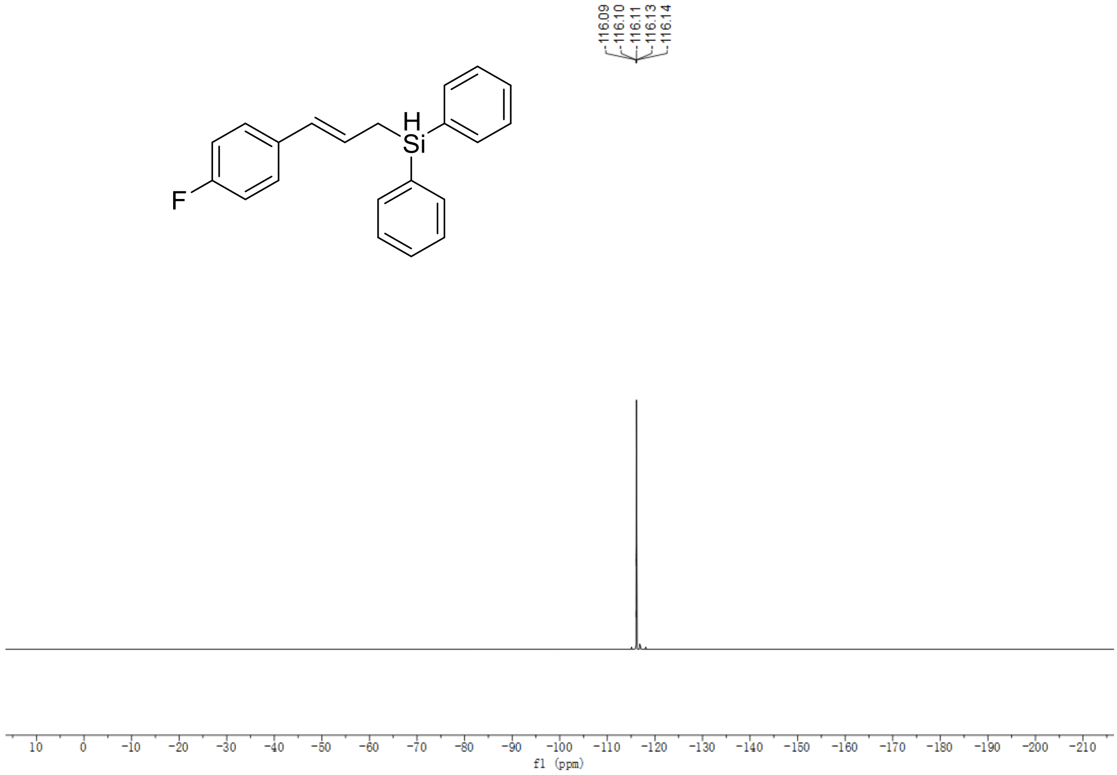

Supplementary Figure 82. ^{19}F NMR (565 MHz, $CDCl_3$, 25 ºC) spectra of **5h**.

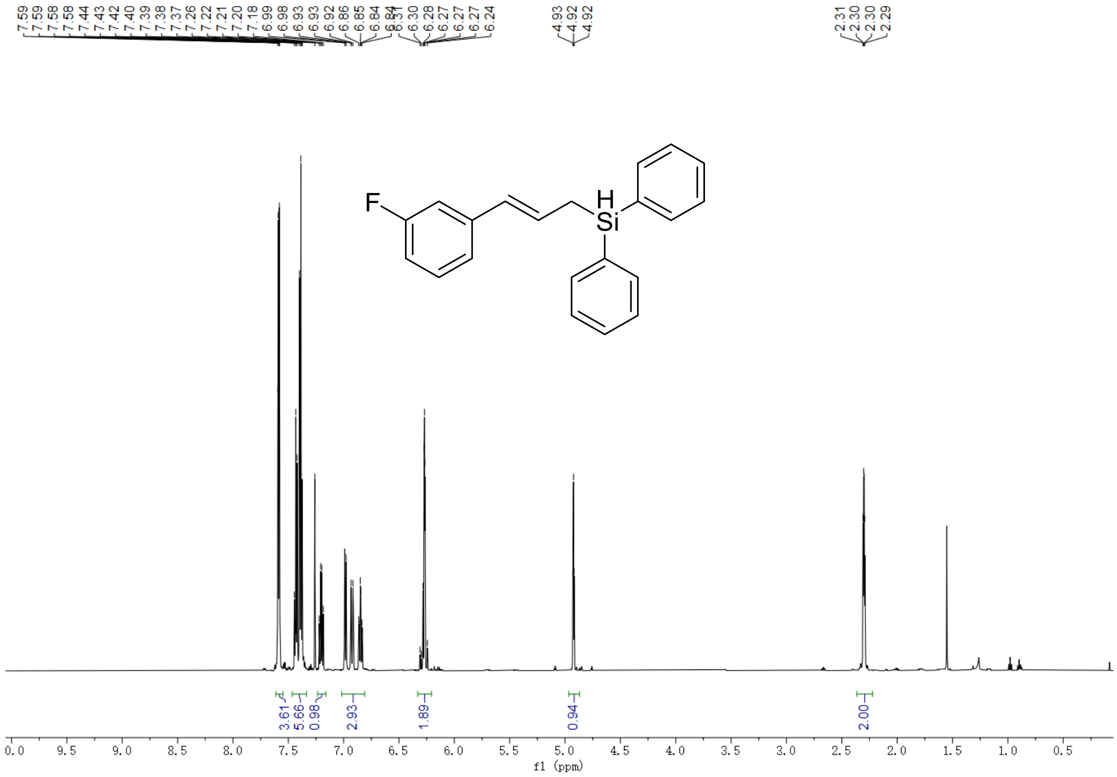

Supplementary Figure 83. ^{1}H NMR (600 MHz, $CDCl_3$, 25 ºC) spectra of **5i**.

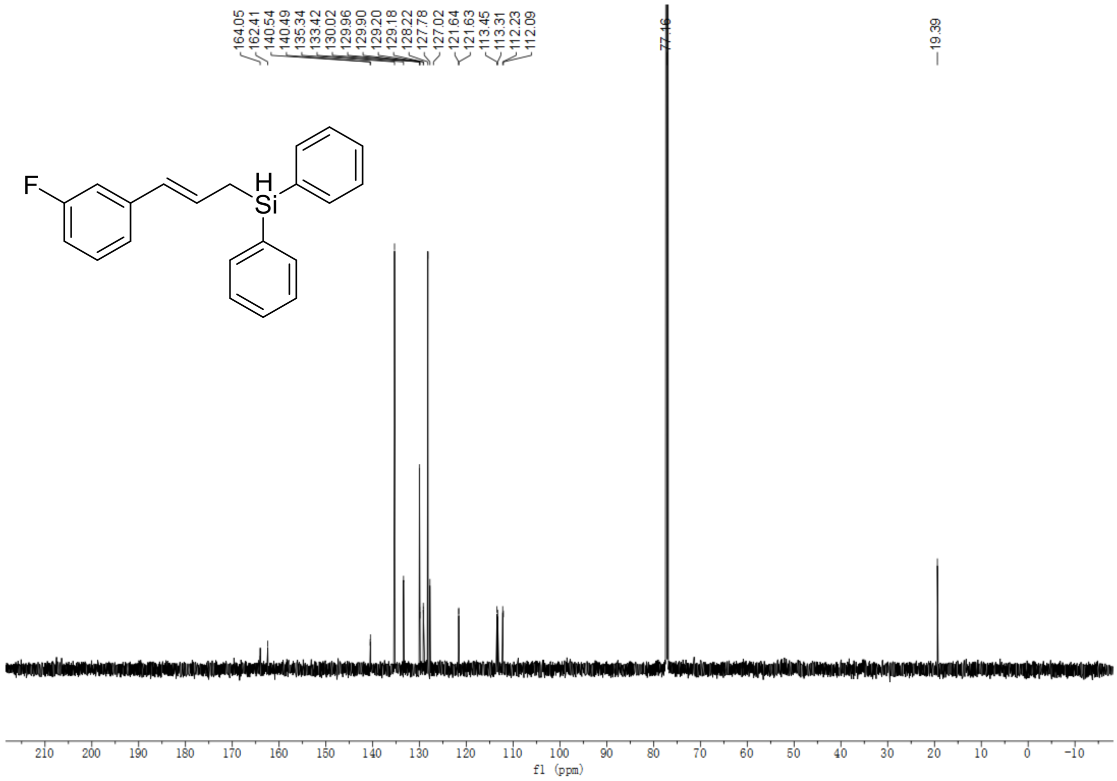

Supplementary Figure 84. ^{13}C NMR (151 MHz, $CDCl_3$, 25 ºC) spectra of **5i**.

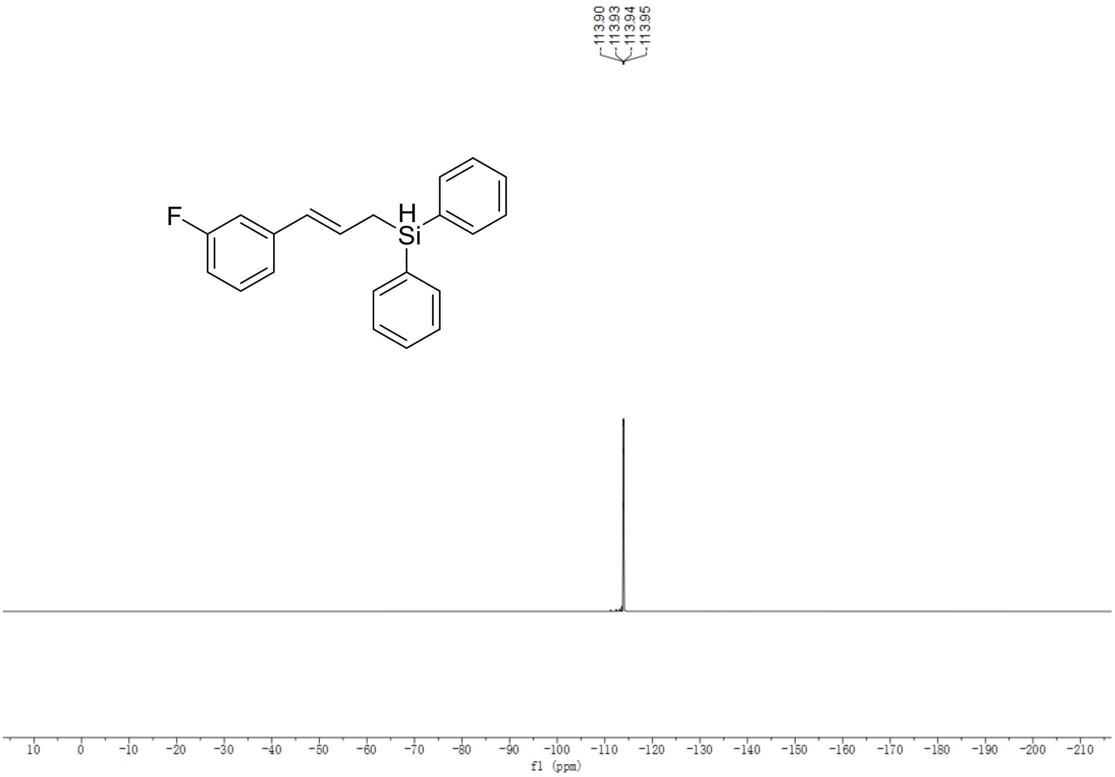

Supplementary Figure 85. ^{19}F NMR (565 MHz, $CDCl_3$, 25 ºC) spectra of **5i**.

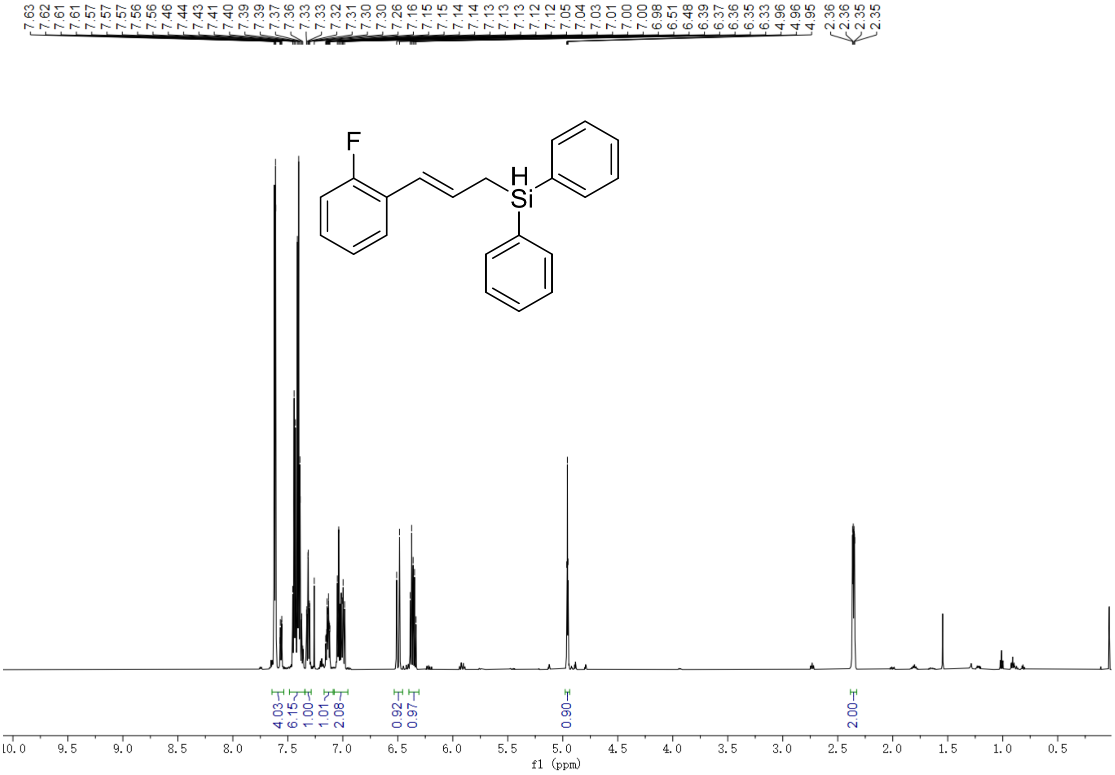

Supplementary Figure 86. ^{1}H NMR (600 MHz, $CDCl_3$, 25 ºC) spectra of **5j**.

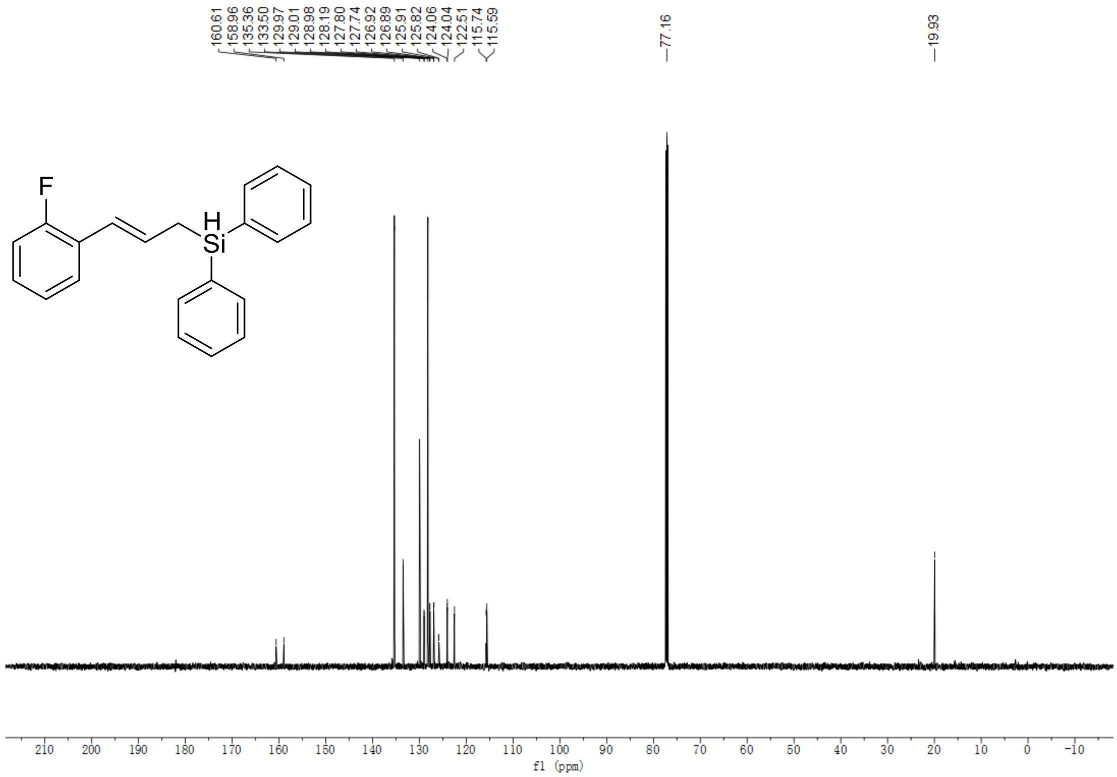

Supplementary Figure 87. ^{13}C NMR (151 MHz, $CDCl_3$, 25 ºC) spectra of **5j**.

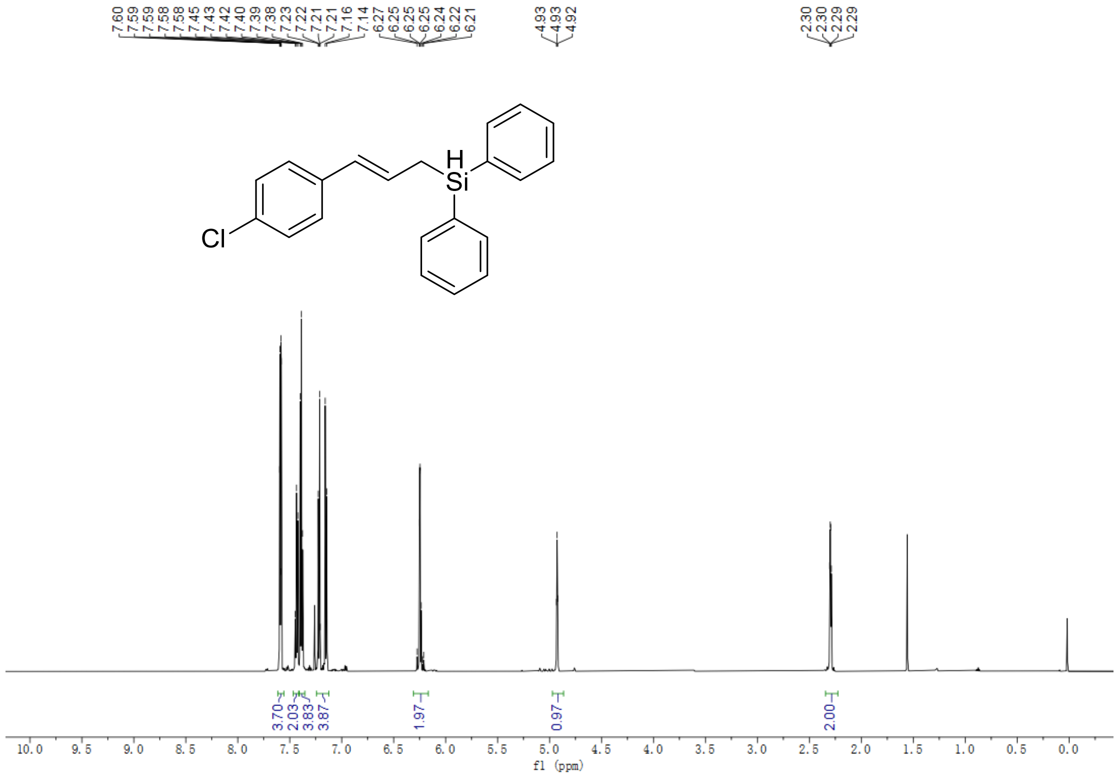

Supplementary Figure 88. ^{1}H NMR (600 MHz, $CDCl_3$, 25 ºC) spectra of **5k**.

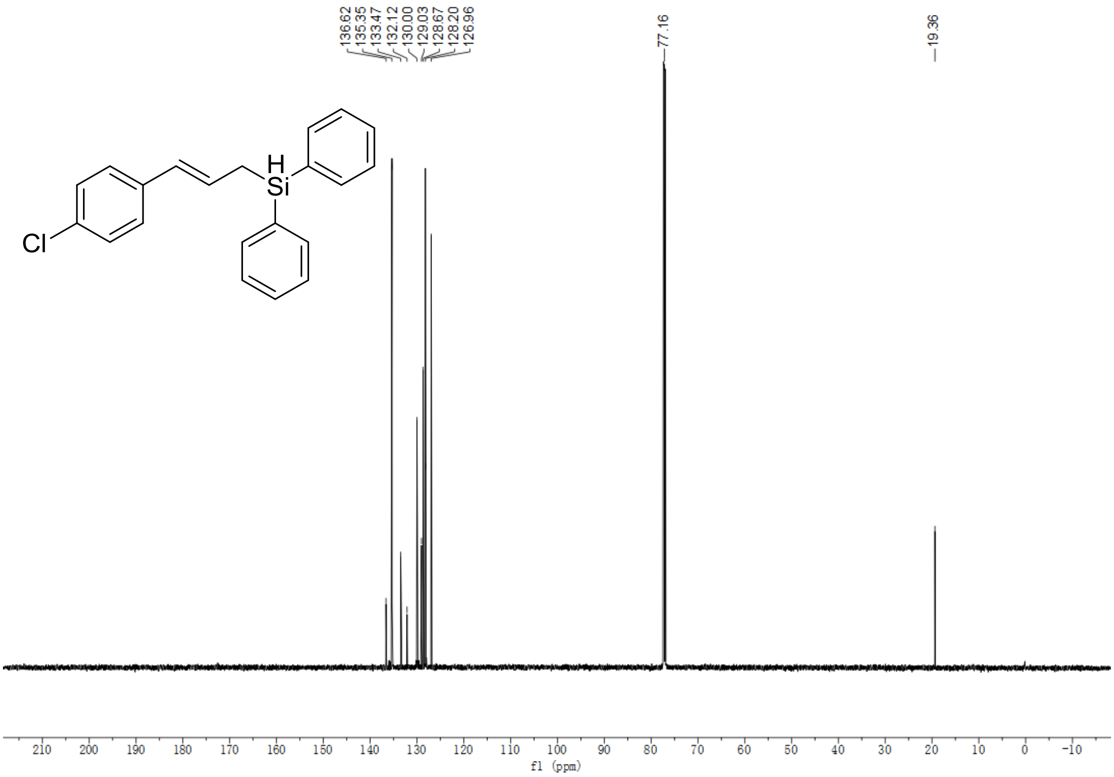

Supplementary Figure 89. ^{13}C NMR (151 MHz, $CDCl_3$, 25 ºC) spectra of **5k**.

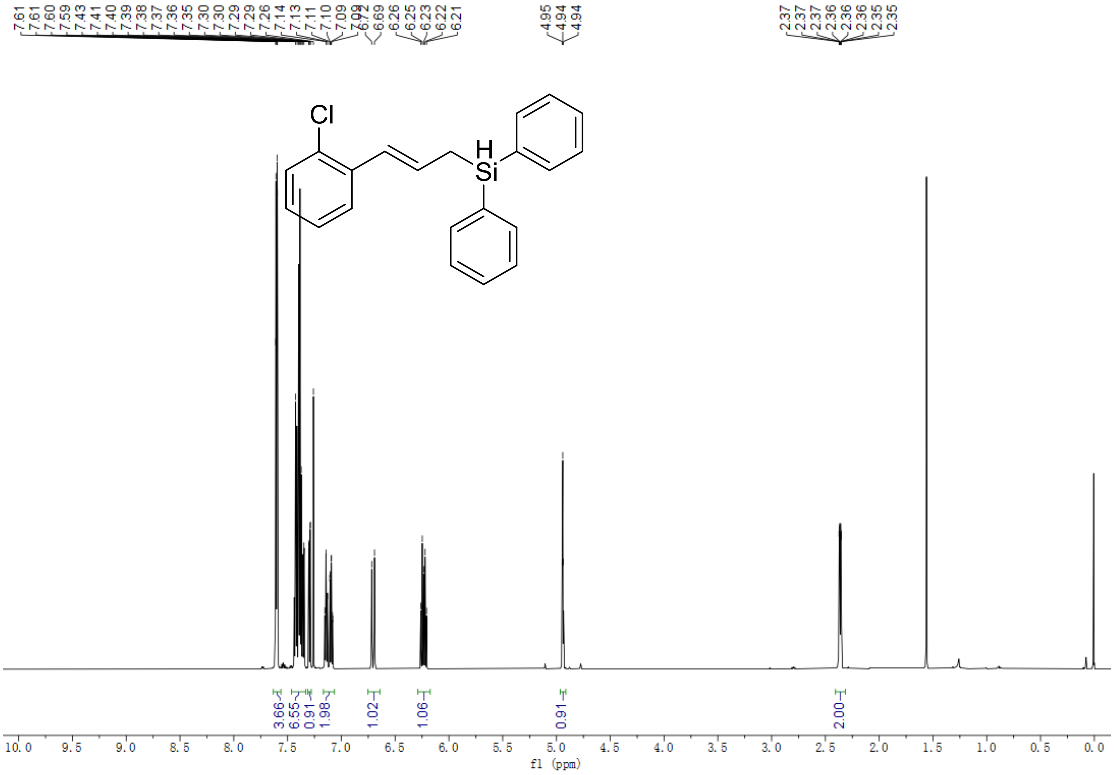

Supplementary Figure 90. ^{1}H NMR (600 MHz, $CDCl_3$, 25 ºC) spectra of **5l**.

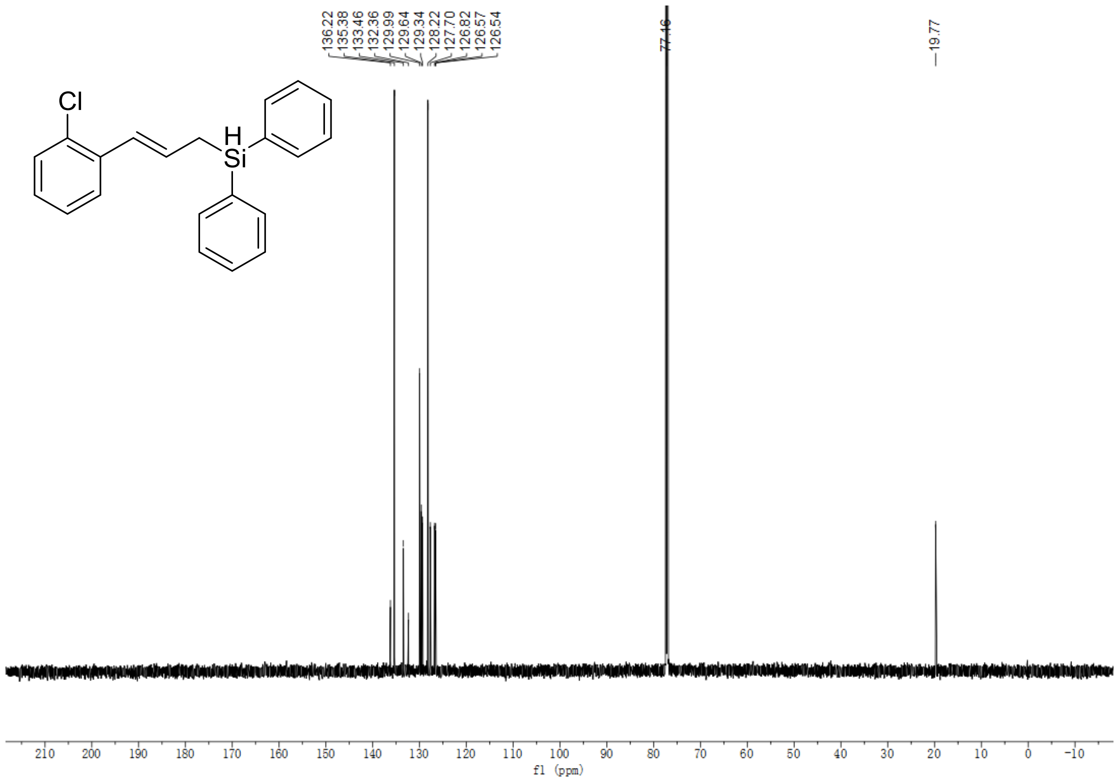

Supplementary Figure 91. ^{13}C NMR (151 MHz, $CDCl_3$, 25 ºC) spectra of **5l**.

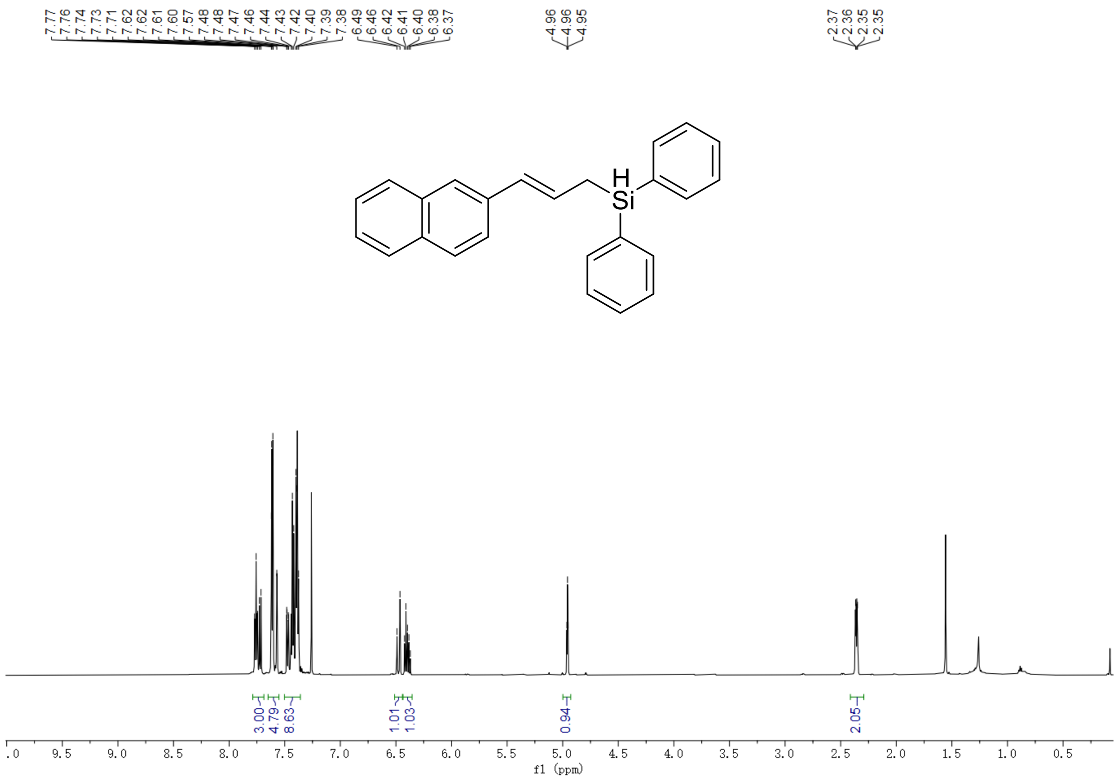

Supplementary Figure 92. ^{1}H NMR (600 MHz, $CDCl_3$, 25 ºC) spectra of **5m**.

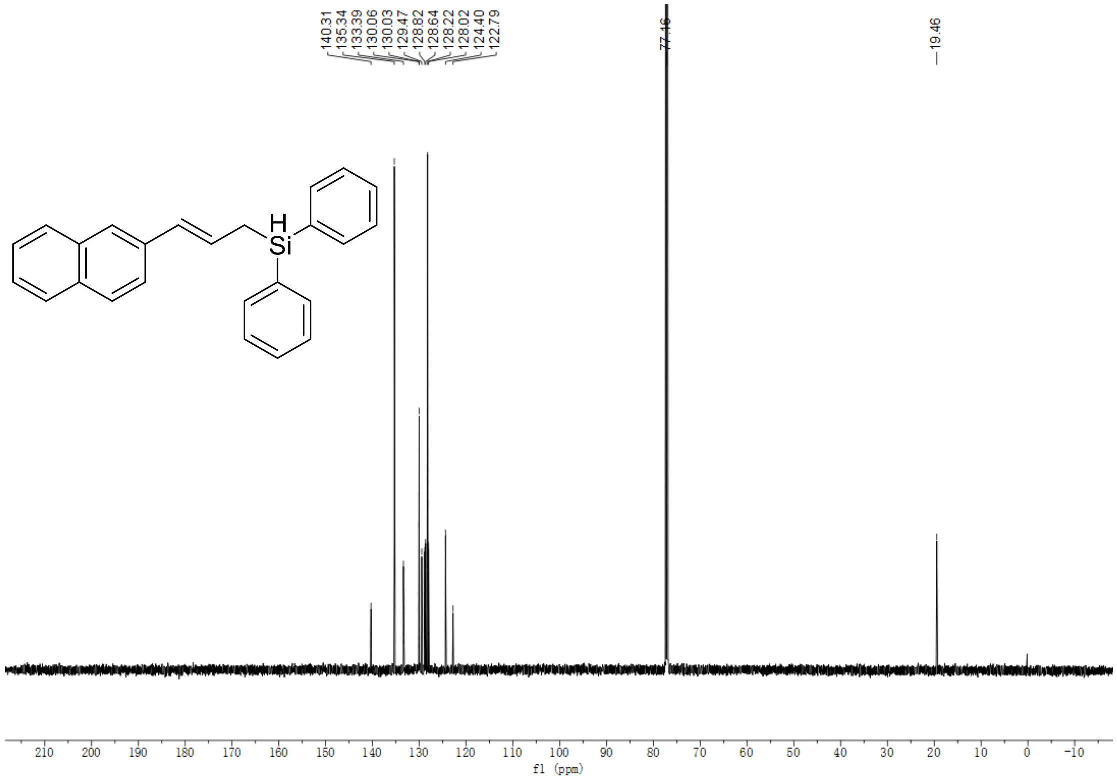

Supplementary Figure 93. ^{13}C NMR (151 MHz, $CDCl_3$, 25 ºC) spectra of **5m**.

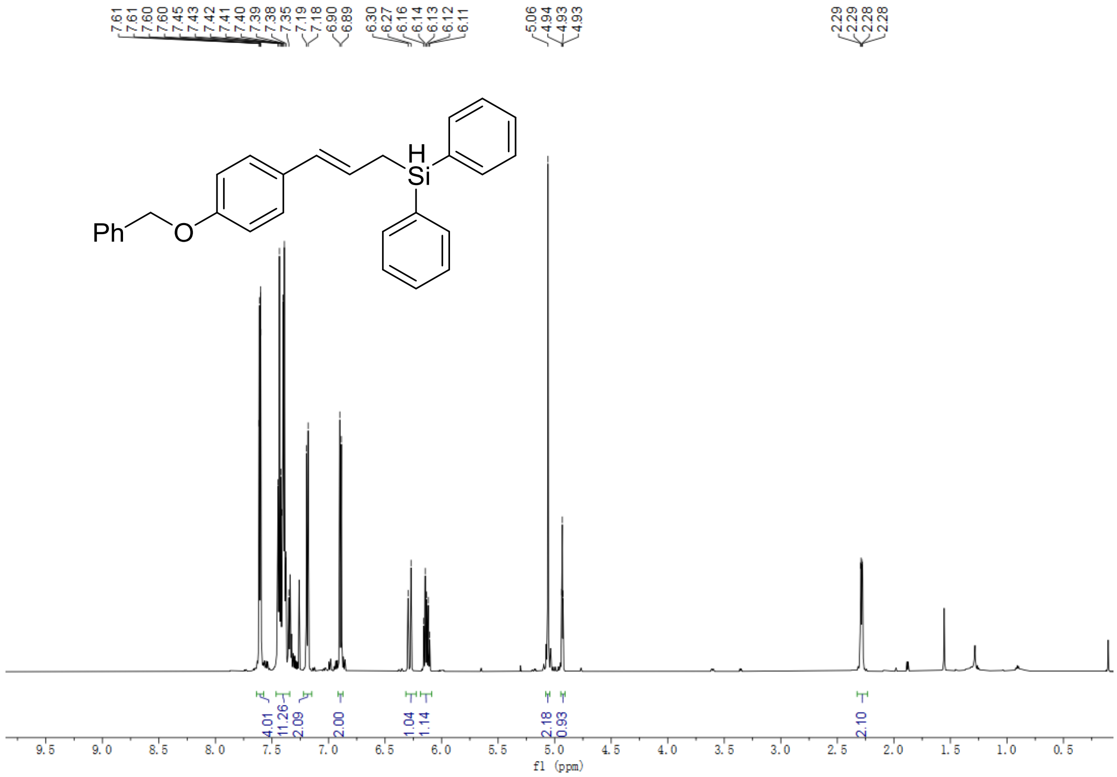

Supplementary Figure 94. ^{1}H NMR (600 MHz, $CDCl_3$, 25 ºC) spectra of **5o**.

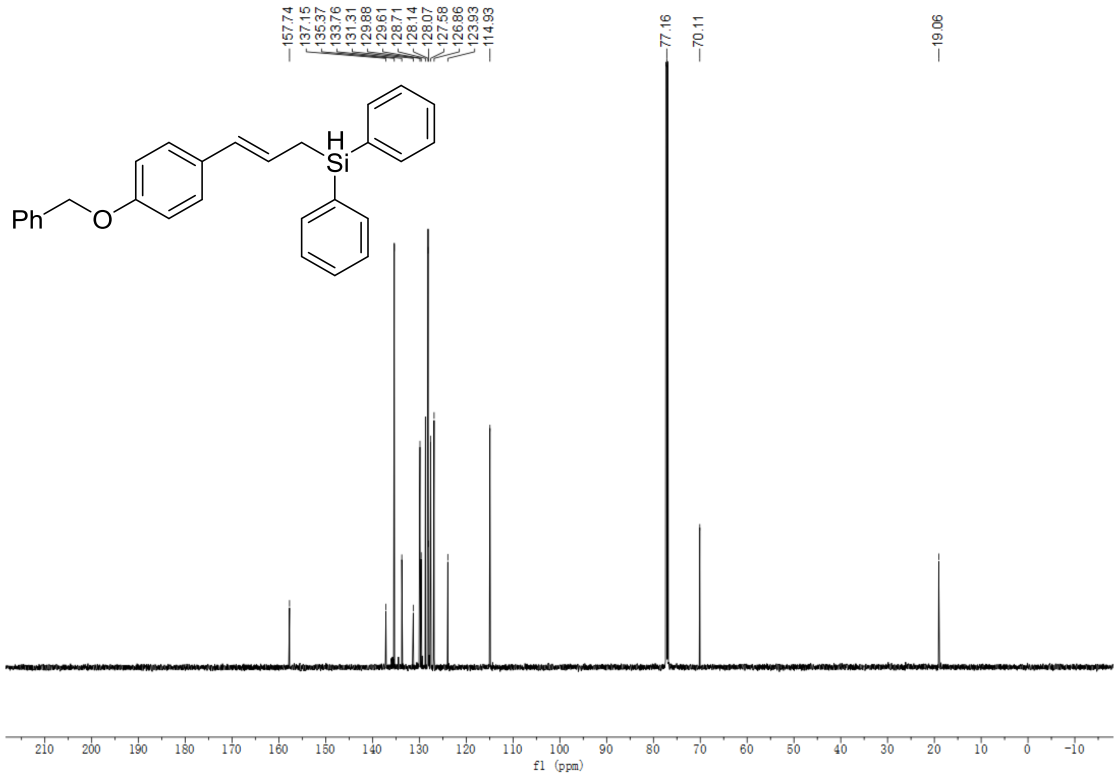

Supplementary Figure 95. ^{13}C NMR (151 MHz, $CDCl_3$, 25 ºC) spectra of **5o**.

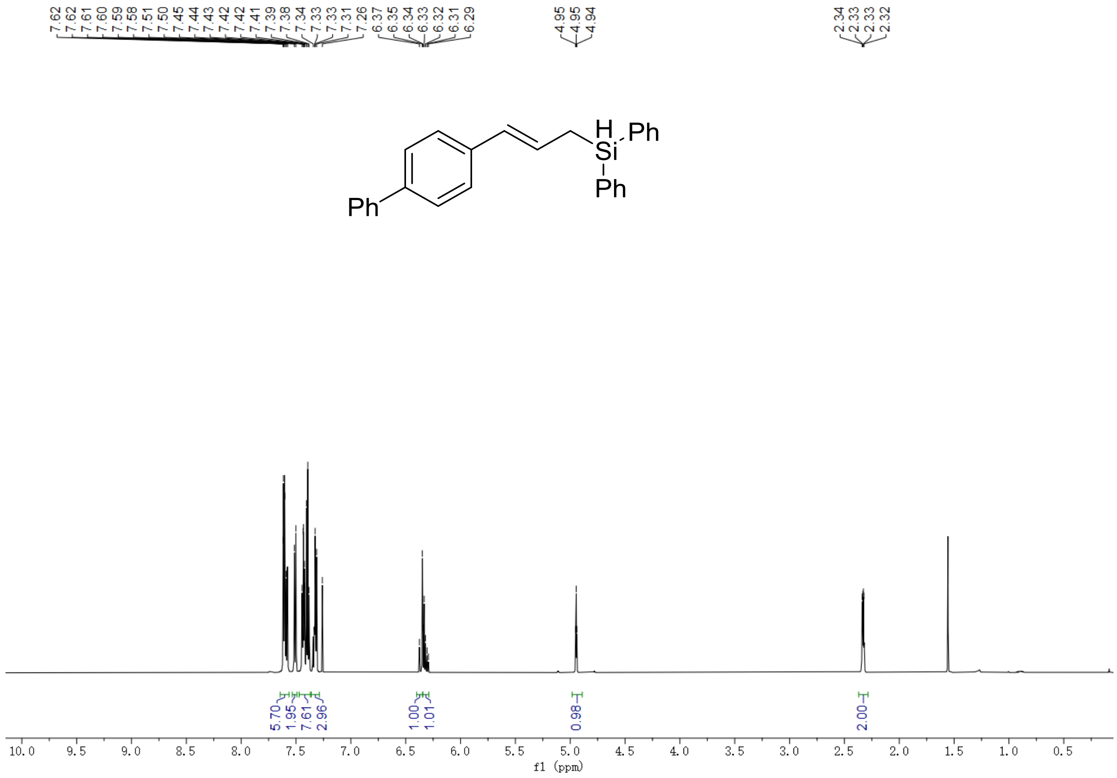

Supplementary Figure 96. ^{1}H NMR (600 MHz, $CDCl_3$, 25 ºC) spectra of **5p**.

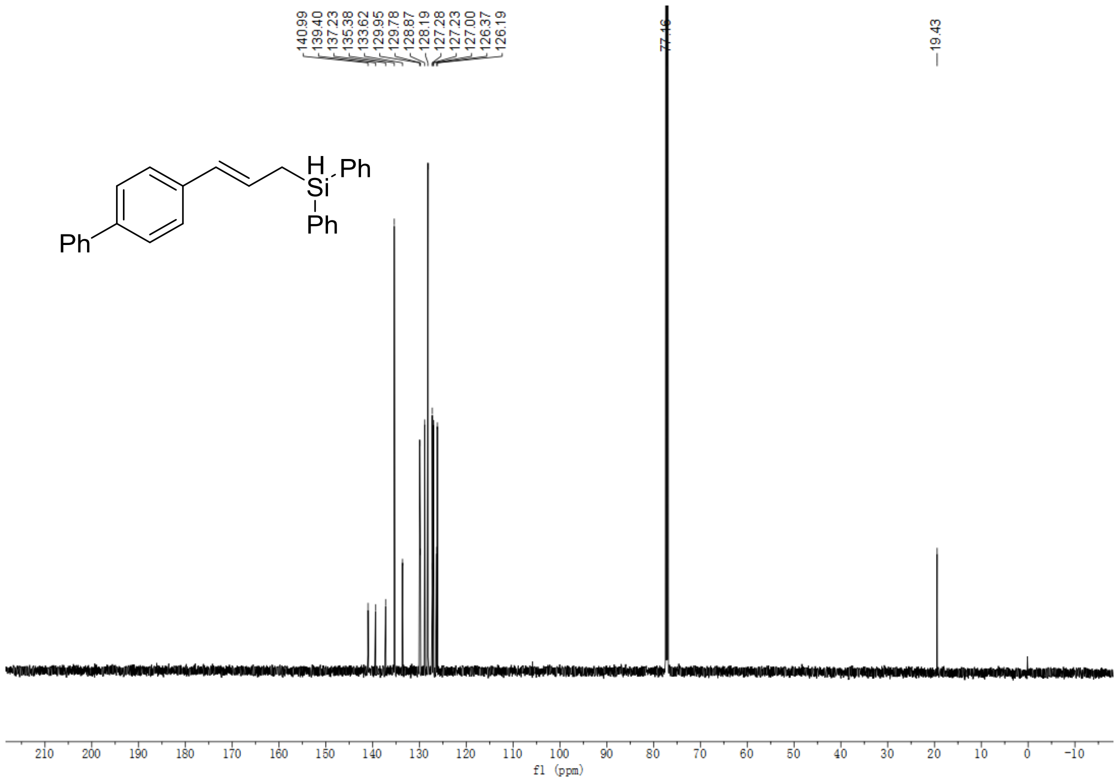

Supplementary Figure 97. ^{13}C NMR (151 MHz, $CDCl_3$, 25 ºC) spectra of **5p**.

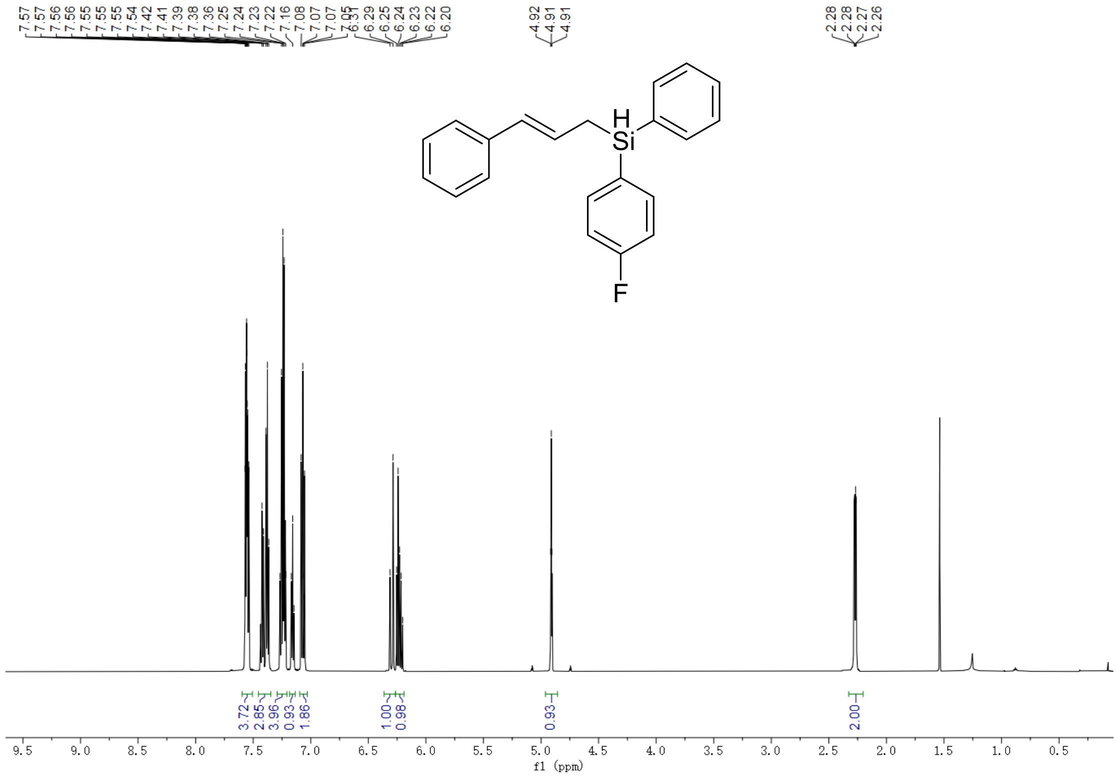

Supplementary Figure 98. ^{1}H NMR (600 MHz, $CDCl_3$, 25 ºC) spectra of **5q**.

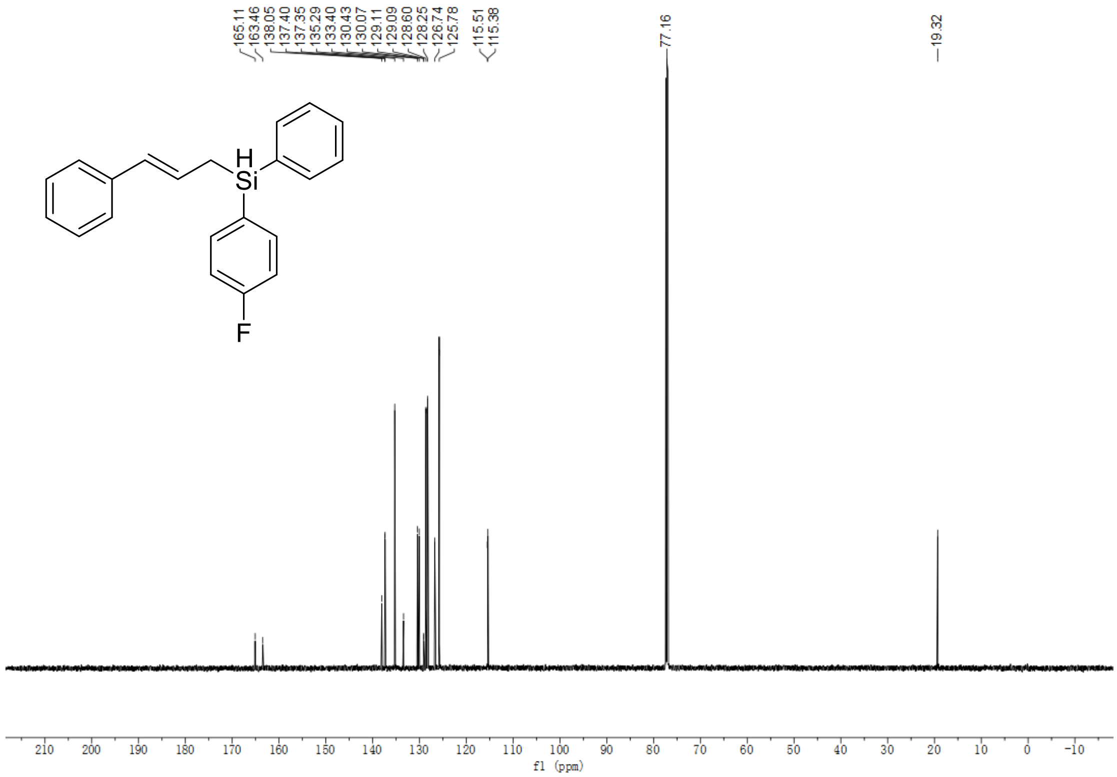

Supplementary Figure 99. ^{13}C NMR (151 MHz, $CDCl_3$, 25 ºC) spectra of **5q**.

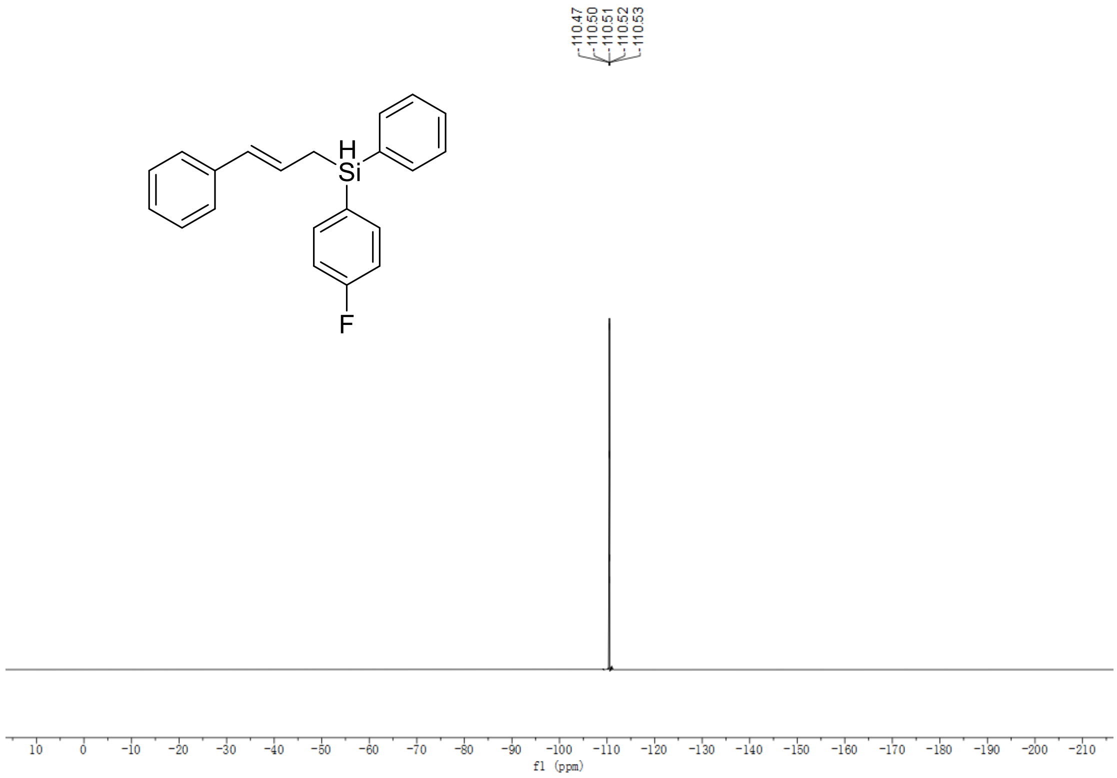

Supplementary Figure 100. ^{19}F NMR (565 MHz, $CDCl_3$, 25 ºC) spectra of **5q**.

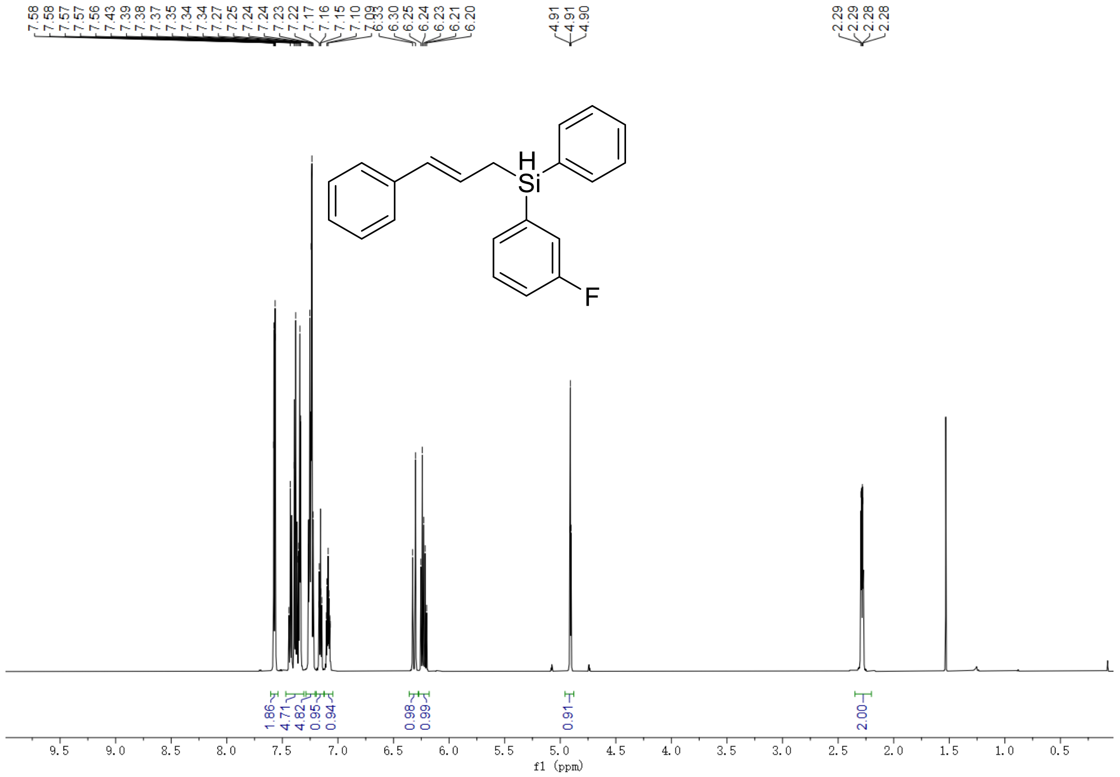

Supplementary Figure 101. ^{1}H NMR (600 MHz, $CDCl_3$, 25 ºC) spectra of **5r**.

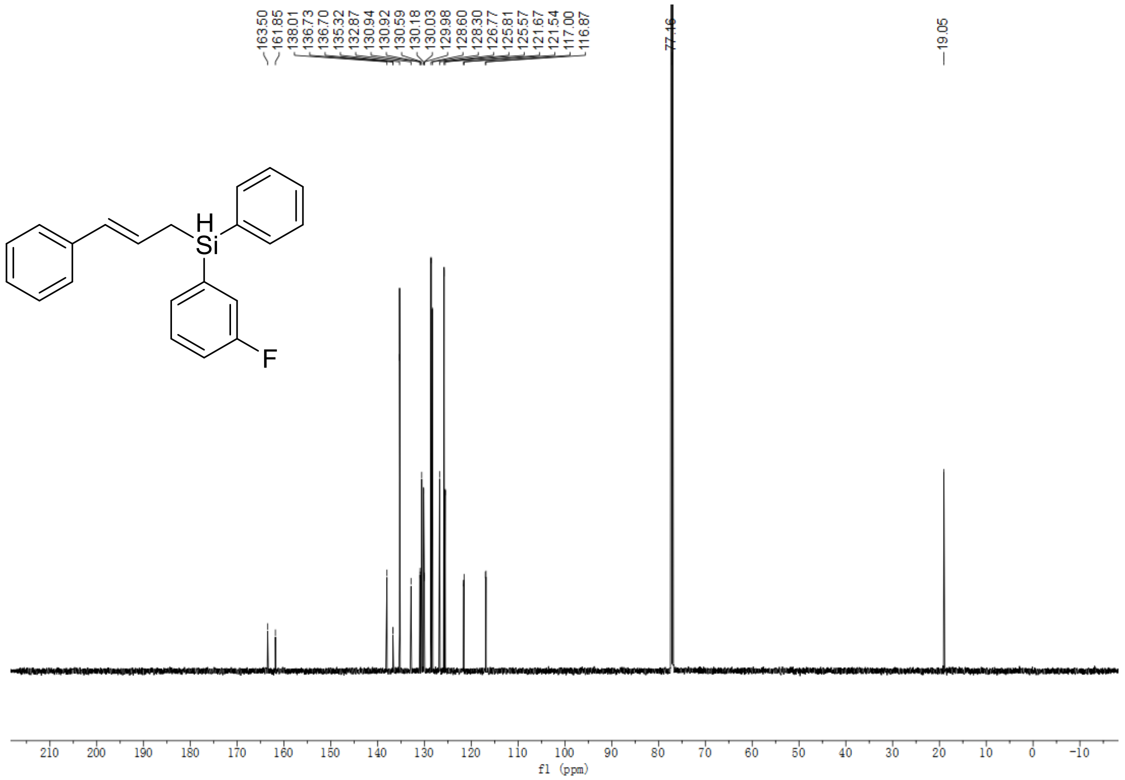

Supplementary Figure 102. ^{13}C NMR (151 MHz, $CDCl_3$, 25 ºC) spectra of **5r**.

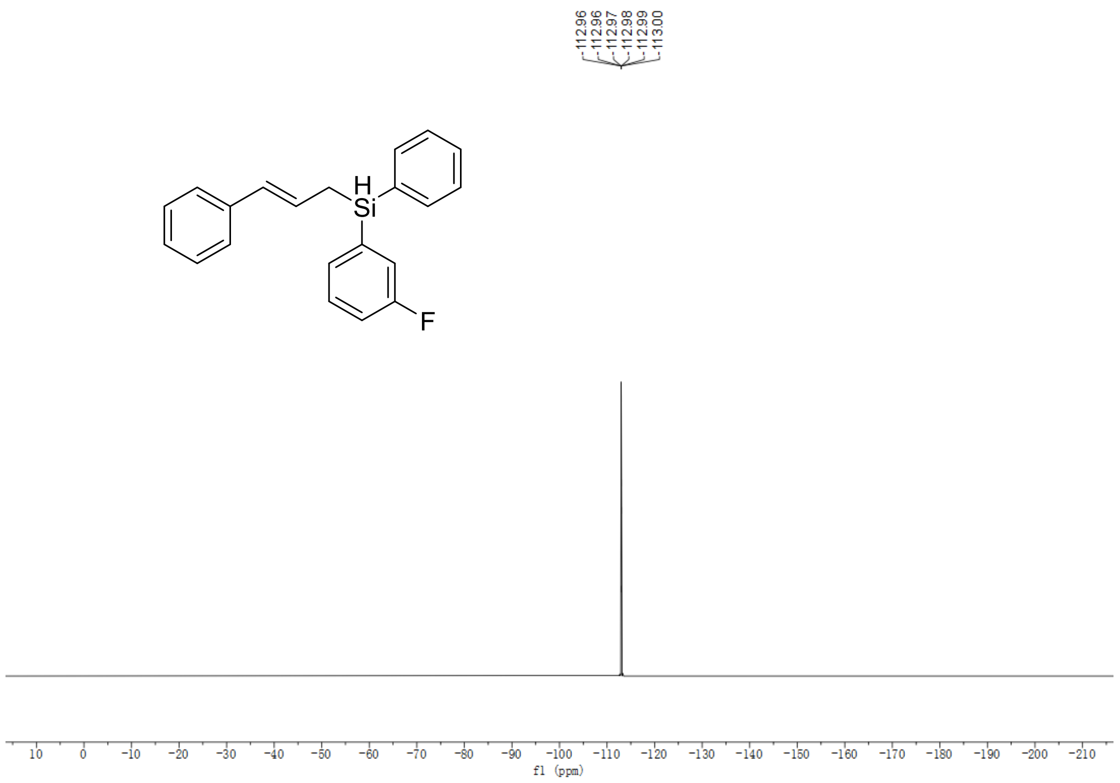

Supplementary Figure 103. ^{19}F NMR (565 MHz, $CDCl_3$, 25 ºC) spectra of **5r**.

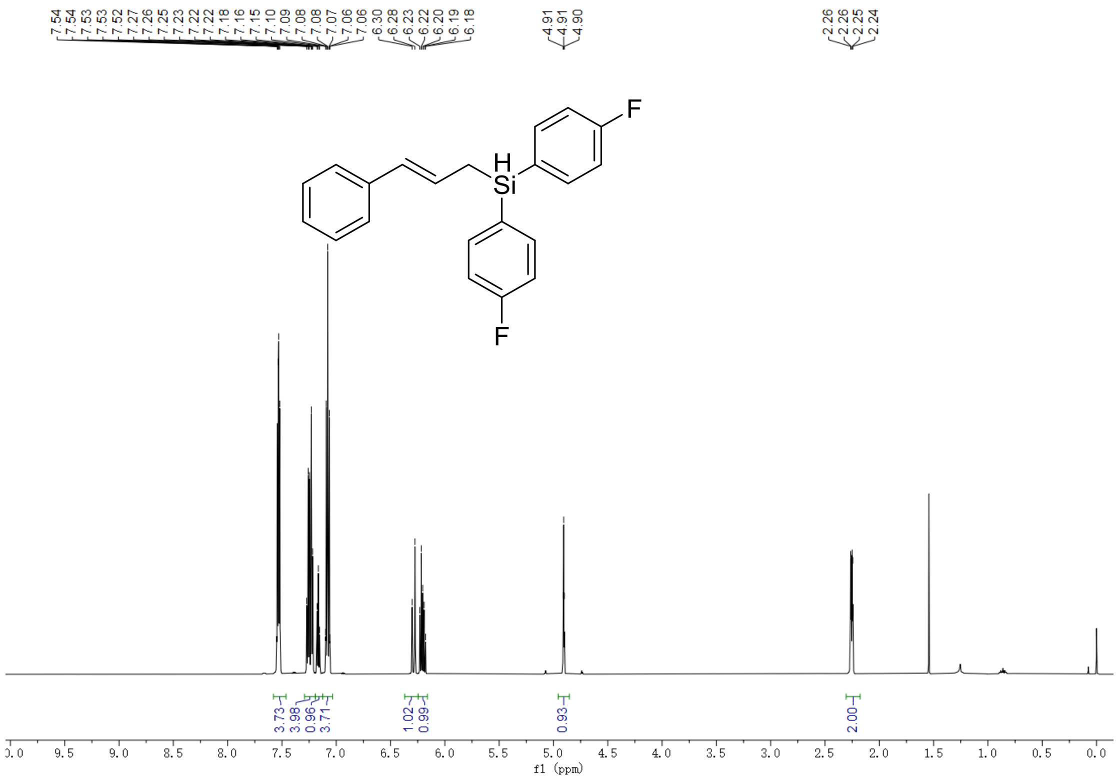

Supplementary Figure 104. ^{1}H NMR (600 MHz, $CDCl_3$, 25 ºC) spectra of **5s**.

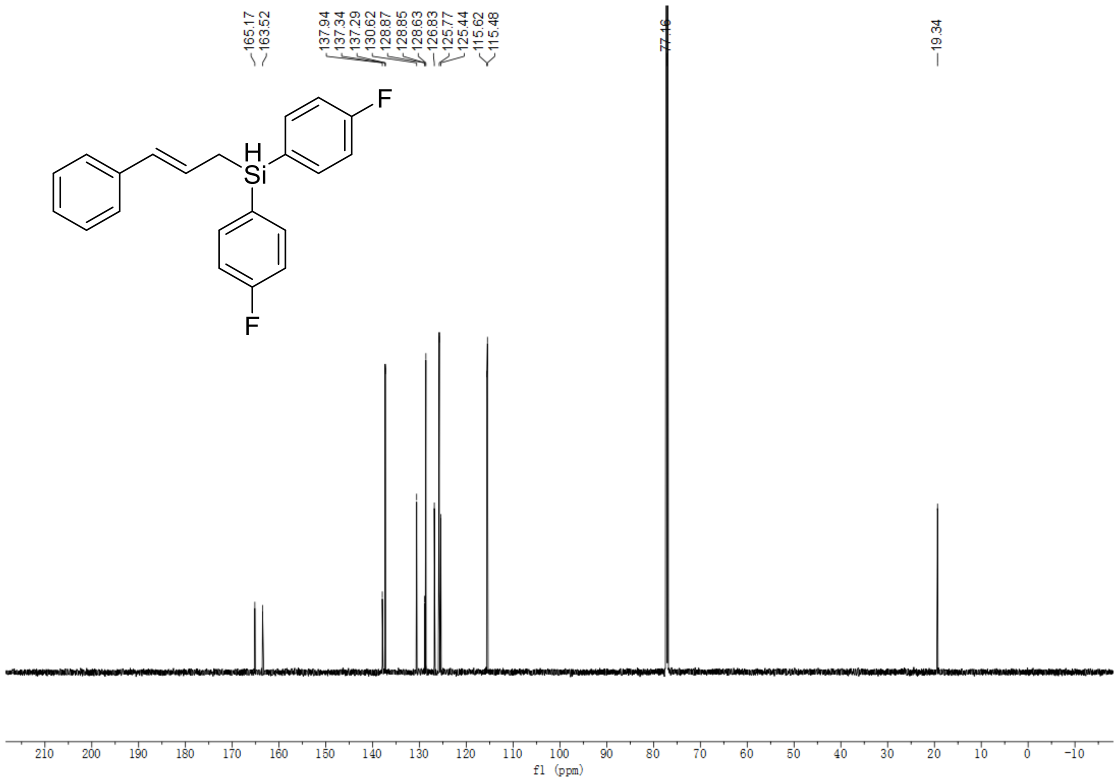

Supplementary Figure 105. ^{13}C NMR (151 MHz, $CDCl_3$, 25 ºC) spectra of **5s**.

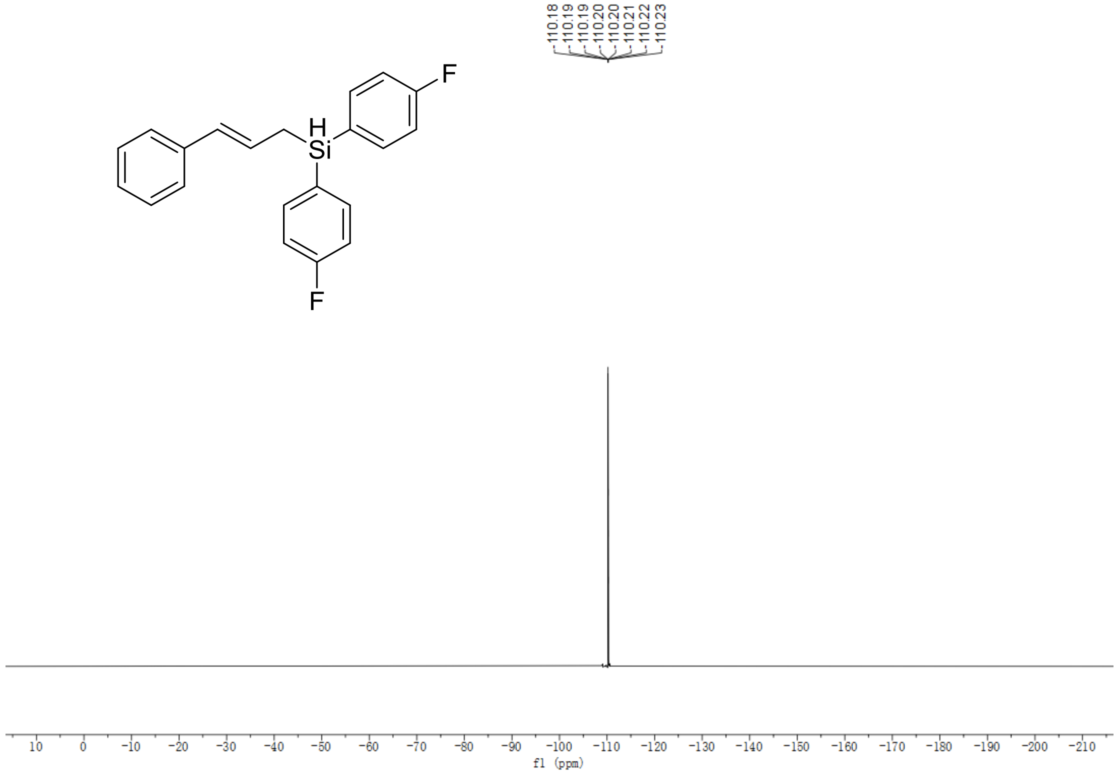

Supplementary Figure 106. ^{19}F NMR (565 MHz, $CDCl_3$, 25 ºC) spectra of **5s**.

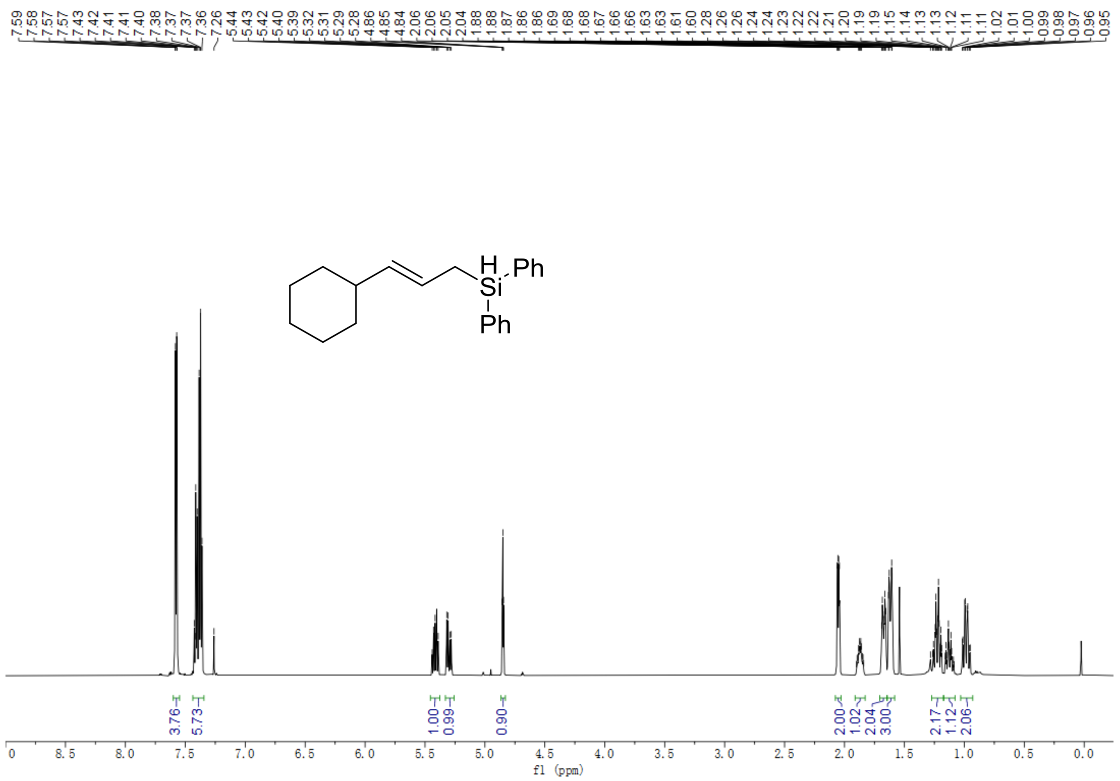

Supplementary Figure 107. ^{1}H NMR (600 MHz, $CDCl_3$, 25 ºC) spectra of **5t** (*E* product).

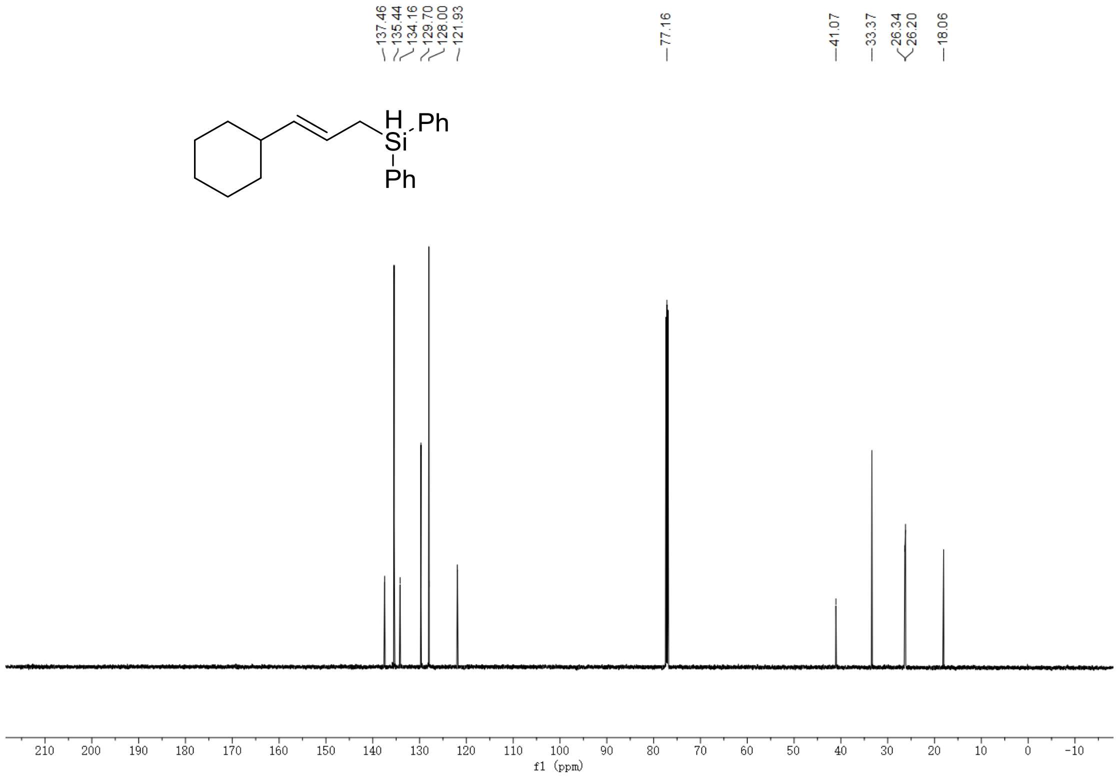

Supplementary Figure 108. ^{13}C NMR (151 MHz, $CDCl_3$, 25 ºC) spectra of **5t** (*E* product)

.

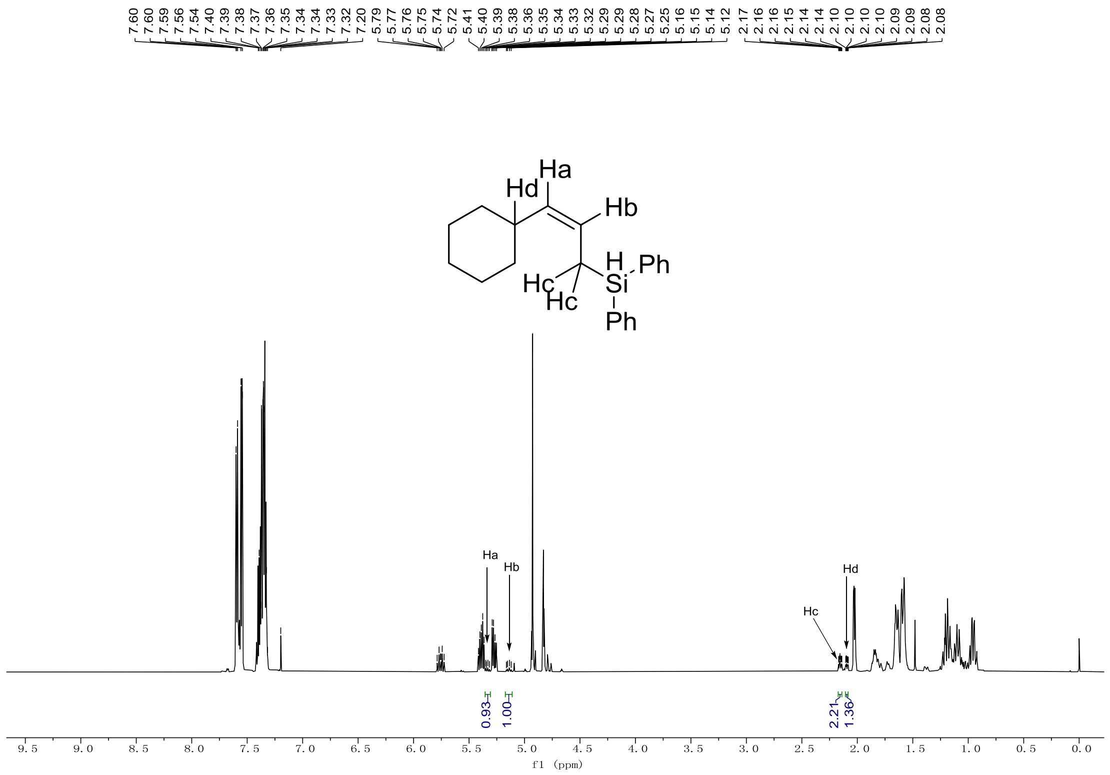

Supplementary Figure 109. ^{1}H NMR (600 MHz, $CDCl_3$, 25 ºC) spectra of **5t** (*Z* product).

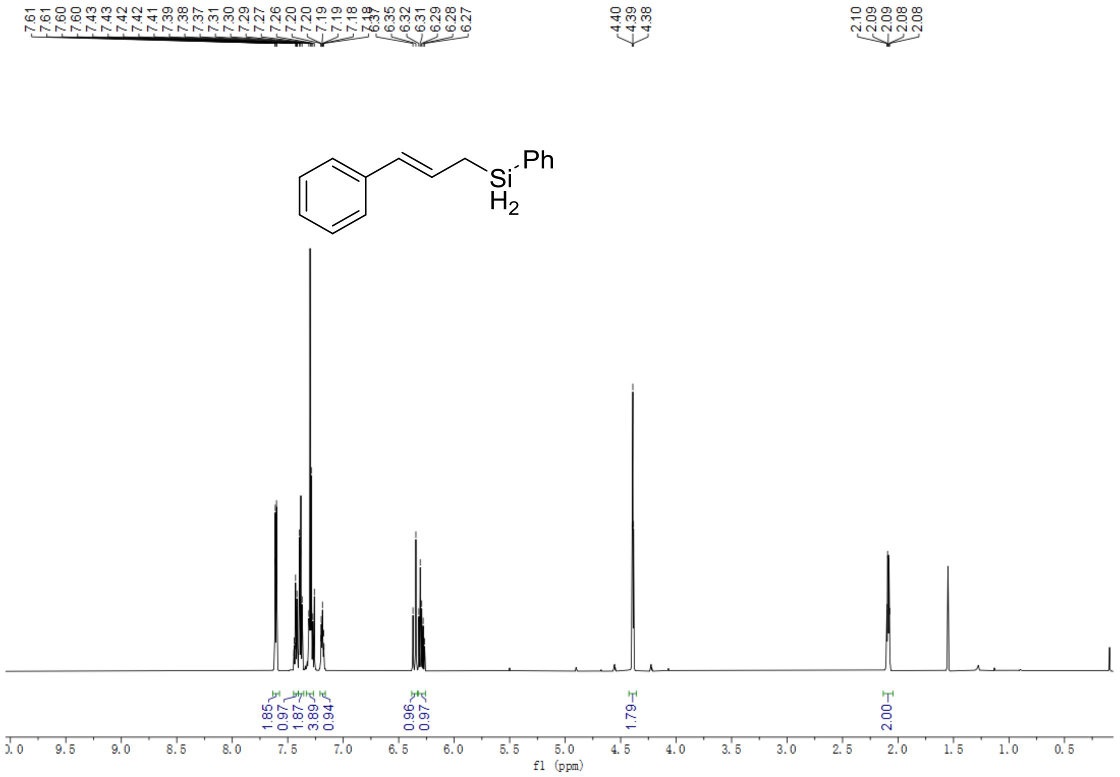

Supplementary Figure 110. ^{1}H NMR (600 MHz, $CDCl_3$, 25 ºC) spectra of **5u**.

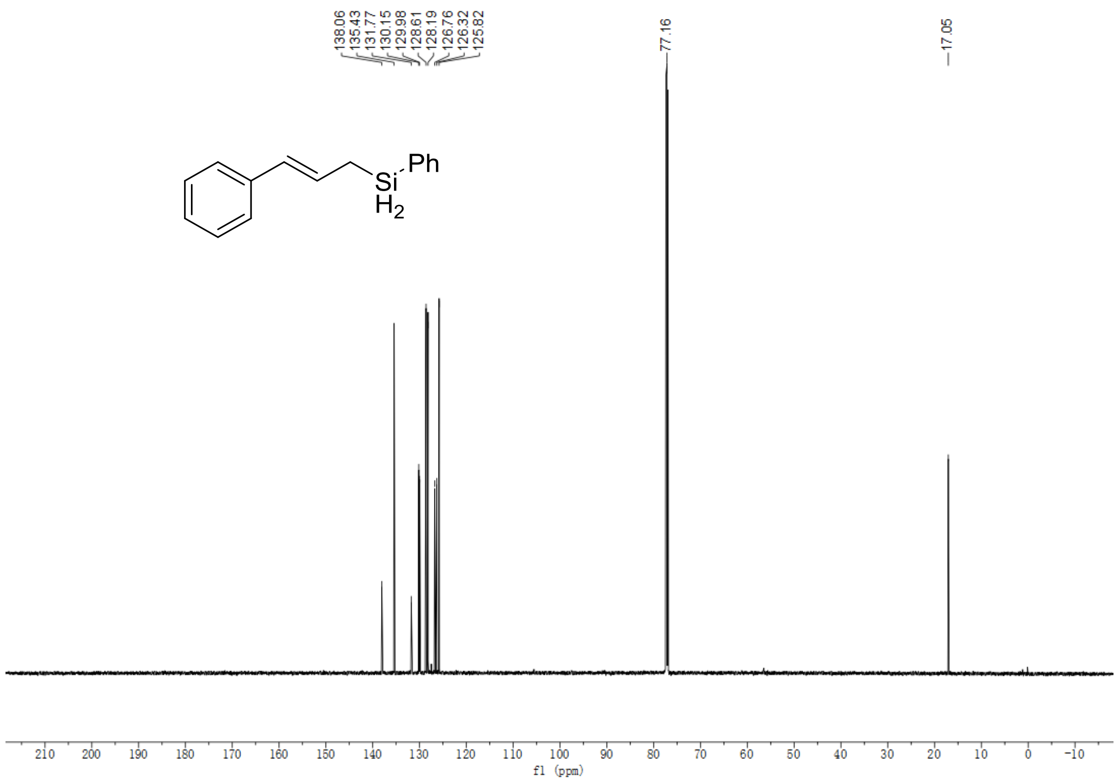

Supplementary Figure 111. ^{13}C NMR (151 MHz, $CDCl_3$, 25 ºC) spectra of **5u**.

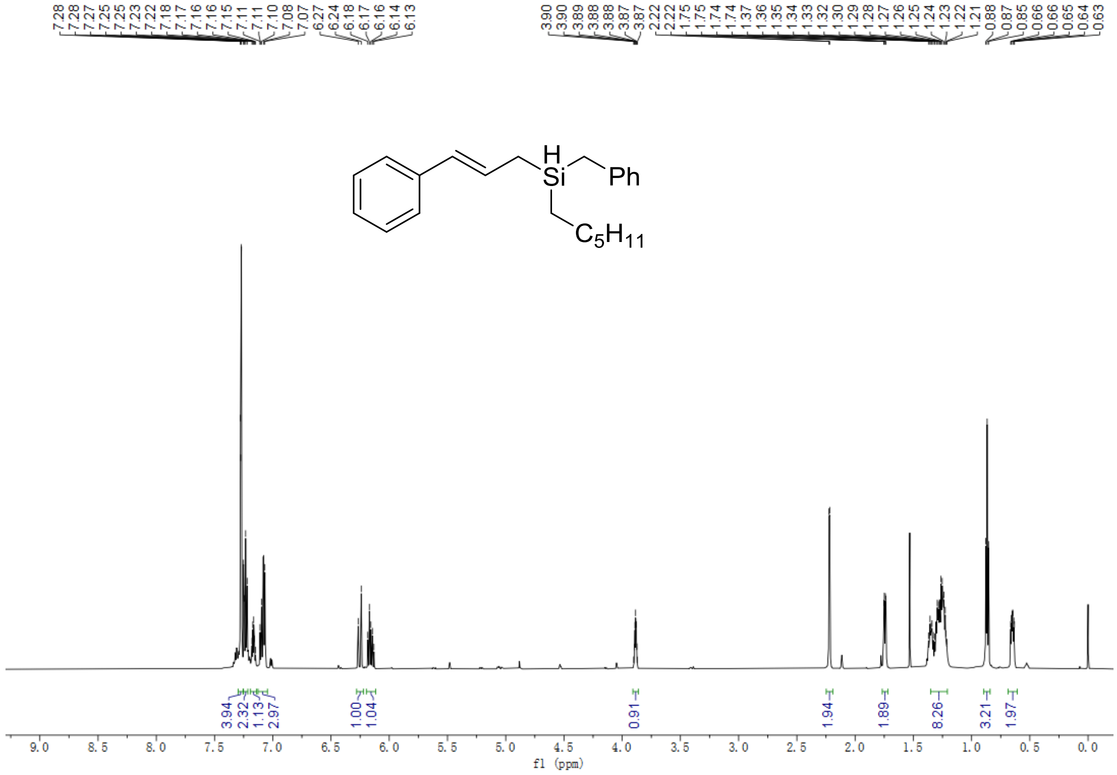

Supplementary Figure 112. ^{1}H NMR (600 MHz, $CDCl_3$, 25 ºC) spectra of **5v**.

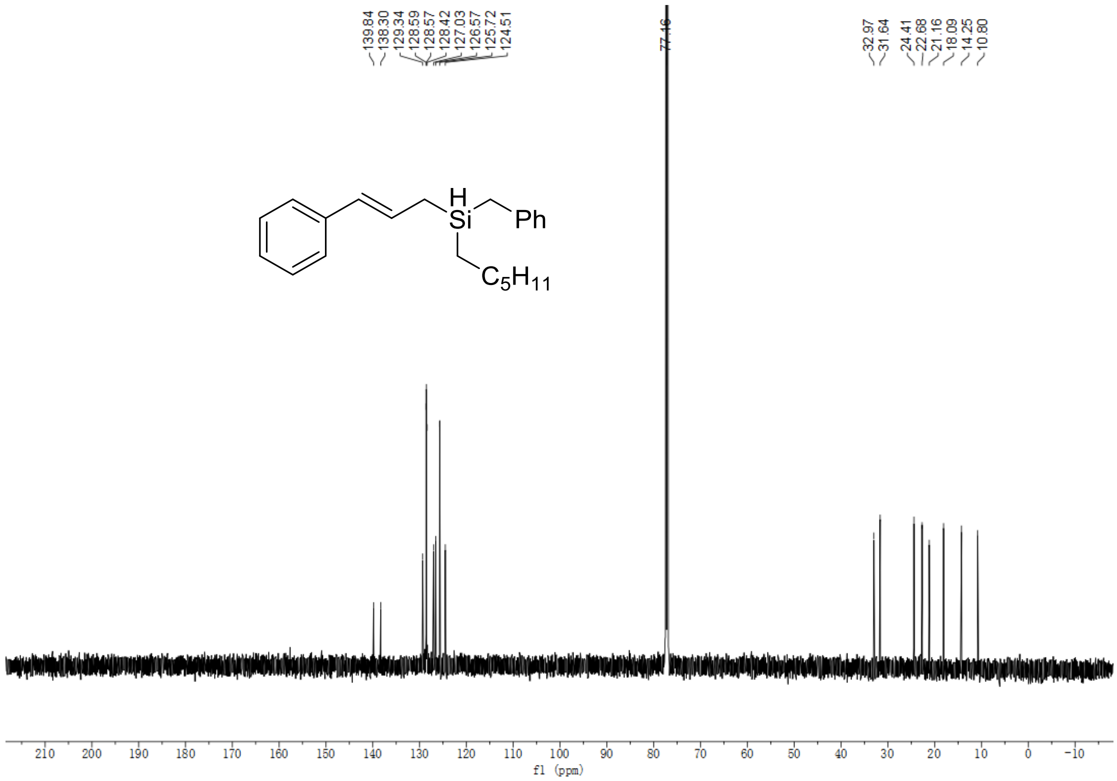

Supplementary Figure 113. ^{13}C NMR (151 MHz, $CDCl_3$, 25 ºC) spectra of **5v**.

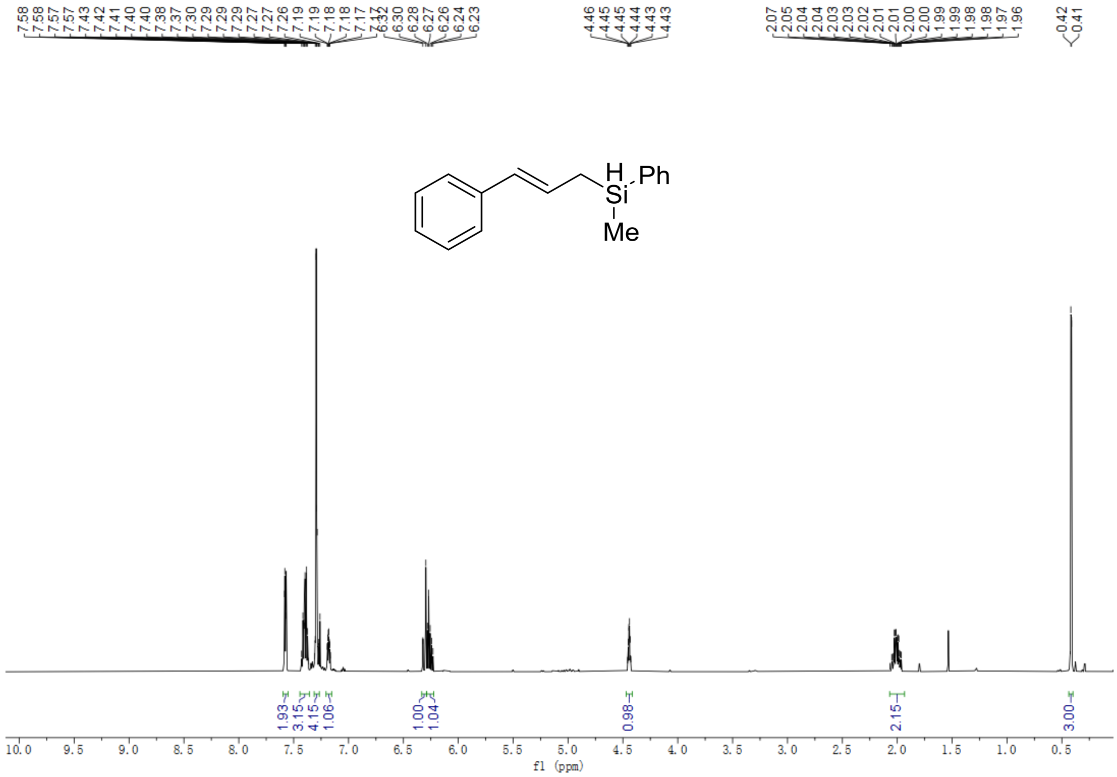

Supplementary Figure 114. ^{1}H NMR (600 MHz, $CDCl_3$, 25 ºC) spectra of **5w**.

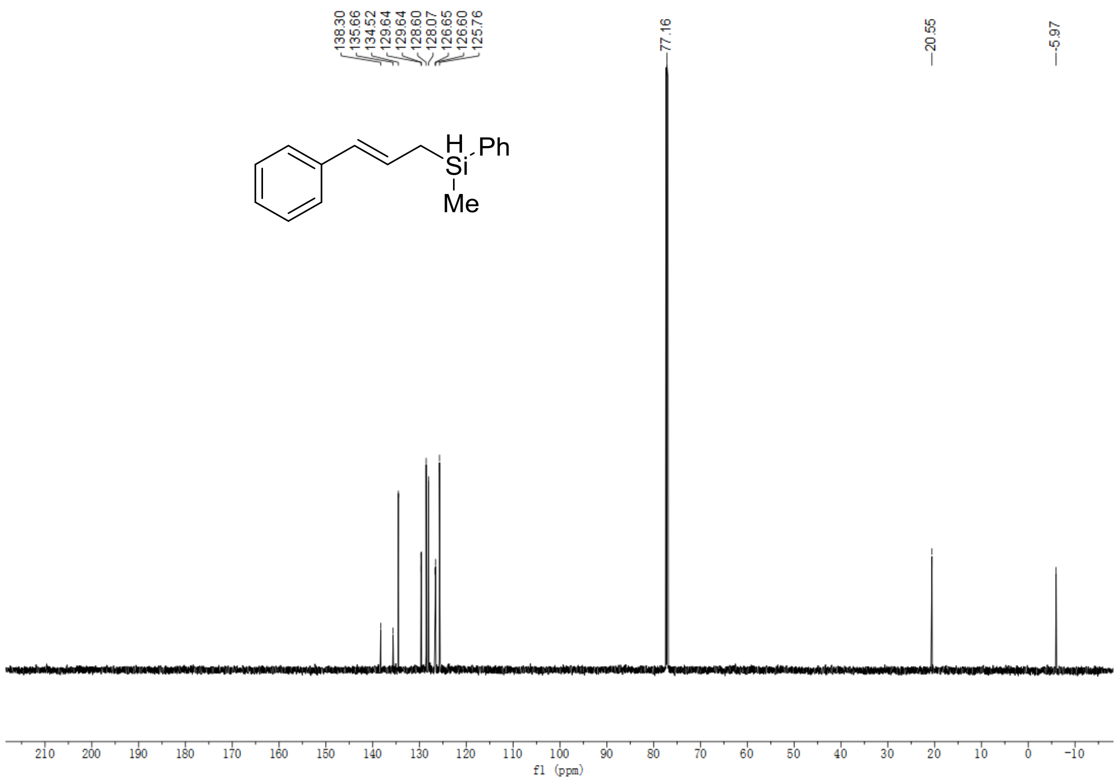

Supplementary Figure 115. ^{13}C NMR (151 MHz, $CDCl_3$, 25 ºC) spectra of **5w**.

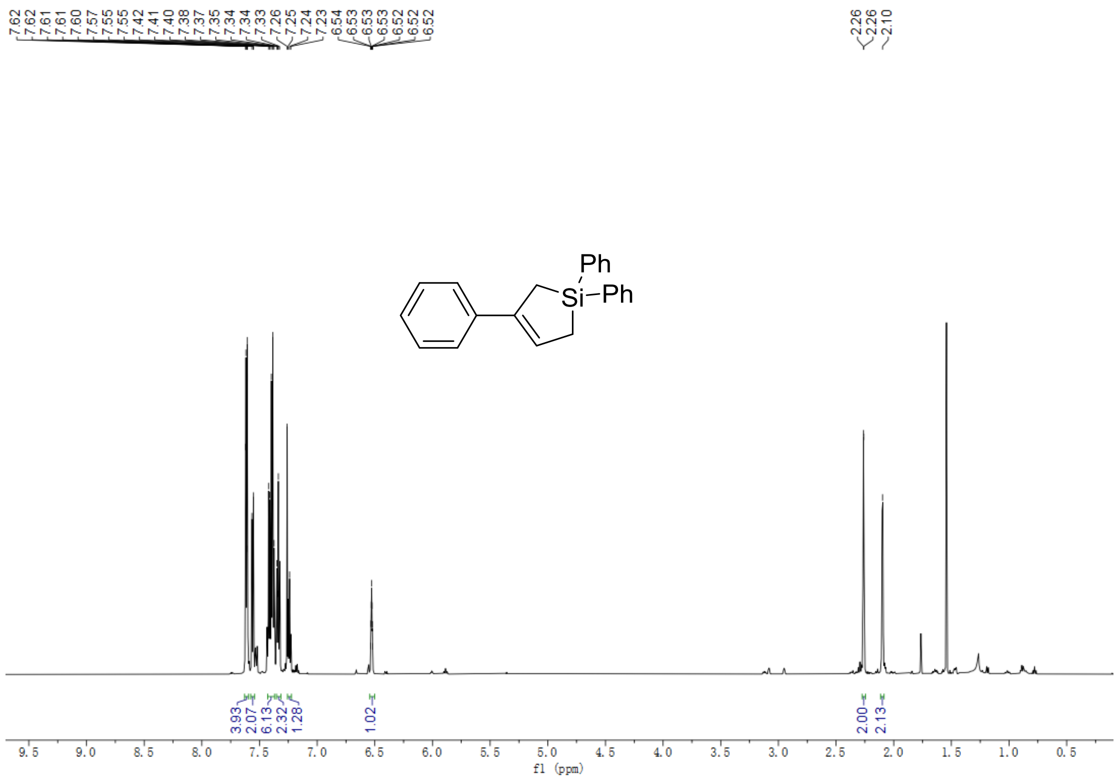

Supplementary Figure 116. ^{1}H NMR (600 MHz, $CDCl_3$, 25 ºC) spectra of **6**.

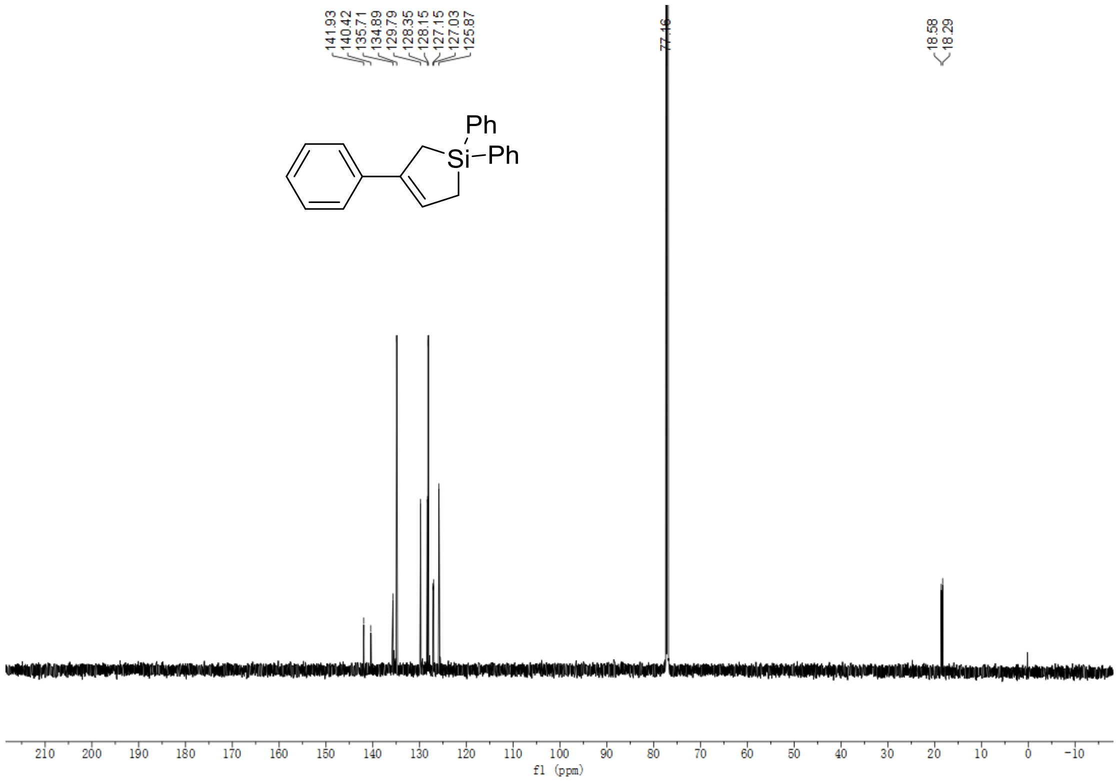

Supplementary Figure 117. ^{13}C NMR (151 MHz, $CDCl_3$, 25 ºC) spectra of **6**.

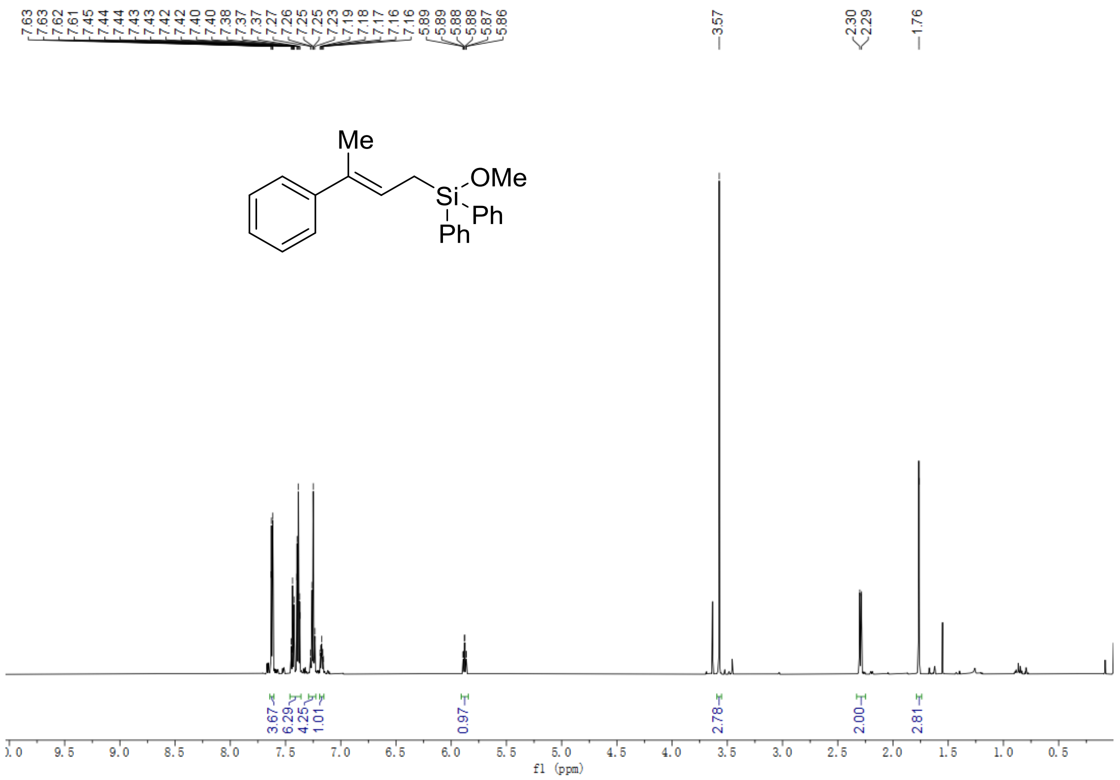

Supplementary Figure 118. ^{1}H NMR (600 MHz, $CDCl_3$, 25 ºC) spectra of **7**.

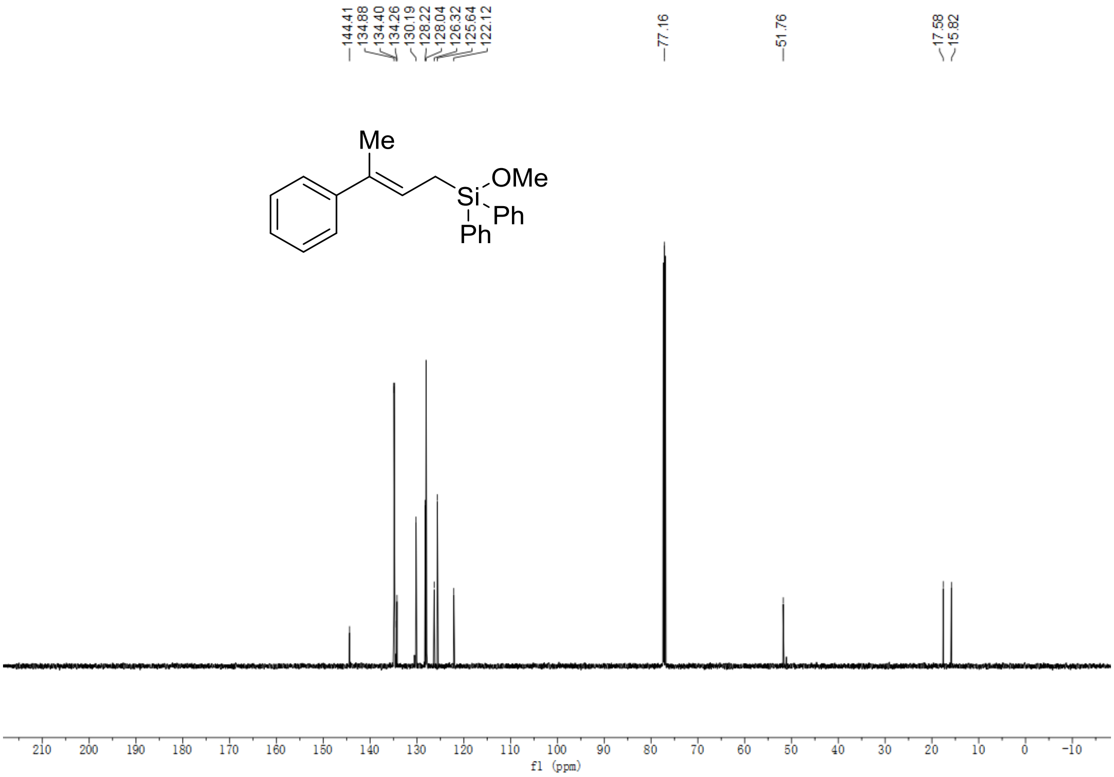

Supplementary Figure 119. ^{13}C NMR (151 MHz, $CDCl_3$, 25 ºC) spectra of **7**.

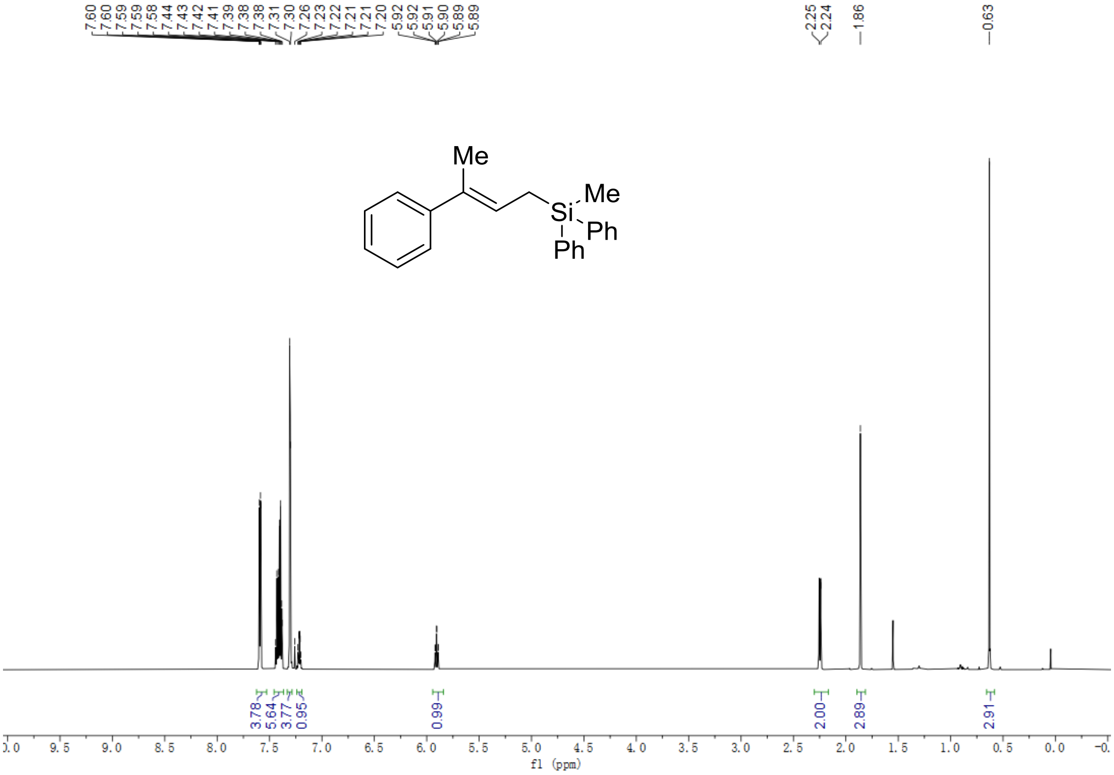

Supplementary Figure 120. ^{1}H NMR (600 MHz, $CDCl_3$, 25 ºC) spectra of **8**.

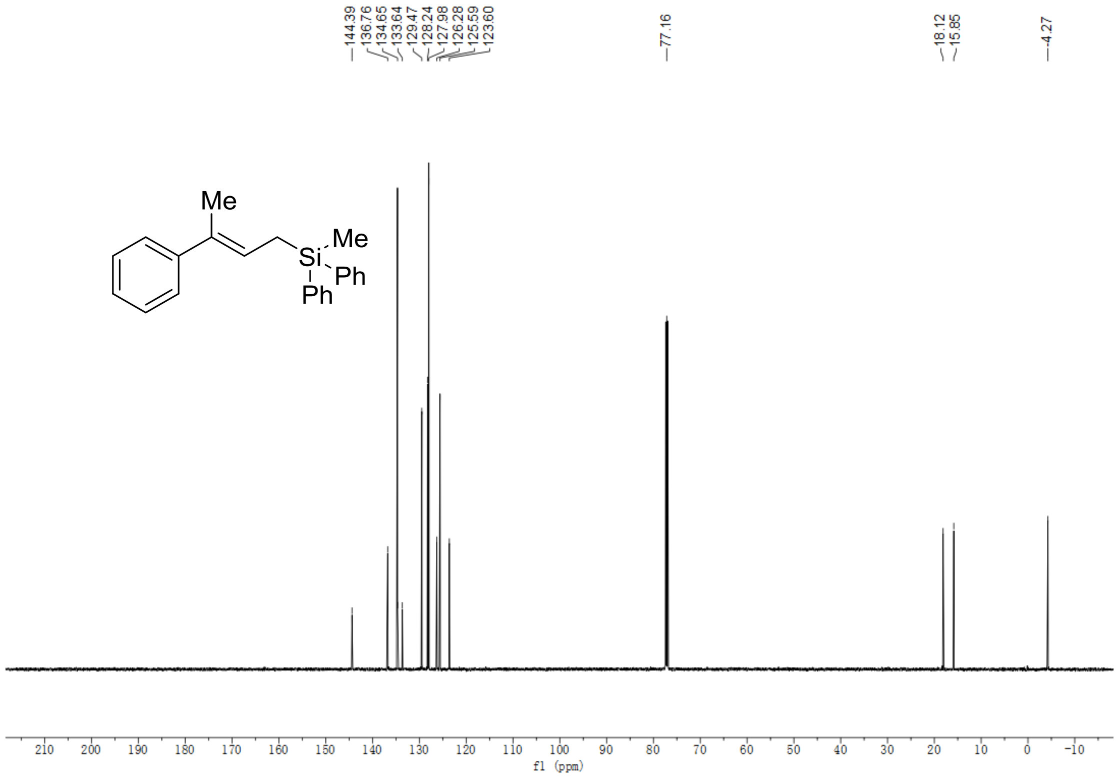

Supplementary Figure 121. ^{13}C NMR (151 MHz, $CDCl_3$, 25 ºC) spectra of **8**.

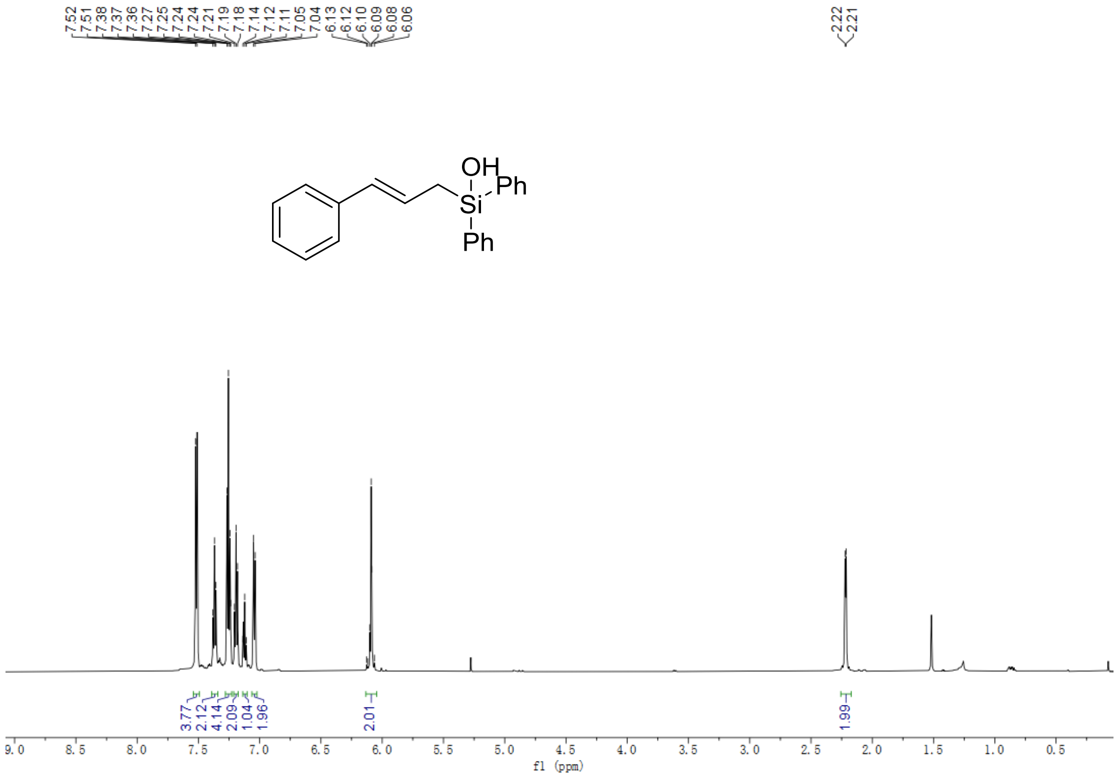

Supplementary Figure 122. ^{1}H NMR (600 MHz, $CDCl_3$, 25 ºC) spectra of **10**.

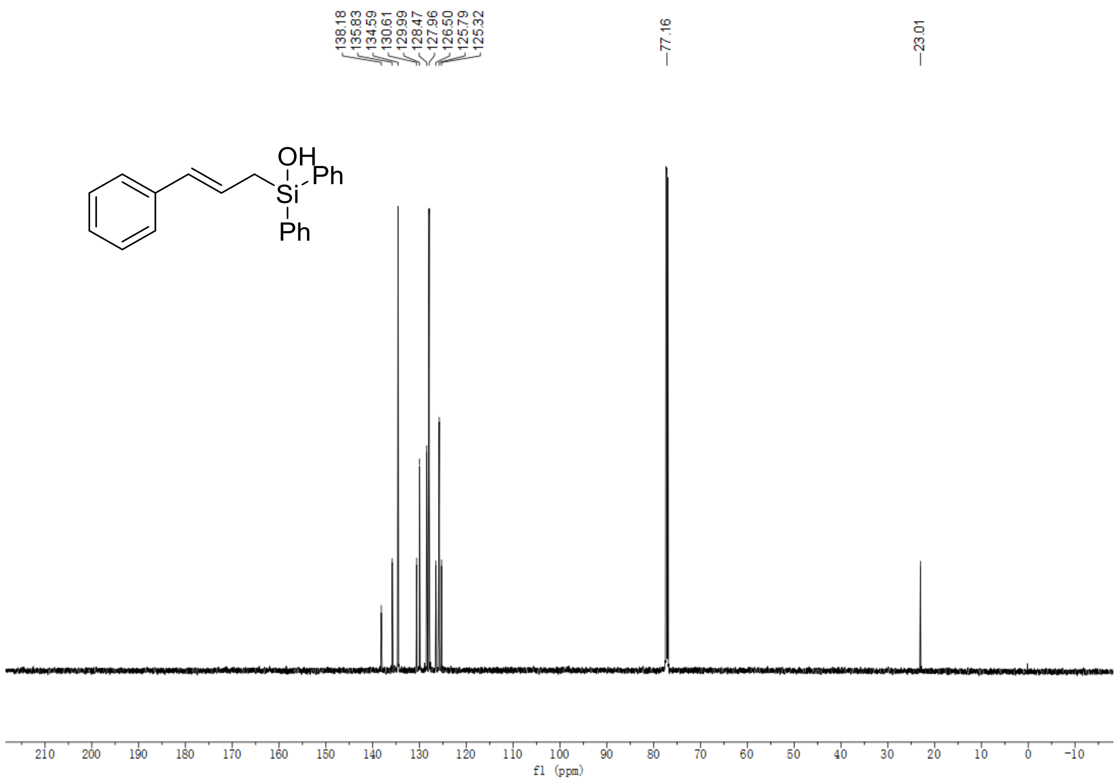

Supplementary Figure 123. ^{13}C NMR (151 MHz, $CDCl_3$, 25 ºC) spectra of **10**.

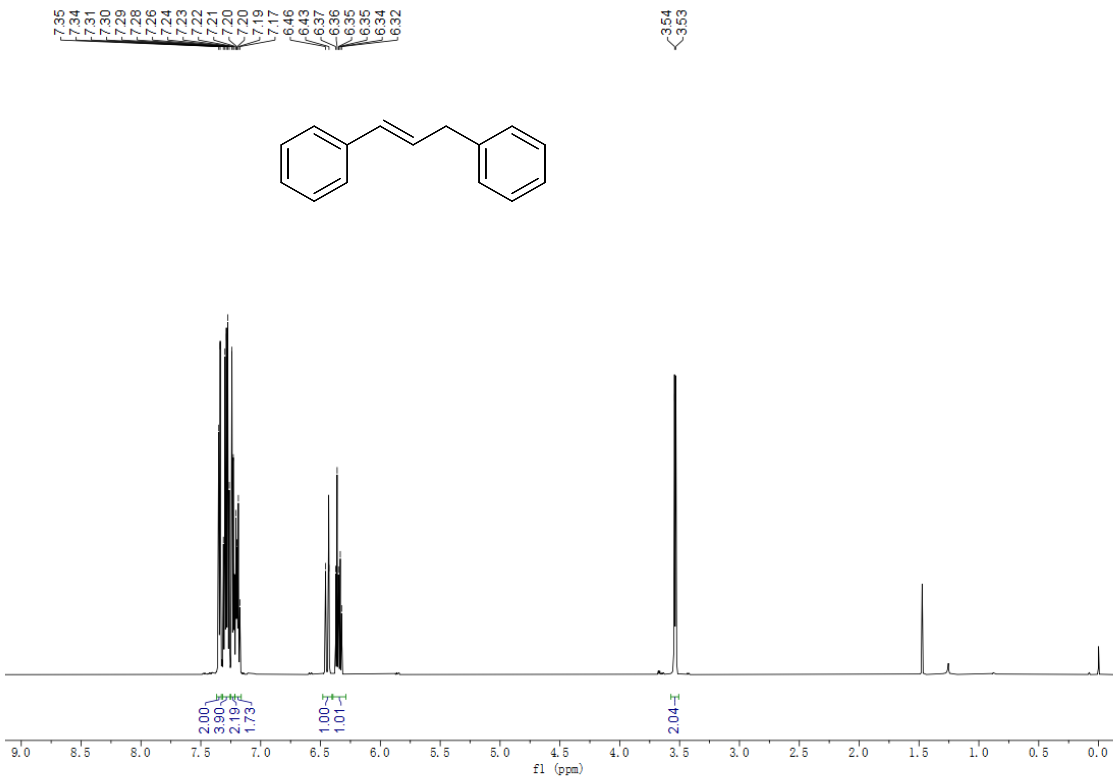

Supplementary Figure 124. ^{1}H NMR (600 MHz, $CDCl_3$, 25 ºC) spectra of **11**.

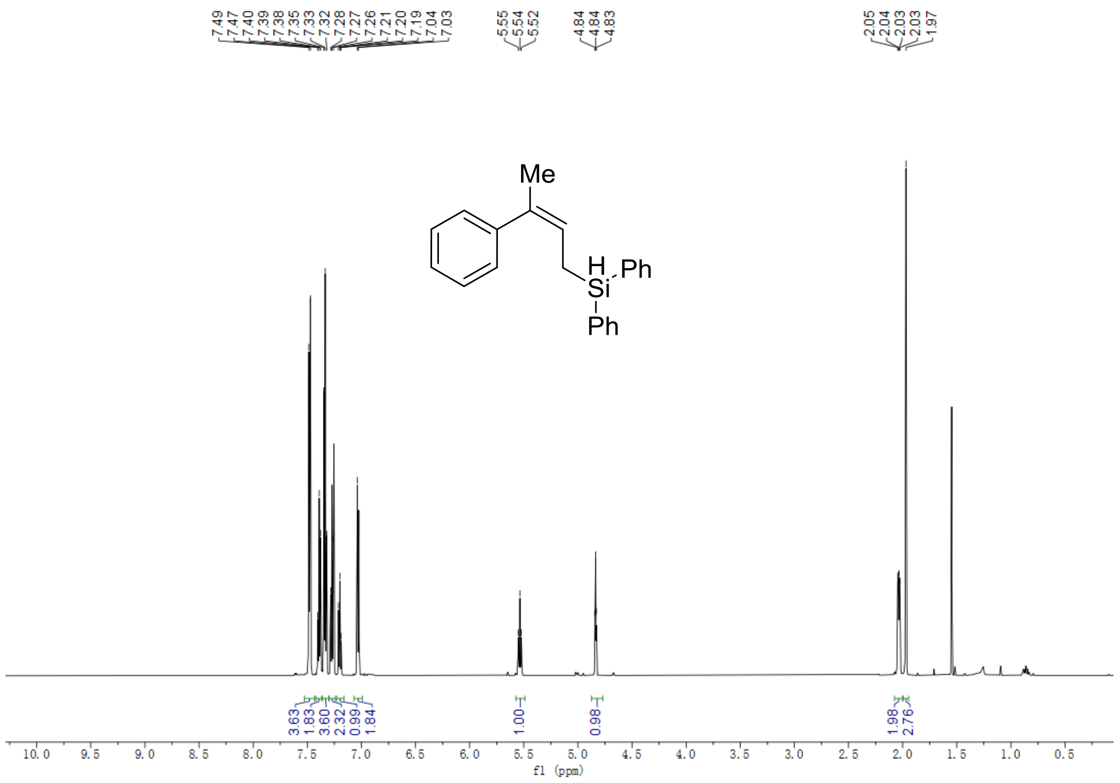

Supplementary Figure 125. ^{1}H NMR (600 MHz, $CDCl_3$, 25 ºC) spectra of ***Z*-3a**.

3. Supplementary References

1. Qiu, J., Gao, S., Li, C., Zhang, L., Wang, Z., Wang, X & Ding, K. Construction of All-Carbon Chiral Quaternary Centers through Cu(I) -Catalyzed Enantioselective Reductive Hydroxymethylation of 1,1-Disubstituted Allenes with CO_2. *Chem. Eur. J.* **25**, 13874–13878 (2019).
2. Xu, Z., Shen, C., Zhang, H., Wang, P & Dong, K. Constructing chiral aza-quaternary carbon centers by enantioselective carbonylative Heck reaction of o-iodoanilines with allenes. *Org. Chem. Front.* **8**, 1163–1169 (2021).
3. Kiyokawa, K., Hata, S., Kainuma, S & Minakata, S. Electrophilic cyanation of allylic boranes: synthesis of, β,γ-unsaturated nitriles containing allylic quaternary carbon centers. *Chem Commun.* **55**, 458–461 (2019).
4. Liu, J., Nie, M., Zhou, Q., Gao, S., Jiang, W., Chung, L. W., Tang, W & Ding, K. Enantioselective palladium-catalyzed diboration of 1,1-disubstituted allenes. *Chem Sci.* **8**, 5161–5165 (2017).
5. Wang, J.–S., Yao, L., Ying, J., Luo, X & Wu, X.–F. Palladium-catalyzed directing group assisted and regioselectivity reversed cyclocarbonylation of arylallenes with 2–iodoanilines. *Organic Chemistry Frontiers*. **8**, 792–798 (2021).
6. Zhao, Z & Murphy, G. K. Chlorination of phenylallene derivatives with 1-chloro-1,2-benziodoxol-3-one: synthesis of vicinal-dichlorides and chlorodienes. *Beilstein J Org Chem.* **14**, 796–802 (2018).
7. Wang, Z. L., Wang, Y., Xu, J. L., Zhao, M., Dai, K. Y., Shan, C. C & Xu, Y. H. Synthesis of Structurally Diverse Allylsilanes via Copper-Catalyzed Regiodivergent Hydrosilylation of 1,3-

Dienes. *Org. Lett.* **23**, 4736–4742 (2021).
8. Asako, S., Ishikawa, S & Takai, K. Synthesis of Linear Allylsilanes via Molybdenum-Catalyzed Regioselective Hydrosilylation of Allenes. *ACS Catal.* **6**, 3387–3395 (2016).
9. Sokolova, D & Tiefenbacher, K. Optimized iminium-catalysed 1,4-reductions inside the resorcinarene capsule: achieving >90% ee with proline as catalyst. *RSC Adv.* **11**, 24607–24612 (2021).
10. Wang, C., Teo, W. J & Ge, S. Access to stereodefined (Z)-allylsilanes and (Z)-allylic alcohols via cobalt-catalyzed regioselective hydrosilylation of allenes. *Nat. Commun.* **8**, 2258 (2017).
11. Wang, Y., Shao, Z., Zhang, K & Liu, Q. Manganese-Catalyzed Dual-Deoxygenative Coupling of Primary Alcohols with 2-Arylethanols. *Angew. Chem. Int. Ed.* **57**, 15143–15147 (2018).
12. Gaussian 16, Revision C.01, Frisch, M. J.; Trucks, G. W.; Schlegel, H. B.; Scuseria, G. E.; Robb, M. A.; Cheeseman, J. R.; Scalmani, G.; Barone, V.; Petersson, G. A.; Nakatsuji, H.; Li, X.; Caricato, M.; Marenich, A. V.; Bloino, J.; Janesko, B. G.; Gomperts, R.; Mennucci, B.; Hratchian, H. P.; Ortiz, J. V.; Izmaylov, A. F.; Sonnenberg, J. L.; Williams-Young, D.; Ding, F.; Lipparini, F.; Egidi, F.; Goings, J.; Peng, B.; Petrone, A.; Henderson, T.; Ranasinghe, D.; Zakrzewski, V. G.; Gao, J.; Rega, N.; Zheng, G.; Liang, W.; Hada, M.; Ehara, M.; Toyota, K.; Fukuda, R.; Hasegawa, J.; Ishida, M.; Nakajima, T.; Honda, Y.; Kitao, O.; Nakai, H.; Vreven, T.; Throssell, K.; Montgomery, J. A., Jr.; Peralta, J. E.; Ogliaro, F.; Bearpark, M. J.; Heyd, J. J.; Brothers, E. N.; Kudin, K. N.; Staroverov, V. N.; Keith, T. A.; Kobayashi, R.; Normand, J.; Raghavachari, K.; Rendell, A. P.; Burant, J. C.; Iyengar, S. S.; Tomasi, J.; Cossi, M.; Millam, J. M.; Klene, M.; Adamo, C.; Cammi, R.; Ochterski, J. W.; Martin, R. L.; Morokuma, K.; Farkas, O.; Foresman, J. B.; Fox, D. J. Gaussian, Inc., Wallingford CT, 2019.
13. (a) Lee, C.; Yang, W.; Parr, R. G. Development of the Colle-Salvetti Correlation-Energy Formula into a Functional of the Electron Density. *Phys. Rev. B* **37**, 785-789 (1988); (b) Becke, A. D. Density‐Functional Thermochemistry. III. The Role of Exact Exchange." *J. Chem. Phys.* **98**, 5648-5652 (1993).
14. Zhao, Y.; Truhlar, D. G. The M06 Suite of Density Functionals for Main Group Thermochemistry, Thermochemical Kinetics, Noncovalent Interactions, Excited States, and Transition Elements: Two New Functionals and Systematic Testing of Four M06-Class Functionals and 12 Other Functionals. *Theor. Chem. Acc.* **120**, 215-241 (2008).
15. Marenich, A. V.; Cramer, C. J.; Truhlar, D. G. Universal Solvation Model Based on Solute Electron Density and on a Continuum Model of the Solvent Defined by the Bulk S-95 Dielectric Constant and Atomic Surface Tensions. *J. Phys. Chem.* B **113**, 6378-6396 (2009).
16. CYLview, 1.0b; C. Y. Legault, Université de Sherbrooke, (2009).
17. Lefebvre, C.; Rubez, G.; Khartabil, H.; Boisson, J. C.; Contreras-García, J.; Hénon, E. Accurately extracting the signature of intermolecular interactions present in the NCI plot of the reduced density gradient versus electron density. *Phys. Chem. Chem.* **19**, 17928-17936 (2017.)
