# Supplementary material for: Copper-catalyzed regio- and stereo-selective hydrosilylation of terminal allenes to access (E)-allylsilanes
Source: Nat Commun. 2022 Jun 27;13:3691. doi: 10.1038/s41467-022-31458-2 (PMC9237096; doi:10.1038/s41467-022-31458-2)
Supplement: Supplementary file 3 — Description of Additional Supplementary Files [file 41467_2022_31458_MOESM3_ESM.pdf]

Supplementary Data File 1: Cartesian coordinates and energies of the optimized structures
