# Supplementary material for: Copper-catalyzed regio- and stereo-selective hydrosilylation of terminal allenes to access (E)-allylsilanes
Source: Nat Commun. 2022 Jun 27;13:3691. doi: 10.1038/s41467-022-31458-2 (PMC9237096; doi:10.1038/s41467-022-31458-2)
Supplement: Supplementary file 4 — Supplementary data 1 [file 41467_2022_31458_MOESM4_ESM.pdf]

Cartesian coordinates (Å) and energies of optimized structures

1a

B3LYP-D3(BJ) SCF energy: -387.06148507 a.u.
B3LYP-D3(BJ) enthalpy: -386.884745 a.u.
B3LYP-D3(BJ) free energy: -386.929256 a.u.
M06-L SCF energy in solution: -387.08081211 a.u.
M06-L enthalpy in solution: -386.904072 a.u.
M06-L free energy in solution: -386.948583 a.u.

Cartesian coordinates

ATOM	X	Y	Z
C	-2.250691	0.468612	0.000029
C	-3.163197	1.402498	0.000857
H	-3.564142	1.812122	0.927666
H	-3.565081	1.813562	-0.924906
C	-1.335156	-0.478496	-0.000463
C	-1.772002	-1.930783	-0.000567
H	-2.861595	-2.004713	-0.000587
H	-1.390900	-2.455950	-0.885061
H	-1.391083	-2.455906	0.884053
C	0.115699	-0.158221	-0.000166
C	1.078192	-1.179568	0.000385
C	0.564305	1.175129	-0.000738
C	2.441630	-0.879568	0.000655
H	0.767950	-2.218729	0.000675
C	1.921875	1.473035	-0.000462
H	-0.168254	1.976552	-0.001348
C	2.871140	0.445872	0.000269
H	3.167062	-1.688534	0.001131
H	2.243336	2.511038	-0.000878
H	3.931939	0.679497	0.000465

2a

B3LYP-D3(BJ) SCF energy: -754.04615574 a.u.
B3LYP-D3(BJ) enthalpy: -753.834037 a.u.
B3LYP-D3(BJ) free energy: -753.886133 a.u.
M06-L SCF energy in solution: -754.06236775 a.u.
M06-L enthalpy in solution: -753.850249 a.u.
M06-L free energy in solution: -753.902345 a.u.

Cartesian coordinates

ATOM	X	Y	Z
Si	0.000044	-1.578172	-0.000119
H	-0.114117	-2.463633	1.194120

H	0.114324	-2.463497	-1.194447
C	1.538997	-0.507491	0.059913
C	1.561523	0.684483	0.805885
C	2.705798	-0.877370	-0.630150
C	2.709988	1.473646	0.867016
H	0.667255	1.005807	1.334250
C	3.857842	-0.091920	-0.571411
H	2.713841	-1.787286	-1.225797
C	3.861208	1.084984	0.178867
H	2.706921	2.392092	1.447829
H	4.749433	-0.394634	-1.113883
H	4.756017	1.699871	0.223488
C	-1.539016	-0.507654	-0.060036
C	-2.705532	-0.877216	0.630667
C	-1.561833	0.684011	-0.806503
C	-3.857575	-0.091749	0.572107
H	-2.713351	-1.786902	1.226667
C	-2.710294	1.473188	-0.867458
H	-0.667790	1.005096	-1.335396
C	-3.861227	1.084848	-0.178644
H	-4.748943	-0.394216	1.115083
H	-2.707448	2.391395	-1.448650
H	-4.756033	1.699750	-0.223122

12

B3LYP-D3(BJ) SCF energy: -2460.32789434 a.u.
B3LYP-D3(BJ) enthalpy: -2459.683931 a.u.
B3LYP-D3(BJ) free energy: -2459.795049 a.u.
M06-L SCF energy in solution: -2461.58768049 a.u.
M06-L enthalpy in solution: -2460.943717 a.u.
M06-L free energy in solution: -2461.054835 a.u.

Cartesian coordinates

ATOM	X	Y	Z
C	2.309074	1.091012	-0.419614
C	3.523458	1.650751	-0.833294
H	4.328006	1.001304	-1.160509
C	3.687646	3.036824	-0.842666
H	4.629889	3.464438	-1.172045
C	2.651350	3.881159	-0.436794
H	2.802715	4.954765	-0.458480
C	1.429256	3.356918	-0.003086
C	1.299751	1.967946	-0.010477
C	0.249828	4.172650	0.535358

C	-1.037562	3.470047	0.090242
C	-1.039962	2.073061	0.069704
C	-2.152225	1.297587	-0.274845
C	-3.332194	1.978749	-0.602099
H	-4.213536	1.416057	-0.887599
C	-3.367401	3.372682	-0.588597
H	-4.285941	3.889610	-0.849834
C	-2.230796	4.111711	-0.252353
H	-2.280809	5.194915	-0.259209
C	0.309502	4.134105	2.086504
H	0.287666	3.103706	2.453902
H	-0.545629	4.670630	2.512209
H	1.233269	4.604681	2.441044
C	0.299038	5.632561	0.068541
H	1.216437	6.117077	0.415288
H	-0.535730	6.200810	0.489100
H	0.256792	5.707687	-1.022735
C	3.424602	-1.457426	-1.116016
C	4.546418	-1.678871	-0.303840
H	4.510546	-1.417506	0.749554
C	5.701640	-2.240689	-0.845600
H	6.567582	-2.412978	-0.212190
C	5.744392	-2.584542	-2.199630
H	6.645242	-3.024307	-2.618891
C	4.628161	-2.371726	-3.010090
H	4.655655	-2.647262	-4.060550
C	3.467290	-1.814273	-2.471593
H	2.584453	-1.661036	-3.088203
C	1.828031	-1.181190	1.270980
C	1.206052	-2.398284	1.583658
H	0.756698	-2.990260	0.791084
C	1.142459	-2.839179	2.902886
H	0.649708	-3.777932	3.133531
C	1.679068	-2.057522	3.927834
H	1.615956	-2.394970	4.958565
C	2.283090	-0.836263	3.627402
H	2.696351	-0.221906	4.422901
C	2.361668	-0.400158	2.303869
H	2.834834	0.549309	2.072936
C	-3.516345	-1.070083	-1.164356
C	-4.701655	-1.145057	-0.415245
H	-4.687183	-0.900494	0.642601
C	-5.891493	-1.539904	-1.023867
H	-6.805442	-1.596963	-0.438907

C	-5.907786	-1.863194	-2.384052
H	-6.836087	-2.173255	-2.856171
C	-4.732029	-1.793966	-3.131801
H	-4.740045	-2.052378	-4.186941
C	-3.536390	-1.401183	-2.526337
H	-2.610027	-1.358562	-3.095149
C	-2.020744	-1.161909	1.274648
C	-2.342159	-2.516970	1.458163
H	-2.585356	-3.134970	0.598222
C	-2.378023	-3.069460	2.736621
H	-2.643163	-4.115682	2.863188
C	-2.074514	-2.282241	3.849467
H	-2.097264	-2.713359	4.846342
C	-1.734231	-0.941411	3.672654
H	-1.486244	-0.324454	4.531618
C	-1.709903	-0.381973	2.395180
H	-1.450687	0.663257	2.275617
O	0.114025	1.385809	0.390700
P	1.880985	-0.692206	-0.494737
P	-1.921493	-0.522101	-0.445620
Cu	-0.035902	-1.012077	-1.842779
H	-0.130244	-1.343364	-3.394689

13-ts

B3LYP-D3(BJ) SCF energy: -2847.39451154 a.u.

B3LYP-D3(BJ) enthalpy: -2846.573499 a.u.

B3LYP-D3(BJ) free energy: -2846.706765 a.u.

M06-L SCF energy in solution: -2848.66272143 a.u.

M06-L enthalpy in solution: -2847.841709 a.u.

M06-L free energy in solution: -2847.974975 a.u.

Imaginary frequency: -669.3983 cm^{-1}

Cartesian coordinates

ATOM	X	Y	Z
C	-2.923935	0.781643	-0.916370
C	-3.886680	1.441418	-1.689128
H	-3.923639	2.525038	-1.689605
C	-4.784446	0.708328	-2.467810
H	-5.522508	1.229305	-3.070438
C	-4.747301	-0.687578	-2.476836
H	-5.459645	-1.234103	-3.085015
C	-3.805805	-1.381724	-1.709281
C	-2.912720	-0.616355	-0.959784
C	-3.717056	-2.904266	-1.569480

C	-2.232856	-3.266694	-1.457607
C	-1.404932	-2.392624	-0.747469
C	-0.036804	-2.605592	-0.550821
C	0.510816	-3.790726	-1.060448
H	1.567955	-3.990346	-0.926739
C	-0.289238	-4.691661	-1.761905
H	0.147281	-5.603237	-2.158966
C	-1.644919	-4.426684	-1.969225
H	-2.244169	-5.134338	-2.531479
C	-4.414479	-3.304114	-0.240777
H	-3.953723	-2.796127	0.611677
H	-4.335841	-4.385398	-0.082302
H	-5.474490	-3.028728	-0.272657
C	-4.401548	-3.631429	-2.733054
H	-5.462388	-3.368673	-2.780160
H	-4.351049	-4.715375	-2.594717
H	-3.938192	-3.381113	-3.692802
C	-1.947647	3.364137	-0.091001
C	-3.135677	3.927229	0.399171
H	-3.867375	3.295421	0.894551
C	-3.372371	5.293689	0.262200
H	-4.293498	5.724778	0.644676
C	-2.421279	6.109075	-0.358512
H	-2.605781	7.174883	-0.461521
C	-1.230576	5.557133	-0.831717
H	-0.481945	6.190977	-1.298474
C	-0.989886	4.188685	-0.694925
H	-0.049176	3.756918	-1.021957
C	-2.044758	1.189906	1.783727
C	-1.070061	1.408541	2.766926
H	-0.086382	1.762266	2.470525
C	-1.357081	1.155873	4.106343
H	-0.593819	1.321555	4.859577
C	-2.610189	0.662114	4.474139
H	-2.827226	0.452139	5.517853
C	-3.579341	0.428791	3.497799
H	-4.554556	0.040042	3.778802
C	-3.300644	0.696037	2.156705
H	-4.057739	0.513803	1.400219
C	2.677973	-1.857684	-0.097624
C	3.405531	-2.624632	0.821500
H	2.950045	-2.921492	1.760344
C	4.723168	-2.988402	0.541808
H	5.285784	-3.571492	1.265719

C	5.319377	-2.599296	-0.659064
H	6.350644	-2.870041	-0.866802
C	4.592498	-1.851434	-1.586242
H	5.057030	-1.527440	-2.512619
C	3.279859	-1.479714	-1.306129
H	2.735354	-0.849908	-2.001726
C	0.747180	-1.475927	2.010931
C	1.606789	-0.766654	2.866212
H	2.386806	-0.138118	2.447284
C	1.470804	-0.870885	4.247678
H	2.152470	-0.329422	4.897904
C	0.453226	-1.657352	4.794257
H	0.338849	-1.729061	5.872189
C	-0.421757	-2.339581	3.950425
H	-1.223197	-2.942229	4.368147
C	-0.271176	-2.258027	2.564906
H	-0.946798	-2.806371	1.917610
O	-1.934701	-1.239234	-0.208841
P	-1.581861	1.575053	0.051074
P	0.970550	-1.261624	0.203003
Cu	0.569284	0.949840	-0.490593
H	1.508824	2.246066	-0.103077
C	1.895068	1.748122	-1.926544
C	0.766711	1.462258	-2.660273
H	-0.077998	2.142985	-2.729903
H	0.759153	0.613902	-3.342882
C	3.229149	1.877066	-2.070539
C	3.703018	2.249491	-3.468281
H	2.853877	2.320645	-4.149346
H	4.225794	3.215592	-3.474893
H	4.403593	1.502626	-3.866281
C	4.262902	1.718806	-1.031955
C	3.993671	1.192222	0.247912
C	5.605598	2.049766	-1.312404
C	5.001590	1.011171	1.188941
H	2.979437	0.918308	0.490588
C	6.616899	1.860715	-0.372941
H	5.871086	2.459968	-2.279721
C	6.324456	1.336215	0.886410
H	4.754136	0.586572	2.159108
H	7.639749	2.124500	-0.629835
H	7.114559	1.175436	1.614853

14-ts

B3LYP-D3(BJ) SCF energy: -2847.39769588 a.u.
B3LYP-D3(BJ) enthalpy: -2846.576759 a.u.
B3LYP-D3(BJ) free energy: -2846.708393 a.u.
M06-L SCF energy in solution: -2848.66553678 a.u.
M06-L enthalpy in solution: -2847.844600 a.u.
M06-L free energy in solution: -2847.976234 a.u.
Imaginary frequency: -701.7300 cm^{-1}

Cartesian coordinates

ATOM	X	Y	Z
C	-2.607263	1.157828	-1.303221
C	-3.270735	2.014049	-2.189243
H	-3.246725	3.084836	-2.019883
C	-3.948215	1.492791	-3.293497
H	-4.453030	2.165334	-3.980620
C	-3.989861	0.115523	-3.520378
H	-4.530763	-0.265080	-4.379782
C	-3.346943	-0.771515	-2.650212
C	-2.659760	-0.214098	-1.572208
C	-3.394783	-2.299757	-2.738020
C	-2.028132	-2.826197	-2.288915
C	-1.381000	-2.146857	-1.252091
C	-0.134788	-2.521106	-0.738343
C	0.457525	-3.678051	-1.263729
H	1.422017	-4.000578	-0.887957
C	-0.169306	-4.390162	-2.284923
H	0.302974	-5.280290	-2.689639
C	-1.393584	-3.960252	-2.802340
H	-1.854066	-4.519025	-3.609644
C	-4.463051	-2.802336	-1.728970
H	-4.232057	-2.466097	-0.713839
H	-4.499321	-3.897405	-1.730512
H	-5.451535	-2.416648	-2.002222
C	-3.765921	-2.785574	-4.144275
H	-4.751389	-2.408949	-4.433488
H	-3.824917	-3.877500	-4.173178
H	-3.035124	-2.456677	-4.890106
C	-1.808433	3.475300	0.229697
C	-3.063953	4.070526	0.423152
H	-3.946781	3.446383	0.527213
C	-3.178498	5.457676	0.494147
H	-4.153289	5.913524	0.644208
C	-2.039200	6.260053	0.381913
H	-2.129957	7.341156	0.440999

C	-0.785476	5.672709	0.208864
H	0.103610	6.292972	0.139310
C	-0.668110	4.283562	0.136805
H	0.306140	3.814271	0.041554
C	-2.553356	1.024232	1.551470
C	-1.887158	0.886250	2.776347
H	-0.832370	1.138708	2.836689
C	-2.567142	0.406215	3.894102
H	-2.038960	0.291721	4.834799
C	-3.911495	0.043845	3.794867
H	-4.436416	-0.343495	4.663806
C	-4.577894	0.170871	2.574905
H	-5.623417	-0.113954	2.491958
C	-3.903238	0.662433	1.456958
H	-4.423500	0.752202	0.508174
C	2.424282	-2.042400	0.407638
C	2.958206	-2.917873	1.361615
H	2.336565	-3.276418	2.175114
C	4.293032	-3.319577	1.276135
H	4.700618	-3.993701	2.024517
C	5.100770	-2.855459	0.236744
H	6.141981	-3.160054	0.178558
C	4.570488	-1.993573	-0.726585
H	5.198250	-1.612331	-1.525273
C	3.241037	-1.587306	-0.639522
H	2.837998	-0.899029	-1.374168
C	0.075044	-1.922468	2.095692
C	0.679232	-1.362584	3.233665
H	1.506767	-0.669004	3.114445
C	0.221202	-1.691797	4.506540
H	0.705740	-1.265163	5.380568
C	-0.868881	-2.553941	4.658107
H	-1.235192	-2.799677	5.650962
C	-1.490331	-3.089150	3.530680
H	-2.344461	-3.751218	3.641679
C	-1.014691	-2.784200	2.253814
H	-1.493387	-3.216573	1.381418
O	-1.972325	-1.032397	-0.696959
P	-1.580003	1.661808	0.130731
P	0.706807	-1.400997	0.457137
Cu	0.584561	0.933493	0.186314
H	1.394058	1.888583	1.258076
C	2.481232	1.678940	-0.274177
C	1.818317	1.774960	-1.476369

H	1.261707	2.663187	-1.765335
H	2.019166	1.054509	-2.268230
C	3.681278	1.428947	0.281723
C	3.840943	0.935670	1.695458
H	2.922015	1.148378	2.250314
H	4.012645	-0.147094	1.737222
H	4.687908	1.424444	2.191781
C	4.915446	1.620998	-0.520996
C	4.950519	2.404760	-1.690943
C	6.112898	0.991778	-0.127852
C	6.120799	2.549533	-2.428119
H	4.044437	2.903517	-2.015622
C	7.283984	1.129295	-0.873818
H	6.123895	0.367412	0.757943
C	7.298155	1.909217	-2.028885
H	6.115547	3.169140	-3.321412
H	8.188061	0.621802	-0.546482
H	8.209686	2.019234	-2.609823

15-ts

B3LYP-D3(BJ) SCF energy: -2847.40560622 a.u.
B3LYP-D3(BJ) enthalpy: -2846.584174 a.u.
B3LYP-D3(BJ) free energy: -2846.714140 a.u.
M06-L SCF energy in solution: -2848.66554299 a.u.
M06-L enthalpy in solution: -2847.844111 a.u.
M06-L free energy in solution: -2847.974077 a.u.
Imaginary frequency: -743.5796 cm^{-1}

Cartesian coordinates

ATOM	X	Y	Z
C	-2.136314	-0.118388	1.007582
C	-3.477171	-0.470093	1.175412
H	-4.242861	0.035439	0.600709
C	-3.825777	-1.510083	2.037769
H	-4.870081	-1.784978	2.147936
C	-2.847436	-2.221922	2.730122
H	-3.144325	-3.035879	3.382265
C	-1.492866	-1.902719	2.582556
C	-1.182732	-0.845785	1.729532
C	-0.339433	-2.572501	3.333752
C	0.881694	-2.577174	2.407751
C	1.077800	-1.468348	1.579185
C	2.188086	-1.307172	0.741536
C	3.148642	-2.326678	0.747780

H	4.013677	-2.252246	0.099628
C	2.979301	-3.452588	1.552305
H	3.725845	-4.240973	1.537971
C	1.856045	-3.578673	2.371720
H	1.744816	-4.464384	2.987112
C	0.001654	-1.698324	4.570712
H	0.257717	-0.678116	4.268821
H	0.853779	-2.122087	5.113738
H	-0.858940	-1.648985	5.247030
C	-0.707490	-3.985694	3.803438
H	-1.566078	-3.955519	4.480164
H	0.117736	-4.436212	4.362553
H	-0.953788	-4.637089	2.958729
C	-2.955369	1.918118	-0.906048
C	-3.959548	2.497131	-0.115598
H	-3.875219	2.474958	0.966865
C	-5.058651	3.109722	-0.714195
H	-5.836164	3.549733	-0.095801
C	-5.154602	3.166944	-2.107609
H	-6.011488	3.646328	-2.572942
C	-4.142318	2.622982	-2.898639
H	-4.204197	2.681292	-3.981723
C	-3.044497	2.002083	-2.300497
H	-2.242982	1.587416	-2.903921
C	-0.858034	2.446601	0.925215
C	-0.057792	3.440860	0.346241
H	0.220815	3.359761	-0.700953
C	0.388525	4.516763	1.109365
H	1.013865	5.277307	0.653146
C	0.059733	4.598927	2.463770
H	0.419566	5.432126	3.060985
C	-0.718705	3.601561	3.051151
H	-0.970963	3.657009	4.106856
C	-1.180643	2.530747	2.284517
H	-1.791072	1.759645	2.744091
C	3.673112	-0.237785	-1.472708
C	4.974900	-0.099793	-0.964325
H	5.121116	0.240261	0.056404
C	6.076253	-0.389585	-1.767186
H	7.080539	-0.279964	-1.367266
C	5.888951	-0.817454	-3.085155
H	6.749141	-1.041050	-3.710149
C	4.598460	-0.948504	-3.597644
H	4.447305	-1.271797	-4.623730

C	3.492417	-0.655890	-2.796754
H	2.488386	-0.741258	-3.198351
C	2.740252	1.540959	0.537093
C	3.297094	2.632366	-0.148097
H	3.403282	2.593148	-1.228569
C	3.735638	3.754919	0.549884
H	4.178151	4.586598	0.008380
C	3.604852	3.811190	1.939253
H	3.941570	4.688622	2.484160
C	3.029939	2.740365	2.622267
H	2.911182	2.782400	3.701079
C	2.603290	1.608026	1.927651
H	2.166441	0.779820	2.472512
O	0.134942	-0.463883	1.552711
P	-1.478232	1.114265	-0.177071
P	2.184096	0.096084	-0.454766
Cu	0.137329	0.232037	-1.586331
H	-0.036922	0.640994	-3.156953
C	-0.101169	-1.114268	-3.278484
C	-0.486067	-1.965139	-2.242098
C	0.245198	-1.174450	-4.559971
C	-1.898079	-2.120302	-1.887088
C	-2.323893	-3.088814	-0.951944
C	-2.901205	-1.340324	-2.506374
C	-3.675767	-3.278657	-0.676140
H	-1.590182	-3.703134	-0.441658
C	-4.246695	-1.515335	-2.207341
H	-2.603790	-0.600880	-3.240197
C	-4.651266	-2.496981	-1.296942
H	-3.967009	-4.038265	0.044917
H	-4.985078	-0.885032	-2.697049
H	-5.704081	-2.643957	-1.072507
C	0.519406	-2.977276	-1.721739
H	0.294563	-3.981959	-2.113326
H	1.532087	-2.717145	-2.038402
H	0.526541	-3.053118	-0.629538
H	0.157100	-2.120242	-5.095157
H	0.613631	-0.313240	-5.108298

16

B3LYP-D3(BJ) SCF energy: -2847.47612995 a.u.

B3LYP-D3(BJ) enthalpy: -2846.648035 a.u.

B3LYP-D3(BJ) free energy: -2846.781761 a.u.

M06-L SCF energy in solution: -2848.73621741 a.u.

M06-L enthalpy in solution: -2847.908122 a.u.
M06-L free energy in solution: -2848.041848 a.u.

Cartesian coordinates

ATOM	X	Y	Z
C	-2.199725	1.719174	-0.336244
C	-2.825278	2.963340	-0.199124
H	-3.529708	3.122359	0.609998
C	-2.529835	3.995455	-1.090699
H	-3.009652	4.962320	-0.972503
C	-1.630134	3.795919	-2.140936
H	-1.426253	4.611634	-2.825557
C	-0.998576	2.560806	-2.319159
C	-1.292133	1.564334	-1.388082
C	-0.080720	2.191059	-3.489115
C	0.991735	1.236172	-2.956045
C	0.624021	0.334548	-1.955388
C	1.506784	-0.573982	-1.361760
C	2.817161	-0.615205	-1.854933
H	3.538306	-1.290265	-1.410741
C	3.208419	0.244999	-2.879859
H	4.230048	0.211947	-3.245413
C	2.311814	1.172981	-3.412937
H	2.650001	1.853892	-4.185963
C	-0.933530	1.426269	-4.537145
H	-1.390991	0.536225	-4.094103
H	-0.306170	1.108703	-5.377371
H	-1.733645	2.070679	-4.918042
C	0.535766	3.431437	-4.147648
H	-0.246068	4.090993	-4.535204
H	1.158931	3.147652	-5.000543
H	1.150179	3.996491	-3.439545
C	-3.609971	0.925972	2.028116
C	-4.987984	0.943192	1.765792
H	-5.361605	0.554196	0.823625
C	-5.875264	1.453684	2.712322
H	-6.941525	1.462519	2.503440
C	-5.395172	1.952071	3.926334
H	-6.088716	2.348816	4.662570
C	-4.025402	1.935214	4.193895
H	-3.648809	2.317571	5.138419
C	-3.134263	1.420565	3.251228
H	-2.068320	1.397537	3.454383
C	-3.358638	-0.904665	-0.162344

C	-3.608535	-2.154236	0.423581
H	-3.208203	-2.376780	1.408825
C	-4.356041	-3.114868	-0.251967
H	-4.539716	-4.078505	0.213272
C	-4.845982	-2.849192	-1.532425
H	-5.420752	-3.602916	-2.063293
C	-4.585795	-1.616158	-2.130273
H	-4.961943	-1.403254	-3.127371
C	-3.850684	-0.645386	-1.447892
H	-3.667206	0.317045	-1.913826
C	2.516234	-2.283278	0.671819
C	2.855354	-3.588758	0.289950
H	2.155553	-4.186606	-0.284263
C	4.096275	-4.118708	0.645352
H	4.351783	-5.133109	0.351146
C	5.011847	-3.345548	1.363227
H	5.981624	-3.758691	1.626863
C	4.682531	-2.040825	1.735567
H	5.391386	-1.418659	2.271905
C	3.434990	-1.515929	1.402260
H	3.183105	-0.499856	1.678331
C	-0.094985	-2.832996	-0.411322
C	-0.455886	-3.810004	0.530903
H	-0.082208	-3.740763	1.549601
C	-1.266107	-4.881341	0.162663
H	-1.524164	-5.639749	0.896723
C	-1.751626	-4.973834	-1.143691
H	-2.392940	-5.802595	-1.429581
C	-1.421394	-3.991098	-2.075846
H	-1.809410	-4.048897	-3.088556
C	-0.591021	-2.929146	-1.715847
H	-0.326216	-2.179251	-2.452759
O	-0.669192	0.334798	-1.471929
P	-2.382639	0.303734	0.817869
P	0.965155	-1.452944	0.161601
Cu	-0.133314	0.055840	1.605405
H	2.535664	0.953041	3.243450
C	1.928694	1.569272	2.578387
C	0.502789	1.533930	2.857426
H	0.268308	1.288016	3.897218
H	-0.061109	2.413828	2.529280
C	2.572535	2.160378	1.525973
C	1.781473	2.921359	0.489919
H	0.931053	3.440829	0.943375

H	2.394036	3.671449	-0.022618
H	1.365038	2.260848	-0.285413
C	4.000564	1.922167	1.286935
C	4.907610	1.639594	2.334091
C	4.524262	1.919136	-0.024182
C	6.240645	1.330101	2.080300
H	4.559215	1.687814	3.361473
C	5.859287	1.610087	-0.276493
H	3.863971	2.124602	-0.859058
C	6.730334	1.304495	0.770307
H	6.909306	1.126315	2.913858
H	6.219068	1.601003	-1.303189
H	7.771989	1.066664	0.573865

17

B3LYP-D3(BJ) SCF energy: -2847.47929942 a.u.
B3LYP-D3(BJ) enthalpy: -2846.650529 a.u.
B3LYP-D3(BJ) free energy: -2846.780483 a.u.
M06-L SCF energy in solution: -2848.73529539 a.u.
M06-L enthalpy in solution: -2847.906525 a.u.
M06-L free energy in solution: -2848.036479 a.u.

Cartesian coordinates

ATOM	X	Y	Z
C	-2.393878	-0.921884	0.433793
C	-3.504662	-1.773056	0.400824
H	-4.326295	-1.558032	-0.273869
C	-3.542951	-2.900531	1.223558
H	-4.407077	-3.557582	1.195656
C	-2.472496	-3.202920	2.069251
H	-2.514354	-4.100154	2.676466
C	-1.347298	-2.375811	2.126930
C	-1.357422	-1.242011	1.315937
C	-0.130267	-2.591923	3.029798
C	1.105402	-2.066744	2.288922
C	0.961643	-0.911935	1.518363
C	2.021626	-0.240825	0.899896
C	3.295547	-0.807819	1.028890
H	4.142074	-0.347578	0.534946
C	3.471094	-1.984198	1.755292
H	4.460718	-2.424207	1.831209
C	2.389762	-2.605172	2.383769
H	2.554225	-3.517823	2.944552
C	-0.322970	-1.726052	4.304618

H	-0.457736	-0.670675	4.047762
H	0.552619	-1.812625	4.957295
H	-1.209048	-2.058381	4.857117
C	0.029569	-4.060661	3.440344
H	-0.854270	-4.408647	3.983327
H	0.880404	-4.178397	4.118027
H	0.184309	-4.696879	2.565225
C	-3.573057	0.467264	-1.778020
C	-4.837457	0.954942	-1.418882
H	-4.980341	1.420382	-0.448385
C	-5.906664	0.848593	-2.308099
H	-6.883880	1.230721	-2.025990
C	-5.721771	0.254301	-3.559027
H	-6.556370	0.173468	-4.249882
C	-4.463281	-0.227308	-3.924212
H	-4.314387	-0.681982	-4.899463
C	-3.389596	-0.116128	-3.040414
H	-2.401753	-0.470521	-3.322683
C	-2.452023	1.946664	0.441023
C	-1.953529	3.198735	0.054744
H	-1.371227	3.284930	-0.858658
C	-2.178440	4.321721	0.846702
H	-1.776374	5.283267	0.544980
C	-2.885943	4.201456	2.044239
H	-3.046962	5.074924	2.669696
C	-3.369986	2.955722	2.445026
H	-3.913732	2.857239	3.380695
C	-3.156623	1.831453	1.646351
H	-3.531838	0.862403	1.960640
C	3.217854	1.769249	-0.835884
C	4.298901	2.127546	-0.014250
H	4.199404	2.060212	1.064945
C	5.490480	2.579751	-0.576550
H	6.324632	2.847166	0.066091
C	5.609007	2.697826	-1.964751
H	6.538378	3.052277	-2.401679
C	4.529870	2.372832	-2.786131
H	4.612509	2.476405	-3.864254
C	3.337726	1.912043	-2.223000
H	2.491739	1.662206	-2.854126
C	1.273985	2.580644	1.035858
C	1.484199	3.897832	0.595043
H	1.892425	4.077087	-0.395343
C	1.198450	4.979851	1.424853

H	1.379919	5.991175	1.070793
C	0.685271	4.765386	2.705254
H	0.458874	5.607969	3.352508
C	0.461095	3.461396	3.145351
H	0.055803	3.283219	4.137360
C	0.754216	2.375720	2.320056
H	0.576930	1.371831	2.682647
O	-0.295849	-0.364441	1.338416
P	-2.121545	0.523899	-0.663509
P	1.611382	1.210449	-0.153440
Cu	-0.018625	0.412514	-1.706139
H	2.703843	-0.445183	-3.415592
C	1.915290	-1.078543	-3.008076
C	0.576452	-0.619165	-3.365632
H	0.599439	0.050142	-4.231814
H	-0.165533	-1.404558	-3.532134
C	2.401463	-2.098349	-2.231180
C	3.908101	-2.235884	-2.131683
H	4.404386	-1.378801	-2.598157
H	4.251369	-2.289592	-1.089925
H	4.283227	-3.141503	-2.631569
C	1.596511	-3.083605	-1.504608
C	0.192707	-2.985294	-1.349719
C	2.211750	-4.202402	-0.892077
C	-0.541468	-3.953094	-0.671497
H	-0.334814	-2.135517	-1.755888
C	1.476776	-5.169018	-0.212713
H	3.286307	-4.324184	-0.960462
C	0.088045	-5.061859	-0.104327
H	-1.616758	-3.833477	-0.584826
H	1.993675	-6.018713	0.228005
H	-0.491166	-5.827019	0.405996

18

B3LYP-D3(BJ) SCF energy: -2847.47035063 a.u.
B3LYP-D3(BJ) enthalpy: -2846.642253 a.u.
B3LYP-D3(BJ) free energy: -2846.773379 a.u.
M06-L SCF energy in solution: -2848.72519741 a.u.
M06-L enthalpy in solution: -2847.897100 a.u.
M06-L free energy in solution: -2848.028226 a.u.

Cartesian coordinates

ATOM	X	Y	Z
C	2.149636	0.765513	-0.944070

C	3.516433	0.759043	-1.237338
H	4.227804	1.081061	-0.485232
C	3.961788	0.300521	-2.477414
H	5.024852	0.292393	-2.697691
C	3.056981	-0.179089	-3.425810
H	3.431946	-0.559213	-4.369630
C	1.680886	-0.182388	-3.169593
C	1.270255	0.324930	-1.938514
C	0.602800	-0.657630	-4.148275
C	-0.560491	-1.234830	-3.331461
C	-0.879997	-0.622430	-2.117763
C	-1.982958	-0.971270	-1.327950
C	-2.782395	-2.029077	-1.776481
H	-3.625130	-2.355757	-1.178674
C	-2.476955	-2.685115	-2.968923
H	-3.097119	-3.512076	-3.301137
C	-1.382231	-2.289979	-3.740456
H	-1.172235	-2.811871	-4.667194
C	0.073678	0.578145	-4.924180
H	-0.319568	1.333003	-4.236183
H	-0.730137	0.283468	-5.607783
H	0.882512	1.034539	-5.505544
C	1.157517	-1.680435	-5.148988
H	1.966582	-1.243252	-5.740744
H	0.383064	-1.989137	-5.856931
H	1.540064	-2.570990	-4.640115
C	2.915161	1.461719	1.724307
C	3.405454	2.751190	1.969059
H	2.890201	3.614042	1.559655
C	4.556066	2.925340	2.739640
H	4.929258	3.927781	2.930689
C	5.227901	1.817269	3.260005
H	6.124909	1.957119	3.856949
C	4.743154	0.530495	3.014478
H	5.260639	-0.337468	3.413175
C	3.585878	0.351475	2.258930
H	3.213934	-0.649880	2.067608
C	0.652858	2.766331	0.490625
C	-0.343978	3.143744	1.400431
H	-0.650099	2.448460	2.177558
C	-0.957937	4.389540	1.296238
H	-1.739150	4.666218	1.996445
C	-0.597041	5.261276	0.267691
H	-1.090002	6.224800	0.175399

C	0.384544	4.887266	-0.651338
H	0.663029	5.561752	-1.456525
C	1.010960	3.645793	-0.539309
H	1.776923	3.355613	-1.252054
C	-3.555950	-0.991460	1.122701
C	-4.862436	-0.965784	0.606276
H	-5.068628	-0.424697	-0.312516
C	-5.894038	-1.619260	1.276519
H	-6.901339	-1.599368	0.869782
C	-5.634157	-2.294853	2.473691
H	-6.441126	-2.802118	2.995221
C	-4.342811	-2.310261	2.999339
H	-4.132479	-2.828521	3.930329
C	-3.305437	-1.658445	2.328575
H	-2.300561	-1.681318	2.740478
C	-2.894308	1.499918	-0.074262
C	-3.719008	2.104544	0.888782
H	-3.964324	1.570850	1.801911
C	-4.246491	3.376652	0.675125
H	-4.893714	3.822275	1.425836
C	-3.947683	4.072924	-0.497044
H	-4.356417	5.065537	-0.663666
C	-3.114795	3.486678	-1.450119
H	-2.867662	4.023423	-2.361684
C	-2.591835	2.210819	-1.243482
H	-1.942964	1.776647	-1.993005
O	-0.073279	0.385291	-1.625734
P	1.431735	1.124777	0.707655
P	-2.153531	-0.144639	0.311474
Cu	-0.011763	-0.663172	1.185885
C	0.393199	-2.654364	1.580570
C	-0.556527	-3.139569	3.887407
C	1.755290	-2.794208	0.982743
C	1.944930	-2.691494	-0.415595
C	2.910727	-3.046895	1.753178
C	3.202537	-2.785923	-0.996291
H	1.081627	-2.510174	-1.049966
C	4.179781	-3.119690	1.171964
H	2.809524	-3.205542	2.822075
C	4.340801	-2.981379	-0.205225
H	3.300647	-2.682756	-2.072943
H	5.045010	-3.306510	1.804829
H	5.326263	-3.041913	-0.658576
C	-0.630783	-3.601904	0.951960

H	-0.434878	-4.648639	1.243107
H	-1.652438	-3.367230	1.264300
H	-0.613245	-3.556919	-0.140196
H	-1.349269	-3.794074	3.536476
H	-0.515600	-2.953333	4.956932
C	0.347696	-2.586479	3.046761
H	1.133570	-1.980119	3.504806

19-ts

B3LYP-D3(BJ) SCF energy: -2847.46751248 a.u.
B3LYP-D3(BJ) enthalpy: -2846.640458 a.u.
B3LYP-D3(BJ) free energy: -2846.769113 a.u.
M06-L SCF energy in solution: -2848.72531477 a.u.
M06-L enthalpy in solution: -2847.898260 a.u.
M06-L free energy in solution: -2848.026915 a.u.
Imaginary frequency: -69.1047 cm^{-1}

Cartesian coordinates

ATOM	X	Y	Z
C	-2.247182	0.173820	0.949483
C	-3.625958	0.014318	1.126933
H	-4.313394	0.674366	0.610525
C	-4.113735	-1.010534	1.937078
H	-5.185384	-1.134943	2.058621
C	-3.239741	-1.891144	2.575587
H	-3.644564	-2.696212	3.178546
C	-1.856482	-1.751659	2.435780
C	-1.403540	-0.705107	1.634785
C	-0.803498	-2.629531	3.116369
C	0.383729	-2.760223	2.155535
C	0.748803	-1.629913	1.420473
C	1.885118	-1.557858	0.605428
C	2.650282	-2.724351	0.473178
H	3.515063	-2.729549	-0.179357
C	2.285867	-3.887135	1.152070
H	2.879279	-4.788047	1.029287
C	1.172359	-3.902431	1.995417
H	0.920145	-4.813978	2.525413
C	-0.307582	-1.890408	4.389025
H	0.087283	-0.900765	4.140113
H	0.485898	-2.465532	4.878974
H	-1.135437	-1.758717	5.094481
C	-1.367670	-3.995129	3.526036
H	-2.191896	-3.873747	4.234664

H	-0.602927	-4.593009	4.030491
H	-1.734359	-4.555038	2.660706
C	-2.873085	2.195823	-1.003566
C	-3.138118	3.560417	-0.832904
H	-2.518189	4.154383	-0.170033
C	-4.199647	4.159257	-1.514268
H	-4.395226	5.219219	-1.377078
C	-5.008874	3.402463	-2.362047
H	-5.834762	3.871369	-2.889528
C	-4.752335	2.039353	-2.530738
H	-5.379180	1.441902	-3.186929
C	-3.686193	1.439763	-1.863668
H	-3.495462	0.378726	-1.989753
C	-0.764635	2.637448	0.908414
C	0.292427	3.424874	0.434022
H	0.703377	3.227439	-0.552348
C	0.826306	4.438167	1.227565
H	1.651786	5.035669	0.855411
C	0.326017	4.654742	2.512181
H	0.755166	5.432454	3.137676
C	-0.716358	3.862973	2.997853
H	-1.104340	4.026924	3.999548
C	-1.265390	2.861635	2.197234
H	-2.081386	2.250818	2.571141
C	3.681827	-0.302260	-1.319298
C	4.894465	-0.651323	-0.702191
H	4.933878	-0.778374	0.375520
C	6.047338	-0.820043	-1.465254
H	6.980141	-1.096602	-0.981856
C	6.006195	-0.623137	-2.849346
H	6.907420	-0.751970	-3.442177
C	4.812324	-0.248843	-3.464628
H	4.778487	-0.080386	-4.537140
C	3.653413	-0.086574	-2.701752
H	2.724635	0.220091	-3.172431
C	2.775398	1.212348	0.874647
C	3.597432	2.250775	0.407101
H	3.883298	2.283924	-0.640038
C	4.069933	3.229814	1.278077
H	4.715299	4.018432	0.900561
C	3.717677	3.196287	2.628629
H	4.082143	3.961256	3.308389
C	2.890854	2.176327	3.097511
H	2.604846	2.144495	4.144994

C	2.423905	1.188883	2.229746
H	1.785472	0.405502	2.615420
O	-0.045995	-0.502438	1.468229
P	-1.450970	1.358174	-0.207755
P	2.149298	-0.010022	-0.360335
Cu	0.118349	0.169553	-1.451859
C	-0.385699	-1.494725	-2.683106
C	0.254643	0.283426	-4.393820
C	-1.538851	-2.170734	-2.059742
C	-1.352050	-3.091215	-1.000321
C	-2.876170	-1.964057	-2.473115
C	-2.423765	-3.704992	-0.364918
H	-0.346492	-3.302044	-0.652807
C	-3.951687	-2.563819	-1.817866
H	-3.072580	-1.349451	-3.346143
C	-3.740912	-3.435656	-0.750083
H	-2.228376	-4.399377	0.448801
H	-4.963248	-2.366553	-2.167098
H	-4.576875	-3.907495	-0.242038
C	0.831752	-2.365646	-2.957460
H	0.626118	-3.100001	-3.754921
H	1.691903	-1.775251	-3.279964
H	1.153186	-2.928575	-2.076594
H	1.227505	-0.124737	-4.647850
H	-0.045762	1.190779	-4.906952
C	-0.619188	-0.360362	-3.553722
H	-1.599861	0.108193	-3.460865

20-ts

B3LYP-D3(BJ) SCF energy: -2847.46675194 a.u.

B3LYP-D3(BJ) enthalpy: -2846.639910 a.u.

B3LYP-D3(BJ) free energy: -2846.768878 a.u.

M06-L SCF energy in solution: -2848.72568227 a.u.

M06-L enthalpy in solution: -2847.898840 a.u.

M06-L free energy in solution: -2848.027808 a.u.

Imaginary frequency: -69.9369 cm^{-1}

Cartesian coordinates

ATOM	X	Y	Z
C	-2.012419	-1.509256	0.438753
C	-2.797311	-2.651137	0.242424
H	-3.603452	-2.630713	-0.482096
C	-2.526716	-3.817982	0.959245
H	-3.129742	-4.704654	0.788945
C	-1.495266	-3.855143	1.901185

H	-1.315482	-4.769452	2.455603
C	-0.698130	-2.729615	2.132532
C	-0.966766	-1.597089	1.363675
C	0.397008	-2.611642	3.196676
C	1.525254	-1.763980	2.601903
C	1.167471	-0.708914	1.761098
C	2.089395	0.140423	1.142242
C	3.445056	-0.063153	1.430493
H	4.192643	0.568453	0.965440
C	3.836141	-1.097034	2.278937
H	4.890690	-1.253797	2.483706
C	2.887334	-1.946068	2.850487
H	3.217748	-2.759708	3.486448
C	-0.200002	-1.844250	4.407770
H	-0.568581	-0.859660	4.104190
H	0.564768	-1.702689	5.179430
H	-1.035695	-2.406880	4.838811
C	0.892301	-3.983421	3.668820
H	0.070937	-4.560035	4.104430
H	1.649947	-3.872689	4.449878
H	1.324196	-4.560929	2.845484
C	-3.688544	-0.098798	-1.462555
C	-4.919535	-0.348676	-0.835759
H	-4.961599	-0.466438	0.243060
C	-6.086356	-0.434475	-1.591604
H	-7.035324	-0.632435	-1.100994
C	-6.037444	-0.256979	-2.977942
H	-6.949237	-0.321690	-3.565127
C	-4.820896	0.013656	-3.603504
H	-4.779970	0.163372	-4.678617
C	-3.648996	0.094847	-2.848081
H	-2.699937	0.317283	-3.325786
C	-2.635189	1.269449	0.780140
C	-2.665373	2.621217	0.414304
H	-2.341064	2.915936	-0.579784
C	-3.096572	3.587436	1.319629
H	-3.111964	4.631705	1.024635
C	-3.480887	3.217220	2.609635
H	-3.805817	3.973092	3.319118
C	-3.436432	1.875133	2.986845
H	-3.730073	1.580278	3.990771
C	-3.021309	0.903448	2.075216
H	-2.999593	-0.139437	2.373808
C	3.056473	1.952338	-0.838128

C	3.615626	3.190438	-0.495436
H	3.109938	3.839331	0.210766
C	4.826225	3.594171	-1.061621
H	5.248745	4.558431	-0.792697
C	5.495604	2.763095	-1.961158
H	6.437831	3.080818	-2.398838
C	4.951763	1.519788	-2.291397
H	5.470128	0.859627	-2.981163
C	3.737112	1.119124	-1.738707
H	3.334217	0.140862	-1.975364
C	0.822913	2.745319	0.790401
C	0.476174	3.895586	0.063099
H	0.603626	3.908768	-1.016647
C	-0.006491	5.026076	0.717298
H	-0.251928	5.916843	0.145479
C	-0.187665	5.007978	2.102509
H	-0.576554	5.884566	2.612836
C	0.117585	3.855485	2.825132
H	-0.037433	3.829457	3.899890
C	0.630539	2.731725	2.175501
H	0.886899	1.847415	2.748633
O	-0.166693	-0.478804	1.484038
P	-2.130627	0.057798	-0.509726
P	1.482834	1.311227	-0.142673
Cu	-0.033853	0.157924	-1.492209
H	1.705713	-0.019901	-3.487997
C	0.710475	-0.460988	-3.555533
C	-0.160558	0.183572	-4.400268
H	0.153120	1.069175	-4.942657
H	-1.142254	-0.214759	-4.633683
C	0.451648	-1.564740	-2.657195
C	-0.796113	-2.399164	-2.905482
H	-1.673749	-1.779939	-3.112238
H	-0.663360	-3.066711	-3.774635
H	-1.048870	-3.029205	-2.049093
C	1.590260	-2.257006	-2.022633
C	2.916733	-2.160357	-2.501142
C	1.398183	-3.062457	-0.874780
C	3.987753	-2.747854	-1.824875
H	3.107025	-1.633923	-3.432073
C	2.461558	-3.664495	-0.215988
H	0.395873	-3.181692	-0.477510
C	3.775270	-3.497934	-0.669477
H	4.994270	-2.634990	-2.222594

H	2.267446	-4.259001	0.673484
H	4.606773	-3.958322	-0.143607

21-ts

B3LYP-D3(BJ) SCF energy: -3601.53147473 a.u.

B3LYP-D3(BJ) enthalpy: -3600.489424 a.u.

B3LYP-D3(BJ) free energy: -3600.645308 a.u.

M06-L SCF energy in solution: -3602.79144066 a.u.

M06-L enthalpy in solution: -3601.749390 a.u.

M06-L free energy in solution: -3601.905274 a.u.

Imaginary frequency: -93.6064 cm^{-1}

Cartesian coordinates

ATOM	X	Y	Z
C	0.753036	-3.253768	-0.080167
C	0.762570	-4.517450	-0.681109
H	-0.175632	-4.987260	-0.955700
C	1.975033	-5.150852	-0.955915
H	1.977904	-6.128079	-1.429252
C	3.186326	-4.535531	-0.633051
H	4.115720	-5.042163	-0.867848
C	3.214315	-3.282982	-0.010418
C	1.984454	-2.681433	0.258306
C	4.477941	-2.550156	0.443784
C	4.230299	-1.047239	0.276390
C	2.949572	-0.557214	0.547721
C	2.618071	0.802206	0.524422
C	3.635929	1.704563	0.187596
H	3.417308	2.761819	0.122929
C	4.917123	1.248159	-0.110317
H	5.681291	1.957457	-0.407418
C	5.211727	-0.113395	-0.066409
H	6.214787	-0.446145	-0.307478
C	4.684228	-2.839250	1.955132
H	3.810217	-2.528956	2.535879
H	5.557510	-2.293540	2.328853
H	4.840687	-3.911313	2.118889
C	5.718601	-3.015716	-0.329220
H	5.887845	-4.086073	-0.180880
H	6.614073	-2.503230	0.033176
H	5.618127	-2.823322	-1.402268
C	-2.070630	-3.129615	-0.656471
C	-3.186234	-3.604047	0.043843
H	-3.225091	-3.511970	1.123899

C	-4.253425	-4.184835	-0.644846
H	-5.116798	-4.543989	-0.091966
C	-4.213742	-4.302054	-2.034162
H	-5.047016	-4.749596	-2.568063
C	-3.102196	-3.833288	-2.739133
H	-3.069625	-3.910471	-3.821784
C	-2.040702	-3.245818	-2.056254
H	-1.187629	-2.869876	-2.613526
C	-1.075474	-2.355727	1.954292
C	-1.792420	-1.325553	2.578475
H	-2.092678	-0.455556	2.005718
C	-2.105682	-1.411545	3.933329
H	-2.651428	-0.602209	4.406280
C	-1.689320	-2.515472	4.678685
H	-1.922620	-2.575700	5.738112
C	-0.963251	-3.538458	4.064904
H	-0.634184	-4.396870	4.644123
C	-0.660583	-3.463278	2.705429
H	-0.099427	-4.260532	2.227532
C	0.964998	3.140868	0.612076
C	1.602914	3.928065	1.584436
H	2.027613	3.456312	2.465007
C	1.687093	5.309183	1.425834
H	2.182280	5.910188	2.183275
C	1.133675	5.920221	0.296219
H	1.198731	6.997846	0.174862
C	0.489369	5.145232	-0.666594
H	0.040066	5.612411	-1.538215
C	0.401165	3.761180	-0.505128
H	-0.128582	3.167842	-1.238665
C	0.565455	1.153150	2.586495
C	-0.510929	1.882219	3.117712
H	-1.098378	2.534085	2.480855
C	-0.824504	1.781749	4.470473
H	-1.658360	2.355947	4.863256
C	-0.079499	0.944846	5.304621
H	-0.328309	0.862345	6.359068
C	0.982079	0.209763	4.777400
H	1.562176	-0.447739	5.418758
C	1.307906	0.314303	3.424233
H	2.140306	-0.255200	3.029367
O	1.931526	-1.441723	0.858505
P	-0.724067	-2.203577	0.165707
P	0.870072	1.318801	0.782010

Cu	-0.691735	0.062650	-0.380727
H	0.219513	2.153235	-3.077043
C	0.243242	1.080893	-2.889517
C	-1.090934	0.503186	-2.653229
H	-1.796805	0.852757	-3.413233
H	-1.126982	-0.590222	-2.678286
C	1.463094	0.481038	-2.900109
C	1.607359	-1.016513	-2.770401
H	0.696856	-1.479541	-2.390430
H	1.832037	-1.489063	-3.737337
H	2.418785	-1.286564	-2.087787
C	2.709570	1.254288	-3.079009
C	2.774674	2.649275	-2.876852
C	3.911248	0.610163	-3.434975
C	3.962247	3.355706	-3.031166
H	1.891275	3.188702	-2.559997
C	5.103218	1.316548	-3.589043
H	3.917281	-0.461665	-3.598737
C	5.141405	2.696006	-3.389264
H	3.968205	4.428713	-2.855764
H	6.007801	0.781214	-3.867269
H	6.070236	3.247368	-3.506565
Si	-2.543254	1.474801	-1.261541
H	-2.411644	0.154886	-0.291899
H	-2.184512	2.739743	-2.042103
C	-3.001521	2.439542	0.351506
C	-3.615883	1.771556	1.424878
C	-2.736815	3.806041	0.514027
C	-3.949135	2.430274	2.607888
H	-3.840757	0.711317	1.322307
C	-3.029963	4.473427	1.707349
H	-2.282266	4.356873	-0.304653
C	-3.638747	3.785766	2.759008
H	-4.438401	1.890450	3.415640
H	-2.785395	5.527460	1.815405
H	-3.874631	4.301722	3.686487
C	-4.114397	0.836472	-2.090887
C	-4.573320	-0.477291	-1.900319
C	-4.860526	1.675574	-2.935024
C	-5.731965	-0.936816	-2.527880
H	-4.015333	-1.150358	-1.256876
C	-6.022926	1.224440	-3.562648
H	-4.525856	2.696754	-3.103459
C	-6.461149	-0.085791	-3.360370

H	-6.060403	-1.960130	-2.366685
H	-6.585747	1.893015	-4.209571
H	-7.364945	-0.440693	-3.849534

22-ts

B3LYP-D3(BJ) SCF energy: -3601.53400669 a.u.

B3LYP-D3(BJ) enthalpy: -3600.492120 a.u.

B3LYP-D3(BJ) free energy: -3600.647917 a.u.

M06-L SCF energy in solution: -3602.79042417 a.u.

M06-L enthalpy in solution: -3601.748537 a.u.

M06-L free energy in solution: -3601.904334 a.u.

Imaginary frequency: -107.4839 cm^{-1}

Cartesian coordinates

ATOM	X	Y	Z
C	-2.324816	2.015166	0.791328
C	-3.006886	3.236391	0.754841
H	-2.445897	4.164311	0.778967
C	-4.399638	3.256216	0.669310
H	-4.923138	4.207218	0.640395
C	-5.128115	2.065322	0.613160
H	-6.208948	2.107675	0.533289
C	-4.479484	0.828208	0.654425
C	-3.089018	0.846989	0.758024
C	-5.165241	-0.540342	0.652248
C	-4.253535	-1.514870	-0.103228
C	-2.875380	-1.389266	0.090451
C	-1.930394	-2.283536	-0.419639
C	-2.403610	-3.336086	-1.213709
H	-1.701030	-4.044943	-1.636842
C	-3.768670	-3.460999	-1.473658
H	-4.124068	-4.271603	-2.102534
C	-4.685845	-2.565212	-0.916473
H	-5.743655	-2.695951	-1.116370
C	-5.263457	-1.028865	2.123865
H	-4.275205	-1.066763	2.591669
H	-5.703526	-2.031385	2.162564
H	-5.890299	-0.345148	2.706815
C	-6.577132	-0.481585	0.057236
H	-7.210958	0.193833	0.639021
H	-7.051051	-1.466903	0.090771
H	-6.568254	-0.136099	-0.981128
C	0.142196	3.492467	0.637652
C	0.923715	4.122915	1.613216
H	1.080576	3.643113	2.573282

C	1.512180	5.360917	1.347978
H	2.123273	5.838497	2.108554
C	1.321965	5.980411	0.112363
H	1.784768	6.941508	-0.092707
C	0.539063	5.357723	-0.863520
H	0.391956	5.831946	-1.829700
C	-0.042478	4.118529	-0.606439
H	-0.641601	3.631006	-1.369099
C	-0.212357	1.401074	2.616646
C	0.928020	0.661324	2.958838
H	1.600304	0.320372	2.179281
C	1.189881	0.352503	4.292607
H	2.069852	-0.231379	4.540739
C	0.309539	0.765284	5.293037
H	0.508186	0.513383	6.331144
C	-0.832730	1.495799	4.957900
H	-1.522054	1.816993	5.734080
C	-1.092231	1.816515	3.625929
H	-1.980630	2.384723	3.368278
C	0.663911	-3.362106	-0.964690
C	0.813075	-4.633754	-0.392914
H	0.449536	-4.822289	0.611427
C	1.447982	-5.650057	-1.105203
H	1.570531	-6.629528	-0.651429
C	1.925927	-5.411490	-2.396669
H	2.423310	-6.205216	-2.947056
C	1.765745	-4.151853	-2.975031
H	2.139365	-3.955300	-3.975853
C	1.143189	-3.129956	-2.258657
H	1.038988	-2.144250	-2.693115
C	0.074998	-2.425498	1.677452
C	1.384794	-2.672656	2.118143
H	2.211558	-2.630959	1.420838
C	1.630168	-2.987767	3.452225
H	2.650781	-3.181735	3.767045
C	0.577380	-3.040670	4.367719
H	0.770292	-3.276461	5.410658
C	-0.724569	-2.787708	3.936737
H	-1.550502	-2.826476	4.641446
C	-0.977966	-2.489079	2.597373
H	-1.997576	-2.315602	2.277836
O	-2.386225	-0.337403	0.833895
P	-0.505134	1.794930	0.848874
P	-0.146792	-1.970966	-0.091382

Cu	0.603689	0.205283	-0.468306
H	1.569290	-0.521286	-3.924300
C	0.802385	0.106401	-3.465286
C	1.331532	1.034836	-2.457248
H	2.179961	1.613533	-2.833888
H	0.603167	1.769963	-2.103578
C	-0.477467	-0.162590	-3.821681
C	-0.791430	-1.362142	-4.688369
H	0.122954	-1.774643	-5.128171
H	-1.264402	-2.172418	-4.113162
H	-1.481266	-1.108018	-5.503150
C	-1.632777	0.643172	-3.368315
C	-1.584472	2.051168	-3.366197
C	-2.849444	0.035779	-3.011030
C	-2.691103	2.814156	-3.000876
H	-0.674535	2.542559	-3.694329
C	-3.959335	0.800661	-2.655021
H	-2.929801	-1.045992	-3.008642
C	-3.888820	2.192357	-2.642469
H	-2.626157	3.899451	-3.017316
H	-4.884232	0.299680	-2.388300
H	-4.751871	2.784279	-2.356459
Si	3.007817	0.151347	-1.156975
H	2.169252	0.632972	0.147451
H	3.274580	-0.619343	-2.438586
C	3.847780	-1.160382	-0.023932
C	4.199171	-0.838352	1.298813
C	4.099396	-2.468174	-0.460697
C	4.786147	-1.775611	2.147847
H	4.008200	0.169643	1.664889
C	4.651794	-3.429906	0.391749
H	3.840726	-2.745125	-1.479334
C	5.000931	-3.083553	1.698023
H	5.067150	-1.496439	3.161013
H	4.806814	-4.445804	0.036625
H	5.438731	-3.824719	2.362154
C	4.131345	1.667604	-1.161201
C	3.667745	2.926428	-0.746695
C	5.471268	1.559382	-1.565190
C	4.508218	4.040481	-0.741604
H	2.636938	3.036782	-0.424814
C	6.319282	2.668226	-1.560315
H	5.858549	0.593174	-1.881648
C	5.837703	3.912904	-1.148903

H	4.122437	5.003131	-0.417220
H	7.354421	2.562400	-1.875727
H	6.496364	4.777885	-1.144446

23-ts

B3LYP-D3(BJ) SCF energy: -3601.51895406 a.u.
B3LYP-D3(BJ) enthalpy: -3600.478119 a.u.
B3LYP-D3(BJ) free energy: -3600.636902 a.u.
M06-L SCF energy in solution: -3602.79034334 a.u.
M06-L enthalpy in solution: -3601.749508 a.u.
M06-L free energy in solution: -3601.908291 a.u.
Imaginary frequency: -46.8444 cm^{-1}

Cartesian coordinates

ATOM	X	Y	Z
C	1.680677	-2.476984	0.065129
C	1.651104	-3.866811	-0.074843
H	0.940943	-4.447548	0.502054
C	2.495778	-4.490274	-0.992872
H	2.460716	-5.568780	-1.110060
C	3.368346	-3.737204	-1.780379
H	4.002893	-4.245340	-2.497587
C	3.437022	-2.344465	-1.654600
C	2.587907	-1.754455	-0.719622
C	4.422141	-1.444475	-2.404851
C	3.759454	-0.078897	-2.616762
C	2.900296	0.402878	-1.626253
C	2.309835	1.673834	-1.666582
C	2.601415	2.493706	-2.763068
H	2.147621	3.475857	-2.832677
C	3.445271	2.037947	-3.774351
H	3.658687	2.675390	-4.626761
C	4.014373	0.765629	-3.701831
H	4.666184	0.433232	-4.501658
C	5.663025	-1.234098	-1.495439
H	5.373984	-0.794345	-0.535553
H	6.380486	-0.562636	-1.979990
H	6.154584	-2.193038	-1.297720
C	4.870855	-2.072985	-3.731352
H	5.365110	-3.032531	-3.556280
H	5.600826	-1.434135	-4.236275
H	4.023039	-2.235140	-4.404197
C	-0.556356	-2.734683	1.921056
C	-0.452831	-2.999428	3.294453
H	0.325797	-2.527916	3.883238

C	-1.366416	-3.854624	3.909540
H	-1.285206	-4.051081	4.975178
C	-2.380636	-4.455546	3.159616
H	-3.092467	-5.119326	3.642754
C	-2.477096	-4.203591	1.789818
H	-3.249685	-4.668091	1.187209
C	-1.574269	-3.339786	1.170038
H	-1.670778	-3.139101	0.110401
C	1.567909	-0.867860	2.443888
C	1.072745	0.202695	3.198570
H	0.130524	0.663104	2.929297
C	1.786391	0.673523	4.297474
H	1.389806	1.501698	4.874985
C	3.014468	0.101025	4.631761
H	3.577690	0.478719	5.480575
C	3.523751	-0.951347	3.868430
H	4.481225	-1.397004	4.123984
C	2.799507	-1.442167	2.781708
H	3.185998	-2.273438	2.200251
C	0.301344	3.653938	-1.012924
C	0.903504	4.911582	-0.846319
H	1.848067	4.994289	-0.318240
C	0.284694	6.055563	-1.348402
H	0.755280	7.025454	-1.213615
C	-0.935249	5.955756	-2.023917
H	-1.414214	6.849963	-2.413005
C	-1.532250	4.707185	-2.199709
H	-2.475793	4.612904	-2.729792
C	-0.919905	3.559318	-1.694608
H	-1.389891	2.592559	-1.860826
C	2.020068	2.664378	1.065454
C	1.382848	3.476345	2.017150
H	0.356061	3.789281	1.859369
C	2.068147	3.903325	3.151701
H	1.567871	4.543486	3.873206
C	3.390691	3.507505	3.361704
H	3.924275	3.836264	4.248862
C	4.020008	2.679466	2.432478
H	5.044187	2.357102	2.595381
C	3.342002	2.261603	1.287567
H	3.845095	1.626264	0.568141
O	2.592544	-0.383799	-0.535395
P	0.519197	-1.513454	1.093967
P	1.061241	2.108881	-0.393738

Cu	-0.326034	0.247359	-0.125562
C	-1.613852	-1.234125	-2.666646
C	-2.443979	0.985199	-3.477825
C	-1.899394	-2.602898	-2.340794
C	-0.880521	-3.603679	-2.290681
C	-3.213606	-3.072599	-2.022092
C	-1.144822	-4.920499	-1.933249
H	0.136243	-3.342207	-2.560547
C	-3.467182	-4.393588	-1.677833
H	-4.047184	-2.378597	-2.048251
C	-2.439133	-5.344256	-1.613787
H	-0.321453	-5.632590	-1.915163
H	-4.490319	-4.688192	-1.448683
H	-2.643032	-6.375175	-1.340096
C	-0.194714	-0.756980	-2.804485
H	0.146312	-0.633328	-3.849065
H	-0.090763	0.260340	-2.378058
H	0.528536	-1.404567	-2.305386
H	-1.464619	1.396375	-3.702855
H	-3.299456	1.597170	-3.745816
C	-2.617216	-0.307500	-3.049882
H	-3.651227	-0.643972	-2.982105
Si	-2.972998	0.148721	0.091074
H	-3.171573	-1.311471	0.055118
H	-1.876508	0.813375	-0.733214
C	-2.561099	0.650957	1.861656
C	-2.133174	1.962126	2.149240
C	-2.663511	-0.258371	2.928902
C	-1.837146	2.355163	3.455197
H	-2.033603	2.684822	1.341455
C	-2.356831	0.127023	4.234680
H	-2.971263	-1.281787	2.735987
C	-1.949735	1.435860	4.501662
H	-1.514172	3.372922	3.657581
H	-2.428599	-0.597970	5.040377
H	-1.713019	1.736549	5.518657
C	-4.514018	0.994775	-0.537191
C	-5.701152	0.262236	-0.685634
C	-4.525430	2.360324	-0.865779
C	-6.869178	0.874136	-1.145778
H	-5.709981	-0.800630	-0.454945
C	-5.690104	2.977106	-1.318088
H	-3.613318	2.944766	-0.783344
C	-6.864591	2.233043	-1.460548

H	-7.777644	0.289785	-1.264205
H	-5.682290	4.034588	-1.569540
H	-7.771023	2.711290	-1.821935

***E*-3a**

B3LYP-D3(BJ) SCF energy: -1141.18015863 a.u.
B3LYP-D3(BJ) enthalpy: -1140.784857 a.u.
B3LYP-D3(BJ) free energy: -1140.860822 a.u.
M06-L SCF energy in solution: -1141.20513376 a.u.
M06-L enthalpy in solution: -1140.809832 a.u.
M06-L free energy in solution: -1140.885797 a.u.

Cartesian coordinates

ATOM	X	Y	Z
C	-1.047206	-1.106045	0.858207
H	-1.279698	-0.275938	1.520500
C	-2.033438	-1.571489	0.065951
C	-3.380431	-0.947502	0.097399
C	-3.548151	0.420065	0.386159
C	-4.532539	-1.701485	-0.185901
C	-4.814387	0.998256	0.419669
H	-2.674408	1.041161	0.553989
C	-5.800778	-1.122756	-0.152881
H	-4.439615	-2.757092	-0.420900
C	-5.950016	0.229994	0.153232
H	-4.913120	2.058010	0.639894
H	-6.674425	-1.732695	-0.366872
H	-6.937184	0.683004	0.173576
C	-1.839490	-2.742113	-0.871330
H	-0.783037	-2.963218	-1.035312
H	-2.310918	-3.654491	-0.481419
H	-2.294256	-2.540049	-1.847614
C	0.389637	-1.537958	0.882453
H	0.516857	-2.602221	0.656199
H	0.823694	-1.363424	1.874054
Si	1.337429	-0.497416	-0.403315
H	0.899571	-0.968136	-1.748042
C	3.193856	-0.685451	-0.189442
C	3.739770	-1.736595	0.566766
C	4.083725	0.223251	-0.791216
C	5.120322	-1.880366	0.714740
H	3.081210	-2.453751	1.050736
C	5.464004	0.085030	-0.646887
H	3.691996	1.056083	-1.370310
C	5.984703	-0.968885	0.107085

H	5.520578	-2.700182	1.305172
H	6.133201	0.799230	-1.119134
H	7.059730	-1.077160	0.222473
C	0.841740	1.305675	-0.245517
C	-0.326516	1.774013	-0.873618
C	1.562658	2.203102	0.560156
C	-0.764734	3.086511	-0.695808
H	-0.906307	1.102547	-1.501351
C	1.130865	3.518675	0.738597
H	2.475645	1.872570	1.049116
C	-0.035534	3.961349	0.112788
H	-1.671876	3.425943	-1.188592
H	1.703541	4.197198	1.365247
H	-0.373542	4.984772	0.251376

***Z*-3a**

B3LYP-D3(BJ) SCF energy: -1141.18251255 a.u.

B3LYP-D3(BJ) enthalpy: -1140.787566 a.u.

B3LYP-D3(BJ) free energy: -1140.862688 a.u.

M06-L SCF energy in solution: -1141.20415541 a.u.

M06-L enthalpy in solution: -1140.809209 a.u.

M06-L free energy in solution: -1140.884331 a.u.

Cartesian coordinates

ATOM	X	Y	Z
C	1.138538	1.190405	-2.144614
H	1.104841	1.050798	-3.225638
C	2.304271	0.947611	-1.521643
C	2.467234	1.108343	-0.048827
C	2.324768	2.366223	0.556314
C	2.779568	0.007922	0.761003
C	2.474821	2.517655	1.934856
H	2.098414	3.227211	-0.066618
C	2.924121	0.156304	2.139438
H	2.871695	-0.975654	0.313246
C	2.773497	1.411144	2.732104
H	2.361121	3.499987	2.385546
H	3.144247	-0.714263	2.751087
H	2.887299	1.526529	3.806487
C	3.523268	0.476361	-2.275872
H	3.325338	0.400904	-3.350112
H	4.372426	1.155102	-2.123784
H	3.844531	-0.511492	-1.919445
C	-0.174824	1.504297	-1.491022

H	-0.741750	2.240059	-2.076347
H	-0.038472	1.912781	-0.485249
Si	-1.196622	-0.094684	-1.345963
H	-1.464771	-0.587701	-2.729155
C	-2.802013	0.231679	-0.425370
C	-3.262851	1.538216	-0.191557
C	-3.581403	-0.836144	0.056367
C	-4.457538	1.772855	0.491459
H	-2.682259	2.387719	-0.542149
C	-4.776118	-0.608417	0.738688
H	-3.243400	-1.858789	-0.093412
C	-5.216737	0.698749	0.956940
H	-4.793562	2.792077	0.662520
H	-5.361771	-1.448334	1.102773
H	-6.146127	0.878670	1.490475
C	-0.218498	-1.431493	-0.462897
C	0.667561	-2.255710	-1.178296
C	-0.303655	-1.607234	0.927959
C	1.441120	-3.219228	-0.530167
H	0.754938	-2.143322	-2.256068
C	0.466840	-2.568749	1.582791
H	-0.981613	-0.986286	1.507425
C	1.340978	-3.377023	0.854467
H	2.117063	-3.848936	-1.102746
H	0.386634	-2.685625	2.660128
H	1.940202	-4.128437	1.361783

3a″
B3LYP-D3(BJ) SCF energy: -1141.16634447 a.u.
B3LYP-D3(BJ) enthalpy: -1140.770925 a.u.
B3LYP-D3(BJ) free energy: -1140.845700 a.u.
M06-L SCF energy in solution: -1141.18559246 a.u.
M06-L enthalpy in solution: -1140.790173 a.u.
M06-L free energy in solution: -1140.864948 a.u.

Cartesian coordinates

ATOM	X	Y	Z
C	0.174564	-2.683449	-0.035984
H	-0.023612	-2.917492	-1.082418
C	-0.409140	-1.380008	0.446843
C	0.927823	-3.538338	0.661275
H	1.181720	-3.381245	1.704529
H	1.327622	-4.435646	0.197332
C	-0.095796	-1.125299	1.934186

H	0.984796	-1.083495	2.109556
H	-0.529968	-0.183227	2.276029
H	-0.514526	-1.926956	2.554580
C	-1.919609	-1.307368	0.187387
C	-2.655201	-0.164302	0.548551
C	-2.625530	-2.372173	-0.393288
C	-4.027002	-0.082664	0.319977
H	-2.155227	0.677639	1.010685
C	-3.999887	-2.292091	-0.625273
H	-2.105990	-3.288246	-0.649118
C	-4.709112	-1.144887	-0.275099
H	-4.561976	0.818150	0.608395
H	-4.514993	-3.136911	-1.074830
H	-5.778587	-1.081411	-0.455123
Si	0.480402	-0.044626	-0.651820
H	0.146405	-0.383620	-2.062400
C	-0.168548	1.685478	-0.322273
C	-0.903864	2.350969	-1.317141
C	-0.000248	2.336772	0.914535
C	-1.452250	3.614152	-1.089868
H	-1.059664	1.870650	-2.279475
C	-0.546887	3.598483	1.148357
H	0.556792	1.851618	1.711633
C	-1.276449	4.239675	0.144772
H	-2.019836	4.107362	-1.874271
H	-0.405976	4.080381	2.112077
H	-1.705185	5.221534	0.325465
C	2.346413	-0.104581	-0.409746
C	3.091593	-1.221366	-0.834986
C	3.050412	0.960777	0.179499
C	4.473858	-1.278701	-0.662649
H	2.587592	-2.059283	-1.304496
C	4.433682	0.906492	0.358505
H	2.517288	1.853278	0.490664
C	5.148620	-0.215898	-0.058939
H	5.025005	-2.152212	-1.000663
H	4.952200	1.743737	0.818126
H	6.225842	-0.259876	0.077353
